# Comparative effectiveness of multiple different treatment regimens for nonalcoholic fatty liver disease with type 2 diabetes mellitus: a systematic review and Bayesian network meta-analysis of randomised controlled trials

**DOI:** 10.1186/s12916-023-03129-6

**Published:** 2023-11-16

**Authors:** Manjun Deng, Yonghao Wen, JingXin Yan, Yichen Fan, Zhixin Wang, Ruixia Zhang, Li Ren, Yinggui Ba, Haijiu Wang, Qian Lu, Haining Fan

**Affiliations:** 1 Department of Hepatopancreatobiliary Surgery, Affiliated Hospital of Qinghai University, Xining, 810001 Qinghai China; 2 Qinghai Research Key Laboratory for Echinococcosis, Xining, 810000 Qinghai China; 3 Department of Interventional Therapy, Affiliated Hospital of Qinghai University, Xining, 810001 Qinghai China; 4 Department of Endocrinology, Affiliated Hospital of Qinghai University, Xining, 810001 Qinghai China; 5 Department of Nephrology, Affiliated Hospital of Qinghai University, Xining, 810001 Qinghai China; 6 Department of Hepatopancreatobiliary Surgery, Tsinghua Changgung Hospital, Tsinghua University, Beijing, 102218 China

**Keywords:** Nonalcoholic fatty liver disease, Diabetes mellitus, type 2, Bayesian network meta-analysis, Systematic review, Weight loss

## Abstract

**Background:**

Nonalcoholic fatty liver disease (NAFLD) and type 2 diabetes mellitus (T2DM) are closely related and mutually contribute to the disease’s development. There are many treatment options available to patients. We provide a comprehensive overview of the evidence on the treatment effects of several potential interventions for NAFLD with T2DM.

**Methods:**

This systematic review and network meta-analysis included searches of PubMed, Embase, Cochrane Library, and Web of Science from inception to June 30, 2023, for randomised controlled trials of treatment of NAFLD with T2DM. We performed Bayesian network meta-analyses to summarise effect estimates of comparisons between interventions. We applied the Grading of Recommendations Assessment, Development, and Evaluation (GRADE) frameworks to rate all comparative outcomes’ certainty in effect estimates, categorise interventions, and present the findings. This study was registered with PROSPERO, CRD42022342373.

**Results:**

Four thousand three hundred and sixty-nine records were retrieved from the database and other methods, of which 24 records were eligible for studies enrolling 1589 participants. Eight clinical indicators and 14 interventions were finally in focus. Referring to the lower surface under the cumulative ranking curves (SUCRA) and the league matrix table, exenatide and liraglutide, which are also glucagon-like peptide-1 receptor agonists (GLP-1RAs), showed excellent potential to reduce liver fat content, control glycemia, reduce body weight, and improve liver function and insulin resistance. Exenatide was more effective in reducing glycated haemoglobin (HbA_1c_) (mean difference (MD) 0.32, 95%CI 0.12 to 0.52), lowering BMI (MD 0.81, 95%CI 0.18 to 1.45), and lowering alanine transaminase (ALT) (MD 10.96, 95%CI 5.27 to 16.66) compared to liraglutide. However, this evidence was assessed as low certainty. Omega-3 was the only intervention that did not have a tendency to lower HbA_1c_, with standard-treatment (STA-TRE) as reference (MD − 0.17, 95%CI − 0.42 to 0.07). Glimepiride is the only intervention that causes an increase in ALT levels, with standard-treatment (STA-TRE) as reference (MD − 11.72, 95%CI − 17.82 to − 5.57). Based on the available evidence, the treatment effects of pioglitazone, dapagliflozin, and liraglutide have a high degree of confidence.

**Conclusions:**

The high confidence mandates the confident application of these findings as guides for clinical practice. Dapagliflozin and pioglitazone are used for glycaemic control in patients with NAFLD combined with T2DM, and liraglutide is used for weight loss therapy in patients with abdominal obesity. The available evidence does not demonstrate the credibility of the effectiveness of other interventions in reducing liver fat content, visceral fat area, ALT, and insulin resistance. Future studies should focus on the clinical application of GLP-1Ras and the long-term prognosis of patients.

**Supplementary Information:**

The online version contains supplementary material available at 10.1186/s12916-023-03129-6.

## Background

Nonalcoholic fatty liver disease (NAFLD) is the most common worldwide cause of chronic liver disease [1, 2]. NAFLD encompasses a spectrum of liver diseases characterised by the progression of fat accumulation in the liver, leading to inflammatory damage and fibrosis of hepatocytes [3, 4]. NAFLD has been shown to be closely associated with hepatocellular carcinoma, cardiovascular disease, and chronic kidney disease [5–9].

Some studies have shown that the global prevalence of NAFLD among patients with type 2 diabetes mellitus (T2DM) was 55.5% [10, 11]. Insulin resistance in T2DM leads to increased levels of free fatty acids in the circulation, which increases the lipotoxic and metabolic load on the liver [12, 13]. T2DM and NAFLD are closely related and mutually contribute to the development of each other.

In the current studies, hypoglycemic therapy was a potential treatment for NAFLD with T2DM [14, 15]. An increasing number of studies have used different hypoglycemic agents in treating NAFLD with T2DM, with drugs such as sodium-dependent glucose transporters 2 inhibitors (SGLT-2Is) and glucagon-like peptide-1 receptor agonists (GLP-1RAs), and showing excellent therapeutic effects [16, 17]. Previously, some researchers conducted meta-analyses comparing two or more interventions [18–20]. The results of these studies were not uniform, and all hypoglycemic drugs appear to be recommendable. The utility of these meta-analyses is limited. First, traditional meta-analyses cannot assess treatment modalities that have not been directly compared, nor can they rank the strengths and weaknesses of several treatment modalities. Secondly, these studies neglected to assess the certainty of the evidence, which can lead to interventions with high statistical effect sizes but low evidence ratings being recommended in the first place, which is not rigorous.

Therefore, we attempted to compare the effects of potentially therapeutically effective drugs for the treatment of NAFLD with T2DM through a network meta-analysis based on a Bayesian model and to evaluate the certainty of evidence according to the latest methods recommended by the Grading of Recommendations Assessment, Development, and Evaluation (GRADE) working group. This study will provide a more accurate evidence-based medical reference for future clinical decision-making and development of the guidelines.

## Methods

This study was reported according to the Preferred Reporting Items for Systematic Reviews and Systematic Reviews and Meta-Analyses (PRISMA) and PRISMA-2020 guidelines and the extension statement for network meta-analysis (PRISMA-NMA) [21] (Additional file 1: Table S1) and registered at http://www.crd.york.ac.uk/PROSPERO/ (Review registry CRD42022342373).

### Literature search

Three-step search by 3 independent researchers for all potentially included studies from inception to June 30, 2023. Firstly, we searched English databases, including PubMed, Embase, Cochrane Library, and Web of Science. Additional file 1: Table S2 provides a search strategy for the corresponding databases. Second, to maximise the availability of the data, we searched various Clinical Trial Registry websites and specialised journals (Additional file 1: Table S3) for ongoing and unpublished trials and potential trials; relevant systematic reviews and guideline references were also considered. EndNote 21 software managed the references and eliminated duplicated literature from different databases.

### Selection criteria and data extraction

We used the PRISMA2020 flow diagram for literature screening. Three independent reviewers read the title, abstract, and full text to determine if the PICO principles-based literature inclusion–exclusion criteria met the inclusion criteria (Additional file 1: Table S4). The fourth reviewer pooled the three results, and the fifth reviewer resolved the divergence points that arose jointly. We have developed standardised extraction data forms based on the Cochrane Handbook for Systematic Reviews of Interventions [22], which includes, among other things, the name of the author, the year of publication, the region of the subject population, the intervention, the measure, the duration of the intervention, and all clinical indicators that were recorded. If the amount of change from baseline to end of follow-up was not reported, the formula recommended in Cochrane Handbook (16.1.3.2 Imputing standard deviations for changes from baseline) was used to calculate (https://training.cochrane.org/handbook). Two independent researchers entered the relevant data into a spreadsheet, which a third researcher finally summarised to detect possible clerical errors in data entry. The panel judged the following outcomes as crucial: change in liver fat content, change in glycated haemoglobin (HbA_1C_), change in body mass index (BMI), and waist circumference. The panel judged the following outcomes as important but not crucial: alanine transaminase (ALT), insulin resistance [23], visceral adipose tissue area (VATA) [24], and subcutaneous adipose tissue area (SATA) [25].

### Quality assessment

The quality of the included studies was evaluated using the Cochrane Risk of Bias Tool (2.0) for RCTs [26], which is based on the following five dimensions: risk of bias arising from the randomisation process, risk of bias owing to deviations from the intended interventions, risk of bias from missing outcome data, risk of bias in the measurement of the outcome, and risk of bias in the selection of the reported result.

### Statistical analysis

Stata 16.0 software created a network map that visually represents the relationship between the comparisons of different interventions. The circle size indicates the sample size of the corresponding intervention, and the width of the line indicates the number of studies compared to the corresponding intervention. Statistical analysis was performed by R software (4.2.1) calling JAGS software (4.3.1) in a Bayesian framework using the Markov chain-Monte Carlo method for direct and indirect comparisons. The R packages “gemtc”, “rjags”, “openxlsx”, “ggplot2”, and “forestplot” were used for statistical analysis and data output. Parameter settings: number of chains was 6, initial value was 2.5, amount of adaptation (or tuning) iterations was 50,000, amount of simulation iterations was 200,000, thinning factor was 10. Relative treatment effects of continuous variables as effect sizes mean difference (MD) and 95% confidence interval (95% CI). Iterative model convergence was assessed visually and quantitatively using trajectory density plots, Brooks-Gelman-Rubin diagnostic plots, and potential scale reduction factor (PSRF). PSRF was limited to 1.00 ~ 1.05 to reach satisfactory convergence. Statistical heterogeneity was assessed using the *Q* test and the statistic index (*I*^2^) to assess the heterogeneity of the two-arm and reticulated comparisons. Considerable heterogeneity was considered to exist when *I*^2^ values were greater than 50%, which initiated sensitivity analysis to identify the source [27]. Bayesian network meta-analysis provided an overall ranking probability for each intervention, making it possible to rank each intervention from best to worst. The ranking order was visualised by surface under the cumulative ranking curves (SUCRA); the higher the SUCRA, the higher the likelihood that the intervention was in the top ranking. Inconsistency tests were performed for comparisons of closed loops using the node-splitting models, and when *P* < 0.05 was considered inconsistent, finally, this part of the result is visualised using a forest plot [28]. Of course, this part of the results was also used to assess the certainty in effect estimates of interventions from the network meta-analysis. Egger’s test and funnel plot were employed to quantitatively assess publication bias (*P* < 0.05 was considered to have publication bias) [29].

### Assessment of certainty of evidence

We set up a panel to summarise all comparative outcomes’ certainty in effect estimates using the Grading of Recommendations Assessment, Development, and Evaluation (GRADE) working group recommended method to avoid inflated recommendations for interventions that perform well statistically but have a low level of evidence [30–33]. The assessment of certainty in effect estimates for direct comparison evidence includes the risk of bias, inconsistency, indirectness, imprecision, and other considerations. The certainty in effect estimates of indirect comparative evidence was primarily concerned with incoherence (baseline demographic and clinical characteristics of participants). The certainty in effect estimates of the network comparison evidence was based on direct and indirect comparison results and will be downgraded if there is inconsistency and imprecision. The final certainty in effect estimates of the network comparison was presented in the league matrix table. High certainty, moderate certainty, low certainty, and very low certainty were included. The specific meanings are presented in Additional file 1: Table S5.

Finally, conclusions were drawn from the network meta-analysis through the Minimising Contextual Framework approach [34]. The minimally contextualised framework is based on two principles: interventions should be grouped in categories, from the most to the least effective or harmful, and the judgements that place interventions in such categories should simultaneously consider the estimates of effect, the certainty of the evidence, and the rankings. The specific process refers to the steps recommended by the GRADE working group. The conclusions mainly include most effective, effective, and ineffective of high confidence and probably most effective, probably effective, and probably ineffective of low confidence.

## Result

### Summary of included articles

**Fig. 1:**
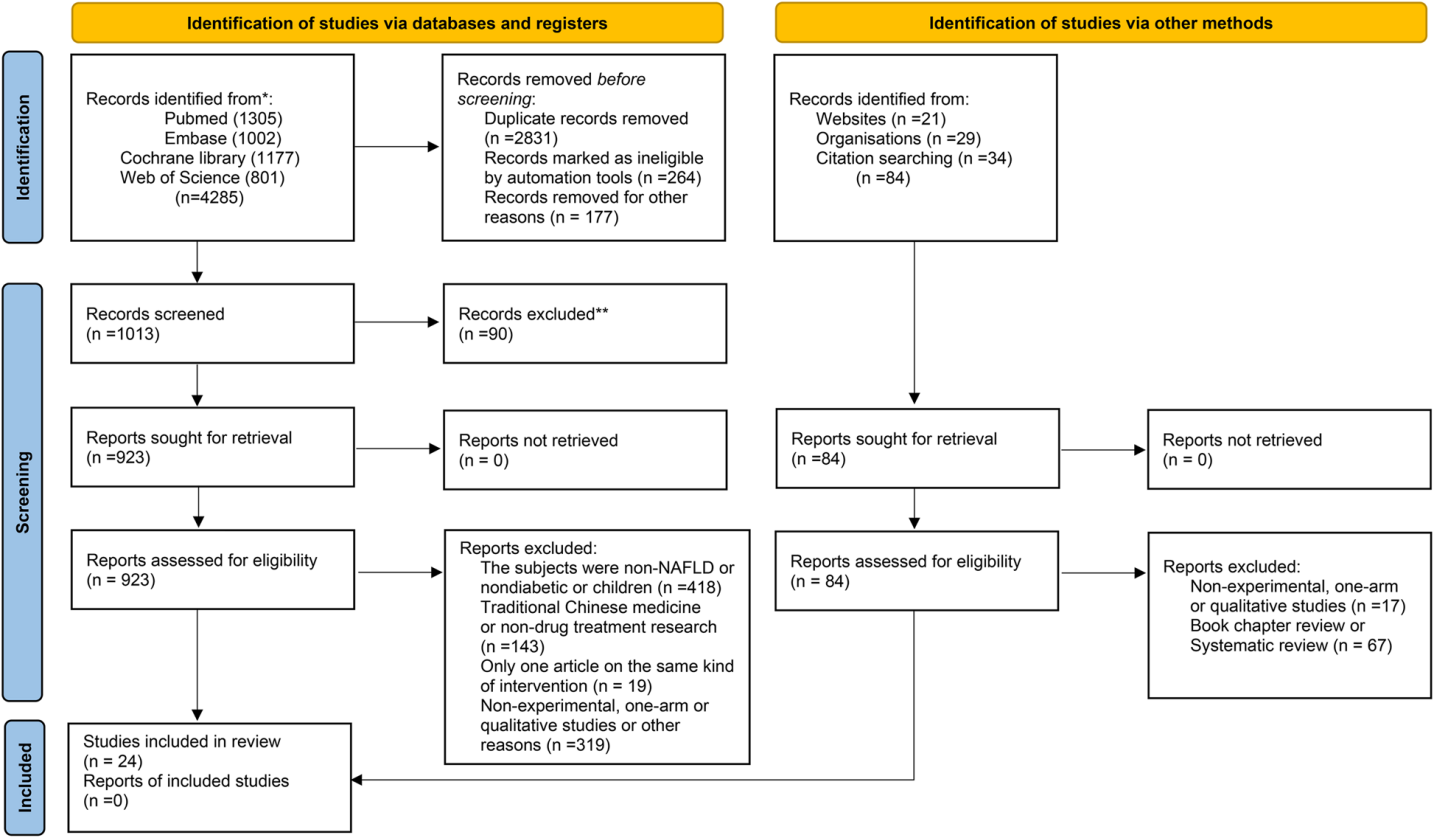
PRISMA 2020 flow diagram for new systematic reviews which included searches of databases, registers, and other sources

A total of 4369 records were retrieved from the database and other methods, and 923 studies were considered eligible for the full-text review by removing duplicate records and irrelevant articles. Some of the interventions were reported in only one literature, so these interventions were excluded: obeticholic acid [35], carnitine-orotate complex [36], vitamin D supplementation [37], lobeglitazone [38], luseogliflozin [39], diacerein [40], canagliflozin [41], vitamin E [42], linagliptin [43], coffee components [44], berberine ursodeoxycholate [45], dulaglutide [46], rosiglitazone [47], and PF-06835919 [48]. The final 24 literatures [49–72] were included in this study based on the exclusion criteria. The reasons for the exclusion of all excluded literature are reported in Additional file 1: Table S6. Study selection was graphically illustrated using the PRISMA 2020 flow diagram (Fig. 1). A total of 1589 patients and 14 interventions were included: exenatide, metformin, omega 3 fatty acids (omega 3), insulin glargine, liraglutide, gliclazide, pioglitazone, ipragliflozin, dapagliflozin, empagliflozin, tofogliflozin, glimepiride, sitagliptin, and standard-treatment (STA-TRE). The partially included original study collected the glycaemic control regimen of all enrolled patients prior to participation in the study and randomly divided them into two groups, one group continuing with the glycaemic control regimen prior to participation in the study and one group using the study medication. Maintenance of previous treatment measures is defined as standard treatment. The baseline characteristics of the included studies are presented in Table 1.

**Table 1:** Baseline characteristics of included studies

Author	Year	Region	Race	Follow-up	Number of patients	Intervention	BMI of baseline (mean)	Glycated haemoglobin of baseline	Diet control and exercise
Fan, H [49]	2013	Beijing, China	Asian	12 weeks	Group 1: 49	Group 1: Exenatide 20 μg/d	Group 1: 28.18	7–9%	Yes
					Group 2: 68	Group 2: Metformin 1 g/d	Group 2: 27.61		
Dasarathy, S [50]	2015	America	Caucasian	48 weeks	Group 1: 18	Group 1: Omega 3 3.6 g/d	Group 1: 34.8	< 8.5%	Yes
					Group 2: 19	Group 2: Standard treatment	Group 2: 35.7		
Feng, W [68]	2017	Nanjing, China	Asian	24 weeks	Group 1: 29	Group 1: Liraglutide 1.8 mg/d	Group 1: 28.12	7–14%	Yes
					Group 2: 29	Group 2: Gliclazide 30–120 mg/d	Group 2: 27.85		
					Group 3: 29	Group 3: Metformin 2 g/d	Group 3: 26.82		
Ito, D [51]	2017	Saitama, Japan	Asian	24 weeks	Group 1: 34	Group 1: Pioglitazone 15 or 30 mg/d	Group 1: 29.9	7–11%	No
					Group 2: 32	Group 2: Ipragliflozin 50 mg/d	Group 2: 30.7		
Eriksson, JW [53]	2018	Sweden	Caucasian	12 weeks	Group 1: 19	Group 1: Standard treatment	Group 1: 30.3	NA	No
					Group 2: 15	Group 2: Omega 3 4 g/d	Group 2: 33.0		
					Group 3: 19	Group 3: Dapagliflozin 10 mg/d	Group 3: 30.5		
Shimizu, M [54]	2019	America	Hispanic	24 weeks	Group 1: 33	Group 1: Dapagliflozin 5 mg/d	Group 1: 27.6	6–12%	No
					Group 2: 24	Group 2: Standard treatment	Group 2: 28.7		
Feng, W H [55]	2019	China	Asian	24 weeks	Group 1: 29	Group 1: Liraglutide 1.8 mg/d	Group 1: 28.1	7–14%	Yes
					Group 2: 29	Group 2: Metformin 2 g/d	Group 2: 26.8		
					Group 3: 27	Group 3: Gliclazide 30–120 mg/d	Group 3: 27.5		
Aso, Y [69]	2019	Japan	Asian	24 weeks	Group 1: 33	Group 1: Dapagliflozin 5 mg/d	Group 1: 27.6	6–12%	No
					Group 2: 24	Group 2: Standard treatment	Group 2: 28.7		
Liu, L [56]	2020	Shanghai, China	Asian	24 weeks	Group 1: 35	Group 1: Exenatide 20 μg/d	Group 1: 28.49	7–10%	No
					Group 2: 36	Group 2: Insulin glargine 0.1–0.3 IU/kg	Group 2: 27.84		
Zhang, L Y[57]	2020	Hispanic	Asian	24 weeks	Group 1: 30	Group 1: Liraglutide 1.2 mg/d	Group 1: 27.6	7–14%	Yes
					Group 2: 30	Group 2: Pioglitazone 30 mg/d	Group 2: 27.1		
Kinoshita, T [59]	2020	Japan	Asian	28 weeks	Group 1: 33	Group 1: Pioglitazone 7.5–15 mg/d	Group 1: 28.7	≥ 6.5%	No
					Group 2: 32	Group 2: Dapagliflozin 5 mg/d	Group 2: 29.5		
					Group 3: 33	Group 2: Glimepiride 0.5– 1 mg/d	Group 3: 28.4		
Guo, W [60]	2020	Fujian, China	Asian	26 weeks	Group 1: 30	Group 1: Insulin glargine 10 IU/d	Group 1: 28.3	≥ 6.5%	Yes
					Group 2: 31	Group 2: Liraglutide 1.8 mg/d	Group 2: 29.2		
					Group 3: 30	Group 3: Metformin	Group 3: 28.6		
Sangouni, AA [61]	2021	Yazd, Iran	Asian	12 weeks	Group 1: 28	Group 1: Omega 3 2 g/d	Group 1: 30.26	NA	Yes
					Group 2: 28	Group 2: Metformin	Group 2: 29.8		
Tian, F [63]	2018	China	Asian	12 weeks	Group 1: 52	Group 1: Liraglutide 0.6–1.2 mg/d	Group 1: 28.18	< 9%	Yes
					Group 2: 75	Group 2: Metformin 1–1.5 g/d	Group 2: 27.61		
Bando, Y [70]	2017	Japan	Asian	12 weeks	Group 1: 40	Group 1: Ipragliflozin 50 mg/d	Group 1: 27.8	7–10%	Yes
					Group 2: 22	Group 2: Standard treatment	Group 2: 27.3		
Cho, K Y [71]	2021	Japan	Asian	24 weeks	Group 1: 27	Group 1: Dapagliflozin 5 mg/d	NA	6.5–8.5%	No
					Group 2: 26	Group 2: Pioglitazone 30 mg/d			
Phrueksotsai, S [62]	2021	Thailand	Asian	12 weeks	Group 1: 18	Group 1: Dapagliflozin 10 mg/d	Group 1: 29.6	7–10%	No
					Group 2: 20	Group 2: Standard treatment	Group 2: 28.8		
Kuchay, MS [52]	2018	India	Asian	20 weeks	Group 1: 20	Group 1: Standard treatment	Group 1: 29.4	7–10%	No
					Group 2: 22	Group 2: Empagliflozin10mg/d	Group 2: 30.0		
Chehrehgosha, H [72]	2021	Iran	Asian	24 weeks	Group 1: 37	Group 1: Standard treatment	Group 1: 30.2	7–10%	Yes
					Group 2: 35	Group 2: Empagliflozin 10 mg/d	Group 2: 30.9		
					Group 3: 26	Group 3: Pioglitazone 30 mg/d	Group 3: 29.4		
Deng, XL [67]	2017	China	Asian	26 weeks	Group 1: 36	Group 1: Sitagliptin 50 mg/d	Group 1: 24.1	NA	Yes
					Group 2: 36	Group 2: Standard treatment	Group 2: 23.9		
Yoneda, M [58]	2021	Japan	Asian	24 weeks	Group 1: 19	Group 1: Pioglitazone 15–30 mg/d	Group 1: 30.8	≥ 6.5%	Yes
					Group 2: 21	Group 2: Tofogliflozin 20 mg/d	Group 2: 29.4		
Takeshita, Y [64]	2022	Japan	Asian	48 weeks	Group 1: 20	Group 1: Tofogliflozin 20 mg/d	Group 1: 31.0	7.3–8.8%	Yes
					Group 2: 20	Group 2: Glimepiride 0.5 mg/d	Group 2: 32.0		
Hiruma, S [65]	2023	Japan	Asian	12 weeks	Group 1: 23	Group 1: Empagliflozin10mg/d	Group 1: 30.6	< 10%	No
					Group 2: 19	Group 2: Sitagliptin 100 mg/d	Group 2: 28.6		
Nar, A [66]	2009	Turkey	Caucasian	24 weeks	Group 1: 19	Group 1: Metformin 1.7 g/d	Group 1: 31.0	NA	Yes
					Group 2: 15	Group 2: Standard treatment	Group 2: 33.7		

**Fig. 2:**
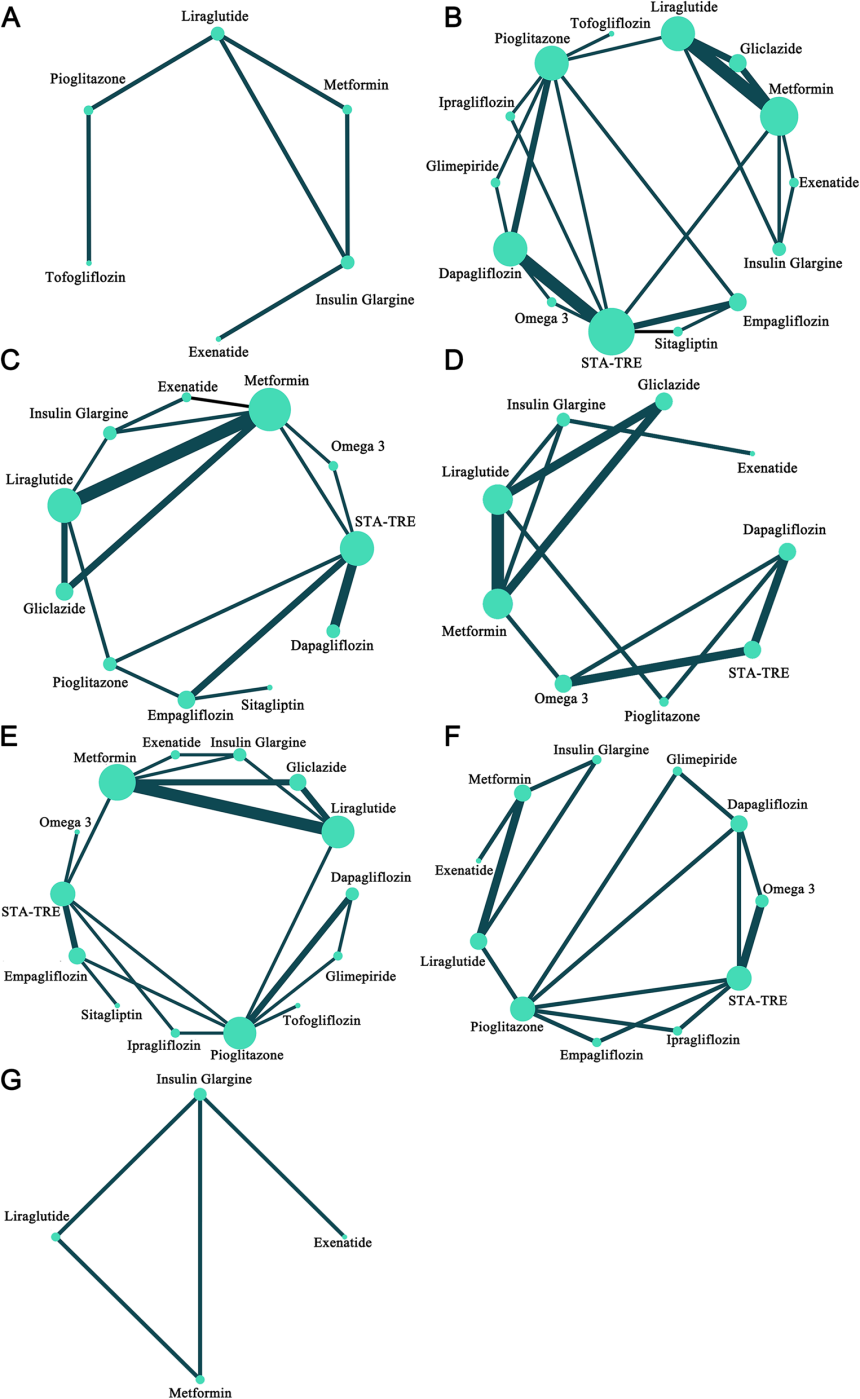
The network maps. **A** Liver fat content. **B** HbA_1c_. **C** BMI. **D** Waist circumference. **E** ALT. **F** Insulin resistance. **G** VATA and SATA. The size of the nodes is proportional to the sample size, the width of the solid line is proportional to the number of studies, and interventions connected by a solid line imply the existence of a direct comparison

A total of 7 clinical indicators were of interest. We showed relationships between interventions through network maps (Fig. 2). We assessed the certainty in effect estimates for each comparison according to the methods recommended by the GRADE group, as the certainty of the evidence may be downgraded for a variety of reasons, and this process was described in Additional file 1: Table S7, where the results were coloured differently in the league matrix table.

### Quality assessment of the included studies

We assessed the risk of bias for each included study using the Cochrane Risk of Bias Tool (2.0) for RCTs. Seven studies [49, 54, 63, 66, 69, 71, 72] did not have a clear process for random sequence generation, seventeen studies [49, 51, 52, 54–60, 63–66, 68, 69, 71] had inadequate descriptions of allocation concealment, and nine studies [50, 52, 54, 55, 57, 68, 69, 71, 72] had changes from baseline in clinical indicators calculated by a formula and were assessed as having an unclear risk of bias for these reasons. Nine studies [49, 55, 58, 63–68] were assessed as having a high risk of bias because of differences in the mode of administration (e.g. subcutaneous exenatide injection versus oral metformin), which made double-blinding difficult to achieve. A visualisation is presented in Additional file 1: Fig. S1.

## Results of Bayesian network meta-analysis and ranking

### Liver fat content

**Fig. 3:**
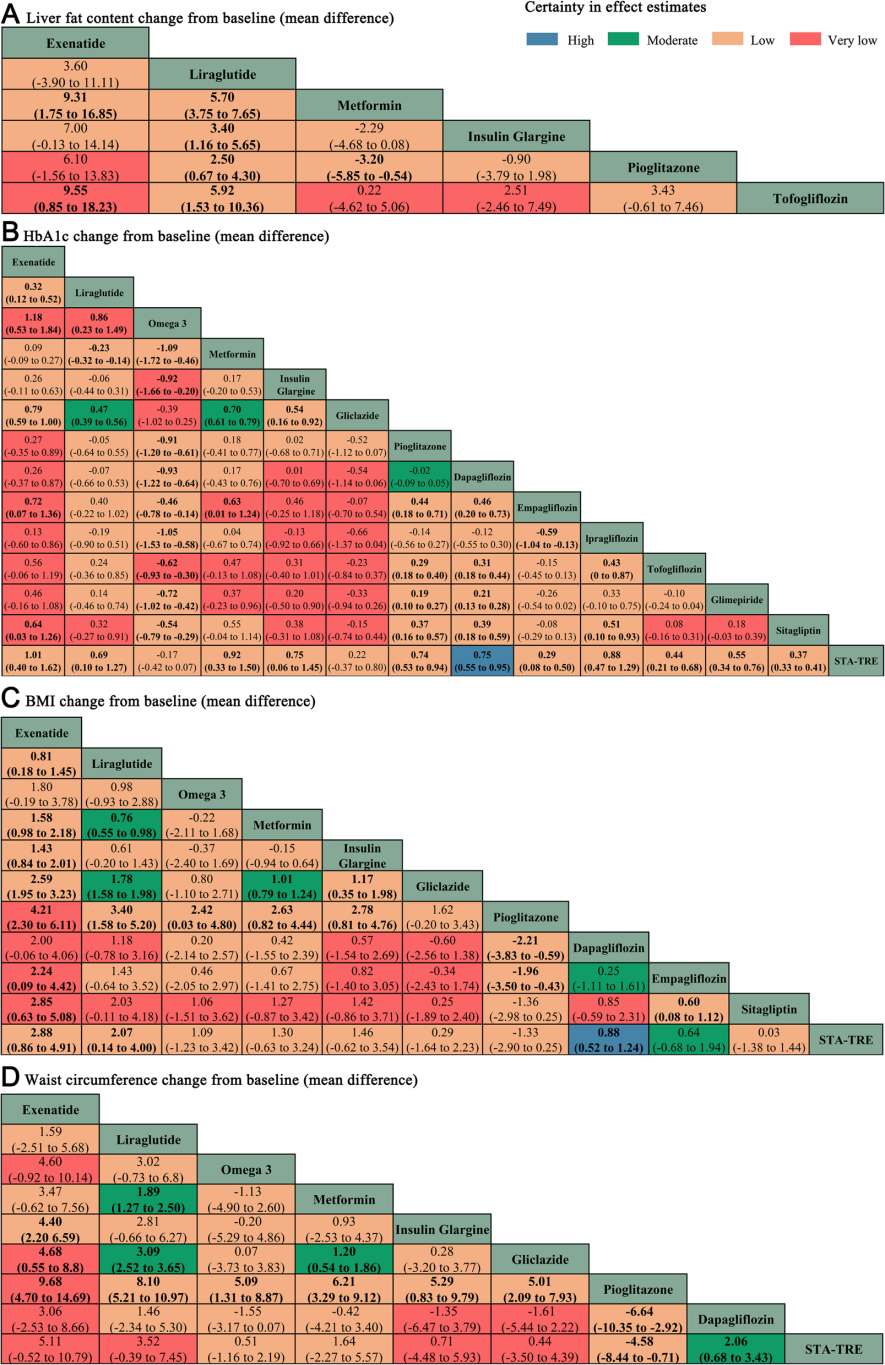
League matrix table of critical outcome analyses. **A** Liver fat content. **B** HbA_1c_. **C** BMI. **D** Waist circumference. Effect sizes where statistical differences exist are bolded, and the colour of each cell indicates the certainty in effect estimates

Liver fat content change from baseline was reported in 4 studies [56–58, 60], and all studies included in the analysis used magnetic resonance imaging proton density fat-fraction (MRI-PDFF) to measure liver fat content; some studies using CT and ultrasound as a means of measurement were not included. We used a coalition matrix (Fig. 3A) to describe the effects of the 6 interventions on participants’ liver fat content, and Bayesian net meta-analyses were performed for a total of 15 comparisons. Exenatide (MD 9.31, 95%CI 1.75 to 16.85) and liraglutide (MD 5.70, 95%CI 3.75 to 7.65) significantly reduce liver fat content compared to metformin. The SUCRA ranking plot (Additional file 1: Fig. S2A) displayed the ranking of the effectiveness of reducing liver fat content, with the most effective interventions being exenatide, followed by liraglutide and pioglitazone (SUCRA, 94.31%, 83.28%, and 54.75%, respectively).

### HbA_1c_

HbA_1c_ changes from baseline were reported in 21 studies [49, 51–60, 62, 63, 65–72]; we used a league matrix table (Fig. 3B) to describe the effect of 14 interventions on the effect of HbA_1c_ in participants with a total of 91 comparisons. Most interventions reduced HbA_1c_ compared to STA-TRE, with a statistically significant difference, but there was no statistically significant difference in the effect of Omega 3 (MD − 0.17, 95%CI − 0.42 to 0.07) compared to STA-TRE in reducing HbA_1c_, or even a trend towards lowering HbA_1c_. The SUCRA ranking plot (Additional file 1: Fig. S2B) displayed the ranking of the effectiveness of reducing HbA_1c_, with the most effective interventions being exenatide, followed by metformin and ipragliflozin (SUCRA, 91.06%, 82.54%, and 80.63%, respectively). Other results were amply demonstrated in the figure.

### BMI and waist circumference

Seventeen studies [49, 50, 52, 54–57, 60–66, 68, 69, 72] reported BMI change from baseline, and 10 studies [50, 53, 55–57, 60–62, 68, 71] reported waist circumference change from baseline. The league matrix (Fig. 3C, D) depicts the results of a total of 91 comparisons between different interventions. The remaining interventions, except exenatide, liraglutide, and dapagliflozin, did not show a weight reduction effect. SUCRA ranking plots (Additional file 1: Fig. S2C- Fig. S2D) displayed the ranking of the effects of BMI and waist circumference reduction, with the most effective interventions being both exenatide (SUCRA, 98.98%%, and *93.48*%, respectively), followed by liraglutide (SUCRA, *85.24*%%, and *85.59*%, respectively).

### Important but not crucial outcomes

Ten studies reported insulin resistance, 17 studies reported ALT, 2 studies reported VATA, and 2 studies reported SATA. The league matrix table (Additional file 1: Fig. S3A—Fig. S3D) describes the comparative results between the different interventions. SUCRA ranking plots (Additional file 1: Fig. S2E- Fig. S2H) displayed the ranking of the different interventions. Pioglitazone, GLP-1RAs, and dapagliflozin showed a significant improvement in the therapeutic effect of insulin resistance. The intervention with the best effect in improving insulin resistance was liraglutide (SUCRA, 91.03%). GLP-1RAs significantly reduced ALT levels in patients, but glimepiride demonstrated inferior results. The intervention that had the best effect in reducing ALT levels was exenatide (SUCRA, 99.77%). The intervention with the best effect in reducing VATA and SATA was also exenatide (SUCRA, 86.95% and 91.80%, respectively). The rest of the results were well presented in the league matrix table.

Some of the studies reported adverse events for the interventions, which we also summarised. Additional file 1: Table S8 clearly depicts the number of adverse events reported for the corresponding intervention. Hypoglycemia and gastrointestinal reactions were the most reported adverse drug events. Liraglutide had more pronounced appetite suppression reactions (22/29). One adverse event of heart failure was reported with pioglitazone, but it could not be determined to be caused by the drug.

No significant publication bias was detected in the results of all network meta-analyses, whether judged visually by funnel plots or quantitatively by Egger’s tests. These results were thoroughly demonstrated in Additional file 1: Fig. S4. Sufficient convergence was obtained for all models used in the calculations, and there was no heterogeneity in the results of the reticulated Meta-analysis for all outcomes. PSRF values for assessing convergence and *I*^2^ values for heterogeneity are shown in Additional file 1: Table S7.

### Certainty of evidence

**Fig. 4:**
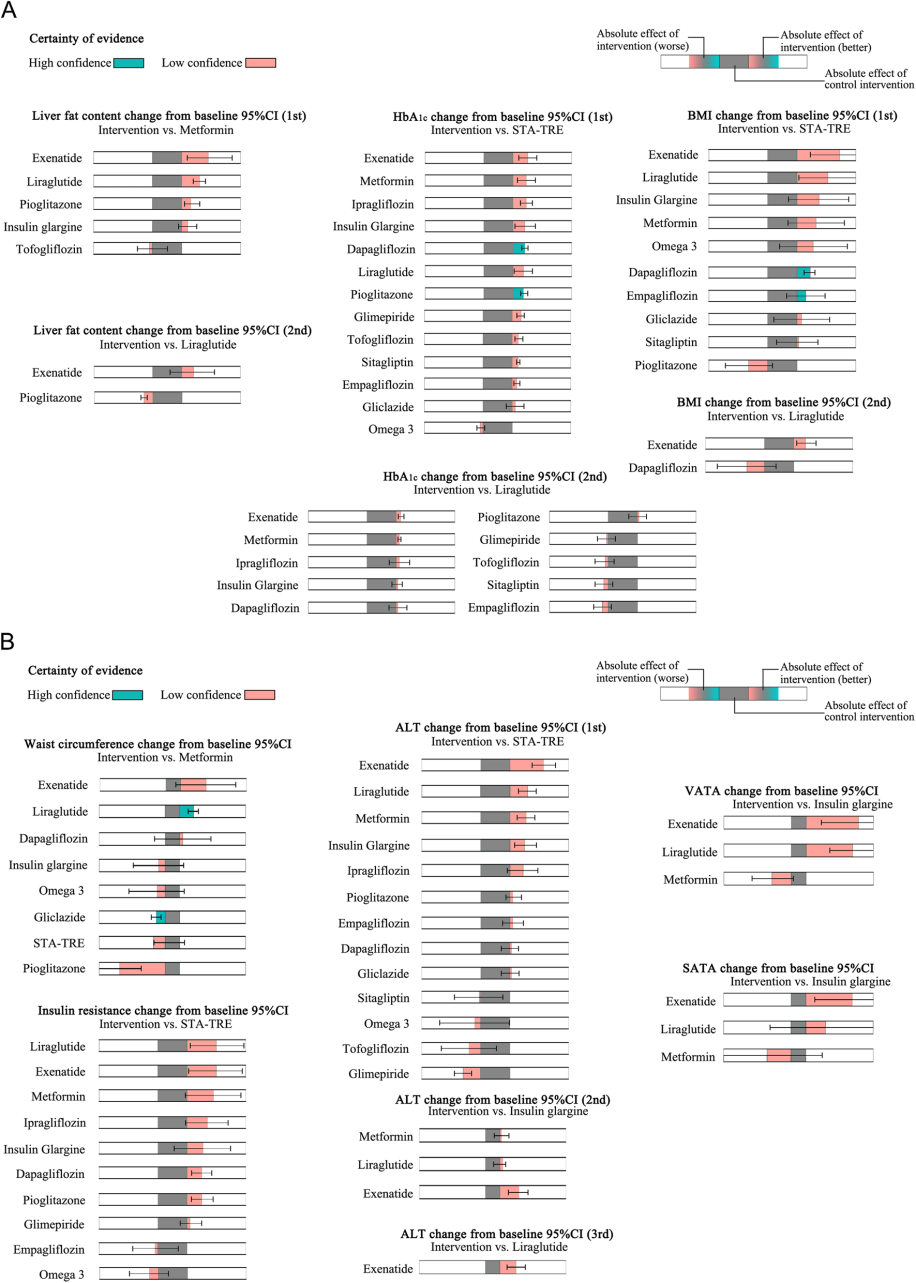
Summary of absolute effects of clinical indicators by minimally contextualised framework approach. **A** and **B** pooled effect represents the degree of improvement in each clinical indicator compared to the reference group. The conclusions mainly include most effective, effective, ineffective of high confidence and probably most effective, probably effective, and probably ineffective of low confidence. The upper bounds of some confidence intervals are truncated in the bar plots due to space

We summarise the certainty-in-effect estimates for all comparisons using the methodology recommended by the GRAD working group. Certainty-in-effect estimates for each comparison were displayed in all league matrix tables, and the reasons for downgrading were also shown. For transparency, this process is described in Additional file 1: Table S7. The generation of conclusions from the network meta-analysis should also be transparent, and the results were presented in Fig. 4A, B. In terms of lowering HbA_1c_ and lowering BMI, the certainty of evidence was assessed as low confidence, although exenatide demonstrated the best statistical effect. Therefore, dapagliflozin was raised. Liraglutide demonstrated a high statistical effect in reducing waist circumference and a high confidence level of certainty of evidence. At last, the results of the loop inconsistency test were displayed visually by means of forest plots (Additional file 1: Fig. S5). By evaluating the results of the direct comparisons, the results of the indirect comparisons, and the results of the network comparisons, no significant inconsistencies were observed among the results.

## Discussion

This network meta-analysis provides a comprehensive overview of the evidence on the treatment effects of several potential interventions for NAFLD with T2DM, capturing all recent publications. We evaluated the conclusion using the minimally contextualised framework approach recommended by the GRADE Working Group, which will help develop future guidelines.

The effects of blood glucose control and reduction of liver fat content are of primary concern and reasonable glycemic control reduces underlying cardiovascular risk in patients [73]. Based on the evidence provided, dapagliflozin and pioglitazone are effective interventions for reducing HbA_1c_ and sufficient evidence (high confidence) recommended dapagliflozin and pioglitazone for glycemic control in patients with NAFLD combined with T2DM. In addition, exenatide may be a superior intervention to dapagliflozin and pioglitazone, but the available evidence does not support this certainty (low confidence). A frequentist network meta-analysis combining drugs of the same type yielded statistically similar results to our study [74], but this study did not assess the certainty of the evidence, and we believe that GLP-1Ras received a too-high grade of recommendation in this study. Exenatide may be the most effective intervention as a GLP-1Ras class, but the available evidence is insufficient to recommend it as a first-line agent. However, exenatide may be tried in patients who do not have stable glycaemic control with dapagliflozin or pioglitazone. Reducing liver fat content is the key to avoiding the progression of NAFLD to nonalcoholic steatohepatitis. Exenatide, liraglutide, and pioglitazone may be effective interventions to reduce liver fat content (low confidence), with exenatide probably being the most effective intervention. Current research suggests that GLP-1Ras analogues reduce liver fat content primarily through a multidimensional combination of improved inflammation, insulin sensitisation, and glucose reduction [75, 76]. In our study, GLP-1Ras has a good ranking in all three aspects, which further proves these mechanisms.

Weight management is the cornerstone of NAFLD and T2DM treatment has been verified to be beneficial, but exercise and lifestyle interventions are challenging and unsustainable for some patients. Based on the evidence provided, dapagliflozin was an effective intervention for reducing BMI (moderate certainty evidence) in patients with NAFLD combined with T2DM. Exenatide and liraglutide are probably the most effective interventions for reducing BMI. Liraglutide is the most effective intervention to reduce waist circumference, and gliclazide is an ineffective intervention to reduce waist circumference. Currently, liraglutide has been approved for weight reduction in several countries; the included study [55] reported appetite suppression as an adverse event with an incidence rate of 22/29. This effect does not appear to be an adverse event for patients requiring weight loss. The results of this study confirm the effectiveness of liraglutide for weight reduction in patients with NAFLD combined with T2DM. In conclusion, sufficient evidence has shown that liraglutide can be recommended for weight reduction in patients with NAFLD combined with T2DM, especially for patients with abdominal obesity. On the other hand, exenatide might also be a good choice, as it is not significantly different from liraglutide in all weight-related metrics, except that the available evidence does not allow him to be given a higher level of evidence.

Laboratory indicators and clinical formula calculations have also received attention. Based on the available evidence, exenatide and liraglutide are probably the most effective interventions to improve liver function and insulin resistance and reduce VATA and SATA. However, glimepiride was significantly detrimental to liver function; it was the only drug among all interventions that elevated ALT levels, and the difference was statistically significant. Therefore, we do not recommend glimepiride in patients with NAFLD combined with T2DM who have liver function impairment. Omega-3 was the only intervention that did not have a trend towards lowering HbA_1c_ and did not perform well in other areas. A systematic review and meta-analysis of randomised controlled trials of high quality have concluded that Omega 3 has little or no effect on the prevention and treatment of T2DM [77]. Therefore, careful consideration should be given when selecting Omega 3, which should not be recommended for use. Unfortunately, these findings are of low evidence level, and it should be clarified that the reason for the low evidence level is not the quality of the included studies but because only a small number of investigators have focused on these indicators. These findings require future studies to enrich the evidence.

The assessment of inconsistency is very important, and we tested the comparisons of a closed loop for inconsistency using node-splitting models, and none of the results showed significant inconsistency. However, it is worth noting that the statistical value of the inconsistency test result for insulin glargine vs metformin in the HbA_1c_-lowering outcome was located at the edge of the boundaries (*P* = 0.051). A statistical value of 0.001 to sway a conclusion has the potential to cause bias. We therefore referred to the results of the forest plot and decided to downgrade the comparison in the certainty of effect estimates on the grounds of “Inconsistency”. Thanks to the well-established search strategy and quality assessment, all results did not show significant publication bias, which side-steps the credibility of the findings. In addition, as double-blinding was not possible due to differences in the mode of administration, we considered this part of the study to be at high risk of bias and decided to reduce the certainty in effect estimates on the basis of “Risk of bias”.

The above conclusions are generated based on the methodology recommended by the GRADE Working Group. In general, these conclusions are conservative and available for guideline development. According to the SUCRA, exenatide and liraglutide—which are GLP-1RAs—are ranked at the top (first or second) for the majority of interventions. GLP-1RAs can stimulate increased insulin secretion (glucose-dependent) and β-cell proliferation and inhibit gastric emptying; these mechanisms show remarkable promise for glycemic control and weight control. In addition, GLP-1RAs analogues can prevent abnormal endoplasmic reticulum stress by reducing intestinal absorption of dietary lipids, thus preventing fatty acid-related cell death in the liver, and thus, GLP-1RAs also show excellent potential for improving liver function [78, 79].

For the reason the pharmacokinetics of exenatide and liraglutide are different [80, 81], we did not combine exenatide and liraglutide into the GLP-1RAs classification for analysis, which led to some interesting results. Exenatide was superior to liraglutide in reducing HbA_1c_ in patients with NAFLD with T2DM, and exenatide ranked higher than liraglutide in most outcomes, especially in the outcome of HbA_1c_ where the superiority of exenatide over liraglutide was statistically significant; this contradicts the current consensus of T2DM. Based on several previous network meta-analyses of drugs for the treatment of T2DM, exenatide was less effective than liraglutide in lowering HbA_1c_ despite being the same as GLP-1RAs classification [82]. Regarding the evidence for the treatment of T2DM, the latest guidelines in China and Italy also recommend liraglutide as a representative of the GLP-1RAs class (level of evidence: B, II) [83, 84]. Of course, we must treat this result with caution, as this conclusion is assessed as low evidence.

By consulting the literature on liraglutide pharmacokinetics, we found a 13% reduction in liraglutide exposure in subjects with mild hepatic impairment (Child-Pugh score = 6) compared to healthy subjects. Subjects with moderate hepatic impairment (Child-Pugh score = 7–9) and subjects with severe hepatic impairment (Child-Pugh score > 9) had 23–44% lower liraglutide exposure [80, 85]. Child-Pugh scores of patients with NAFLD and T2DM easily achieve 6 or higher. In addition, liraglutide binds to albumin in plasma, and patients with NAFLD with T2DM exhibit chronic microproteinuria, further reducing liraglutide exposure [86]. In summary, we believe the patient’s hepatic impairment and prolonged microproteinuria may contribute to this outcome. However, it seems that no investigators have focused on this issue and that the therapeutic effect of liraglutide—a GLP-1RAs drug metabolised by the liver—may be reduced in patients with NAFLD. This certainty of the evidence is low and was derived by indirect prediction. To verify this conclusion, our centre designed a relevant RCT comparing the efficacy of exenatide and liraglutide in patients with NAFLD with T2DM, which will be published in the future and add more clinical evidence to this field; in addition, inspired by the results of related studies, we included once-weekly exenatide as an intervention added to it [87]. We are registering on the clinical trial registry website and will continue to report this finding.

Based on current studies, it is difficult to determine whether insulin resistance is the cause or consequence of NAFLD and T2DM. The interplay between T2DM, NAFLD, and insulin resistance could be considered a two-way street. However, what is certain is that insulin resistance and inflammation form a vicious spiral that promotes each other and accelerates the development of NAFLD and other metabolic disorders in the presence of lipotoxicity [88]. The improvement of insulin resistance indirectly responds to the treatment effect on NAFLD, and the results of its ranking showed a good agreement with other results, which proves that the above results are robust.

Only a small number of adverse events of drugs were reported in the literature, mainly including hypoglycemia, edema, urinary tract infection, and cutaneous symptom. Due to limitations of the number of literature, we could not perform a meta-analysis of rates of adverse reactions. It is noteworthy that no one withdrew from the 24 included studies due to adverse events of the drugs. We, therefore, consider these interventions to be relatively safe.

So far, we have done all we can to enrich our research within the limits of control, but we should still consider some limitations. Clinical treatment effects are a combination of drug and time, and the confounding factor of duration of drug action cannot be ruled out; the clinical effects of interventions may be magnified by longer dosing times. Although the investigators claim that the clinical indicators of the subjects reached stable levels at the end, the bias at the time of the study will potentially impact the amount of change in the corresponding clinical indicators. However, this did not significantly affect the final results, with omega 3, tofogliflozin, and glimepiride, dosed for up to 48 weeks, not ranking highly. In addition, the amount of available literature does not support our subgroup analysis, so the inherent differences in ethnicity cannot be addressed. Admittedly, this study has very limited reference value for the ethnicity of African and Hispanic. Not only that, the population included in the study was mostly overweight (BMI > 24) which is a very limited reference for normal weight patients. Moreover, the findings for ALT, insulin resistance, and the visceral fat area must be interpreted with caution due to limitations in the amount of literature. Too small a sample size may lead to results that are not stable due to limitations in the literature. The results of studies on ALT, insulin resistance, and the visceral fat area must be interpreted with caution; the ranking of the advantages and disadvantages of this section is likely to change with the addition of subsequent studies.

The available evidence is insufficient to elevate the GLP-1RAs to a higher priority, but they show excellent potential. We, therefore, suggest that future investigators should pay more attention to such drugs. It is important to note that there is a lack of cardiovascular outcome studies in patients with NAFLD with T2DM, and it is not known whether relevant studies in patients with T2DM can be directly referenced. We are, therefore, unable to advise on cardiovascular events. Glycemic management in patients with NAFLD combined with type 2 diabetes mellitus is a long-term process, and it is not possible to determine whether long-term treatment will result in a stable benefit for patients, whether the fat content of the liver will be reduced to normal and remain stable, whether the blood glucose level will be stabilised at a desirable level, whether normal-weight patients will also benefit from these medications, and whether the long-term use of GLP-1Ras will result in an even better cardiovascular benefit, which will have to be addressed in subsequent studies.

In the analysis of the current situation, the clinical treatment of NAFLD with T2DM is in its preliminary stage, and researchers have noted that NAFLD with T2DM cannot be treated simply by referring to T2DM, and relevant clinical studies are being conducted in different regions of the world. As a phase summary, this study provides a reference for guideline development and future research directions.

## Conclusions

The high confidence mandates the confident application of these findings as guides for clinical practice. Dapagliflozin and pioglitazone are used for glycaemic control in patients with NAFLD combined with T2DM, and liraglutide is used for weight loss therapy in patients with abdominal obesity. The available evidence does not demonstrate the credibility of the effectiveness of other interventions in reducing liver fat content, visceral fat area, ALT, and insulin resistance. Future studies should focus on the clinical application of GLP-1Ras and the long-term prognosis of patients.

### Supplementary Information

**Additional file 1: Table S1.** PRISMA 2020 Checklist. **Table S2.** Index and keyword terms used in the databases. **Table S3.** Lists of clinical trial registries and specialised journals. **Table S4.** Eligibility criteria. **Table S5.** Specific meaning of certainty in effect estimates. **Table S6.** List of excluded studies. **Table S7.** Certainty of evidence for direct, indirect and network estimates. **Table S8.** Adverse events of the interventions. **Fig. S1.** Quality assessment of the included studies and risk of bias summary for RCTs and risk of bias graph for RCTs. Individual risk of bias and overall risk of bias for each included study are visualised in green yellow and red representing low risk of bias unclear risk of bias and high risk of bias, respectively. **Fig. S2.** SUCRA ranking plot of intervention. (A) Liver fat content; (B) HbA_1c_; (C) BMI; (D) Waist circumference; (E) Insulin resistance; (F) ALT; (G) VATA; (H)SATA. The ranking plot represents the cumulative probability of being the best intervention, second and third. x-axis shows the relative rankings and y-axis shows the cumulative probability of each ranking. the larger the SUCRA, the higher it is ranked. **Fig. S3.** League matrix table of important but not crucial outcomes. (A) Insulin resistance; (B) ALT; (C) VATA; (D) SATA. Effect sizes where statistical differences exist are bolded, and the color of each cell indicates the certainty in effect estimates. **Fig. S4.** Funnel plots and Egger’s test. (A) HbA_1c_; (B) BMI; (C) Waist circumference; (D) Insulin resistance; (E) ALT. **Fig. S5.** Forest plot for inconsistency testing. (A) HbA_1c_; (B) Waist circumference; (C) BMI; (D) Insulin resistance; (E) ALT.

## Data Availability

The data used in this study are based on published studies, and the original data can be obtained by searching the relevant literature. In addition, the data supporting the results of this study and the code for the R software are available from the corresponding author, Haining Fan, upon reasonable request.

## Abbreviations

ALT: Alanine transaminase
BMI: Body mass index
CI: Confidence interval
GLP-1RAs: Glucagon-like peptide-1 receptor agonists
HbA_1C_: Glycated haemoglobin
MD: Mean difference
MRI-PDFF: Magnetic resonance imaging proton density fat-fraction
NAFLD: Nonalcoholic fatty liver disease
PSRF: Potential scale reduction factor
SATA: Subcutaneous adipose tissue area
SGLT-2Is: Sodium-dependent glucose transporters 2 inhibitors
STA-TRE: Standard-treatment
SUCRA: Surface under the cumulative ranking curves
T2DM: Type 2 diabetes mellitus
VATA: Visceral adipose tissue area
