# Supplementary material for: Comparative effectiveness of multiple different treatment regimens for nonalcoholic fatty liver disease with type 2 diabetes mellitus: a systematic review and Bayesian network meta-analysis of randomised controlled trials
Source: BMC Med. 2023 Nov 16;21:447. doi: 10.1186/s12916-023-03129-6 (PMC10655371; doi:10.1186/s12916-023-03129-6)
Supplement: Supplementary file 1 — Additional file 1: Table S1. PRISMA 2020 Checklist. Table S2. Index and keyword terms used in the databases. Table S3. Lists of clinical trial registries and specialised journals. Table S4. Eligibility criteria. Table S5. Specific meaning of certainty in effect estimates. Table S6. List of excluded studies. Table S7. Certainty of evidence for direct, indirect and network estimates. Table S8. Adverse events of the interventions. Fig. S1. Quality assessment of the included studies and risk of bias summary for RCTs and risk of bias graph for RCTs. Individual risk of bias and overall risk of bias for each included study are visualised in green yellow and red representing low risk of bias unclear risk of bias and high risk of bias, respectively. Fig. S2. SUCRA ranking plot of intervention. (A) Liver fat content; (B) HbA1c; (C) BMI; (D) Waist circumference; (E) Insulin resistance; (F) ALT; (G) VATA; (H)SATA. The ranking plot represents the cumulative probability of being the best intervention, second and third. x-axis shows the relative rankings and y-axis shows the cumulative probability of each ranking. the larger the SUCRA, the higher it is ranked. Fig. S3. League matrix table of important but not crucial outcomes. (A) Insulin resistance; (B) ALT; (C) VATA; (D) SATA. Effect sizes where statistical differences exist are bolded, and the color of each cell indicates the certainty in effect estimates. Fig. S4. Funnel plots and Egger’s test. (A) HbA1c; (B) BMI; (C) Waist circumference; (D) Insulin resistance; (E) ALT. Fig. S5. Forest plot for inconsistency testing. (A) HbA1c; (B) Waist circumference; (C) BMI; (D) Insulin resistance; (E) ALT. [file 12916_2023_3129_MOESM1_ESM.docx]

Supplementary Information for:

Comparative Effectiveness of Multiple Different Treatment Regimens for Nonalcoholic Fatty Liver Disease with Type 2 Diabetes Mellitus: A Systematic Review and Bayesian Network Meta-analysis of Randomized Controlled Trials

Manjun Deng ^1,2†^ Yonghao Wen ^1†^ JingXin Yan^1,3^ Yichen Fan^1^ Zhixin Wang^1,2^ Ruixia Zhang^4^ Li Ren^1,2^ Yinggui Ba ^5^ Haijiu Wang^1,2^ Qian Lu^6^ Haining Fan^1,2^

1 Department of Hepatopancreatobiliary Surgery, Affiliated Hospital of Qinghai University, Xining 810001, Qinghai, China

2 Qinghai Research Key Laboratory for Echinococcosis, Xining 810000, Xining 810001, Qinghai, China

3 Department of Interventional Therapy, Affiliated Hospital of Qinghai University, Xining 810001, Qinghai, China

4 Department of Endocrinology, Affiliated Hospital of Qinghai University, Xining 810001, Qinghai, China

5 Department of Nephrology, Affiliated Hospital of Qinghai University, Xining 810001, Qinghai, China

6 Department of Hepatopancreatobiliary Surgery, Tsinghua Changgung Hospital, Tsinghua University, Beijing 102218, China

†Manjun Deng and Yonghao Wen contributed equally to this work

Corresponding authors: Haining Fan and Qian Lu

E-mail: fanhaining@medmail.com.cn (Haining Fan); luqianbtch@163.com (Qian Lu)

Table of contents

[**Table S1** PRISMA 2020 checklist 2](#_Toc150817121)

[**Table S2** Index and keyword terms used in the databases. 6](#_Toc150817122)

[**Table S3** Lists of clinical trial registries and specialized journals. 8](#_Toc150817123)

[**Table S4** Eligibility criteria. 10](#_Toc150817124)

[**Table S5** Specific meaning of certainty in effect estimates 11](#_Toc150817125)

[**Table S6** List of excluded studies. 11](#_Toc150817126)

[**Table S7** Certainty of evidence for direct, indirect and network estimates 102](#_Toc150817127)

[**Table S8** Adverse events of the interventions 133](#_Toc150817128)

[**Continued Table S8** Adverse events of the interventions 134](#_Toc150817129)

[**Continued Table S8** Adverse events of the interventions 135](#_Toc150817130)

[**Figure S1** Quality assessment of the included studies and risk of bias summary for RCTs and risk of bias graph for RCTs. 138](#_Toc150817131)

[**Figure S2** SUCRA ranking plot of intervention 139](#_Toc150817132)

[**Figure S3** League matrix table of important but not crucial results 142](#_Toc150817133)

[**Figure S4** Funnel plots and Egger's test 143](#_Toc150817134)

[**Figure S5** Forest plot for inconsistency testing 146](#_Toc150817135)

# **Table S1** PRISMA 2020 checklist

| **Section and Topic** | **Item #** | **Checklist item** | **Location where item is reported** |
| --- | --- | --- | --- |
| **TITLE** | | |  |
| Title | 1 | Identify the report as a systematic review. | Page 1 |
| **ABSTRACT** | | |  |
| Abstract | 2 | See the PRISMA 2020 for Abstracts checklist. | Page 1-3 |
| **INTRODUCTION** | | |  |
| Rationale | 3 | Describe the rationale for the review in the context of existing knowledge. | Page 3-4 |
| Objectives | 4 | Provide an explicit statement of the objective(s) or question(s) the review addresses. | Page 3-4 |
| **METHODS** | | |  |
| Eligibility criteria | 5 | Specify the inclusion and exclusion criteria for the review and how studies were grouped for the syntheses. | Table S4 |
| Information sources | 6 | Specify all databases, registers, websites, organisations, reference lists and other sources searched or consulted to identify studies. Specify the date when each source was last searched or consulted. | Table S3 |
| Search strategy | 7 | Present the full search strategies for all databases, registers and websites, including any filters and limits used. | Table S2 |
| Selection process | 8 | Specify the methods used to decide whether a study met the inclusion criteria of the review, including how many reviewers screened each record and each report retrieved, whether they worked independently, and if applicable, details of automation tools used in the process. | Table S4 |
| Data collection process | 9 | Specify the methods used to collect data from reports, including how many reviewers collected data from each report, whether they worked independently, any processes for obtaining or confirming data from study investigators, and if applicable, details of automation tools used in the process. | Page 5-6 |
| Data items | 10a | List and define all outcomes for which data were sought. Specify whether all results that were compatible with each outcome domain in each study were sought (e.g. for all measures, time points, analyses), and if not, the methods used to decide which results to collect. | Page 6 |
|  | 10b | List and define all other variables for which data were sought (e.g. participant and intervention characteristics, funding sources). Describe any assumptions made about any missing or unclear information. | Page 5-6 |
| Study risk of bias assessment | 11 | Specify the methods used to assess risk of bias in the included studies, including details of the tool(s) used, how many reviewers assessed each study and whether they worked independently, and if applicable, details of automation tools used in the process. | Page 6 |
| Effect measures | 12 | Specify for each outcome the effect measure(s) (e.g. risk ratio, mean difference) used in the synthesis or presentation of results. | Page6-7 |
| Synthesis methods | 13a | Describe the processes used to decide which studies were eligible for each synthesis (e.g. tabulating the study intervention characteristics and comparing against the planned groups for each synthesis (item #5)). | Page 5-7 |
|  | 13b | Describe any methods required to prepare the data for presentation or synthesis, such as handling of missing summary statistics, or data conversions. | Page 5-7 |
|  | 13c | Describe any methods used to tabulate or visually display results of individual studies and syntheses. | Page 6-7 |
|  | 13d | Describe any methods used to synthesize results and provide a rationale for the choice(s). If meta-analysis was performed, describe the model(s), method(s) to identify the presence and extent of statistical heterogeneity, and software package(s) used. | Page6-7 |
|  | 13e | Describe any methods used to explore possible causes of heterogeneity among study results (e.g. subgroup analysis, meta-regression). | Page6-7 |
|  | 13f | Describe any sensitivity analyses conducted to assess robustness of the synthesized results. | Page 6-7 |
| Reporting bias assessment | 14 | Describe any methods used to assess risk of bias due to missing results in a synthesis (arising from reporting biases). | Page 9-10 & Figure S1 |
| Certainty assessment | 15 | Describe any methods used to assess certainty (or confidence) in the body of evidence for an outcome. | Page 7-8 |
| **RESULTS** | | |  |
| Study selection | 16a | Describe the results of the search and selection process, from the number of records identified in the search to the number of studies included in the review, ideally using a flow diagram. | Page 8-9 & Figure 1 |
|  | 16b | Cite studies that might appear to meet the inclusion criteria, but which were excluded, and explain why they were excluded. | Table S6 |
| Study characteristics | 17 | Cite each included study and present its characteristics. | Table 1 |
| Risk of bias in studies | 18 | Present assessments of risk of bias for each included study. | Figure S1 |
| Results of individual studies | 19 | For all outcomes, present, for each study: (a) summary statistics for each group (where appropriate) and (b) an effect estimate and its precision (e.g. confidence/credible interval), ideally using structured tables or plots. | Table S7 |
| Results of syntheses | 20a | For each synthesis, briefly summarise the characteristics and risk of bias among contributing studies. | Table S7 |
|  | 20b | Present results of all statistical syntheses conducted. If meta-analysis was done, present for each the summary estimate and its precision (e.g. confidence/credible interval) and measures of statistical heterogeneity. If comparing groups, describe the direction of the effect. | Table S7 |
|  | 20c | Present results of all investigations of possible causes of heterogeneity among study results. | Table S7 |
|  | 20d | Present results of all sensitivity analyses conducted to assess the robustness of the synthesized results. | Table S7 |
| Reporting biases | 21 | Present assessments of risk of bias due to missing results (arising from reporting biases) for each synthesis assessed. | Not applicable |
| Certainty of evidence | 22 | Present assessments of certainty (or confidence) in the body of evidence for each outcome assessed. | Table S7 & Figure 3 |
| **DISCUSSION** | | |  |
| Discussion | 23a | Provide a general interpretation of the results in the context of other evidence. | Page 13-20 |
|  | 23b | Discuss any limitations of the evidence included in the review. | Page 13-20 |
|  | 23c | Discuss any limitations of the review processes used. | Page 13-20 |
|  | 23d | Discuss implications of the results for practice, policy, and future research. | Page 13-20 |
| **OTHER INFORMATION** | | |  |
| Registration and protocol | 24a | Provide registration information for the review, including register name and registration number, or state that the review was not registered. | Page 5 |
|  | 24b | Indicate where the review protocol can be accessed, or state that a protocol was not prepared. | Page 5 |
|  | 24c | Describe and explain any amendments to information provided at registration or in the protocol. | Page 5 |
| Support | 25 | Describe sources of financial or non-financial support for the review, and the role of the funders or sponsors in the review. | Page 21 |
| Competing interests | 26 | Declare any competing interests of review authors. | Page 21 |
| Availability of data, code and other materials | 27 | Report which of the following are publicly available and where they can be found: template data collection forms; data extracted from included studies; data used for all analyses; analytic code; any other materials used in the review. | Page 4-7 |

# **Table S2** Index and keyword terms used in the databases.

| **Database** | **Index and keyword terms** |
| --- | --- |
| **PubMed** | (((((((((Non-alcoholic Fatty Liver Disease[MeSH Terms]) OR (Non alcoholic Fatty Liver Disease[Title/Abstract])) OR (NAFLD[Title/Abstract])) OR (Nonalcoholic Fatty Liver Disease[Title/Abstract])) OR (Fatty Liver, Nonalcoholic[Title/Abstract])) OR (Liver, Nonalcoholic Fatty[Title/Abstract])) OR (Fatty Livers, Nonalcoholic[Title/Abstract])) OR (Livers, Nonalcoholic Fatty[Title/Abstract])) OR (Nonalcoholic Fatty Liver[Title/Abstract])) OR (Nonalcoholic Fatty Livers[Title/Abstract])  AND  Randomized Controlled Trial |
| **Cochrane** | ID Search  #1 MeSH descriptor: [Non-alcoholic Fatty Liver Disease] explode all trees  #2 Non alcoholic Fatty Liver Disease  #3 Nonalcoholic Fatty Liver Disease  #4 NAFLD  #5 Fatty Liver, Nonalcoholic  #6 Fatty Livers, Nonalcoholic  #7 Liver, Nonalcoholic Fatty  #8 Livers, Nonalcoholic Fatty  #9 Nonalcoholic Fatty Liver  #10 #1 OR #2 OR #3 OR #4 OR #5 OR #6 OR #7 OR #8 OR #9  #11 Randomized Controlled Trial  #12 #10 AND #11 |
| **EMBASE** | #1. 'non alcoholic' AND fatty AND ('liver'/exp OR liver) AND ('disease'/exp OR disease)  #2. non AND alcoholic AND fatty AND liver AND disease  #3. nonalcoholic AND fatty AND liver AND disease  #4. fatty AND liver, AND nonalcoholic  #5. fatty AND livers, AND nonalcoholic  #6. liver, AND nonalcoholic AND fatty  #7. livers, AND nonalcoholic AND fatty  #8. nonalcoholic AND fatty AND liver  #9. nonalcoholic AND fatty AND livers  #10. #1 OR #2 OR #3 OR #4 OR #5 OR #6 OR #7 OR #8 OR #9  #11. randomized AND controlled AND trial  #12. #10 AND #11 |
| **Web of Science** | TS=(Non-alcoholic Fatty Liver Disease OR Non alcoholic Fatty Liver Disease OR Nonalcoholic Fatty Liver Disease OR NAFLD OR Fatty Liver*, Nonalcoholic OR Liver*, Nonalcoholic Fatty OR Nonalcoholic Fatty Liver*)  AND  TS=(randomized controlled trial) |

# **Table S3** Lists of clinical trial registries and specialized journals.

| Sources | Website |
| --- | --- |
| ***Clinical trial registries:*** |  |
| Australian New Zealand Clinical Trials Registry | http://www.anzctr.org.au/ |
| CenterWatch | http://www.centerwatch.com/ |
| ClinicalTrials.gov | https://clinicaltrials.gov/ |
| Cochrane Central Register of Controlled Trials | https://www.cochranelibrary.com/central/about-central |
| EU Clinical Trials Register | https://www.clinicaltrialsregister.eu/ctr-search/search |
|  |  |
| Singapore Clinical Trials Register | https://www.hsa.gov.sg/clinical-trials/clinical-trials-register |
| UK Clinical Research Network: Portfolio Database | https://www.nihr.ac.uk/research-and-impact/nihr-clinical-research-network-portfolio/ |
| World Health Organization International Clinical Trials Registry Platform  European Medicines Agency  International Standard Randomised Controlled Trial Number Registry | https://www.who.int/ictrp  https://www.ema.europa.eu/ema/  https://www.isrctn.com/ |
| ***Specialized journals:*** |  |
| Annals of Surgery | https://journals.lww.com/annalsofsurgery/pages/default.aspx |
| BMC Medical Informatics and Decision Making | https://bmcmedinformdecismak.biomedcentral.com/ |
| British Journal of Surgery | https://bjssjournals.onlinelibrary.wiley.com/journal/13652168 |
| International Journal of Surgery | https://www.journals.elsevier.com/international-journal-of-surgery |
| JAMA Surgery | https://jamanetwork.com/journals/jamasurgery |
| Journal of the American College of Surgeons | https://www.journalacs.org/ |
| Journal of Vascular Surgery | https://www.jvascsurg.org/ |
| Medical Decision Making | https://journals.sagepub.com/home/mdm |
| Plastic and Reconstructive Surgery | https://journals.lww.com/plasreconsurg/pages/default.aspx |
| Surgery | https://www.journals.elsevier.com/surgery |
| The American Journal of Surgery | https://www.journals.elsevier.com/the-american-journal-of-surgery |
| World Journal of Surgery | https://www.springer.com/journal/268 |
|  |  |
| ***Grey literature resources:*** |  |
| CogPrints | http://cogprints.org/ |
| Google Scholar | http://scholar.google.com.sg/ |
| GreySource | http://www.greynet.org/greysourceindex.html |
| NYAM Grey Literature Report | http://www.greylit.org/ |
| OpenGrey: System for Information on Grey Literature in Europe | http://www.opengrey.eu/ |
| OpenMD.com | https://openmd.com/directory/allergy-immunology |
| Science.gov | http://www.science.gov/scigov/ |
| World Bank Publications | https://www.worldbank.org/en/research |

# **Table S4** Eligibility criteria.

| Criteria | Inclusion | Exclusion |
| --- | --- | --- |
| Population | - Adult patients were diagnosed with non- alcoholic fatty liver disease with type 2 diabetes. | - The study population was children. - Non-NAFLD subjects; Nondiabetic subjects; Prediabetes subjects |
| Intervention | - Established or potentially beneficial therapies for NAFLD according to American Association for the Study of Liver Diseases (AASLD) guidelines. | - Study on the treatment of traditional Chinese medicine. - Non-drug treatment research. - Only one article on the same kind of intervention. |
| Comparison | - Another active substance or hypoglycemic standard treatment. |  |
| Outcomes | The main outcome including:   - Change in glycated hemoglobin (HbA1C); - Change in liver fat content; - Change in body mass index (BMI); - Waist circumference;   The secondary outcome including:   - ALT; - Insulin resistance; - Visceral adipose tissue area (VATA); - Subcutaneous adipose tissue area (SATA); | - Unable to extract usable data. |
| Type of design | - Any types of randomized controlled trials | - Non-experimental, one-arm, qualitative or review studies. |
| Years of publication | - From inception to June 30, 2023 |  |
| Publication type | - Published and unpublished trials | - Abstract only - Book chapter review - Letters - Editorials - Systematic review |
| Language | - English | - Non-English |

# **Table S5** Specific meaning of certainty in effect estimates

| certainty in effect estimates | specific meanings |  |
| --- | --- | --- |
| High certainty | We are very confident that the true effect lies close to that of |  |
|  | the estimate of the effect. |  |
| Moderate certainty  Low certainty  Very low certainty | We are moderately confident in the effect estimate; the true effect is likely to be close to the estimate of the effect, but there is a possibility that it is substantially different.  Our confidence in the effect estimate is limited; the true effect may be substantially different from the estimate of the effect.  We have very little confidence in the effect estimate; the true effect is likely to be substantially different from the estimate of effect. |  |
|  |  |  |

# **Table S6** List of excluded studies.

| **Year** | **Title** | **DOI** | **Reason for exclusion** |
| --- | --- | --- | --- |
| 2021 | Energy Expenditure Improved Risk Factors Associated with Renal Function Loss in NAFLD and MetS Patients. | https://doi.org/10.3390/nu13020629 | Not relevant to our study |
| 2016 | Biochemical parameters response to weight loss in patients with non-alcoholic steatohepatitis. | https://doi.org/10.4314/ahs.v16i1.32 | Not relevant to our study |
| 2021 | The leukotriene receptor antagonist montelukast in the treatment of non-alcoholic steatohepatitis: A proof-of-concept, randomized, double-blind, placebo-controlled trial. | https://doi.org/10.1016/j.ejphar.2021.174295 | The study population was not diagnosed with diabetes |
| 2020 | Effects of high-intensity interval and moderate-intensity continuous aerobic exercise on diabetic obese patients with nonalcoholic fatty liver disease: A comparative randomized controlled trial. | https://doi.org/10.1097/md.0000000000019471 | Non-drug treatment research |
| 2019 | A randomized controlled trial on the effectiveness of 8-week high-intensity interval exercise on intrahepatic triglycerides, visceral lipids, and health-related quality of life in diabetic obese patients with nonalcoholic fatty liver disease. | https://doi.org/10.1097/md.0000000000014918 | Non-drug treatment research |
| 2021 | The FALCON program: Two phase 2b randomized, double-blind, placebo-controlled studies to assess the efficacy and safety of pegbelfermin in the treatment of patients with nonalcoholic steatohepatitis and bridging fibrosis or compensated cirrhosis. | https://doi.org/10.1016/j.cct.2021.106335 | The study population was not diagnosed with diabetes |
| 2009 | Betaine for nonalcoholic fatty liver disease: results of a randomized placebo-controlled trial. | https://doi.org/10.1002/hep.23239 | The study population was not diagnosed with diabetes |
| 2023 | A phase 2, adaptive randomized, double-blind, placebo-controlled, multicenter, 52-week study of HM15211 in patients with biopsy-confirmed non-alcoholic steatohepatitis - Study design and rationale of HM-TRIA-201 study. | https://doi.org/10.1016/j.cct.2023.107176 | The study population was not diagnosed with diabetes |
| 2018 | Rifaximin in nonalcoholic fatty liver disease: hit multiple targets with a single shot. | https://doi.org/10.1097/meg.0000000000001232 | The study population was not diagnosed with diabetes |
| 2010 | Metformin: a therapeutic option for treating nonalcoholic fatty liver disease. | https://doi.org/10.1007/s10620-010-1279-1 | The study population was not diagnosed with diabetes |
| 2017 | Effect of Mediterranean Diet and Antioxidant Formulation in Non-Alcoholic Fatty Liver Disease: A Randomized Study. | https://doi.org/10.3390/nu9080870 | Non-drug treatment research |
| 2015 | Effects of Mediterranean diet supplemented with silybin-vitamin E-phospholipid complex in overweight patients with non-alcoholic fatty liver disease. | https://doi.org/10.1586/17474124.2015.1004312 | Non-drug treatment research |
| 2020 | The effects of Bacillus coagulans supplementation in patients with non-alcoholic fatty liver disease: A randomized, placebo-controlled, clinical trial. | https://doi.org/10.1016/j.clnesp.2020.06.020 | Non-drug treatment research |
| 2010 | The effects of Xanthigen in the weight management of obese premenopausal women with non-alcoholic fatty liver disease and normal liver fat. | https://doi.org/10.1111/j.1463-1326.2009.01132.x | Not relevant to our study |
| 2015 | The impact of phlebotomy in nonalcoholic fatty liver disease: A prospective, randomized, controlled trial. | https://doi.org/10.1002/hep.27662 | Not relevant to our study |
| 2002 | Management of nonalcoholic steatohepatitis: an analytic review. | https://doi.org/10.1097/00004836-200209000-00011 | Review article |
| 2019 | Randomized, Double-blind, Placebo-controlled Study of a Multispecies Probiotic Mixture in Nonalcoholic Fatty Liver Disease. | https://doi.org/10.1038/s41598-019-42059-3 | Non-drug treatment research |
| 2008 | Randomized, placebo-controlled trial of pioglitazone in nondiabetic subjects with nonalcoholic steatohepatitis. | https://doi.org/10.1053/j.gastro.2008.06.047 | The study population was not diagnosed with diabetes |
| 2019 | MRI Assessment of Treatment Response in HIV-associated NAFLD: A Randomized Trial of a Stearoyl-Coenzyme-A-Desaturase-1 Inhibitor (ARRIVE Trial). | https://doi.org/10.1002/hep.30674 | Not relevant to our study |
| 2022 | Effect of rosemary leaf powder with weight loss diet on lipid profile, glycemic status, and liver enzymes in patients with nonalcoholic fatty liver disease: A randomized, double-blind clinical trial. | https://doi.org/10.1002/ptr.7446 | Non-drug treatment research |
| 2022 | The effect of a Mediterranean diet vs. a low-fat diet on non-alcoholic fatty liver disease in children: a randomized trial. | https://doi.org/10.1080/09637486.2021.1979478 | Non-drug treatment research |
| 2016 | Effect of telmisartan on histological activity and fibrosis of non-alcoholic steatohepatitis: A 1-year randomized control trial. | https://doi.org/10.4103/1319-3767.173762 | The study population was not diagnosed with diabetes |
| 2022 | The effect of a fruit-rich diet on liver biomarkers, insulin resistance, and lipid profile in patients with non-alcoholic fatty liver disease: a randomized clinical trial. | https://doi.org/10.1080/00365521.2022.2071109 | Non-drug treatment research |
| 2022 | Effect of resveratrol supplementation on hepatic steatosis and cardiovascular indices in overweight subjects with type 2 diabetes: a double-blind, randomized controlled trial. | https://doi.org/10.1186/s12872-022-02637-2 | The study population was not diagnosed with NAFLD |
| 2014 | Randomised clinical trial: The beneficial effects of VSL#3 in obese children with non-alcoholic steatohepatitis. | https://doi.org/10.1111/apt.12758 | The study population was not diagnosed with diabetes |
| 2013 | Weight reduction improves markers of hepatic function and insulin resistance in type-2 diabetic patients with non-alcoholic fatty liver. | https://doi.org/10.4314/ahs.v13i3.21 | Non-drug treatment research |
| 2022 | Safety and efficacy of combination therapy with semaglutide, cilofexor and firsocostat in patients with non-alcoholic steatohepatitis: A randomised, open-label phase II trial. | https://doi.org/10.1016/j.jhep.2022.04.003 | The study population was not diagnosed with diabetes |
| 2011 | Effect of a probiotic on liver aminotransferases in nonalcoholic fatty liver disease patients: a double blind randomized clinical trial. | https://doi.org/10.1016/j.jhep.2022.04.003 | Non-drug treatment research |
| 2014 | Effect of a high monounsaturated vs high polyunsaturated fat hypocaloric diets in nonalcoholic fatty liver disease. |  | Non-drug treatment research |
| 2015 | Effect of silymarin plus vitamin E in patients with non-alcoholic fatty liver disease. A randomized clinical pilot study. |  | The study population was not diagnosed with diabetes |
| 2018 | Genistein supplementation improves insulin resistance and inflammatory state in non-alcoholic fatty liver patients: A randomized, controlled trial. | https://doi.org/10.1016/j.clnu.2017.05.028 | The study population was not diagnosed with diabetes |
| 2021 | Effect of Mastiha supplementation on NAFLD: The MAST4HEALTH Randomised, Controlled Trial. | https://doi.org/10.1002/mnfr.202001178 | The study population was not diagnosed with diabetes |
| 2021 | Mastiha has efficacy in immune-mediated inflammatory diseases through a microRNA-155 Th17 dependent action. | https://doi.org/10.1016/j.phrs.2021.105753 | Basic research |
| 2019 | Targeting diacylglycerol acyltransferase 2 for the treatment of nonalcoholic steatohepatitis. | https://doi.org/10.1126/scitranslmed.aav9701 | The study population was not diagnosed with diabetes |
| 2022 | Efficacy and safety of an orally administered DGAT2 inhibitor alone or coadministered with a liver-targeted ACC inhibitor in adults with non-alcoholic steatohepatitis (NASH): rationale and design of the phase II, dose-ranging, dose-finding, randomised, placebo-controlled MIRNA (Metabolic Interventions to Resolve NASH with fibrosis) study. | https://doi.org/10.1136/bmjopen-2021-056159 | The study population was not diagnosed with diabetes |
| 2020 | Cenicriviroc for the treatment of liver fibrosis in adults with nonalcoholic steatohepatitis: AURORA Phase 3 study design. | https://doi.org/10.1016/j.cct.2019.105922 | The study population was not diagnosed with diabetes |
| 2019 | Treatment options for nonalcoholic fatty liver disease: a double-blinded randomized placebo-controlled trial. | https://doi.org/10.1097/meg.0000000000001369 | The study population was not diagnosed with diabetes |
| 2022 | The effect of hydroxy citric acid supplementation with calorie-restricted diet on metabolic, atherogenic and inflammatory biomarkers in women with non-alcoholic fatty liver disease: a randomized controlled clinical trial. | https://doi.org/10.1039/d1fo03685h | Non-drug treatment research |
| 2011 | Effect of atorvastatin, vitamin E and C on nonalcoholic fatty liver disease: is the combination required? | https://doi.org/10.1038/ajg.2010.310 | The study population was not diagnosed with diabetes |
| 2015 | Effects of n-3 fish oil on metabolic and histological parameters in NASH: a double-blind, randomized, placebo-controlled trial. | https://doi.org/10.1016/j.jhep.2014.08.036 | The study population was not diagnosed with diabetes |
| 2018 | Physical deconditioning is the common denominator in both obese and overweight subjects with nonalcoholic steatohepatitis. | https://doi.org/10.1111/apt.14803 | Non-drug treatment research |
| 2016 | Liraglutide safety and efficacy in patients with non-alcoholic steatohepatitis (LEAN): a multicentre, double-blind, randomised, placebo-controlled phase 2 study. | https://doi.org/10.1016/s0140-6736(15)00803-x | The study population was not diagnosed with diabetes |
| 2016 | Glucagon-like peptide 1 decreases lipotoxicity in non-alcoholic steatohepatitis. | https://doi.org/10.1016/j.jhep.2015.08.038 | The study population was not diagnosed with diabetes |
| 2022 | Effect of intensive weight-loss intervention on metabolic, ultrasound and anthropometric parameters among patients with obesity and non-alcoholic fatty liver disease: an RCT. | https://doi.org/10.1038/s41430-022-01111-8 | Non-drug treatment research |
| 2007 | Influence of liver biopsy heterogeneity and diagnosis of nonalcoholic steatohepatitis in subjects undergoing gastric bypass. | https://doi.org/10.1007/s11695-007-9041-2 | Non-drug treatment research |
| 2018 | Comparison of Calorie-Restricted Diet and Resveratrol Supplementation on Anthropometric Indices, Metabolic Parameters, and Serum Sirtuin-1 Levels in Patients With Nonalcoholic Fatty Liver Disease: A Randomized Controlled Clinical Trial. | https://doi.org/10.1080/07315724.2017.1392264 | Non-drug treatment research |
| 2022 | Effects of Calorie Restricted Diet on Oxidative/Antioxidative Status Biomarkers and Serum Fibroblast Growth Factor 21 Levels in Nonalcoholic Fatty Liver Disease Patients: A Randomized, Controlled Clinical Trial. | https://doi.org/10.3390/nu14122509 | Non-drug treatment research |
| 2014 | Cinnamon may have therapeutic benefits on lipid profile, liver enzymes, insulin resistance, and high-sensitivity C-reactive protein in nonalcoholic fatty liver disease patients. | https://doi.org/10.1016/j.nutres.2013.11.005 | The study population was not diagnosed with diabetes |
| 2022 | Sesame Oil Ameliorates Alanine Aminotransferase, Aspartate Aminotransferase, and Fatty Liver Grade in Women with Nonalcoholic Fatty Liver Disease Undergoing Low-Calorie Diet: A Randomized Double-Blind Controlled Trial. | https://doi.org/10.1155/2022/4982080 | The study population was not diagnosed with diabetes |
| 2006 | Effect of multifactorial treatment on non-alcoholic fatty liver disease in metabolic syndrome: a randomised study. | https://doi.org/10.1185/030079906x104696 | The study population was not diagnosed with diabetes |
| 2010 | Safety and efficacy of long-term statin treatment for cardiovascular events in patients with coronary heart disease and abnormal liver tests in the Greek Atorvastatin and Coronary Heart Disease Evaluation (GREACE) Study: a post-hoc analysis. | https://doi.org/10.1016/s0140-6736(10)61272-x | Not relevant to our study |
| 2013 | The impact of smoking on cardiovascular outcomes and comorbidities in statin-treated patients with coronary artery disease: a post hoc analysis of the GREACE study. | https://doi.org/10.2174/1570161111311050016 | Not relevant to our study |
| 2011 | Metformin and/or clomiphene do not adversely affect liver or renal function in women with polycystic ovary syndrome. | https://doi.org/10.1210/jc.2011-1093 | Not relevant to our study |
| 2018 | Text messaging approach improves weight loss in patients with nonalcoholic fatty liver disease: A randomized study. | https://doi.org/10.1111/liv.13622 | Non-drug treatment research |
| 2020 | Comparison of the efficacy of oral fenugreek seeds hydroalcoholic extract versus placebo in nonalcoholic fatty liver disease; a randomized, triple-blind controlled pilot clinical trial. | https://doi.org/10.4103/ijp.IJP_17_19 | The study population was not diagnosed with diabetes |
| 2022 | Effects of exercise on NAFLD using non-targeted metabolomics in adipose tissue, plasma, urine, and stool. | https://doi.org/10.1038/s41598-022-10481-9 | Non-drug treatment research |
| 2013 | Both resistance training and aerobic training reduce hepatic fat content in type 2 diabetic subjects with nonalcoholic fatty liver disease (the RAED2 Randomized Trial). | https://doi.org/10.1002/hep.26393 | Non-drug treatment research |
| 2015 | Improvement of Nonalcoholic Fatty Liver Disease With Carnitine-Orotate Complex in Type 2 Diabetes (CORONA): A Randomized Controlled Trial. | https://doi.org/10.2337/dc14-2852 | Only one article on the same kind of intervention |
| 2020 | The effect of melatonin on treatment of patients with non-alcoholic fatty liver disease: a randomized double blind clinical trial. | https://doi.org/10.1016/j.ctim.2020.102452 | The study population was not diagnosed with diabetes |
| 2018 | Daily Consumption of Synbiotic Yogurt Decreases Liver Steatosis in Patients with Nonalcoholic Fatty Liver Disease: A Randomized Controlled Clinical Trial. | https://doi.org/10.1093/jn/nxy088 | Non-drug treatment research |
| 2007 | Pioglitazone treatment increases whole body fat but not total body water in patients with non-alcoholic steatohepatitis. | https://doi.org/10.1016/j.jhep.2007.04.013 | The study population was not diagnosed with diabetes |
| 2017 | Effects of short-term energy restriction on liver lipid content and inflammatory status in severely obese adults: Results of a randomized controlled trial using 2 dietary approaches. | https://doi.org/10.1111/dom.12918 | Non-drug treatment research |
| 2015 | Volume-dependent effect of supervised exercise training on fatty liver and visceral adiposity index in subjects with type 2 diabetes The Italian Diabetes Exercise Study (IDES). | https://doi.org/10.1016/j.diabres.2015.05.033 | Non-drug treatment research |
| 2009 | Effects of ursodeoxycholic acid in combination with vitamin E on adipokines and apoptosis in patients with nonalcoholic steatohepatitis. | https://doi.org/10.1111/j.1478-3231.2009.02037.x | The study population was not diagnosed with diabetes |
| 2019 | Haptoglobin 2 Allele is Associated With Histologic Response to Vitamin E in Subjects With Nonalcoholic Steatohepatitis. | https://doi.org/10.1097/mcg.0000000000001142 | The study population was not diagnosed with diabetes |
| 2019 | Effects of different exercise modalities on novel hepatic steatosis indices in overweight women with type 2 diabetes. | https://doi.org/10.3350/cmh.2018.0086 | Non-drug treatment research |
| 2020 | A reference map of potential determinants for the human serum metabolome. | https://doi.org/10.1038/s41586-020-2896-2 | Basic research |
| 2019 | COMPARATIVE ANALYSIS OF EFFICIENCY OF URSODEOXYCHOLIC ACID AND COMBINATION OF VITAMIN E AND VITAMIN C IN TREATMENT OF NON-DIABETIC NONALCOHOLIC STEATOHEPATITIS. |  | The study population was not diagnosed with diabetes |
| 2023 | Oral 24-week probiotics supplementation did not decrease cardiovascular risk markers in patients with biopsy proven NASH: A double-blind placebo-controlled randomized study. | https://doi.org/10.1016/j.aohep.2022.100769 | The study population was not diagnosed with diabetes |
| 2018 | Procollagen-III peptide identifies adipose tissue-associated inflammation in type 2 diabetes with or without nonalcoholic liver disease. | https://doi.org/10.1002/dmrr.2998 | Basic research |
| 2016 | No effects of oral vitamin D supplementation on non-alcoholic fatty liver disease in patients with type 2 diabetes: a randomized, double-blind, placebo-controlled trial. | https://doi.org/10.1186/s12916-016-0638-y | Only one article on the same kind of intervention |
| 2013 | Effect of exercise training and isoflavones on hepatic steatosis in overweight postmenopausal women. | https://doi.org/10.3109/13697137.2012.662251 | Not relevant to our study |
| 2016 | Nonalcoholic fatty liver may increase the risk of operation in patients with fatty liver and the frequency of cancer in their first-degree relatives. | https://doi.org/10.1007/s00508-015-0744-4 | Not relevant to our study |
| 2014 | Fatty liver disease might increase the risk of abdominal operation in patients with fatty liver and the prevalence of cancer in first-degree relatives. | https://doi.org/10.5152/tjg.2014.7674 | Not relevant to our study |
| 2020 | Efficacy of Hijamat (wet cupping therapy) in Iranian patients with nonalcoholic fatty liver disease: a controlled clinical trial. | https://doi.org/10.3906/sag-1907-82 | The study population was not diagnosed with diabetes |
| 2022 | Association of chronic liver disease with cognition and brain volumes in two randomized controlled trial populations. | https://doi.org/10.1016/j.jns.2021.120117 | Not relevant to our study |
| 2017 | Increased liver fat and glycogen stores after consumption of high versus low glycaemic index food: A randomized crossover study. | https://doi.org/10.1111/dom.12784 | Not relevant to our study |
| 2020 | Effects of probiotic and prebiotic supplementation on metabolic parameters, liver aminotransferases, and systemic inflammation in nonalcoholic fatty liver disease: A randomized clinical trial. | https://doi.org/10.1111/1750-3841.15367 | The study population was not diagnosed with diabetes |
| 2006 | A placebo-controlled trial of pioglitazone in subjects with nonalcoholic steatohepatitis. | https://doi.org/10.1056/NEJMoa060326 | The study population was not diagnosed with diabetes |
| 2012 | Relationship between adipose tissue insulin resistance and liver histology in nonalcoholic steatohepatitis: a pioglitazone versus vitamin E versus placebo for the treatment of nondiabetic patients with nonalcoholic steatohepatitis trial follow-up study. | https://doi.org/10.1002/hep.25805 | The study population was not diagnosed with diabetes |
| 2009 | Metabolic syndrome and alanine aminotransferase: a global perspective from the NAVIGATOR screening population. | https://doi.org/10.1111/j.1464-5491.2009.02864.x | Not relevant to our study |
| 2021 | Inhibition of fatty acid synthase with FT-4101 safely reduces hepatic de novo lipogenesis and steatosis in obese subjects with non-alcoholic fatty liver disease: Results from two early-phase randomized trials. | https://doi.org/10.1111/dom.14272 | Not relevant to our study |
| 2017 | Carvedilol delays the progression of small oesophageal varices in patients with cirrhosis: a randomised placebo-controlled trial. | https://doi.org/10.1136/gutjnl-2016-311735 | Not relevant to our study |
| 2016 | Improvement in non-alcoholic fatty liver disease severity is associated with a reduction in carotid intima-media thickness progression. | https://doi.org/10.1016/j.atherosclerosis.2015.12.028 | Not relevant to our study |
| 2020 | Placebo-controlled randomised trial with liraglutide on magnetic resonance endpoints in individuals with type 2 diabetes: a pre-specified secondary study on ectopic fat accumulation. | https://doi.org/10.1007/s00125-019-05021-6 | The study population was not diagnosed with NAFLD |
| 2015 | Isoleucine-to-methionine substitution at residue 148 variant of PNPLA3 gene and metabolic outcomes in gestational diabetes. | https://doi.org/10.3945/ajcn.114.095125 | Not relevant to our study |
| 2019 | Histological improvement of non-alcoholic steatohepatitis with a prebiotic: a pilot clinical trial. | https://doi.org/10.1007/s00394-018-1721-2 | The study population was not diagnosed with diabetes |
| 2017 | Reduction of visceral fat by liraglutide is associated with ameliorations of hepatic steatosis, albuminuria, and micro-inflammation in type 2 diabetic patients with insulin treatment: a randomized control trial. | https://doi.org/10.1507/endocrj.EJ16-0449 | The study population was not diagnosed with NAFLD |
| 2018 | Screening for therapeutic trials and treatment indication in clinical practice: MACK-3, a new blood test for the diagnosis of fibrotic NASH. | https://doi.org/10.1111/apt.14621 | Not relevant to our study |
| 2015 | Long-Term Treatment with n-3 Polyunsaturated Fatty Acids as a Monotherapy in Children with Nonalcoholic Fatty Liver Disease. | https://doi.org/10.4274/jcrpe.1749 | The study population was not diagnosed with diabetes |
| 2016 | Insulin resistance, postprandial GLP-1 and adaptive immunity are the main predictors of NAFLD in a homogeneous population at high cardiovascular risk. | https://doi.org/10.1016/j.numecd.2016.01.011 | Not relevant to our study |
| 2020 | Protocol for a phase IV, open-label feasibility study investigating non-invasive markers of hepatic fibrosis in people living with HIV-1 and non-alcoholic fatty liver disease randomised to receiving optimised background therapy (OBT) plus maraviroc or OBT alone. | https://doi.org/10.1136/bmjopen-2019-035596 | Not relevant to our study |
| 2020 | Protocol for a randomised trial testing a community fibrosis assessment service for patients with suspected non-alcoholic fatty liver disease: LOCal assessment and triage evaluation of non-alcoholic fatty liver disease (LOCATE-NAFLD). | https://doi.org/10.1186/s12913-020-05233-2 | Not relevant to our study |
| 2020 | Change in hepatic fat content measured by MRI does not predict treatment-induced histological improvement of steatohepatitis. | https://doi.org/10.1016/j.jhep.2019.09.018 | Not relevant to our study |
| 2019 | Role of Vitamin E for Nonalcoholic Steatohepatitis in Patients With Type 2 Diabetes: A Randomized Controlled Trial. | https://doi.org/10.2337/dc19-0167 | Only one article on the same kind of intervention |
| 2017 | Liver Safety of Statins in Prediabetes or T2DM and Nonalcoholic Steatohepatitis: Post Hoc Analysis of a Randomized Trial. | https://doi.org/10.1210/jc.2017-00867 | Prediabetes |
| 2005 | Incidence and risk factors for non-alcoholic steatohepatitis: prospective study of 5408 women enrolled in Italian tamoxifen chemoprevention trial. | https://doi.org/10.1136/bmj.38391.663287.E0 | Not relevant to our study |
| 2005 | A randomized controlled trial of metformin versus vitamin E or prescriptive diet in nonalcoholic fatty liver disease. | https://doi.org/10.1111/j.1572-0241.2005.41583.x | The study population was not diagnosed with diabetes |
| 2010 | [Use of Legalon in non-alcoholic fatty liver disease]. |  | The study population was not diagnosed with diabetes |
| 2019 | Effects of alternate-day fasting on body weight and dyslipidaemia in patients with non-alcoholic fatty liver disease: a randomised controlled trial. | https://doi.org/10.1186/s12876-019-1132-8 | Non-drug treatment research |
| 2021 | Low-carbohydrate diets lead to greater weight loss and better glucose homeostasis than exercise: a randomized clinical trial. | https://doi.org/10.1007/s11684-021-0861-6 | Non-drug treatment research |
| 2022 | A Low Glycemic Index Mediterranean Diet Combined with Aerobic Physical Activity Rearranges the Gut Microbiota Signature in NAFLD Patients. | https://doi.org/10.3390/nu14091773 | Basic research |
| 2009 | Comparison of blood tests for liver fibrosis specific or not to NAFLD. | https://doi.org/10.1016/j.jhep.2008.07.035 | Not relevant to our study |
| 2021 | ACC inhibitor alone or co-administered with a DGAT2 inhibitor in patients with non-alcoholic fatty liver disease: two parallel, placebo-controlled, randomized phase 2a trials. | https://doi.org/10.1038/s41591-021-01489-1 | The study population was not diagnosed with diabetes |
| 2021 | Omega-3 carboxylic acids and fenofibrate differentially alter plasma lipid mediators in patients with non-alcoholic fatty liver disease. | https://doi.org/10.1096/fj.202100380RRR | The study population was not diagnosed with diabetes |
| 2020 | The Effect of Three Mediterranean Diets on Remnant Cholesterol and Non-Alcoholic Fatty Liver Disease: A Secondary Analysis. | https://doi.org/10.3390/nu12061674 | Non-drug treatment research |
| 2012 | The role of pro/anti-inflammatory adipokines on bone metabolism in NAFLD obese adolescents: effects of long-term interdisciplinary therapy. | https://doi.org/10.1007/s12020-012-9613-3 | Not relevant to our study |
| 2020 | Impact of Long-Term Supplementation with Fish Oil in Individuals with Non-Alcoholic Fatty Liver Disease: A Double Blind Randomized Placebo Controlled Clinical Trial. | https://doi.org/10.3390/nu12113372 | The study population was not diagnosed with diabetes |
| 2018 | Dietary Inflammatory Index and liver status in subjects with different adiposity levels within the PREDIMED trial. | https://doi.org/10.1016/j.clnu.2017.06.027 | Not relevant to our study |
| 2017 | Fruit Fiber Consumption Specifically Improves Liver Health Status in Obese Subjects under Energy Restriction. | https://doi.org/10.3390/nu9070667 | Not relevant to our study |
| 2019 | Ultrasound/Elastography techniques, lipidomic and blood markers compared to Magnetic Resonance Imaging in non-alcoholic fatty liver disease adults. | https://doi.org/10.7150/ijms.28044 | Not relevant to our study |
| 2013 | Dual peroxisome proliferator-activated receptor α/δ agonist GFT505 improves hepatic and peripheral insulin sensitivity in abdominally obese subjects. | https://doi.org/10.2337/dc12-2012 | Not relevant to our study |
| 2016 | Macrophage Activation in Pediatric Nonalcoholic Fatty Liver Disease (NAFLD) Correlates with Hepatic Progenitor Cell Response via Wnt3a Pathway. | https://doi.org/10.1371/journal.pone.0157246 | Basic research |
| 2023 | Atezolizumab plus bevacizumab versus lenvatinib for unresectable hepatocellular carcinoma: a large real-life worldwide population. | https://doi.org/10.1016/j.ejca.2022.11.017 | Not relevant to our study |
| 2019 | Altilix(®) Supplement Containing Chlorogenic Acid and Luteolin Improved Hepatic and Cardiometabolic Parameters in Subjects with Metabolic Syndrome: A 6 Month Randomized, Double-Blind, Placebo-Controlled Study. | https://doi.org/10.3390/nu11112580 | Not relevant to our study |
| 2014 | Effects of treatment with melatonin and tryptophan on liver enzymes, parameters of fat metabolism and plasma levels of cytokines in patients with non-alcoholic fatty liver disease--14 months follow up. |  | The study population was not diagnosed with diabetes |
| 2020 | Randomised trial of chronic supplementation with a nutraceutical mixture in subjects with non-alcoholic fatty liver disease. | https://doi.org/10.1017/s0007114519002484 | Non-drug treatment research |
| 2020 | Impact of aerobic training with and without whole-body vibration training on metabolic features and quality of life in non-alcoholic fatty liver disease patients. | https://doi.org/10.1016/j.ando.2020.05.003 | Non-drug treatment research |
| 2014 | Resveratrol does not benefit patients with nonalcoholic fatty liver disease. | https://doi.org/10.1016/j.cgh.2014.02.024 | The study population was not diagnosed with diabetes |
| 2020 | Effects of Belapectin, an Inhibitor of Galectin-3, in Patients With Nonalcoholic Steatohepatitis With Cirrhosis and Portal Hypertension. | https://doi.org/10.1053/j.gastro.2019.11.296 | The study population was not diagnosed with diabetes |
| 2019 | Relationship between three commonly used non-invasive fibrosis biomarkers and improvement in fibrosis stage in patients with non-alcoholic steatohepatitis. | https://doi.org/10.1111/liv.13974 | Not relevant to our study |
| 2018 | Randomised clinical trial: a leucine-metformin-sildenafil combination (NS-0200) vs placebo in patients with non-alcoholic fatty liver disease. | https://doi.org/10.1111/apt.14674 | The study population was not diagnosed with diabetes |
| 2009 | Pioglitazone versus vitamin E versus placebo for the treatment of non-diabetic patients with non-alcoholic steatohepatitis: PIVENS trial design. | https://doi.org/10.1016/j.cct.2008.09.003 | The study population was not diagnosed with diabetes |
| 2019 | The effects of dietary supplementation with inulin and inulin-propionate ester on hepatic steatosis in adults with non-alcoholic fatty liver disease. | https://doi.org/10.1111/dom.13500 | The study population was not diagnosed with diabetes |
| 2010 | Effect of ezetimibe on hepatic fat, inflammatory markers, and apolipoprotein B-100 kinetics in insulin-resistant obese subjects on a weight loss diet. | https://doi.org/10.2337/dc09-1765 | Not relevant to our study |
| 2018 | Lifestyle intervention in obese Chinese adolescents with non-alcoholic fatty liver disease: a randomised controlled study. |  | Non-drug treatment research |
| 2018 | Dietitian-led lifestyle modification programme for obese Chinese adolescents with non-alcoholic fatty liver disease: a randomized controlled study. | https://doi.org/10.1038/s41366-018-0010-8 | Non-drug treatment research |
| 2006 | Yo Jyo Hen Shi Ko (YHK) improves transaminases in nonalcoholic steatohepatitis (NASH): a randomized pilot study. | https://doi.org/10.1007/s10620-006-8030-y | The study population was not diagnosed with diabetes |
| 2013 | Oat prevents obesity and abdominal fat distribution, and improves liver function in humans. | https://doi.org/10.1007/s11130-013-0336-2 | Not relevant to our study |
| 2014 | Hibiscus sabdariffa extract inhibits obesity and fat accumulation, and improves liver steatosis in humans. | https://doi.org/10.1039/c3fo60495k | Not relevant to our study |
| 2016 | Lipid profiling of the therapeutic effects of berberine in patients with nonalcoholic fatty liver disease. | https://doi.org/10.1186/s12967-016-0982-x | The study population was not diagnosed with diabetes |
| 2021 | Moderate-Intensity Aerobic vs Resistance Exercise and Dietary Modification in Patients With Nonalcoholic Fatty Liver Disease: A Randomized Clinical Trial. | https://doi.org/10.14309/ctg.0000000000000316 | Non-drug treatment research |
| 2019 | A randomized 3-way crossover study indicates that high-protein feeding induces de novo lipogenesis in healthy humans. | https://doi.org/10.1172/jci.insight.124819 | Not relevant to our study |
| 2019 | Pegbelfermin (BMS-986036), PEGylated FGF21, in Patients with Obesity and Type 2 Diabetes: Results from a Randomized Phase 2 Study. | https://doi.org/10.1002/oby.22344 | The study population was not diagnosed with NAFLD |
| 2019 | A pilot study of the effect of phospholipid curcumin on serum metabolomic profile in patients with non-alcoholic fatty liver disease: a randomized, double-blind, placebo-controlled trial. | https://doi.org/10.1038/s41430-018-0386-5 | The study population was not diagnosed with diabetes |
| 2022 | Incremental levels of diagnostic information incentivize health-seeking in non-alcoholic fatty liver: a randomized clinical trial. | https://doi.org/10.1038/s41598-022-12295-1 | Not relevant to our study |
| 2022 | The Efficacy of Oligonol in Nonalcoholic Fatty Liver Disease: A Randomized Double-Blinded Placebo-Controlled Trial. | https://doi.org/10.1089/jicm.2021.0362 | The study population was not diagnosed with diabetes |
| 2020 | Impact of a low-carbohydrate and high-fiber diet on nonalcoholic fatty liver disease. | https://doi.org/10.6133/apjcn.202009_29(3).0006 | Non-drug treatment research |
| 2006 | [Effects of resolving phlegm method on fibrinolytic status in non-alcoholic steatohepatitis patients of phlegm and blood-stasis syndrome]. |  | Non-drug treatment research |
| 2015 | Resveratrol improves insulin resistance, glucose and lipid metabolism in patients with non-alcoholic fatty liver disease: a randomized controlled trial. | https://doi.org/10.1016/j.dld.2014.11.015 | The study population was not diagnosed with diabetes |
| 2015 | Dihydromyricetin improves glucose and lipid metabolism and exerts anti-inflammatory effects in nonalcoholic fatty liver disease: A randomized controlled trial. | https://doi.org/10.1016/j.phrs.2015.05.009 | The study population was not diagnosed with diabetes |
| 2019 | Yogurt improves insulin resistance and liver fat in obese women with nonalcoholic fatty liver disease and metabolic syndrome: a randomized controlled trial. | https://doi.org/10.1093/ajcn/nqy358 | The study population was not diagnosed with diabetes |
| 2006 | [Clinical study of treatment nonalcoholic fatty liver with qianggan capsule]. |  | The study population was not diagnosed with diabetes |
| 2022 | A randomized controlled trial for response of microbiome network to exercise and diet intervention in patients with nonalcoholic fatty liver disease. | https://doi.org/10.1038/s41467-022-29968-0 | Non-drug treatment research |
| 2017 | Effect of aerobic exercise and diet on liver fat in pre-diabetic patients with non-alcoholic-fatty-liver-disease: A randomized controlled trial. | https://doi.org/10.1038/s41598-017-16159-x | Non-drug treatment research |
| 2019 | Hesperidin improves hepatic steatosis, hepatic enzymes, and metabolic and inflammatory parameters in patients with nonalcoholic fatty liver disease: A randomized, placebo-controlled, double-blind clinical trial. | https://doi.org/10.1002/ptr.6406 | The study population was not diagnosed with diabetes |
| 2021 | Hepatoprotective Effect of Antrodia cinnamomea Mycelium in Patients with Nonalcoholic Steatohepatitis: A Randomized, Double-Blind, Placebo-Controlled Trial. | https://doi.org/10.1080/07315724.2020.1779850 | The study population was not diagnosed with diabetes |
| 2022 | The Synergic Effect of a Nutraceutical Supplementation Associated to a Mediterranean Hypocaloric Diet in a Population of Overweight/Obese Adults with NAFLD. | https://doi.org/10.3390/nu14224750 | Non-drug treatment research |
| 2022 | Ezetimibe combination therapy with statin for non-alcoholic fatty liver disease: an open-label randomized controlled trial (ESSENTIAL study). | https://doi.org/10.1186/s12916-022-02288-2 | The study population was not diagnosed with diabetes |
| 2014 | Efficacy and safety of human placental extract for alcoholic and nonalcoholic steatohepatitis: an open-label, randomized, comparative study. | https://doi.org/10.1248/bpb.b13-00979 | The study population was not diagnosed with diabetes |
| 2020 | Randomised Double-Blind Placebo-Controlled Trial of Inulin with Metronidazole in Non-Alcoholic Fatty Liver Disease (NAFLD). | https://doi.org/10.3390/nu12040937 | The study population was not diagnosed with diabetes |
| 2021 | A randomised placebo controlled trial of VSL#3(®) probiotic on biomarkers of cardiovascular risk and liver injury in non-alcoholic fatty liver disease. | https://doi.org/10.1186/s12876-021-01660-5 | The study population was not diagnosed with diabetes |
| 2006 | The add-on effects of Gynostemma pentaphyllum on nonalcoholic fatty liver disease. |  | The study population was not diagnosed with diabetes |
| 2020 | Effects of phytosomal curcumin on anthropometric parameters, insulin resistance, cortisolemia and non-alcoholic fatty liver disease indices: a double-blind, placebo-controlled clinical trial. | https://doi.org/10.1007/s00394-019-01916-7 | The study population was not diagnosed with diabetes |
| 2020 | Effects of Epeleuton, a Novel Synthetic Second-Generation n-3 Fatty Acid, on Non-Alcoholic Fatty Liver Disease, Triglycerides, Glycemic Control, and Cardiometabolic and Inflammatory Markers. | https://doi.org/10.1161/jaha.119.016334 | The study population was not diagnosed with diabetes |
| 2016 | Higher body fat percentage is associated with enhanced temperature perception in NAFLD: results from the randomised Wessex Evaluation of fatty Liver and Cardiovascular markers in NAFLD with OMacor thErapy trial (WELCOME) trial. | https://doi.org/10.1007/s00125-016-3966-8 | Not relevant to our study |
| 2023 | Indices of hepatic steatosis and fibrosis in prediabetes and association with diabetes development in the vitamin D and type 2 diabetes study. | https://doi.org/10.1016/j.jdiacomp.2023.108475 | Not relevant to our study |
| 2015 | Improvement in liver histology is associated with reduction in dyslipidemia in children with nonalcoholic fatty liver disease. | https://doi.org/10.1097/mpg.0000000000000584 | Not relevant to our study |
| 2015 | NASH resolution is associated with improvements in HDL and triglyceride levels but not improvement in LDL or non-HDL-C levels. | https://doi.org/10.1111/apt.13035 | Not relevant to our study |
| 2019 | Relationship between resolution of non-alcoholic steatohepatitis and changes in lipoprotein sub-fractions: a post-hoc analysis of the PIVENS trial. | https://doi.org/10.1111/apt.15216 | Not relevant to our study |
| 2019 | Berberis aristata, Elaeis guineensis and Coffea canephora Extracts Modulate the Insulin Receptor Expression and Improve Hepatic Steatosis in NAFLD Patients: A Pilot Clinical Trial. | https://doi.org/10.3390/nu11123070 | The study population was not diagnosed with diabetes |
| 2022 | Reduction of De Novo Lipogenesis Mediates Beneficial Effects of Isoenergetic Diets on Fatty Liver: Mechanistic Insights from the MEDEA Randomized Clinical Trial. | https://doi.org/10.3390/nu14102178 | Non-drug treatment research |
| 2021 | Comparison of Ketogenic Diets with and without Ketone Salts versus a Low-Fat Diet: Liver Fat Responses in Overweight Adults. | https://doi.org/10.3390/nu13030966 | Non-drug treatment research |
| 2020 | Allogenic Fecal Microbiota Transplantation in Patients With Nonalcoholic Fatty Liver Disease Improves Abnormal Small Intestinal Permeability: A Randomized Control Trial. | https://doi.org/10.14309/ajg.0000000000000661 | The study population was not diagnosed with diabetes |
| 2022 | A Specifically Tailored Multistrain Probiotic and Micronutrient Mixture Affects Nonalcoholic Fatty Liver Disease-Related Markers in Patients with Obesity after Mini Gastric Bypass Surgery. | https://doi.org/10.1093/jn/nxab392 | The study population was not diagnosed with diabetes |
| 2016 | Sitagliptin vs. placebo for non-alcoholic fatty liver disease: A randomized controlled trial. | https://doi.org/10.1016/j.jhep.2016.04.021 | Prediabetes |
| 2020 | Efficacy of a 2-Month Very Low-Calorie Ketogenic Diet (VLCKD) Compared to a Standard Low-Calorie Diet in Reducing Visceral and Liver Fat Accumulation in Patients With Obesity. | https://doi.org/10.3389/fendo.2020.00607 | Non-drug treatment research |
| 2021 | Efficacy and safety of PXL770, a direct AMP kinase activator, for the treatment of non-alcoholic fatty liver disease (STAMP-NAFLD): a randomised, double-blind, placebo-controlled, phase 2a study. | https://doi.org/10.1016/s2468-1253(21)00300-9 | The study population was not diagnosed with diabetes |
| 2019 | Effect of canagliflozin treatment on hepatic triglyceride content and glucose metabolism in patients with type 2 diabetes. | https://doi.org/10.1111/dom.13584 | The study population was not diagnosed with NAFLD |
| 2016 | Long-Term Pioglitazone Treatment for Patients With Nonalcoholic Steatohepatitis and Prediabetes or Type 2 Diabetes Mellitus: A Randomized Trial. | https://doi.org/10.7326/m15-1774 | Prediabetes |
| 2009 | Omega-3 fatty acid supplementation decreases liver fat content in polycystic ovary syndrome: a randomized controlled trial employing proton magnetic resonance spectroscopy. | https://doi.org/10.1210/jc.2009-0870 | Not relevant to our study |
| 2016 | Dissociation between exercise-induced reduction in liver fat and changes in hepatic and peripheral glucose homoeostasis in obese patients with non-alcoholic fatty liver disease. | https://doi.org/10.1042/cs20150447 | Not relevant to our study |
| 2018 | Vitamin D supplementation for the treatment of non-alcoholic fatty liver disease: A randomized double blind placebo controlled trial. | https://doi.org/10.1016/j.dsx.2018.03.006 | The study population was not diagnosed with diabetes |
| 2022 | Lingguizhugan Decoction, a Chinese herbal formula, improves insulin resistance in overweight/obese subjects with non-alcoholic fatty liver disease: a translational approach. | https://doi.org/10.1007/s11684-021-0880-3 | Study on the treatment of traditional Chinese medicine |
| 2015 | Essential phospholipids as a supportive adjunct in the management of patients with NAFLD. | https://doi.org/10.1016/j.ajg.2015.09.001 | The study population was not diagnosed with diabetes |
| 2017 | The effects of green cardamom on blood glucose indices, lipids, inflammatory factors, paraxonase-1, sirtuin-1, and irisin in patients with nonalcoholic fatty liver disease and obesity: study protocol for a randomized controlled trial. | https://doi.org/10.1186/s13063-017-1979-3 | The study population was not diagnosed with diabetes |
| 2019 | Green cardamom supplementation improves serum irisin, glucose indices, and lipid profiles in overweight or obese non-alcoholic fatty liver disease patients: a double-blind randomized placebo-controlled clinical trial. | https://doi.org/10.1186/s12906-019-2465-0 | The study population was not diagnosed with diabetes |
| 2019 | Inflammatory markers response to citrulline supplementation in patients with non-alcoholic fatty liver disease: a randomized, double blind, placebo-controlled, clinical trial. | https://doi.org/10.1186/s13104-019-4130-6 | Not relevant to our study |
| 2019 | The effects of black seed supplementation on cardiovascular risk factors in patients with nonalcoholic fatty liver disease: A randomized, double-blind, placebo-controlled clinical trial. | https://doi.org/10.1002/ptr.6424 | The study population was not diagnosed with diabetes |
| 2019 | Nigella sativa and inflammatory biomarkers in patients with non-alcoholic fatty liver disease: Results from a randomized, double-blind, placebo-controlled, clinical trial. | https://doi.org/10.1016/j.ctim.2019.04.014 | The study population was not diagnosed with diabetes |
| 2021 | Effect of Portulaca Oleracea (purslane) extract on liver enzymes, lipid profile, and glycemic status in nonalcoholic fatty liver disease: A randomized, double-blind clinical trial. | https://doi.org/10.1002/ptr.6972 | The study population was not diagnosed with diabetes |
| 2022 | Triglyceride Lowering with Pemafibrate to Reduce Cardiovascular Risk. | https://doi.org/10.1056/NEJMoa2210645 | Not relevant to our study |
| 2005 | Effects of oligofructose on glucose and lipid metabolism in patients with nonalcoholic steatohepatitis: results of a pilot study. | https://doi.org/10.1038/sj.ejcn.1602127 | The study population was not diagnosed with diabetes |
| 2020 | Suboptimal reliability of liver biopsy evaluation has implications for randomized clinical trials. | https://doi.org/10.1016/j.jhep.2020.06.025 | Not relevant to our study |
| 2017 | Endocannabinoid receptor blockade reduces alanine aminotransferase in polycystic ovary syndrome independent of weight loss. | https://doi.org/10.1186/s12902-017-0194-2 | Not relevant to our study |
| 2020 | Bariatric surgery during the evolution of fatty liver-A randomized clinical trial comparing gastric bypass and sleeve gastrectomy based on transient elastography. | https://doi.org/10.1111/cob.12393 | Not relevant to our study |
| 2017 | Aerobic training performed at ventilatory threshold improves liver enzymes and lipid profile related to non-alcoholic fatty liver disease in adolescents with obesity. | https://doi.org/10.1177/0260106017720350 | Non-drug treatment research |
| 2010 | Effect of two different hypocaloric diets in transaminases and insulin resistance in nonalcoholic fatty liver disease and obese patients. |  | Non-drug treatment research |
| 2012 | Long-term effects of aerobic plus resistance training on the adipokines and neuropeptides in nonalcoholic fatty liver disease obese adolescents. | https://doi.org/10.1097/MEG.0b013e32835793ac | Non-drug treatment research |
| 2019 | Comprehensive lifestyle intervention vs soy protein-based meal regimen in non-alcoholic steatohepatitis. | https://doi.org/10.3748/wjg.v25.i9.1116 | Non-drug treatment research |
| 2016 | Docosahexanoic Acid Plus Vitamin D Treatment Improves Features of NAFLD in Children with Serum Vitamin D Deficiency: Results from a Single Centre Trial. | https://doi.org/10.1371/journal.pone.0168216 | The study population was children |
| 2020 | Effects of a multifactorial ecosustainable isocaloric diet on liver fat in patients with type 2 diabetes: randomized clinical trial. | https://doi.org/10.1136/bmjdrc-2020-001342 | Non-drug treatment research |
| 2005 | The state of insulin resistance in patients with nonalcoholic fatty liver and the intervention with Gankangyin. | https://doi.org/10.1007/bf02836467 | The study population was not diagnosed with diabetes |
| 2015 | Perindopril and barnidipine alone or combined with simvastatin on hepatic steatosis and inflammatory parameters in hypertensive patients. | https://doi.org/10.1016/j.ejphar.2015.09.030 | Not relevant to our study |
| 2021 | The Glucagon-Like Peptide-1 Receptor Agonist, Semaglutide, for the Treatment of Nonalcoholic Steatohepatitis. | https://doi.org/10.1002/hep.31886 | The study population was not diagnosed with diabetes |
| 2022 | Changes in the serum metabolomic profiles of subjects with NAFLD in response to n-3 PUFAs and phytosterol ester: a double-blind randomized controlled trial. | https://doi.org/10.1039/d1fo03921k | The study population was not diagnosed with diabetes |
| 2018 | A Khorasan Wheat-Based Replacement Diet Improves Risk Profile of Patients With Nonalcoholic Fatty Liver Disease (NAFLD): A Randomized Clinical Trial. | https://doi.org/10.1080/07315724.2018.1445047 | The study population was not diagnosed with diabetes |
| 2020 | Decreased Consumption of Added Fructose Reduces Waist Circumference and Blood Glucose Concentration in Patients with Overweight and Obesity. The DISFRUTE Study: A Randomised Trial in Primary Care. | https://doi.org/10.3390/nu12041149 | Not relevant to our study |
| 2020 | [Electroacupuncture combined with lifestyle control on obese nonalcoholic fatty liver disease: a randomized controlled trial]. | https://doi.org/10.13703/j.0255-2930.20190201-k00034 | Study on the treatment of traditional Chinese medicine |
| 2016 | Long-term lifestyle interventions in middle-aged and elderly men with nonalcoholic fatty liver disease: a randomized controlled trial. | https://doi.org/10.1038/srep36783 | Non-drug treatment research |
| 2017 | Urinary metabolomics analysis identifies key biomarkers of different stages of nonalcoholic fatty liver disease. | https://doi.org/10.3748/wjg.v23.i15.2771 | Basic research |
| 2020 | Whole-grain consumption and its effects on hepatic steatosis and liver enzymes in patients with non-alcoholic fatty liver disease: a randomised controlled clinical trial. | https://doi.org/10.1017/s0007114519002769 | The study population was not diagnosed with diabetes |
| 2018 | Effectiveness of a carbohydrate restricted diet to treat non-alcoholic fatty liver disease in adolescents with obesity: Trial design and methodology. | https://doi.org/10.1016/j.cct.2018.03.014 | Non-drug treatment research |
| 2020 | Electroacupuncture Versus Aerobic Interval Training on Liver Functions in Patients with Nonalcoholic Fatty Liver. | https://doi.org/10.1089/acm.2019.0182 | Non-drug treatment research |
| 2022 | Two untargeted metabolomics reveals yogurt-associated metabolic alterations in women with multiple metabolic disorders from a randomized controlled study. | https://doi.org/10.1016/j.jprot.2021.104394 | Not relevant to our study |
| 2019 | Different acute effects of fructose and glucose administration on hepatic fat content. | https://doi.org/10.1093/ajcn/nqy386 | Not relevant to our study |
| 2016 | Conjugated linoleic acid improves glycemic response, lipid profile, and oxidative stress in obese patients with non-alcoholic fatty liver disease: a randomized controlled clinical trial. | https://doi.org/10.3325/cmj.2016.57.331 | The study population was not diagnosed with diabetes |
| 2017 | Glucose homeostasis, insulin resistance and inflammatory biomarkers in patients with non-alcoholic fatty liver disease: Beneficial effects of supplementation with microalgae Chlorella vulgaris: A double-blind placebo-controlled randomized clinical trial. | https://doi.org/10.1016/j.clnu.2016.07.004 | The study population was not diagnosed with diabetes |
| 2018 | Utility and cost evaluation of multiparametric magnetic resonance imaging for the assessment of non-alcoholic fatty liver disease. | https://doi.org/10.1111/apt.14469 | Not relevant to our study |
| 2020 | [The potential role of organic and conventional yoghurt consumption in the treatment of non-alcoholic fatty liver disease]. | https://doi.org/10.1556/650.2020.31839 | The study population was not diagnosed with diabetes |
| 2022 | Vitamin D and nonalcoholic fatty liver disease in children: a randomized controlled clinical trial. | https://doi.org/10.1007/s00431-021-04243-4 | The study population was not diagnosed with diabetes |
| 2018 | Local care and treatment of liver disease (LOCATE) - A cluster-randomized feasibility study to discover, assess and manage early liver disease in primary care. | https://doi.org/10.1371/journal.pone.0208798 | Not relevant to our study |
| 2015 | Comparative clinical study between the effect of fenofibrate alone and its combination with pentoxifylline on biochemical parameters and liver stiffness in patients with non-alcoholic fatty liver disease. | https://doi.org/10.1007/s12072-015-9633-1 | The study population was not diagnosed with diabetes |
| 2017 | Randomized Controlled Trial of a MUFA or Fiber-Rich Diet on Hepatic Fat in Prediabetes. | https://doi.org/10.1210/jc.2016-3722 | Prediabetes |
| 2005 | Management of fatty liver disease with vitamin E and C compared to ursodeoxycholic acid treatment. |  | The study population was not diagnosed with diabetes |
| 2019 | Effect of Soy Milk on Metabolic Status of Patients with Nonalcoholic Fatty Liver Disease: A Randomized Clinical Trial. | https://doi.org/10.1080/07315724.2018.1479990 | The study population was not diagnosed with diabetes |
| 2014 | Synbiotic supplementation in nonalcoholic fatty liver disease: a randomized, double-blind, placebo-controlled pilot study. | https://doi.org/10.3945/ajcn.113.068890 | The study population was not diagnosed with diabetes |
| 2021 | Short-term orlistat therapy improves fatty infiltration indices and liver fibrosis scores in patients with non-alcoholic fatty liver disease and metabolic syndrome. | https://doi.org/10.1016/j.ajg.2020.12.005 | The study population was not diagnosed with diabetes |
| 2023 | Effect of alternate day fasting combined with aerobic exercise on non-alcoholic fatty liver disease: A randomized controlled trial. | https://doi.org/10.1016/j.cmet.2022.12.001 | Non-drug treatment research |
| 2023 | Alternate-Day Fasting Combined with Exercise: Effect on Sleep in Adults with Obesity and NAFLD. | https://doi.org/10.3390/nu15061398 | Non-drug treatment research |
| 2010 | Effect of fenofibrate and niacin on intrahepatic triglyceride content, very low-density lipoprotein kinetics, and insulin action in obese subjects with nonalcoholic fatty liver disease. | https://doi.org/10.1210/jc.2009-2622 | The study population was not diagnosed with diabetes |
| 2013 | Using transient elastography to detect chronic liver diseases in a primary care nurse consultancy. | https://doi.org/10.1097/nnr.0000000000000001 | Not relevant to our study |
| 2018 | Prevalence of hepatic steatosis as assessed by controlled attenuation parameter (CAP) in subjects with metabolic risk factors in primary care. A population-based study. | https://doi.org/10.1371/journal.pone.0200656 | Not relevant to our study |
| 2015 | The effects of resveratrol supplementation on cardiovascular risk factors in patients with non-alcoholic fatty liver disease: a randomised, double-blind, placebo-controlled study. | https://doi.org/10.1017/s0007114515002433 | The study population was not diagnosed with diabetes |
| 2014 | Resveratrol supplementation improves inflammatory biomarkers in patients with nonalcoholic fatty liver disease. | https://doi.org/10.1016/j.nutres.2014.09.005 | The study population was not diagnosed with diabetes |
| 2017 | Effects of Probiotics on Nonalcoholic Fatty Liver Disease in Obese Children and Adolescents. | https://doi.org/10.1097/mpg.0000000000001422 | The study population was children |
| 2010 | Effect of Xuezhikang Capsule on serum tumor necrosis factor-alpha and interleukin-6 in patients with nonalcoholic fatty liver disease and hyperlipidemia. | https://doi.org/10.1007/s11655-010-0119-7 | The study population was not diagnosed with diabetes |
| 2022 | Elevated serum phosphatidylcholine (16:1/22:6) levels promoted by fish oil and vitamin D(3) are highly correlated with biomarkers of non-alcoholic fatty liver disease in Chinese subjects. | https://doi.org/10.1039/d2fo02349k | Not relevant to our study |
| 2014 | Oral coenzyme Q10 supplementation in patients with nonalcoholic fatty liver disease: effects on serum vaspin, chemerin, pentraxin 3, insulin resistance and oxidative stress. | https://doi.org/10.1016/j.arcmed.2014.11.001 | Not relevant to our study |
| 2016 | Functions of Coenzyme Q10 Supplementation on Liver Enzymes, Markers of Systemic Inflammation, and Adipokines in Patients Affected by Nonalcoholic Fatty Liver Disease: A Double-Blind, Placebo-Controlled, Randomized Clinical Trial. | https://doi.org/10.1080/07315724.2015.1021057 | The study population was not diagnosed with diabetes |
| 2020 | No beneficial effects of resveratrol supplementation on atherogenic risk factors in patients with nonalcoholic fatty liver disease. | https://doi.org/10.1024/0300-9831/a000528 | The study population was not diagnosed with diabetes |
| 2020 | The Effect of Zinc Supplementation on Steatosis Severity and Liver Function Enzymes in Overweight/Obese Patients with Mild to Moderate Non-alcoholic Fatty Liver Following Calorie-Restricted Diet: a Double-Blind, Randomized Placebo-Controlled Trial. | https://doi.org/10.1007/s12011-019-02015-8 | The study population was not diagnosed with diabetes |
| 2020 | The effects of zinc supplementation on metabolic profile and oxidative stress in overweight/obese patients with non-alcoholic fatty liver disease: A randomized, double-blind, placebo-controlled trial. | https://doi.org/10.1016/j.jtemb.2020.126635 | The study population was not diagnosed with diabetes |
| 2019 | Evaluation of the Effect Derived from Silybin with Vitamin D and Vitamin E Administration on Clinical, Metabolic, Endothelial Dysfunction, Oxidative Stress Parameters, and Serological Worsening Markers in Nonalcoholic Fatty Liver Disease Patients. | https://doi.org/10.1155/2019/8742075 | The study population was not diagnosed with diabetes |
| 2006 | A new silybin-vitamin E-phospholipid complex improves insulin resistance and liver damage in patients with non-alcoholic fatty liver disease: preliminary observations. | https://doi.org/10.1136/gut.2006.091967 | The study population was not diagnosed with diabetes |
| 2021 | Machine learning algorithm outperforms fibrosis markers in predicting significant fibrosis in biopsy-confirmed NAFLD. | https://doi.org/10.1002/jhbp.972 | Not relevant to our study |
| 2023 | Mitochondrial pyruvate carrier inhibition initiates metabolic crosstalk to stimulate branched chain amino acid catabolism. | https://doi.org/10.1016/j.molmet.2023.101694 | Basic research |
| 2016 | Beneficial Effect of Synbiotic Supplementation on Hepatic Steatosis and Anthropometric Parameters, But Not on Gut Permeability in a Population with Nonalcoholic Steatohepatitis. | https://doi.org/10.3390/nu8070397 | The study population was not diagnosed with diabetes |
| 2022 | Citrus Bergamia and Cynara Cardunculus Reduce Serum Uric Acid in Individuals with Non-Alcoholic Fatty Liver Disease. | https://doi.org/10.3390/medicina58121728 | The study population was not diagnosed with diabetes |
| 2020 | Randomized Clinical Trial: Bergamot Citrus and Wild Cardoon Reduce Liver Steatosis and Body Weight in Non-diabetic Individuals Aged Over 50 Years. | https://doi.org/10.3389/fendo.2020.00494 | The study population was not diagnosed with diabetes |
| 2022 | A new nutraceutical (Livogen Plus®) improves liver steatosis in adults with non-alcoholic fatty liver disease. | https://doi.org/10.1186/s12967-022-03579-1 | The study population was not diagnosed with diabetes |
| 2021 | Randomised clinical trial: semaglutide versus placebo reduced liver steatosis but not liver stiffness in subjects with non-alcoholic fatty liver disease assessed by magnetic resonance imaging. | https://doi.org/10.1111/apt.16608 | The study population was not diagnosed with diabetes |
| 2022 | Effect of Dietary Supplementation with Eufortyn(®) Colesterolo Plus on Serum Lipids, Endothelial Reactivity, Indexes of Non-Alcoholic Fatty Liver Disease and Systemic Inflammation in Healthy Subjects with Polygenic Hypercholesterolemia: The ANEMONE Study. | https://doi.org/10.3390/nu14102099 | The study population was not diagnosed with diabetes |
| 2012 | Effects of losartan and amlodipine alone or combined with simvastatin in hypertensive patients with nonalcoholic hepatic steatosis. | https://doi.org/10.1097/MEG.0b013e32834ba188 | The study population was not diagnosed with diabetes |
| 2011 | Atorvastatin and antioxidants for the treatment of nonalcoholic fatty liver disease: the St Francis Heart Study randomized clinical trial. | https://doi.org/10.1038/ajg.2010.299 | The study population was not diagnosed with diabetes |
| 2021 | A randomized controlled trial comparing the effects of Vitamin E, Ursodeoxycholic acid and Pentoxifylline on Egyptian non-alcoholic steatohepatitis patients. | https://doi.org/10.26355/eurrev_202112_27442 | The study population was not diagnosed with diabetes |
| 2021 | Pharmacodynamic effects of direct AMP kinase activation in humans with insulin resistance and non-alcoholic fatty liver disease: A phase 1b study. | https://doi.org/10.1016/j.xcrm.2021.100474 | Basic research |
| 2020 | Effects of tesamorelin on hepatic transcriptomic signatures in HIV-associated NAFLD. | https://doi.org/10.1172/jci.insight.140134 | Basic research |
| 2021 | Clinical Predictors of Liver Fibrosis Presence and Progression in Human Immunodeficiency Virus-Associated Nonalcoholic Fatty Liver Disease. | https://doi.org/10.1093/cid/ciaa382 | Not relevant to our study |
| 2019 | Effectiveness of two physical activity programs on non-alcoholic fatty liver disease. a randomized controlled clinical trial. | https://doi.org/10.31053/1853.0605.v76.n1.21638 | Non-drug treatment research |
| 2020 | Physical Activity and Low Glycemic Index Mediterranean Diet: Main and Modification Effects on NAFLD Score. Results from a Randomized Clinical Trial. | https://doi.org/10.3390/nu13010066 | Non-drug treatment research |
| 2021 | A Randomized, Controlled Trial of the Pan-PPAR Agonist Lanifibranor in NASH. | https://doi.org/10.1056/NEJMoa2036205 | The study population was not diagnosed with diabetes |
| 2021 | Have We Learnt all from IMPROVE-IT? Part I. Core Results and Subanalyses on the Effects of Ezetimibe Added to Statin Therapy Related to Age, Gender and Selected Chronic Diseases (Kidney Disease, Diabetes Mellitus and Non-Alcoholic Fatty Liver Disease). | https://doi.org/10.2174/1570161118999200727224946 | Not relevant to our study |
| 2022 | Effect of progressive resistance training with weight loss compared with weight loss alone on the fatty liver index in older adults with type 2 diabetes: secondary analysis of a 12-month randomized controlled trial. | https://doi.org/10.1136/bmjdrc-2022-002950 | Non-drug treatment research |
| 2021 | Emricasan to prevent new decompensation in patients with NASH-related decompensated cirrhosis. | https://doi.org/10.1016/j.jhep.2020.09.029 | Not relevant to our study |
| 2016 | Efficacy and safety study of cenicriviroc for the treatment of non-alcoholic steatohepatitis in adult subjects with liver fibrosis: CENTAUR Phase 2b study design. | https://doi.org/10.1016/j.cct.2016.02.012 | The study population was not diagnosed with diabetes |
| 2018 | A randomized, placebo-controlled trial of cenicriviroc for treatment of nonalcoholic steatohepatitis with fibrosis. | https://doi.org/10.1002/hep.29477 | The study population was not diagnosed with diabetes |
| 2018 | Effect of liraglutide on ectopic fat in polycystic ovary syndrome: A randomized clinical trial. | https://doi.org/10.1111/dom.13053 | Not relevant to our study |
| 2021 | Effect of empagliflozin on ectopic fat stores and myocardial energetics in type 2 diabetes: the EMPACEF study. | https://doi.org/10.1186/s12933-021-01237-2 | Animal experiment |
| 2020 | Randomized placebo-controlled trial of emricasan for non-alcoholic steatohepatitis-related cirrhosis with severe portal hypertension. | https://doi.org/10.1016/j.jhep.2019.12.010 | The study population was not diagnosed with diabetes |
| 2010 | Metformin versus dietary treatment in nonalcoholic hepatic steatosis: a randomized study. | https://doi.org/10.1038/ijo.2010.40 | The study population was not diagnosed with diabetes |
| 2009 | Importance of changes in adipose tissue insulin resistance to histological response during thiazolidinedione treatment of patients with nonalcoholic steatohepatitis. | https://doi.org/10.1002/hep.23116 | The study population was not diagnosed with diabetes |
| 2021 | PPAR-γ-induced changes in visceral fat and adiponectin levels are associated with improvement of steatohepatitis in patients with NASH. | https://doi.org/10.1111/liv.15005 | The study population was not diagnosed with diabetes |
| 2008 | Effect of a dietary-induced weight loss on liver enzymes in obese subjects. | https://doi.org/10.1093/ajcn/87.5.1141 | Not relevant to our study |
| 2021 | Relationship of Enhanced Liver Fibrosis Score with Pediatric Nonalcoholic Fatty Liver Disease Histology and Response to Vitamin E or Metformin. | https://doi.org/10.1016/j.jpeds.2021.08.012 | Not relevant to our study |
| 2021 | Saroglitazar, a PPAR-α/γ Agonist, for Treatment of NAFLD: A Randomized Controlled Double-Blind Phase 2 Trial. | https://doi.org/10.1002/hep.31843 | The study population was not diagnosed with diabetes |
| 2018 | Treatment of non-alcoholic steatohepatitis patients with vitamin D: a double-blinded, randomized, placebo-controlled pilot study. | https://doi.org/10.1080/00365521.2018.1501091 | The study population was not diagnosed with diabetes |
| 2023 | A Mediterranean and low-fat dietary intervention in non-alcoholic fatty liver disease patients: Exploring participant experience and perceptions about dietary change. | https://doi.org/10.1111/jhn.13069 | Non-drug treatment research |
| 2022 | Impact of a Mediterranean diet on hepatic and metabolic outcomes in non-alcoholic fatty liver disease: The MEDINA randomised controlled trial. | https://doi.org/10.1111/liv.15264 | Non-drug treatment research |
| 2009 | Angiotensin-receptor blockers as therapy for mild-to-moderate hypertension-associated non-alcoholic steatohepatitis. | https://doi.org/10.3748/wjg.15.942 | The study population was not diagnosed with diabetes |
| 2019 | The beneficial effects of Mediterranean diet over low-fat diet may be mediated by decreasing hepatic fat content. | https://doi.org/10.1016/j.jhep.2019.04.013 | Non-drug treatment research |
| 2019 | Turmeric and chicory seed have beneficial effects on obesity markers and lipid profile in non-alcoholic fatty liver disease (NAFLD). | https://doi.org/10.1024/0300-9831/a000568 | The study population was not diagnosed with diabetes |
| 2019 | The clinical effects of purslane (Portulaca oleracea) seeds on metabolic profiles in patients with nonalcoholic fatty liver disease: A randomized controlled clinical trial. | https://doi.org/10.1002/ptr.6342 | The study population was not diagnosed with diabetes |
| 2019 | Effects of Dietary Intervention on Gut Microbiota and Metabolic-Nutritional Profile of Outpatients with Non-Alcoholic Steatohepatitis: a Randomized Clinical Trial. | https://doi.org/10.15403/jgld-197 | Not relevant to our study |
| 2018 | Effect of tele-nursing in the improving of the ultrasound findings in patients with nonalcoholic fatty liver diseases: A Randomized Clinical Trial study. | https://doi.org/10.17533/udea.iee.v36n3e09 | Non-drug treatment research |
| 2022 | Exercise dose on hepatic fat and cardiovascular health in adolescents with excess of adiposity. | https://doi.org/10.1111/ijpo.12869 | Not relevant to our study |
| 2021 | The effect of pomegranate extract on anthropometric indices, serum lipids, glycemic indicators, and blood pressure in patients with nonalcoholic fatty liver disease: A randomized double-blind clinical trial. | https://doi.org/10.1002/ptr.7249 | The study population was not diagnosed with diabetes |
| 2020 | Effects of a carbohydrate-restricted diet on hepatic lipid content in adolescents with non-alcoholic fatty liver disease: A pilot, randomized trial. | https://doi.org/10.1111/ijpo.12630 | Animal experiment |
| 2023 | Postprandial dysfunction in fatty liver disease. | https://doi.org/10.14814/phy2.15653 | Not relevant to our study |
| 2020 | Hepatic de novo lipogenesis is suppressed and fat oxidation is increased by omega-3 fatty acids at the expense of glucose metabolism. | https://doi.org/10.1136/bmjdrc-2019-000871 | Not relevant to our study |
| 2022 | Improvement of non-invasive tests of liver steatosis and fibrosis as indicators for non-alcoholic fatty liver disease in type 2 diabetes mellitus patients with elevated cardiovascular risk profile using the PPAR-α/γ agonist aleglitazar. | https://doi.org/10.1371/journal.pone.0277706 | The study population was not diagnosed with NAFLD |
| 2007 | Effect of tiaozhi yanggan decoction in treating patients with non-alcoholic fatty liver. | https://doi.org/10.1007/s11655-007-0275-6 | Study on the treatment of traditional Chinese medicine |
| 2012 | Choline intake in a large cohort of patients with nonalcoholic fatty liver disease. | https://doi.org/10.3945/ajcn.111.020156 | Not relevant to our study |
| 2014 | Effects of bayberry juice on inflammatory and apoptotic markers in young adults with features of non-alcoholic fatty liver disease. | https://doi.org/10.1016/j.nut.2013.07.023 | Not relevant to our study |
| 2022 | Concentrated fish oil ameliorates non-alcoholic fatty liver disease by regulating fibroblast growth factor 21-adiponectin axis. | https://doi.org/10.1016/j.nut.2022.111659 | Not relevant to our study |
| 2022 | The effects of fish oil plus vitamin D(3) intervention on non-alcoholic fatty liver disease: a randomized controlled trial. | https://doi.org/10.1007/s00394-021-02772-0 | The study population was not diagnosed with diabetes |
| 2021 | Pharmacologic inhibition of ketohexokinase prevents fructose-induced metabolic dysfunction. | https://doi.org/10.1016/j.molmet.2021.101196 | Animal experiment |
| 2017 | Less liver fibrosis in metabolically healthy compared with metabolically unhealthy obese patients with non-alcoholic fatty liver disease. | https://doi.org/10.1016/j.diabet.2017.02.007 | Not relevant to our study |
| 2015 | Treatment response in the PIVENS trial is associated with decreased Hedgehog pathway activity. | https://doi.org/10.1002/hep.27235 | Not relevant to our study |
| 2020 | An energy-restricted high-protein diet supplemented with β-cryptoxanthin alleviated oxidative stress and inflammation in nonalcoholic fatty liver disease: a randomized controlled trial. | https://doi.org/10.1016/j.nutres.2019.08.009 | The study population was not diagnosed with diabetes |
| 2020 | A hypocaloric high-protein diet supplemented with β-cryptoxanthin improves non-alcoholic fatty liver disease: a randomized controlled trial. | https://doi.org/10.1186/s12876-020-01502-w | The study population was not diagnosed with diabetes |
| 2012 | The efficacy of licorice root extract in decreasing transaminase activities in non-alcoholic fatty liver disease: a randomized controlled clinical trial. | https://doi.org/10.1002/ptr.3728 | The study population was not diagnosed with diabetes |
| 2011 | Resistance exercise reduces liver fat and its mediators in non-alcoholic fatty liver disease independent of weight loss. | https://doi.org/10.1136/gut.2011.242073 | Non-drug treatment research |
| 2015 | Modified high-intensity interval training reduces liver fat and improves cardiac function in non-alcoholic fatty liver disease: a randomized controlled trial. | https://doi.org/10.1042/cs20150308 | Non-drug treatment research |
| 2018 | Clinical and metabolic effects associated with weight changes and obeticholic acid in non-alcoholic steatohepatitis. | https://doi.org/10.1111/apt.14492 | The study population was not diagnosed with diabetes |
| 2022 | Efficacy and safety of evogliptin in patients with type 2 diabetes and non-alcoholic fatty liver disease: A multicentre, double-blind, randomized, comparative trial. | https://doi.org/10.1111/dom.14623 | Only one article on the same kind of intervention |
| 2012 | Evaluation of short-term safety and efficacy of HMG-CoA reductase inhibitors in hypercholesterolemic patients with elevated serum alanine transaminase concentrations: PITCH study (PITavastatin versus atorvastatin to evaluate the effect on patients with hypercholesterolemia and mild to moderate hepatic damage). | https://doi.org/10.1016/j.jacl.2012.01.009 | Not relevant to our study |
| 2014 | Randomized, vitamin E-controlled trial of bicyclol plus metformin in non-alcoholic fatty liver disease patients with impaired fasting glucose. | https://doi.org/10.1007/s40261-013-0136-3 | Prediabetes |
| 2019 | Evaluation of metformin therapy using controlled attenuation parameter and transient elastography in patients with non-alcoholic fatty liver disease. | https://doi.org/10.1016/j.pharep.2018.10.013 | The study population was not diagnosed with diabetes |
| 2023 | Effect of Calorie-Unrestricted Low-Carbohydrate, High-Fat Diet Versus High-Carbohydrate, Low-Fat Diet on Type 2 Diabetes and Nonalcoholic Fatty Liver Disease : A Randomized Controlled Trial. | https://doi.org/10.7326/m22-1787 | Non-drug treatment research |
| 2020 | A pilot study of the effect of curcumin on epigenetic changes and DNA damage among patients with non-alcoholic fatty liver disease: A randomized, double-blind, placebo-controlled, clinical trial. | https://doi.org/10.1016/j.ctim.2020.102447 | Not relevant to our study |
| 2021 | Combined exenatide and dapagliflozin has no additive effects on reduction of hepatocellular lipids despite better glycaemic control in patients with type 2 diabetes mellitus treated with metformin: EXENDA, a 24-week, prospective, randomized, placebo-controlled pilot trial. | https://doi.org/10.1111/dom.14319 | The study population was not diagnosed with NAFLD |
| 2018 | Simtuzumab Is Ineffective for Patients With Bridging Fibrosis or Compensated Cirrhosis Caused by Nonalcoholic Steatohepatitis. | https://doi.org/10.1053/j.gastro.2018.07.006 | Not relevant to our study |
| 2022 | Aldafermin in patients with non-alcoholic steatohepatitis (ALPINE 2/3): a randomised, double-blind, placebo-controlled, phase 2b trial. | https://doi.org/10.1016/s2468-1253(22)00017-6 | The study population was not diagnosed with diabetes |
| 2020 | Insulin sensitizer MSDC-0602K in non-alcoholic steatohepatitis: A randomized, double-blind, placebo-controlled phase IIb study. | https://doi.org/10.1016/j.jhep.2019.10.023 | The study population was not diagnosed with diabetes |
| 2021 | Effects of Resmetirom on Noninvasive Endpoints in a 36-Week Phase 2 Active Treatment Extension Study in Patients With NASH. | https://doi.org/10.1002/hep4.1657 | The study population was not diagnosed with diabetes |
| 2019 | Resmetirom (MGL-3196) for the treatment of non-alcoholic steatohepatitis: a multicentre, randomised, double-blind, placebo-controlled, phase 2 trial. | https://doi.org/10.1016/s0140-6736(19)32517-6 | The study population was not diagnosed with diabetes |
| 2021 | A structurally optimized FXR agonist, MET409, reduced liver fat content over 12 weeks in patients with non-alcoholic steatohepatitis. | https://doi.org/10.1016/j.jhep.2021.01.047 | The study population was not diagnosed with diabetes |
| 2021 | Safety, Tolerability, and Biologic Activity of AXA1125 and AXA1957 in Subjects With Nonalcoholic Fatty Liver Disease. | https://doi.org/10.14309/ajg.0000000000001375 | The study population was not diagnosed with diabetes |
| 2020 | Semaglutide for the treatment of non-alcoholic steatohepatitis: Trial design and comparison of non-invasive biomarkers. | https://doi.org/10.1016/j.cct.2020.106174 | The study population was not diagnosed with diabetes |
| 2018 | Utility and variability of three non-invasive liver fibrosis imaging modalities to evaluate efficacy of GR-MD-02 in subjects with NASH and bridging fibrosis during a phase-2 randomized clinical trial. | https://doi.org/10.1371/journal.pone.0203054 | Not relevant to our study |
| 2009 | Orlistat for overweight subjects with nonalcoholic steatohepatitis: A randomized, prospective trial. | https://doi.org/10.1002/hep.22575 | The study population was not diagnosed with diabetes |
| 2004 | A pilot study of orlistat treatment in obese, non-alcoholic steatohepatitis patients. | https://doi.org/10.1111/j.1365-2036.2004.02153.x | The study population was not diagnosed with diabetes |
| 2020 | A randomized, placebo-controlled trial of emricasan in patients with NASH and F1-F3 fibrosis. | https://doi.org/10.1016/j.jhep.2019.11.024 | The study population was not diagnosed with diabetes |
| 2021 | A phase 2, proof of concept, randomised controlled trial of berberine ursodeoxycholate in patients with presumed non-alcoholic steatohepatitis and type 2 diabetes. | https://doi.org/10.1038/s41467-021-25701-5 | Only one article on the same kind of intervention |
| 2022 | Licogliflozin for nonalcoholic steatohepatitis: a randomized, double-blind, placebo-controlled, phase 2a study. | https://doi.org/10.1038/s41591-022-01861-9 | The study population was not diagnosed with diabetes |
| 2016 | Randomised clinical study: GR-MD-02, a galectin-3 inhibitor, vs. placebo in patients having non-alcoholic steatohepatitis with advanced fibrosis. | https://doi.org/10.1111/apt.13816 | The study population was not diagnosed with diabetes |
| 2022 | Rencofilstat, a cyclophilin inhibitor: A phase 2a, multicenter, single-blind, placebo-controlled study in F2/F3 NASH. | https://doi.org/10.1002/hep4.2100 | Not relevant to our study |
| 2021 | Efficacy and Safety of Aldafermin, an Engineered FGF19 Analog, in a Randomized, Double-Blind, Placebo-Controlled Trial of Patients With Nonalcoholic Steatohepatitis. | https://doi.org/10.1053/j.gastro.2020.08.004 | The study population was not diagnosed with diabetes |
| 2020 | A blood-based biomarker panel (NIS4) for non-invasive diagnosis of non-alcoholic steatohepatitis and liver fibrosis: a prospective derivation and global validation study. | https://doi.org/10.1016/s2468-1253(20)30252-1 | Not relevant to our study |
| 2018 | NGM282 for treatment of non-alcoholic steatohepatitis: a multicentre, randomised, double-blind, placebo-controlled, phase 2 trial. | https://doi.org/10.1016/s0140-6736(18)30474-4 | The study population was not diagnosed with diabetes |
| 2021 | Efruxifermin in non-alcoholic steatohepatitis: a randomized, double-blind, placebo-controlled, phase 2a trial. | https://doi.org/10.1038/s41591-021-01425-3 | The study population was not diagnosed with diabetes |
| 2023 | Evaluation of PXL065 - deuterium-stabilized (R)-pioglitazone in patients with NASH: A phase II randomized placebo-controlled trial (DESTINY-1). | https://doi.org/10.1016/j.jhep.2023.02.004 | The study population was not diagnosed with diabetes |
| 2003 | Vitamin E and vitamin C treatment improves fibrosis in patients with nonalcoholic steatohepatitis. | https://doi.org/10.1111/j.1572-0241.2003.08699.x | The study population was not diagnosed with diabetes |
| 2020 | Selonsertib for patients with bridging fibrosis or compensated cirrhosis due to NASH: Results from randomized phase III STELLAR trials. | https://doi.org/10.1016/j.jhep.2020.02.027 | The study population was not diagnosed with diabetes |
| 2020 | Effects of Novel Dual GIP and GLP-1 Receptor Agonist Tirzepatide on Biomarkers of Nonalcoholic Steatohepatitis in Patients With Type 2 Diabetes. | https://doi.org/10.2337/dc19-1892 | Only one article on the same kind of intervention |
| 2013 | Long-lasting improvements in liver fat and metabolism despite body weight regain after dietary weight loss. | https://doi.org/10.2337/dc13-0102 | Not relevant to our study |
| 2012 | Fetuin A in nonalcoholic fatty liver disease: in vivo and in vitro studies. | https://doi.org/10.1530/eje-11-0864 | Not relevant to our study |
| 2009 | Metformin in patients with non-alcoholic fatty liver disease: a randomized, controlled trial. | https://doi.org/10.1080/00365520902845268 | The study population was not diagnosed with diabetes |
| 2022 | Hepatoprotective effect of combination of L-carnitine and magnesium-hydroxide in nonalcoholic fatty liver disease patients: a double-blinded randomized controlled pilot study. | https://doi.org/10.26355/eurrev_202210_30023 | The study population was not diagnosed with diabetes |
| 2022 | A Freshwater Fish-Based Diet Alleviates Liver Steatosis by Modulating Gut Microbiota and Metabolites: A Clinical Randomized Controlled Trial in Chinese Participants With Nonalcoholic Fatty Liver Disease. | https://doi.org/10.14309/ajg.0000000000001885 | Non-drug treatment research |
| 2016 | Placebo-controlled, randomised clinical trial: high-dose resveratrol treatment for non-alcoholic fatty liver disease. | https://doi.org/10.3109/00365521.2015.1107620 | The study population was not diagnosed with diabetes |
| 2017 | Circulating sCD36 levels in patients with non-alcoholic fatty liver disease and controls. | https://doi.org/10.1038/ijo.2016.223 | Not relevant to our study |
| 2021 | Efficacy and Safety of Biphenyl Dimethyl Dicarboxylate and Ursodeoxycholic Acid Combination in Chronic Hepatitis Related to Metabolic Syndrome Components. | https://doi.org/10.4166/kjg.2020.158 | Not relevant to our study |
| 2017 | Acute dietary fat intake initiates alterations in energy metabolism and insulin resistance. | https://doi.org/10.1172/jci89444 | Not relevant to our study |
| 2019 | Absolute Quantification of Apolipoproteins Following Treatment with Omega-3 Carboxylic Acids and Fenofibrate Using a High Precision Stable Isotope-labeled Recombinant Protein Fragments Based SRM Assay. | https://doi.org/10.1074/mcp.RA119.001765 | Not relevant to our study |
| 2017 | Docosahexaenoic acid enrichment in NAFLD is associated with improvements in hepatic metabolism and hepatic insulin sensitivity: a pilot study. | https://doi.org/10.1038/ejcn.2017.9 | Basic research |
| 2019 | Clinical effect of the extract of TCM Fructus akebiae combined with ursodeoxycholic acid on nonalcoholic fatty liver disease. |  | The study population was not diagnosed with diabetes |
| 2013 | Vitamin E and changes in serum alanine aminotransferase levels in patients with non-alcoholic steatohepatitis. | https://doi.org/10.1111/apt.12352 | The study population was not diagnosed with diabetes |
| 2019 | Inter-reader agreement of magnetic resonance imaging proton density fat fraction and its longitudinal change in a clinical trial of adults with nonalcoholic steatohepatitis. | https://doi.org/10.1007/s00261-018-1745-3 | Not relevant to our study |
| 2022 | Metabolic changes induced by dapagliflozin, an SGLT2 inhibitor, in Japanese patients with type 2 diabetes treated by oral anti-diabetic agents: A randomized, clinical trial. | https://doi.org/10.1016/j.diabres.2022.109781 | The study population was not diagnosed with NAFLD |
| 2020 | Combined cranberry supplementation and weight loss diet in non-alcoholic fatty liver disease: a double-blind placebo-controlled randomized clinical trial. | https://doi.org/10.1080/09637486.2020.1746957 | The study population was not diagnosed with diabetes |
| 2020 | Effect of green coffee extract supplementation on serum adiponectin concentration and lipid profile in patients with non-alcoholic fatty liver disease: A randomized, controlled trial. | https://doi.org/10.1016/j.ctim.2019.102290 | The study population was not diagnosed with diabetes |
| 2021 | The effects of Chlorella supplementation on glycemic control, lipid profile and anthropometric measures on patients with type 2 diabetes mellitus. | https://doi.org/10.1007/s00394-021-02492-5 | The study population was not diagnosed with NAFLD |
| 2019 | The effect of alpha-lipoic acid on inflammatory markers and body composition in obese patients with non-alcoholic fatty liver disease: A randomized, double-blind, placebo-controlled trial. | https://doi.org/10.1111/jcpt.12784 | The study population was not diagnosed with diabetes |
| 2017 | Effects of Exercise on Liver Fat and Metabolism in Alcohol Drinkers. | https://doi.org/10.1016/j.cgh.2017.05.001 | Not relevant to our study |
| 2017 | Exercise Reduces Liver Lipids and Visceral Adiposity in Patients With Nonalcoholic Steatohepatitis in a Randomized Controlled Trial. | https://doi.org/10.1016/j.cgh.2016.07.031 | Non-drug treatment research |
| 2022 | HISTOLOGICAL AND HISTOCHEMICAL FEATURES OF LIVER AND LUNG TISSUE IN PATIENTS WITH NONALCOHOLIC STEATOHEPATITIS AND OBESITY DEPENDING ON THE PRESENCE OF COMORBID CHRONIC OBSTRUCTIVE PULMONARY DISEASE. |  | Not relevant to our study |
| 2013 | Effect of testosterone administration on liver fat in older men with mobility limitation: results from a randomized controlled trial. | https://doi.org/10.1093/gerona/gls259 | Not relevant to our study |
| 2021 | First-in-Asian double-blind randomized trial to assess the efficacy and safety of insulin sensitizer in nonalcoholic steatohepatitis patients. | https://doi.org/10.1007/s12072-021-10242-2 | The study population was not diagnosed with diabetes |
| 2016 | [Non-alcoholic fatty liver disease of liver stagnation and spleen deficiency pattern treated with acupoint embedding therapy: a randomized controlled trial]. |  | Study on the treatment of traditional Chinese medicine |
| 2022 | The spleen-strengthening and liver-draining herbal formula treatment of non-alcoholic fatty liver disease by regulation of intestinal flora in clinical trial. | https://doi.org/10.3389/fendo.2022.1107071 | Study on the treatment of traditional Chinese medicine |
| 2019 | Effect of vitamin D supplementation on various parameters in non-alcoholic fatty liver disease patients. |  | The study population was not diagnosed with diabetes |
| 2017 | Effects of nigella sativa on various parameters in Patients of non-alcoholic fatty liver disease. |  | The study population was not diagnosed with diabetes |
| 2008 | Clinical trial: insulin-sensitizing agents may reduce consequences of insulin resistance in individuals with non-alcoholic steatohepatitis. | https://doi.org/10.1111/j.1365-2036.2008.03723.x | The study population was not diagnosed with diabetes |
| 2013 | Effects of D-002, a mixture of high molecular weight beeswax alcohols, on patients with nonalcoholic fatty liver disease. | https://doi.org/10.3904/kjim.2013.28.4.439 | The study population was not diagnosed with diabetes |
| 2020 | Hybrid Training System Improves Insulin Resistance in Patients with Nonalcoholic Fatty Liver Disease: A Randomized Controlled Pilot Study. | https://doi.org/10.1620/tjem.252.23 | Non-drug treatment research |
| 2023 | The Effect of Walking Combined with Neuromuscular Electrical Stimulation on Liver Stiffness and Insulin Resistance in Patients with Non-alcoholic Fatty Liver Disease: An Exploratory Randomized Controlled Trial. | https://doi.org/10.2739/kurumemedj.MS674001 | Not relevant to our study |
| 2021 | Effect of sour tea supplementation on liver enzymes, lipid profile, blood pressure, and antioxidant status in patients with non-alcoholic fatty liver disease: A double-blind randomized controlled clinical trial. | https://doi.org/10.1002/ptr.6826 | The study population was not diagnosed with diabetes |
| 2023 | Effectiveness of the pomegranate extract in improving hepatokines and serum biomarkers of non-alcoholic fatty liver disease: A randomized double blind clinical trial. | https://doi.org/10.1016/j.dsx.2022.102693 | The study population was not diagnosed with diabetes |
| 2013 | Resistance exercise improves autonomic regulation at rest and haemodynamic response to exercise in non-alcoholic fatty liver disease. | https://doi.org/10.1042/cs20120684 | Non-drug treatment research |
| 2015 | Omega-3 Fatty acids therapy in children with nonalcoholic Fatty liver disease: a randomized controlled trial. | https://doi.org/10.1016/j.jpeds.2015.01.056 | The study population was children |
| 2013 | Omega-3 fatty acids for treatment of non-alcoholic fatty liver disease: design and rationale of randomized controlled trial. | https://doi.org/10.1186/1471-2431-13-85 | The study population was not diagnosed with diabetes |
| 2019 | A Randomized, Controlled Trial of Vitamin D Supplementation on Cardiovascular Risk Factors, Hormones, and Liver Markers in Women with Polycystic Ovary Syndrome. | https://doi.org/10.3390/nu11010188 | Not relevant to our study |
| 2019 | Longitudinal correlations between MRE, MRI-PDFF, and liver histology in patients with non-alcoholic steatohepatitis: Analysis of data from a phase II trial of selonsertib. | https://doi.org/10.1016/j.jhep.2018.09.024 | Not relevant to our study |
| 2017 | Efficacy of nanocurcumin supplementation on insulin resistance, lipids, inflammatory factors and nesfatin among obese patients with non-alcoholic fatty liver disease (NAFLD): a trial protocol. | https://doi.org/10.1136/bmjopen-2017-016914 | A trial protocol. |
| 2017 | New botanical drug, HL tablet, reduces hepatic fat as measured by magnetic resonance spectroscopy in patients with nonalcoholic fatty liver disease: A placebo-controlled, randomized, phase II trial. | https://doi.org/10.3748/wjg.v23.i32.5977 | Not relevant to our study |
| 2005 | [Clinical study on treatment of non-alcoholic fatty liver of damp-heat syndrome type by danning tablet]. |  | Study on the treatment of traditional Chinese medicine |
| 2008 | Effectiveness of Danning Tablet in patients with non-alcoholic fatty liver of damp-heat syndrome type: a multicenter randomized controlled trial. | https://doi.org/10.3736/jcim20080205 | Study on the treatment of traditional Chinese medicine |
| 2018 | [Effect of aerobic exercise and resistance exercise in improving non-alcoholic fatty liver disease: a randomized controlled trial]. | https://doi.org/10.3760/cma.j.issn.1007-3418.2018.01.009 | Non-drug treatment research |
| 2021 | Impact of short-term overfeeding of saturated or unsaturated fat or sugars on the gut microbiota in relation to liver fat in obese and overweight adults. | https://doi.org/10.1016/j.clnu.2020.05.008 | Non-drug treatment research |
| 2023 | Effect and Safety of Pioglitazone-Metformin Tablets in the Treatment of Newly Diagnosed Type 2 Diabetes Patients with Nonalcoholic Fatty Liver Disease in Shaanxi Province: A Randomized, Double-Blinded, Double-Simulated Multicenter Study. | https://doi.org/10.1155/2023/2044090 | Only one article on the same kind of intervention |
| 2021 | Week 4 Liver Fat Reduction on MRI as an Early Predictor of Treatment Response in Participants with Nonalcoholic Steatohepatitis. | https://doi.org/10.1148/radiol.2021204325 | Not relevant to our study |
| 2012 | Children with NAFLD are more sensitive to the adverse metabolic effects of fructose beverages than children without NAFLD. | https://doi.org/10.1210/jc.2012-1370 | The study population was children |
| 2014 | Dietary fructose reduction improves markers of cardiovascular disease risk in Hispanic-American adolescents with NAFLD. | https://doi.org/10.3390/nu6083187 | The study population was children |
| 2022 | Litchi-Derived Polyphenol Alleviates Liver Steatosis and Gut Dysbiosis in Patients with Non-Alcoholic Fatty Liver Disease: A Randomized Double-Blinded, Placebo-Controlled Study. | https://doi.org/10.3390/nu14142921 | The study population was not diagnosed with diabetes |
| 2020 | Dapagliflozin plus saxagliptin add-on to metformin reduces liver fat and adipose tissue volume in patients with type 2 diabetes. | https://doi.org/10.1111/dom.14004 | Only one article on the same kind of intervention |
| 2019 | A Randomised Controlled Trial on the Effectiveness and Adherence of Modified Alternate-day Calorie Restriction in Improving Activity of Non-Alcoholic Fatty Liver Disease. | https://doi.org/10.1038/s41598-019-47763-8 | Not relevant to our study |
| 2009 | Aerobic exercise training reduces hepatic and visceral lipids in obese individuals without weight loss. | https://doi.org/10.1002/hep.23129 | Not relevant to our study |
| 2013 | No difference between high-fructose and high-glucose diets on liver triacylglycerol or biochemistry in healthy overweight men. | https://doi.org/10.1053/j.gastro.2013.07.012 | Not relevant to our study |
| 2017 | Sitagliptin in patients with non-alcoholic steatohepatitis: A randomized, placebo-controlled trial. | https://doi.org/10.3748/wjg.v23.i1.141 | The study population was not diagnosed with diabetes |
| 2016 | Impact of effective versus sham continuous positive airway pressure on liver injury in obstructive sleep apnoea: Data from randomized trials. | https://doi.org/10.1111/resp.12672 | Not relevant to our study |
| 2018 | Liver Fat Scores Moderately Reflect Interventional Changes in Liver Fat Content by a Low-Fat Diet but Not by a Low-Carb Diet. | https://doi.org/10.3390/nu10020157 | Not relevant to our study |
| 2021 | Effects of Insoluble Cereal Fibre on Body Fat Distribution in the Optimal Fibre Trial. | https://doi.org/10.1002/mnfr.202000991 | Not relevant to our study |
| 2021 | Liver fat scores do not reflect interventional changes in liver fat content induced by high-protein diets. | https://doi.org/10.1038/s41598-021-87360-2 | Not relevant to our study |
| 2022 | Predicting Factors for Metabolic Non-Response to a Complex Lifestyle Intervention-A Replication Analysis to a Randomized-Controlled Trial. | https://doi.org/10.3390/nu14224721 | Not relevant to our study |
| 2020 | Empagliflozin Effectively Lowers Liver Fat Content in Well-Controlled Type 2 Diabetes: A Randomized, Double-Blind, Phase 4, Placebo-Controlled Trial. | https://doi.org/10.2337/dc19-0641 | The study population was not diagnosed with NAFLD |
| 2022 | Effects of empagliflozin on markers of liver steatosis and fibrosis and their relationship to cardiorenal outcomes. | https://doi.org/10.1111/dom.14670 | The study population was not diagnosed with diabetes |
| 2017 | Liver Function in Patients With Nonalcoholic Fatty Liver Disease Randomized to Roux-en-Y Gastric Bypass Versus Sleeve Gastrectomy: A Secondary Analysis of a Randomized Clinical Trial. | https://doi.org/10.1097/sla.0000000000002397 | Not relevant to our study |
| 2016 | The effect of minimal dietary changes with raisins in NAFLD patients with non-significant fibrosis: a randomized controlled intervention. | https://doi.org/10.1039/c6fo01040g | Not relevant to our study |
| 2020 | Efficacy and safety of PERIOdontal treatment versus usual care for Nonalcoholic liver disease: protocol of the PERION multicenter, two-arm, open-label, randomized trial. | https://doi.org/10.1186/s13063-020-4201-y | Not relevant to our study |
| 2022 | Periodontal Treatment and Usual Care for Nonalcoholic Fatty Liver Disease: A Multicenter, Randomized Controlled Trial. | https://doi.org/10.14309/ctg.0000000000000520 | Not relevant to our study |
| 2014 | Effects of a novel therapeutic diet on liver enzymes and coagulating factors in patients with non-alcoholic fatty liver disease: A parallel randomized trial. | https://doi.org/10.1016/j.nut.2013.11.008 | Non-drug treatment research |
| 2017 | Effects of a Low-Calorie, Low-Carbohydrate Soy Containing Diet on Systemic Inflammation Among Patients with Nonalcoholic Fatty Liver Disease: A Parallel Randomized Clinical Trial. | https://doi.org/10.1055/s-0042-118707 | Non-drug treatment research |
| 2021 | Nutrigenetic Interactions Might Modulate the Antioxidant and Anti-Inflammatory Status in Mastiha-Supplemented Patients With NAFLD. | https://doi.org/10.3389/fimmu.2021.683028 | Basic research |
| 2018 | Effects of resveratrol supplementation on liver fat content in overweight and insulin-resistant subjects: A randomized, double-blind, placebo-controlled clinical trial. | https://doi.org/10.1111/dom.13268 | The study population was not diagnosed with diabetes |
| 2015 | Resolution of non-alcoholic steatohepatitis by rosuvastatin monotherapy in patients with metabolic syndrome. | https://doi.org/10.3748/wjg.v21.i25.7860 | The study population was not diagnosed with diabetes |
| 2014 | Effect of rosuvastatin on non-alcoholic steatohepatitis in patients with metabolic syndrome and hypercholesterolaemia: a preliminary report. | https://doi.org/10.2174/15701611113119990009 | A report |
| 2015 | Evaluation of Transient Elastography, Acoustic Radiation Force Impulse Imaging (ARFI), and Enhanced Liver Function (ELF) Score for Detection of Fibrosis in Morbidly Obese Patients. | https://doi.org/10.1371/journal.pone.0141649 | Not relevant to our study |
| 2018 | Improvements in clinical characteristics of patients with non-alcoholic fatty liver disease, after an intervention based on the Mediterranean lifestyle: a randomised controlled clinical trial. | https://doi.org/10.1017/s000711451800137x | Non-drug treatment research |
| 2021 | Co-supplementation of camelina oil and a prebiotic is more effective for in improving cardiometabolic risk factors and mental health in patients with NAFLD: a randomized clinical trial. | https://doi.org/10.1039/d1fo00448d | The study population was not diagnosed with diabetes |
| 2018 | Effects of isomaltulose on insulin resistance and metabolites in patients with non‑alcoholic fatty liver disease: A metabolomic analysis. | https://doi.org/10.3892/mmr.2018.9223 | The study population was not diagnosed with diabetes |
| 2020 | The effects of sumac (Rhus coriaria L.) powder supplementation in patients with non-alcoholic fatty liver disease: A randomized controlled trial. | https://doi.org/10.1016/j.ctcp.2020.101259 | The study population was not diagnosed with diabetes |
| 2021 | Inhibition of ketohexokinase in adults with NAFLD reduces liver fat and inflammatory markers: A randomized phase 2 trial. | https://doi.org/10.1016/j.medj.2021.04.007 | The study population was not diagnosed with diabetes |
| 2023 | High-Intensity Interval Training is Safe, Feasible and Efficacious in Nonalcoholic Steatohepatitis: A Randomized Controlled Trial. | https://doi.org/10.1007/s10620-022-07779-z | Non-drug treatment research |
| 2015 | Effect of aerobic exercise training dose on liver fat and visceral adiposity. | https://doi.org/10.1016/j.jhep.2015.02.022 | Non-drug treatment research |
| 2011 | Effects of moderate red wine consumption on liver fat and blood lipids: a prospective randomized study. | https://doi.org/10.3109/07853890.2011.588246 | Not relevant to our study |
| 2021 | Efficacy of combining pentoxiphylline and vitamin E versus vitamin E alone in non-alcoholic steatohepatitis- A randomized pilot study. | https://doi.org/10.1007/s12664-020-01131-x | The study population was not diagnosed with diabetes |
| 2021 | Metabolic and Hepatic Effects of Energy-Reduced Anti-Inflammatory Diet in Younger Adults with Obesity. | https://doi.org/10.1155/2021/6649142 | Not relevant to our study |
| 2014 | Accumulation of (18)F-FDG in the liver in hepatic steatosis. | https://doi.org/10.2214/ajr.13.12147 | Not relevant to our study |
| 2018 | Efficacy, safety, and tolerability of lubiprostone for the treatment of non-alcoholic fatty liver disease in adult patients with constipation: The LUBIPRONE, double-blind, randomised, placebo-controlled study design. | https://doi.org/10.1016/j.cct.2018.04.002 | The study population was not diagnosed with diabetes |
| 2020 | Lubiprostone in patients with non-alcoholic fatty liver disease: a randomised, double-blind, placebo-controlled, phase 2a trial. | https://doi.org/10.1016/s2468-1253(20)30216-8 | The study population was not diagnosed with diabetes |
| 2020 | Endogenously released GIP reduces and GLP-1 increases hepatic insulin extraction. | https://doi.org/10.1016/j.peptides.2019.170231 | Not relevant to our study |
| 2022 | Effects of a low free sugar diet on the management of nonalcoholic fatty liver disease: a randomized clinical trial. | https://doi.org/10.1038/s41430-022-01081-x | Non-drug treatment research |
| 2019 | Standardized Nigella sativa seed oil ameliorates hepatic steatosis, aminotransferase and lipid levels in non-alcoholic fatty liver disease: A randomized, double-blind and placebo-controlled clinical trial. | https://doi.org/10.1016/j.jep.2019.01.009 | The study population was not diagnosed with diabetes |
| 2017 | Comparative effects of liraglutide 3 mg vs structured lifestyle modification on body weight, liver fat and liver function in obese patients with non-alcoholic fatty liver disease: A pilot randomized trial. | https://doi.org/10.1111/dom.13007 | The study population was not diagnosed with diabetes |
| 2019 | Randomized trial comparing effects of weight loss by liraglutide with lifestyle modification in non-alcoholic fatty liver disease. | https://doi.org/10.1111/liv.14065 | The study population was not diagnosed with diabetes |
| 2023 | Comparison of the efficacy of pioglitazone and metformin on ultrasound grade and liver enzymes level in patients with non-alcoholic fatty liver disease: A randomized controlled clinical trial. | https://doi.org/10.1055/a-1997-0401 | The study population was not diagnosed with diabetes |
| 2017 | Efficacy and safety of fermented garlic extract on hepatic function in adults with elevated serum gamma-glutamyl transpeptidase levels: a double-blind, randomized, placebo-controlled trial. | https://doi.org/10.1007/s00394-016-1318-6 | Not relevant to our study |
| 2012 | Influence of elevated liver fat on circulating adipocytokines and insulin resistance in obese Hispanic adolescents. | https://doi.org/10.1111/j.2047-6310.2011.00014.x | Not relevant to our study |
| 2017 | Randomised clinical trial: the efficacy and safety of oltipraz, a liver X receptor alpha-inhibitory dithiolethione in patients with non-alcoholic fatty liver disease. | https://doi.org/10.1111/apt.13981 | The study population was not diagnosed with diabetes |
| 2017 | Bariatric surgery for nonalcoholic steatohepatitis: A clinical and cost-effectiveness analysis. | https://doi.org/10.1002/hep.28958 | Not relevant to our study |
| 2015 | [COMPARISON OF DIFFERENT TREATMENT REGIMENS IN PATIENTS WITH NONALCOHOLIC FATTY LIVER DISEASE]. |  | The study population was not diagnosed with diabetes |
| 2018 | Beneficial effects of probiotic combination with omega-3 fatty acids in NAFLD: a randomized clinical study. | https://doi.org/10.23736/s0026-4806.18.05845-7 | The study population was not diagnosed with diabetes |
| 2018 | A Multi-strain Probiotic Reduces the Fatty Liver Index, Cytokines and Aminotransferase levels in NAFLD Patients: Evidence from a Randomized Clinical Trial. | https://doi.org/10.15403/jgld.2014.1121.271.kby | The study population was not diagnosed with diabetes |
| 2021 | Metformin dose increase versus added linagliptin in non-alcoholic fatty liver disease and type 2 diabetes: An analysis of the J-LINK study. | https://doi.org/10.1111/dom.14263 | Only one article on the same kind of intervention |
| 2021 | Chromium picolinate balances the metabolic and clinical markers in nonalcoholic fatty liver disease: a randomized, double-blind, placebo-controlled trial. | https://doi.org/10.1097/meg.0000000000001830 | The study population was not diagnosed with diabetes |
| 2023 | Effects of time-restricted feeding (16/8) combined with a low-sugar diet on the management of non-alcoholic fatty liver disease: A randomized controlled trial. | https://doi.org/10.1016/j.nut.2022.111847 | Non-drug treatment research |
| 2019 | Hydrogen-rich water reduces liver fat accumulation and improves liver enzyme profiles in patients with non-alcoholic fatty liver disease: a randomized controlled pilot trial. | https://doi.org/10.1016/j.clinre.2019.03.008 | The study population was not diagnosed with diabetes |
| 2020 | The Effect of Moderate Weight Loss on a Non-Invasive Biomarker of Liver Fibrosis: A Randomised Controlled Trial. | https://doi.org/10.1159/000505667 | Not relevant to our study |
| 2021 | Acute responses of hepatic fat content to consuming fat, glucose and fructose alone and in combination in non-obese non-diabetic individuals with non-alcoholic fatty liver disease. | https://doi.org/10.26402/jpp.2021.1.05 | The study population was not diagnosed with diabetes |
| 2017 | [Optimization of therapy for hepatobiliary disorders in psoriatic patients]. | https://doi.org/10.17116/terarkh20178912204-210 | Not relevant to our study |
| 2010 | Effects of metformin and weight loss on serum alanine aminotransferase activity in the diabetes prevention program. | https://doi.org/10.1038/oby.2010.21 | Not relevant to our study |
| 2023 | Concerning issues in effect of a fruit rich diet on liver biomarkers, insulin resistance, and lipid profile in patients with non-alcoholic fatty liver disease: a randomized clinical trial"." | https://doi.org/10.1080/00365521.2022.2129440 | Non-drug treatment research |
| 2018 | Gut Permeability Might be Improved by Dietary Fiber in Individuals with Nonalcoholic Fatty Liver Disease (NAFLD) Undergoing Weight Reduction. | https://doi.org/10.3390/nu10111793 | Not relevant to our study |
| 2020 | Dietary Rapeseed Oil Supplementation Reduces Hepatic Steatosis in Obese Men-A Randomized Controlled Trial. | https://doi.org/10.1002/mnfr.202000419 | Non-drug treatment research |
| 2020 | Effect of dulaglutide on liver fat in patients with type 2 diabetes and NAFLD: randomised controlled trial (D-LIFT trial). | https://doi.org/10.1007/s00125-020-05265-7 | Only one article on the same kind of intervention |
| 2012 | Inflammatory markers in relation to nonalcoholic fatty liver disease in urban South Indians. | https://doi.org/10.1089/dia.2011.0213 | Not relevant to our study |
| 2015 | Oral Administration of OKT3 MAb to Patients with NASH, Promotes Regulatory T-cell Induction, and Alleviates Insulin Resistance: Results of a Phase IIa Blinded Placebo-Controlled Trial. | https://doi.org/10.1007/s10875-015-0160-6 | The study population was not diagnosed with diabetes |
| 2015 | Gut microbiota manipulation with prebiotics in patients with non-alcoholic fatty liver disease: a randomized controlled trial protocol. | https://doi.org/10.1186/s12876-015-0400-5 | The study population was not diagnosed with diabetes |
| 2008 | Effect of 6-month calorie restriction and exercise on serum and liver lipids and markers of liver function. | https://doi.org/10.1038/oby.2008.201 | Non-drug treatment research |
| 2010 | Treatment of nonalcoholic fatty liver disease in children: TONIC trial design. | https://doi.org/10.1016/j.cct.2009.09.001 | The study population was children |
| 2011 | Effect of vitamin E or metformin for treatment of nonalcoholic fatty liver disease in children and adolescents: the TONIC randomized controlled trial. | https://doi.org/10.1001/jama.2011.520 | The study population was children |
| 2023 | Fenofibrate Mitigates Hypertriglyceridemia in Nonalcoholic Steatohepatitis Patients Treated With Cilofexor/Firsocostat. | https://doi.org/10.1016/j.cgh.2021.12.044 | The study population was not diagnosed with diabetes |
| 2010 | Effect of a 12-month intensive lifestyle intervention on hepatic steatosis in adults with type 2 diabetes. | https://doi.org/10.2337/dc10-0856 | Non-drug treatment research |
| 2012 | Effect of colesevelam on liver fat quantified by magnetic resonance in nonalcoholic steatohepatitis: a randomized controlled trial. | https://doi.org/10.1002/hep.25731 | The study population was not diagnosed with diabetes |
| 2019 | Pinitol consumption improves liver health status by reducing oxidative stress and fatty acid accumulation in subjects with non-alcoholic fatty liver disease: A randomized, double-blind, placebo-controlled trial. | https://doi.org/10.1016/j.jnutbio.2019.03.006 | The study population was not diagnosed with diabetes |
| 2012 | Effects of aerobic versus resistance exercise without caloric restriction on abdominal fat, intrahepatic lipid, and insulin sensitivity in obese adolescent boys: a randomized, controlled trial. | https://doi.org/10.2337/db12-0214 | Non-drug treatment research |
| 2014 | [Comparison on the efficacy and safety of biphenyl dimethyl dicarboxylate and ursodeoxycholic acid in patients with abnormal alanine aminotransferase: multicenter, double-blinded, randomized, active-controlled clinical trial]. | https://doi.org/10.4166/kjg.2014.64.1.31 | Not relevant to our study |
| 2021 | Effect of Nutrition Education in NAFLD Patients Undergoing Simultaneous Hyperlipidemia Pharmacotherapy: A Randomized Controlled Trial. | https://doi.org/10.3390/nu13124453 | Non-drug treatment research |
| 2012 | Intragastric balloon significantly improves nonalcoholic fatty liver disease activity score in obese patients with nonalcoholic steatohepatitis: a pilot study. | https://doi.org/10.1016/j.gie.2012.05.023 | Not relevant to our study |
| 2010 | Berry meals and risk factors associated with metabolic syndrome. | https://doi.org/10.1038/ejcn.2010.27 | Not relevant to our study |
| 2019 | Efficacy of diacerein in reducing liver steatosis and fibrosis in patients with type 2 diabetes and non-alcoholic fatty liver disease: A randomized, placebo-controlled trial. | https://doi.org/10.1111/dom.13643 | Only one article on the same kind of intervention |
| 2010 | High-dose ursodeoxycholic acid therapy for nonalcoholic steatohepatitis: a double-blind, randomized, placebo-controlled trial. | https://doi.org/10.1002/hep.23727 | The study population was not diagnosed with diabetes |
| 2007 | Efficacy and safety of high-dose pravastatin in hypercholesterolemic patients with well-compensated chronic liver disease: Results of a prospective, randomized, double-blind, placebo-controlled, multicenter trial. | https://doi.org/10.1002/hep.21848 | Not relevant to our study |
| 2022 | Risk factors and prediction model for nonalcoholic fatty liver disease in northwest China. | https://doi.org/10.1038/s41598-022-17511-6 | Not relevant to our study |
| 2014 | [Treatment of nonalcoholic steatohepatitis by Jianpi Shugan Recipe: a multi-center, randomized, controlled clinical trial]. |  | Study on the treatment of traditional Chinese medicine |
| 2022 | Change lifestyle modification plan/transtheoretical model in non-alcoholic simple fatty liver disease: a pilot randomized study. | https://doi.org/10.1186/s12876-022-02506-4 | Not relevant to our study |
| 2010 | Treatment of non-alcoholic fatty liver disease by Qianggan Capsule. | https://doi.org/10.1007/s11655-010-0023-1 | The study population was not diagnosed with diabetes |
| 2022 | Risk Factors and Prediction Models for Nonalcoholic Fatty Liver Disease Based on Random Forest. | https://doi.org/10.1155/2022/8793659 | Not relevant to our study |
| 2015 | Efficacy of poly-unsaturated fatty acid therapy on patients with nonalcoholic steatohepatitis. | https://doi.org/10.3748/wjg.v21.i22.7008 | The study population was not diagnosed with diabetes |
| 2020 | Lifestyle Intervention Enabled by Mobile Technology on Weight Loss in Patients With Nonalcoholic Fatty Liver Disease: Randomized Controlled Trial. | https://doi.org/10.2196/14802 | Not relevant to our study |
| 2017 | Assessment of treatment response in non-alcoholic steatohepatitis using advanced magnetic resonance imaging. | https://doi.org/10.1111/apt.13951 | Not relevant to our study |
| 2009 | Efficacy and safety of very-low-calorie diet in Taiwanese: a multicenter randomized, controlled trial. | https://doi.org/10.1016/j.nut.2009.02.008 | Not relevant to our study |
| 2004 | Ursodeoxycholic acid for treatment of nonalcoholic steatohepatitis: results of a randomized trial. | https://doi.org/10.1002/hep.20092 | The study population was not diagnosed with diabetes |
| 2023 | Macronutrient composition and its effect on body composition changes during weight loss therapy in patients with non-alcoholic fatty liver disease: Secondary analysis of a randomized controlled trial. | https://doi.org/10.1016/j.nut.2023.111982 | Not relevant to our study |
| 2012 | Effect of insulin versus triple oral therapy on the progression of hepatic steatosis in type 2 diabetes. | https://doi.org/10.2310/JIM.0b013e3182621c5f | The study population was not diagnosed with NAFLD |
| 2014 | Effect of aerobic exercise and low carbohydrate diet on pre-diabetic non-alcoholic fatty liver disease in postmenopausal women and middle aged men--the role of gut microbiota composition: study protocol for the AELC randomized controlled trial. | https://doi.org/10.1186/1471-2458-14-48 | Not relevant to our study |
| 2010 | [Clinical observation on acupoint catgut embedding for treatment of non-alcoholic steatohepatitis]. |  | Study on the treatment of traditional Chinese medicine |
| 2017 | Effects of dark chocolate on endothelial function in patients with non-alcoholic steatohepatitis. | https://doi.org/10.1016/j.numecd.2017.10.027 | The study population was not diagnosed with diabetes |
| 2016 | Effects of dark chocolate on NOX-2-generated oxidative stress in patients with non-alcoholic steatohepatitis. | https://doi.org/10.1111/apt.13687 | The study population was not diagnosed with diabetes |
| 2012 | Silybin combined with phosphatidylcholine and vitamin E in patients with nonalcoholic fatty liver disease: a randomized controlled trial. | https://doi.org/10.1016/j.freeradbiomed.2012.02.008 | The study population was not diagnosed with diabetes |
| 2007 | Hepatic steatosis in hepatitis C: comparison of diabetic and nondiabetic patients in the hepatitis C antiviral long-term treatment against cirrhosis trial. | https://doi.org/10.1016/j.cgh.2006.11.002 | Not relevant to our study |
| 2023 | Semaglutide 2·4 mg once weekly in patients with non-alcoholic steatohepatitis-related cirrhosis: a randomised, placebo-controlled phase 2 trial. | https://doi.org/10.1016/s2468-1253(23)00068-7 | Not relevant to our study |
| 2018 | GS-0976 Reduces Hepatic Steatosis and Fibrosis Markers in Patients With Nonalcoholic Fatty Liver Disease. | https://doi.org/10.1053/j.gastro.2018.07.027 | The study population was not diagnosed with diabetes |
| 2018 | The ASK1 inhibitor selonsertib in patients with nonalcoholic steatohepatitis: A randomized, phase 2 trial. | https://doi.org/10.1002/hep.29514 | The study population was not diagnosed with diabetes |
| 2023 | Safety, pharmacokinetics, and pharmacodynamics of pegozafermin in patients with non-alcoholic steatohepatitis: a randomised, double-blind, placebo-controlled, phase 1b/2a multiple-ascending-dose study. | https://doi.org/10.1016/s2468-1253(22)00347-8 | The study population was not diagnosed with diabetes |
| 2021 | The Commensal Microbe Veillonella as a Marker for Response to an FGF19 Analog in NASH. | https://doi.org/10.1002/hep.31523 | Not relevant to our study |
| 2021 | TVB-2640 (FASN Inhibitor) for the Treatment of Nonalcoholic Steatohepatitis: FASCINATE-1, a Randomized, Placebo-Controlled Phase 2a Trial. | https://doi.org/10.1053/j.gastro.2021.07.025 | The study population was not diagnosed with diabetes |
| 2020 | Novel antisense inhibition of diacylglycerol O-acyltransferase 2 for treatment of non-alcoholic fatty liver disease: a multicentre, double-blind, randomised, placebo-controlled phase 2 trial. | https://doi.org/10.1016/s2468-1253(20)30186-2 | The study population was not diagnosed with diabetes |
| 2020 | Multicenter Validation of Association Between Decline in MRI-PDFF and Histologic Response in NASH. | https://doi.org/10.1002/hep.31121 | Not relevant to our study |
| 2021 | Combination Therapies Including Cilofexor and Firsocostat for Bridging Fibrosis and Cirrhosis Attributable to NASH. | https://doi.org/10.1002/hep.31622 | The study population was not diagnosed with diabetes |
| 2019 | Factors Associated With Histologic Response in Adult Patients With Nonalcoholic Steatohepatitis. | https://doi.org/10.1053/j.gastro.2018.09.021 | Not relevant to our study |
| 2015 | Ezetimibe for the treatment of nonalcoholic steatohepatitis: assessment by novel magnetic resonance imaging and magnetic resonance elastography in a randomized trial (MOZART trial). | https://doi.org/10.1002/hep.27647 | The study population was not diagnosed with diabetes |
| 2016 | Regression of Non-Alcoholic Fatty Liver by Vitamin D Supplement: A Double-Blind Randomized Controlled Clinical Trial. |  | The study population was not diagnosed with diabetes |
| 2017 | Effect of daily calcitriol supplementation with and without calcium on disease regression in non-alcoholic fatty liver patients following an energy-restricted diet: Randomized, controlled, double-blind trial. | https://doi.org/10.1016/j.clnu.2016.09.020 | The study population was not diagnosed with diabetes |
| 2008 | [Effects of Yiqi Sanju Formula on non-alcoholic fatty liver disease: a randomized controlled trial]. | https://doi.org/10.3736/jcim20080805 | Study on the treatment of traditional Chinese medicine |
| 2005 | [Diisopropylamine dichloroacetate in the treatment of nonalcoholic fatty liver disease: a multicenter random double-blind controlled trial]. |  | The study population was not diagnosed with diabetes |
| 2017 | Vitamin D(3) Loading Is Superior to Conventional Supplementation After Weight Loss Surgery in Vitamin D-Deficient Morbidly Obese Patients: a Double-Blind Randomized Placebo-Controlled Trial. | https://doi.org/10.1007/s11695-016-2437-0 | Not relevant to our study |
| 2015 | The link between obesity and vitamin D in bariatric patients with omega-loop gastric bypass surgery - a vitamin D supplementation trial to compare the efficacy of postoperative cholecalciferol loading (LOAD): study protocol for a randomized controlled trial. | https://doi.org/10.1186/s13063-015-0877-9 | Not relevant to our study |
| 2020 | Vitamin D for treatment of non-alcoholic fatty liver disease detected by transient elastography: A randomized, double-blind, placebo-controlled trial. | https://doi.org/10.1111/dom.14129 | The study population was not diagnosed with diabetes |
| 2014 | Effects of acute glucocorticoid blockade on metabolic dysfunction in patients with Type 2 diabetes with and without fatty liver. | https://doi.org/10.1152/ajpgi.00030.2014 | The study population was not diagnosed with NAFLD |
| 2017 | Changes in Liver Steatosis After Switching From Efavirenz to Raltegravir Among Human Immunodeficiency Virus-Infected Patients With Nonalcoholic Fatty Liver Disease. | https://doi.org/10.1093/cid/cix467 | Not relevant to our study |
| 2022 | Fatty Acids and Eicosanoids Change during High-Fiber Diet in NAFLD Patients-Randomized Control Trials (RCT). | https://doi.org/10.3390/nu14204310 | Non-drug treatment research |
| 2013 | Tocotrienols for normalisation of hepatic echogenic response in nonalcoholic fatty liver: a randomised placebo-controlled clinical trial. | https://doi.org/10.1186/1475-2891-12-166 | The study population was not diagnosed with diabetes |
| 2021 | A randomized controlled clinical trial comparing calcitriol versus cholecalciferol supplementation to reduce insulin resistance in patients with non-alcoholic fatty liver disease. | https://doi.org/10.1016/j.clnu.2020.11.037 | The study population was not diagnosed with diabetes |
| 2021 | The effects of co-administration of artichoke leaf extract supplementation with metformin and vitamin E in patients with nonalcoholic fatty liver disease: A randomized clinical trial. | https://doi.org/10.1002/ptr.7279 | The study population was not diagnosed with diabetes |
| 2023 | A phase I/II study of ARO-HSD, an RNA interference therapeutic, for the treatment of non-alcoholic steatohepatitis. | https://doi.org/10.1016/j.jhep.2022.11.025 | The study population was not diagnosed with diabetes |
| 2010 | L-carnitine supplementation to diet: a new tool in treatment of nonalcoholic steatohepatitis--a randomized and controlled clinical trial. | https://doi.org/10.1038/ajg.2009.719 | The study population was not diagnosed with diabetes |
| 2012 | Bifidobacterium longum with fructo-oligosaccharides in patients with non alcoholic steatohepatitis. | https://doi.org/10.1007/s10620-011-1887-4 | The study population was not diagnosed with diabetes |
| 2019 | Effect of soy milk consumption on glycemic status, blood pressure, fibrinogen and malondialdehyde in patients with non-alcoholic fatty liver disease: a randomized controlled trial. | https://doi.org/10.1016/j.ctim.2019.02.020 | Non-drug treatment research |
| 2019 | Marine omega-3 fatty acid supplementation in non-alcoholic fatty liver disease: Plasma proteomics in the randomized WELCOME* trial. | https://doi.org/10.1016/j.clnu.2018.07.037 | The study population was not diagnosed with diabetes |
| 2021 | Effects of supplementation with main coffee components including caffeine and/or chlorogenic acid on hepatic, metabolic, and inflammatory indices in patients with non-alcoholic fatty liver disease and type 2 diabetes: a randomized, double-blind, placebo-controlled, clinical trial. | https://doi.org/10.1186/s12937-021-00694-5 | Only one article on the same kind of intervention |
| 2017 | Treatment efficacy of a probiotic preparation for non-alcoholic steatohepatitis: A pilot trial. | https://doi.org/10.1111/1751-2980.12561 | The study population was not diagnosed with diabetes |
| 2019 | The Metabolic and Hepatic Impact of Two Personalized Dietary Strategies in Subjects with Obesity and Nonalcoholic Fatty Liver Disease: The Fatty Liver in Obesity (FLiO) Randomized Controlled Trial. | https://doi.org/10.3390/nu11102543 | Not relevant to our study |
| 2021 | Effects of two personalized dietary strategies during a 2-year intervention in subjects with nonalcoholic fatty liver disease: A randomized trial. | https://doi.org/10.1111/liv.14818 | The study population was not diagnosed with diabetes |
| 2017 | Isocaloric Diets High in Animal or Plant Protein Reduce Liver Fat and Inflammation in Individuals With Type 2 Diabetes. | https://doi.org/10.1053/j.gastro.2016.10.007 | Non-drug treatment research |
| 2022 | Effect of a Six-Month Lifestyle Intervention on the Physical Activity and Fitness Status of Adults with NAFLD and Metabolic Syndrome. | https://doi.org/10.3390/nu14091813 | Non-drug treatment research |
| 2021 | Effect of cranberry supplementation on liver enzymes and cardiometabolic risk factors in patients with NAFLD: a randomized clinical trial. | https://doi.org/10.1186/s12906-021-03436-6 | The study population was not diagnosed with diabetes |
| 2017 | Efficacy of nutritional interventions to lower circulating ceramides in young adults: FRUVEDomic pilot study. | https://doi.org/10.14814/phy2.13329 | Not relevant to our study |
| 2019 | Liraglutide treatment improves postprandial lipid metabolism and cardiometabolic risk factors in humans with adequately controlled type 2 diabetes: A single-centre randomized controlled study. | https://doi.org/10.1111/dom.13487 | The study population was not diagnosed with NAFLD |
| 2019 | Nonalcoholic fatty liver disease does not predict worse perioperative outcomes in bariatric surgery. | https://doi.org/10.1016/j.orcp.2019.06.006 | Not relevant to our study |
| 2015 | Impact of high dose n-3 polyunsaturated fatty acid treatment on measures of microvascular function and vibration perception in non-alcoholic fatty liver disease: results from the randomised WELCOME trial. | https://doi.org/10.1007/s00125-015-3628-2 | The study population was not diagnosed with diabetes |
| 2006 | Effects of HRT on liver enzyme levels in women with type 2 diabetes: a randomized placebo-controlled trial. | https://doi.org/10.1111/j.1365-2265.2006.02543.x | The study population was not diagnosed with NAFLD |
| 2017 | A randomised controlled trial of losartan as an anti-fibrotic agent in non-alcoholic steatohepatitis. | https://doi.org/10.1371/journal.pone.0175717 | The study population was not diagnosed with diabetes |
| 2020 | Prevalence of responders for hepatic fat, adiposity and liver enzyme levels in response to a lifestyle intervention in children with overweight/obesity: EFIGRO randomized controlled trial. | https://doi.org/10.1111/pedi.12949 | The study population was children |
| 2015 | The effect of a multidisciplinary intervention program on hepatic adiposity in overweight-obese children: protocol of the EFIGRO study. | https://doi.org/10.1016/j.cct.2015.09.017 | The study population was children |
| 2009 | [Observation on therapeutic effect of acupuncture for treatment of patients with nonalcoholic steatohepatitis]. |  | Study on the treatment of traditional Chinese medicine |
| 2022 | Polypill for prevention of cardiovascular diseases with focus on non-alcoholic steatohepatitis: the PolyIran-Liver trial. | https://doi.org/10.1093/eurheartj/ehab919 | The study population was not diagnosed with diabetes |
| 2003 | Probucol in the treatment of non-alcoholic steatohepatitis: a double-blind randomized controlled study. | https://doi.org/10.1016/s0168-8278(02)00441-5 | The study population was not diagnosed with diabetes |
| 2015 | PolyPill for Prevention of Cardiovascular Disease in an Urban Iranian Population with Special Focus on Nonalcoholic Steatohepatitis: A Pragmatic Randomized Controlled Trial within a Cohort (PolyIran - Liver) - Study Protocol. |  | The study population was not diagnosed with diabetes |
| 2022 | Leptin increases hepatic triglyceride export via a vagal mechanism in humans. | https://doi.org/10.1016/j.cmet.2022.09.020 | Not relevant to our study |
| 2015 | Urinary (1)H-NMR-based metabolic profiling of children with NAFLD undergoing VSL#3 treatment. | https://doi.org/10.1038/ijo.2015.40 | Basic research |
| 2017 | Agreement Between Magnetic Resonance Imaging Proton Density Fat Fraction Measurements and Pathologist-Assigned Steatosis Grades of Liver Biopsies From Adults With Nonalcoholic Steatohepatitis. | https://doi.org/10.1053/j.gastro.2017.06.005 | Not relevant to our study |
| 2018 | Diagnostic accuracy of magnetic resonance imaging hepatic proton density fat fraction in pediatric nonalcoholic fatty liver disease. | https://doi.org/10.1002/hep.29596 | Not relevant to our study |
| 2000 | Efficacy and safety of oral betaine glucuronate in non-alcoholic steatohepatitis. A double-blind, randomized, parallel-group, placebo-controlled prospective clinical study. | https://doi.org/10.1055/s-0031-1300279 | The study population was not diagnosed with diabetes |
| 2022 | Safety and efficacy of hydrothermal duodenal mucosal resurfacing in patients with type 2 diabetes: the randomised, double-blind, sham-controlled, multicentre REVITA-2 feasibility trial. | https://doi.org/10.1136/gutjnl-2020-323608 | Not relevant to our study |
| 2021 | The Effect of Curcumin Phytosome on the Treatment of Patients with Non-alcoholic Fatty Liver Disease: A Double-Blind, Randomized, Placebo-Controlled Trial. | https://doi.org/10.1007/978-3-030-64872-5_3 | The study population was not diagnosed with diabetes |
| 2021 | Curcumin and Piperine Combination for the Treatment of Patients with Non-alcoholic Fatty Liver Disease: A Double-Blind Randomized Placebo-Controlled Trial. | https://doi.org/10.1007/978-3-030-73234-9_2 | The study population was not diagnosed with diabetes |
| 2019 | Effect of Phytosomal Curcumin on Circulating Levels of Adiponectin and Leptin in Patients with Non-Alcoholic Fatty Liver Disease: A Randomized, Double-Blind, Placebo-Controlled Clinical Trial. | https://doi.org/10.15403/jgld-179 | The study population was not diagnosed with diabetes |
| 2019 | The effect of curcumin with piperine supplementation on pro-oxidant and antioxidant balance in patients with non-alcoholic fatty liver disease: a randomized, double-blind, placebo-controlled trial. | https://doi.org/10.1515/dmpt-2018-0040 | The study population was not diagnosed with diabetes |
| 2021 | Efficacy of phytosomal curcumin among patients with non-alcoholic fatty liver disease. | https://doi.org/10.1024/0300-9831/a000629 | The study population was not diagnosed with diabetes |
| 2017 | Effect of a Low Glycemic Index Mediterranean Diet on Non-Alcoholic Fatty Liver Disease. A Randomized Controlled Clinici Trial. | https://doi.org/10.1007/s12603-016-0809-8 | Non-drug treatment research |
| 2018 | Effect of Low (5 mg) vs. High (20-40 mg) Rosuvastatin Dose on 24h Arterial Stiffness, Central Haemodynamics, and Non-Alcoholic Fatty Liver Disease in Patients with Optimally Controlled Arterial Hypertension. | https://doi.org/10.2174/1570161115666170630122833 | The study population was not diagnosed with diabetes |
| 1997 | Abnormality in fatty acid composition of gastric mucosal phospholipids in patients with liver cirrhosis and its correction with a polyunsaturated fatty acid-enriched soft oil capsule. | https://doi.org/10.1111/j.1440-1746.1997.tb00528.x | Not relevant to our study |
| 2022 | Lifestyle Intervention for Patients with Nonalcoholic Fatty Liver Disease: A Randomized Clinical Trial Based on the Theory of Planned Behavior. | https://doi.org/10.1155/2022/3465980 | Non-drug treatment research |
| 2017 | Synbiotic supplementation in lean patients with non-alcoholic fatty liver disease: a pilot, randomised, double-blind, placebo-controlled, clinical trial. | https://doi.org/10.1017/s0007114517000204 | The study population was not diagnosed with diabetes |
| 2016 | Effects of Synbiotics Supplementation in Lean Patients with Nonalcoholic Fatty Liver Disease: Study Protocol of a Pilot Randomized Double-blind Clinical Trial. |  | The study population was not diagnosed with diabetes |
| 2021 | The Effect of Probiotics (MCP(®) BCMC(®) Strains) on Hepatic Steatosis, Small Intestinal Mucosal Immune Function, and Intestinal Barrier in Patients with Non-Alcoholic Fatty Liver Disease. | https://doi.org/10.3390/nu13093192 | The study population was not diagnosed with diabetes |
| 2014 | Dietary determinants of hepatic steatosis and visceral adiposity in overweight and obese youth at risk of type 2 diabetes. | https://doi.org/10.3945/ajcn.113.079277 | The study population was children |
| 2022 | Effect of Dietary and Lifestyle Interventions on the Amelioration of NAFLD in Patients with Metabolic Syndrome: The FLIPAN Study. | https://doi.org/10.3390/nu14112223 | Non-drug treatment research |
| 2022 | Adherence to Mediterranean Diet and NAFLD in Patients with Metabolic Syndrome: The FLIPAN Study. | https://doi.org/10.3390/nu14153186 | Not relevant to our study |
| 2021 | A pilot study of the effects of chromium picolinate supplementation on serum fetuin-A, metabolic and inflammatory factors in patients with nonalcoholic fatty liver disease: A double-blind, placebo-controlled trial. | https://doi.org/10.1016/j.jtemb.2020.126659 | The study population was not diagnosed with diabetes |
| 2020 | Effects of non-linear resistance training and curcumin supplementation on the liver biochemical markers levels and structure in older women with non-alcoholic fatty liver disease. | https://doi.org/10.1016/j.jbmt.2020.02.021 | The study population was not diagnosed with diabetes |
| 2005 | Nateglinide is useful for nonalcoholic steatohepatitis (NASH) patients with type 2 diabetes. |  | Only one article on the same kind of intervention |
| 2016 | Incidence of non-alcoholic fatty liver disease and metabolic dysfunction in first episode schizophrenia and related psychotic disorders: a 3-year prospective randomized interventional study. | https://doi.org/10.1007/s00213-016-4422-7 | Not relevant to our study |
| 2021 | Antioxidant activity of Hydroxytyrosol and Vitamin E reduces systemic inflammation in children with paediatric NAFLD. | https://doi.org/10.1016/j.dld.2020.09.021 | The study population was children |
| 2013 | Efficacy and safety of the farnesoid X receptor agonist obeticholic acid in patients with type 2 diabetes and nonalcoholic fatty liver disease. | https://doi.org/10.1053/j.gastro.2013.05.042 | Only one article on the same kind of intervention |
| 2018 | Ursodeoxycholic acid: Effects on hepatic unfolded protein response, apoptosis and oxidative stress in morbidly obese patients. | https://doi.org/10.1111/liv.13562 | Not relevant to our study |
| 2015 | Ursodeoxycholic acid exerts farnesoid X receptor-antagonistic effects on bile acid and lipid metabolism in morbid obesity. | https://doi.org/10.1016/j.jhep.2014.12.034 | Not relevant to our study |
| 2023 | In nonalcoholic fatty liver disease, a diet of freshwater fish vs. freshwater fish plus red meat reduced liver fat. | https://doi.org/10.7326/j22-0117 | Non-drug treatment research |
| 2020 | A metabolomics-based molecular pathway analysis of how the sodium-glucose co-transporter-2 inhibitor dapagliflozin may slow kidney function decline in patients with diabetes. | https://doi.org/10.1111/dom.14018 | Basic research |
| 2014 | Antioxidant supplements reduced oxidative stress and stabilized liver function tests but did not reduce inflammation in a randomized controlled trial in obese children and adolescents. | https://doi.org/10.3945/jn.113.185561 | Not relevant to our study |
| 2022 | Efficacy of omega-3-rich Camelina sativa on the metabolic and clinical markers in nonalcoholic fatty liver disease: a randomized, controlled trial. | https://doi.org/10.1097/meg.0000000000002297 | The study population was not diagnosed with diabetes |
| 2021 | Omega 3-rich Camelina sativa oil in the context of a weight loss program improves glucose homeostasis, inflammation and oxidative stress in patients with NAFLD: A randomised placebo-controlled clinical trial. | https://doi.org/10.1111/ijcp.14744 | The study population was not diagnosed with diabetes |
| 2014 | Effects of probiotic yogurt consumption on metabolic factors in individuals with nonalcoholic fatty liver disease. | https://doi.org/10.3168/jds.2014-8500 | The study population was not diagnosed with diabetes |
| 2009 | Treatment of non-alcoholic fatty liver disease with metformin versus lifestyle intervention in insulin-resistant adolescents. | https://doi.org/10.1111/j.1399-5448.2008.00450.x | The study population was children |
| 2018 | The relationship between 25-hydroxyvitamin D concentration and liver enzymes in overweight or obese adults: Cross-sectional and interventional outcomes. | https://doi.org/10.1016/j.jsbmb.2017.09.009 | Not relevant to our study |
| 2022 | Effects of naringenin supplementation on cardiovascular risk factors in overweight/obese patients with nonalcoholic fatty liver disease: a pilot double-blind, placebo-controlled, randomized clinical trial. | https://doi.org/10.1097/meg.0000000000002323 | The study population was not diagnosed with diabetes |
| 2021 | Effects of Cotadutide on Metabolic and Hepatic Parameters in Adults With Overweight or Obesity and Type 2 Diabetes: A 54-Week Randomized Phase 2b Study. | https://doi.org/10.2337/dc20-2151 | Only one article on the same kind of intervention |
| 2021 | Randomised clinical trial: Pemafibrate, a novel selective peroxisome proliferator-activated receptor α modulator (SPPARMα), versus placebo in patients with non-alcoholic fatty liver disease. | https://doi.org/10.1111/apt.16596 | The study population was not diagnosed with diabetes |
| 2021 | Does naringenin supplementation improve lipid profile, severity of hepatic steatosis and probability of liver fibrosis in overweight/obese patients with NAFLD? A randomised, double-blind, placebo-controlled, clinical trial. | https://doi.org/10.1111/ijcp.14852 | The study population was not diagnosed with diabetes |
| 2022 | Curcumin Offers No Additional Benefit to Lifestyle Intervention on Cardiometabolic Status in Patients with Non-Alcoholic Fatty Liver Disease. | https://doi.org/10.3390/nu14153224 | The study population was not diagnosed with diabetes |
| 2019 | Silymarin in non-cirrhotics with non-alcoholic steatohepatitis: A randomized, double-blind, placebo controlled trial. | https://doi.org/10.1371/journal.pone.0221683 | The study population was not diagnosed with diabetes |
| 2017 | Turmeric Supplementation Improves Serum Glucose Indices and Leptin Levels in Patients with Nonalcoholic Fatty Liver Diseases. | https://doi.org/10.1080/07315724.2016.1267597 | The study population was not diagnosed with diabetes |
| 2022 | Novel nutraceutical supplements with yeast β-glucan, prebiotics, minerals, and Silybum marianum (silymarin) ameliorate obesity-related metabolic and clinical parameters: A double-blind randomized trial. | https://doi.org/10.3389/fendo.2022.1089938 | Not relevant to our study |
| 2009 | A pilot study using simvastatin in the treatment of nonalcoholic steatohepatitis: A randomized placebo-controlled trial. | https://doi.org/10.1097/MCG.0b013e31819c392e | The study population was not diagnosed with diabetes |
| 2015 | Farnesoid X nuclear receptor ligand obeticholic acid for non-cirrhotic, non-alcoholic steatohepatitis (FLINT): a multicentre, randomised, placebo-controlled trial. | https://doi.org/10.1016/s0140-6736(14)61933-4 | The study population was not diagnosed with diabetes |
| 2021 | A Placebo-Controlled Trial of Subcutaneous Semaglutide in Nonalcoholic Steatohepatitis. | https://doi.org/10.1056/NEJMoa2028395 | The study population was not diagnosed with diabetes |
| 2020 | Volixibat in adults with non-alcoholic steatohepatitis: 24-week interim analysis from a randomized, phase II study. | https://doi.org/10.1016/j.jhep.2020.03.024 | The study population was not diagnosed with diabetes |
| 2021 | Alanine Aminotransferase and Gamma-Glutamyl Transpeptidase Predict Histologic Improvement in Pediatric Nonalcoholic Steatohepatitis. | https://doi.org/10.1002/hep.31317 | Not relevant to our study |
| 2021 | Continuous Positive Airway Pressure Does Not Improve Nonalcoholic Fatty Liver Disease in Patients with Obstructive Sleep Apnea. A Randomized Clinical Trial. | https://doi.org/10.1164/rccm.202005-1868OC | Not relevant to our study |
| 2022 | Effects of Shenxiang Suhe Pill on coronary heart disease complicated with nonalcoholic fatty liver disease: A case-control study. | https://doi.org/10.1097/md.0000000000031525 | Not relevant to our study |
| 2014 | Effect of a 6-month intervention with cooking oils containing a high concentration of monounsaturated fatty acids (olive and canola oils) compared with control oil in male Asian Indians with nonalcoholic fatty liver disease. | https://doi.org/10.1089/dia.2013.0178 | The study population was not diagnosed with diabetes |
| 2022 | Propolis supplementation in obese patients with non-alcoholic fatty liver disease: effects on glucose homeostasis, lipid profile, liver function, anthropometric indices and meta-inflammation. | https://doi.org/10.1039/d2fo01280d | The study population was not diagnosed with diabetes |
| 2013 | Docosahexaenoic acid for the treatment of fatty liver: randomised controlled trial in children. | https://doi.org/10.1016/j.numecd.2012.10.010 | The study population was children |
| 2019 | The Antioxidant Effects of Hydroxytyrosol and Vitamin E on Pediatric Nonalcoholic Fatty Liver Disease, in a Clinical Trial: A New Treatment? | https://doi.org/10.1089/ars.2018.7704 | The study population was children |
| 2011 | Docosahexaenoic acid supplementation decreases liver fat content in children with non-alcoholic fatty liver disease: double-blind randomised controlled clinical trial. | https://doi.org/10.1136/adc.2010.192401 | The study population was children |
| 2013 | The I148M variant of PNPLA3 reduces the response to docosahexaenoic acid in children with non-alcoholic fatty liver disease. | https://doi.org/10.1089/jmf.2013.0043 | The study population was children |
| 2008 | Metformin use in children with nonalcoholic fatty liver disease: an open-label, 24-month, observational pilot study. | https://doi.org/10.1016/j.clinthera.2008.06.012 | The study population was children |
| 2006 | Effect of vitamin E on aminotransferase levels and insulin resistance in children with non-alcoholic fatty liver disease. | https://doi.org/10.1111/j.1365-2036.2006.03161.x | The study population was children |
| 2008 | Lifestyle intervention and antioxidant therapy in children with nonalcoholic fatty liver disease: a randomized, controlled trial. | https://doi.org/10.1002/hep.22336 | The study population was children |
| 2016 | Omega-3 polyunsaturated fatty acids in treating non-alcoholic steatohepatitis: A randomized, double-blind, placebo-controlled trial. | https://doi.org/10.1016/j.clnu.2015.05.001 | The study population was not diagnosed with diabetes |
| 2022 | The effect of combined exercises on the plasma levels of retinol-binding protein 4 and its relationship with insulin resistance and hepatic fat content in postmenopausal women with nonalcoholic fatty liver disease. | https://doi.org/10.23736/s0022-4707.21.12233-9 | Not relevant to our study |
| 2022 | Lifestyle versus ezetimibe plus lifestyle in patients with biopsy-proven non-alcoholic steatohepatitis (LISTEN): A double-blind randomised placebo-controlled trial. | https://doi.org/10.1016/j.numecd.2022.01.024 | The study population was not diagnosed with diabetes |
| 2013 | Utility of magnetic resonance imaging versus histology for quantifying changes in liver fat in nonalcoholic fatty liver disease trials. | https://doi.org/10.1002/hep.26455 | Not relevant to our study |
| 2020 | Effect of lifestyle modification education based on health belief model in overweight/obese patients with non-alcoholic fatty liver disease: A parallel randomized controlled clinical trial. | https://doi.org/10.1016/j.clnesp.2020.04.004 | Not relevant to our study |
| 2019 | Factors influencing longitudinal changes of circulating liver enzyme concentrations in subjects randomized to placebo in four clinical trials. | https://doi.org/10.1152/ajpgi.00051.2018 | Not relevant to our study |
| 2016 | Efficacy and safety of ursodeoxycholic acid composite on fatigued patients with elevated liver function and/or fatty liver: a multi-centre, randomised, double-blinded, placebo-controlled trial. | https://doi.org/10.1111/ijcp.12790 | Not relevant to our study |
| 2018 | Omega-3 PUFA modulate lipogenesis, ER stress, and mitochondrial dysfunction markers in NASH - Proteomic and lipidomic insight. | https://doi.org/10.1016/j.clnu.2017.08.031 | Basic research |
| 2019 | A population analysis of the DGAT1 inhibitor GSK3008356 and its effect on endogenous and meal-induced triglyceride turnover in healthy subjects. | https://doi.org/10.1111/fcp.12455 | Not relevant to our study |
| 2019 | N-ACETYLCYSTEINE AND/OR URSODEOXYCHOLIC ACID ASSOCIATED WITH METFORMIN IN NON-ALCOHOLIC STEATOHEPATITIS: AN OPEN-LABEL MULTICENTER RANDOMIZED CONTROLLED TRIAL. | https://doi.org/10.1590/s0004-2803.201900000-36 | The study population was not diagnosed with diabetes |
| 2010 | Efficacy of insulin-sensitizing agents in nonalcoholic fatty liver disease. | https://doi.org/10.1097/MEG.0b013e32832e2baf | The study population was not diagnosed with diabetes |
| 2022 | [Multifaceted Clinical Research on Obesity-related Disease Prevention Focusing on the DsbA-L Gene]. | https://doi.org/10.1248/yakushi.22-00128 | Basic research |
| 2017 | Inhibition of IKKɛ and TBK1 Improves Glucose Control in a Subset of Patients with Type 2 Diabetes. | https://doi.org/10.1016/j.cmet.2017.06.006 | Not relevant to our study |
| 2018 | Effects of free omega-3 carboxylic acids and fenofibrate on liver fat content in patients with hypertriglyceridemia and non-alcoholic fatty liver disease: A double-blind, randomized, placebo-controlled study. | https://doi.org/10.1016/j.jacl.2018.08.003 | The study population was not diagnosed with diabetes |
| 2019 | Severe nonalcoholic steatohepatitis and type 2 diabetes: liver histology after weight loss therapy in a randomized clinical trial. | https://doi.org/10.1080/03007995.2018.1547696 | Weight loss therapy |
| 2017 | Dimethylguanidino valeric acid is a marker of liver fat and predicts diabetes. | https://doi.org/10.1172/jci95995 | Not relevant to our study |
| 2016 | Strong and persistent effect on liver fat with a Paleolithic diet during a two-year intervention. | https://doi.org/10.1038/ijo.2016.4 | Non-drug treatment research |
| 2022 | Changes in Biomarkers of Non-Alcoholic Fatty Liver Disease (NAFLD) upon Access to Avocados in Hispanic/Latino Adults: Secondary Data Analysis of a Cluster Randomized Controlled Trial. | https://doi.org/10.3390/nu14132744 | The study population was not diagnosed with diabetes |
| 2015 | A double-blind, placebo-controlled randomized trial to evaluate the efficacy of docosahexaenoic acid supplementation on hepatic fat and associated cardiovascular risk factors in overweight children with nonalcoholic fatty liver disease. | https://doi.org/10.1016/j.numecd.2015.04.003 | The study population was children |
| 2012 | [Relation of non-alcoholic hepatic steatosis to early carotid atherosclerosis in diet-controlled impaired glucose tolerance subjects]. |  | Not relevant to our study |
| 2018 | A randomised, double-blind, placebo-controlled phase 1 study of the safety, tolerability and pharmacodynamics of volixibat in overweight and obese but otherwise healthy adults: implications for treatment of non-alcoholic steatohepatitis. | https://doi.org/10.1186/s40360-018-0200-y | The study population was not diagnosed with diabetes |
| 2003 | N-acetylcysteine in the treatment of non-alcoholic steatohepatitis. | https://doi.org/10.1046/j.1440-1746.2003.03156.x | The study population was not diagnosed with diabetes |
| 2021 | Effect of recombinant human growth hormone on liver fat content in young adults with nonalcoholic fatty liver disease. | https://doi.org/10.1111/cen.14344 | The study population was not diagnosed with diabetes |
| 2012 | Investigation of the effects of Chlorella vulgaris supplementation in patients with non-alcoholic fatty liver disease: a randomized clinical trial. | https://doi.org/10.5754/hge10860 | The study population was not diagnosed with diabetes |
| 2018 | Efficacy of artichoke leaf extract in non-alcoholic fatty liver disease: A pilot double-blind randomized controlled trial. | https://doi.org/10.1002/ptr.6073 | The study population was not diagnosed with diabetes |
| 2016 | Curcumin Lowers Serum Lipids and Uric Acid in Subjects With Nonalcoholic Fatty Liver Disease: A Randomized Controlled Trial. | https://doi.org/10.1097/fjc.0000000000000406 | The study population was not diagnosed with diabetes |
| 2017 | Efficacy and Safety of Phytosomal Curcumin in Non-Alcoholic Fatty Liver Disease: A Randomized Controlled Trial. | https://doi.org/10.1055/s-0043-100019 | The study population was not diagnosed with diabetes |
| 2019 | Curcuminoids plus piperine improve nonalcoholic fatty liver disease: A clinical trial. | https://doi.org/10.1002/jcb.28877 | The study population was not diagnosed with diabetes |
| 2016 | A randomised controlled trial of a Mediterranean Dietary Intervention for Adults with Non Alcoholic Fatty Liver Disease (MEDINA): study protocol. | https://doi.org/10.1186/s12876-016-0426-3 | Non-drug treatment research |
| 2016 | An open-label randomized control study to compare the efficacy of vitamin e versus ursodeoxycholic acid in nondiabetic and noncirrhotic Indian NAFLD patients. | https://doi.org/10.4103/1319-3767.182451 | The study population was not diagnosed with diabetes |
| 2019 | Effect of Fish Oil Supplementation on Hepatic and Visceral Fat in Overweight Men: A Randomized Controlled Trial. | https://doi.org/10.3390/nu11020475 | The study population was not diagnosed with diabetes |
| 2020 | Intrahepatic Fat and Postprandial Glycemia Increase After Consumption of a Diet Enriched in Saturated Fat Compared With Free Sugars. | https://doi.org/10.2337/dc19-2331 | Not relevant to our study |
| 2020 | Cilofexor, a Nonsteroidal FXR Agonist, in Patients With Noncirrhotic NASH: A Phase 2 Randomized Controlled Trial. | https://doi.org/10.1002/hep.31205 | The study population was not diagnosed with diabetes |
| 2015 | Effect of weight loss on magnetic resonance imaging estimation of liver fat and volume in patients with nonalcoholic steatohepatitis. | https://doi.org/10.1016/j.cgh.2014.08.039 | Weight loss therapy |
| 2008 | Clinical correlates of histopathology in pediatric nonalcoholic steatohepatitis. | https://doi.org/10.1053/j.gastro.2008.08.050 | The study population was children |
| 2012 | [Clinical study of Qinggan Huatan Huoxue Recipe on the treatment of non-alcoholic steatohepatitis]. |  | Study on the treatment of traditional Chinese medicine |
| 2016 | Effects of obeticholic acid on lipoprotein metabolism in healthy volunteers. | https://doi.org/10.1111/dom.12681 | Not relevant to our study |
| 2022 | A nutrigenetic tool for precision dietary management of non-alcoholic fatty liver disease deeming insulin resistance markers. | https://doi.org/10.23736/s0031-0808.22.04590-6 | Not relevant to our study |
| 2022 | Hepato-Protective Effects of Delta-Tocotrienol and Alpha-Tocopherol in Patients with Non-Alcoholic Fatty Liver Disease: Regulation of Circulating MicroRNA Expression. | https://doi.org/10.3390/ijms24010079 | Basic research |
| 2018 | Effects of Delta-tocotrienol Supplementation on Liver Enzymes, Inflammation, Oxidative stress and Hepatic Steatosis in Patients with Nonalcoholic Fatty Liver Disease. | https://doi.org/10.5152/tjg.2018.17297 | Not relevant to our study |
| 2022 | Comparison of delta-tocotrienol and alpha-tocopherol effects on hepatic steatosis and inflammatory biomarkers in patients with non-alcoholic fatty liver disease: A randomized double-blind active-controlled trial. | https://doi.org/10.1016/j.ctim.2022.102866 | The study population was not diagnosed with diabetes |
| 2020 | Delta-tocotrienol supplementation improves biochemical markers of hepatocellular injury and steatosis in patients with nonalcoholic fatty liver disease: A randomized, placebo-controlled trial. | https://doi.org/10.1016/j.ctim.2020.102494 | The study population was not diagnosed with diabetes |
| 2018 | The PNPLA3 I148M variant is associated with transaminase elevations in type 2 diabetes patients treated with basal insulin peglispro. | https://doi.org/10.1038/tpj.2017.45 | Basic research |
| 2019 | A Mediterranean Diet Rich in Extra-Virgin Olive Oil Is Associated with a Reduced Prevalence of Nonalcoholic Fatty Liver Disease in Older Individuals at High Cardiovascular Risk. | https://doi.org/10.1093/jn/nxz147 | The study population was not diagnosed with diabetes |
| 2019 | CONTROL: A randomized phase 2 study of obeticholic acid and atorvastatin on lipoproteins in nonalcoholic steatohepatitis patients. | https://doi.org/10.1111/liv.14209 | The study population was not diagnosed with diabetes |
| 2021 | Telemedicine as a tool for dietary intervention in NAFLD-HIV patients during the COVID-19 lockdown: A randomized controlled trial. | https://doi.org/10.1016/j.clnesp.2021.03.031 | Not relevant to our study |
| 2018 | Noggin levels in nonalcoholic fatty liver disease: the effect of vitamin E treatment. | https://doi.org/10.1007/s42000-018-0083-8 | The study population was not diagnosed with diabetes |
| 2017 | Effects of combined low-dose spironolactone plus vitamin E vs vitamin E monotherapy on insulin resistance, non-invasive indices of steatosis and fibrosis, and adipokine levels in non-alcoholic fatty liver disease: a randomized controlled trial. | https://doi.org/10.1111/dom.12989 | The study population was not diagnosed with diabetes |
| 2011 | Effect of spironolactone and vitamin E on serum metabolic parameters and insulin resistance in patients with nonalcoholic fatty liver disease. | https://doi.org/10.1177/1470320311402110 | The study population was not diagnosed with diabetes |
| 2019 | Effect of pioglitazone on bone mineral density in patients with nonalcoholic steatohepatitis: A 36-month clinical trial. | https://doi.org/10.1111/1753-0407.12833 | The primary outcome measure was not consistent |
| 2018 | No effect of resveratrol on VLDL-TG kinetics and insulin sensitivity in obese men with nonalcoholic fatty liver disease. | https://doi.org/10.1111/dom.13409 | The study population was not diagnosed with diabetes |
| 2020 | The effect of saffron supplementation on some inflammatory and oxidative markers, leptin, adiponectin, and body composition in patients with nonalcoholic fatty liver disease: A double-blind randomized clinical trial. | https://doi.org/10.1002/ptr.6791 | The study population was not diagnosed with diabetes |
| 2008 | The effects of 8 months of metformin on circulating GGT and ALT levels in obese women with polycystic ovarian syndrome. | https://doi.org/10.1111/j.1742-1241.2008.01825.x | Not relevant to our study |
| 2010 | Randomized controlled trial testing the effects of weight loss on nonalcoholic steatohepatitis. | https://doi.org/10.1002/hep.23276 | Weight loss therapy |
| 2018 | Ad Libitum Mediterranean and Low-Fat Diets Both Significantly Reduce Hepatic Steatosis: A Randomized Controlled Trial. | https://doi.org/10.1002/hep.30076 | Non-drug treatment research |
| 2015 | High-risk coronary plaque at coronary CT angiography is associated with nonalcoholic fatty liver disease, independent of coronary plaque and stenosis burden: results from the ROMICAT II trial. | https://doi.org/10.1148/radiol.14140933 | Not relevant to our study |
| 2013 | Exercise training improves cutaneous microvascular function in nonalcoholic fatty liver disease. | https://doi.org/10.1152/ajpendo.00055.2013 | Non-drug treatment research |
| 2014 | Exercise training reverses endothelial dysfunction in nonalcoholic fatty liver disease. | https://doi.org/10.1152/ajpheart.00306.2014 | Non-drug treatment research |
| 2022 | A clinical and laboratory-based nomogram for predicting nonalcoholic fatty liver disease in non-diabetic adults: a cross-sectional study. | https://doi.org/10.21037/apm-21-2988 | A cross-sectional study. |
| 2015 | Fish Oil Supplements Lower Serum Lipids and Glucose in Correlation with a Reduction in Plasma Fibroblast Growth Factor 21 and Prostaglandin E2 in Nonalcoholic Fatty Liver Disease Associated with Hyperlipidemia: A Randomized Clinical Trial. | https://doi.org/10.1371/journal.pone.0133496 | The study population was not diagnosed with diabetes |
| 2020 | Exercise training modulates the gut microbiota profile and impairs inflammatory signaling pathways in obese children. | https://doi.org/10.1038/s12276-020-0459-0 | Basic research |
| 2022 | Noninvasive predictors of clinically significant portal hypertension in NASH cirrhosis: Validation of ANTICIPATE models and development of a lab-based model. | https://doi.org/10.1002/hep4.2091 | Anticipate models |
| 2011 | Reversal of muscle insulin resistance with exercise reduces postprandial hepatic de novo lipogenesis in insulin resistant individuals. | https://doi.org/10.1073/pnas.1110105108 | Not relevant to our study |
| 2017 | Differential Impact of Weight Loss on Nonalcoholic Fatty Liver Resolution in a North American Cohort with Obesity. | https://doi.org/10.1002/oby.21890 | Weight loss therapy |
| 2022 | LLF580, an FGF21 Analog, Reduces Triglycerides and Hepatic Fat in Obese Adults With Modest Hypertriglyceridemia. | https://doi.org/10.1210/clinem/dgab624 | Not relevant to our study |
| 2022 | Alterations of liver enzymes and lipid profile in response to exhaustive eccentric exercise: vitamin D supplementation trial in overweight females with non-alcoholic fatty liver disease. | https://doi.org/10.1186/s12876-022-02457-w | The study population was not diagnosed with diabetes |
| 2019 | Oral α-lipoic acid supplementation in patients with non-alcoholic fatty liver disease: effects on adipokines and liver histology features. | https://doi.org/10.1039/c9fo00449a | The study population was not diagnosed with diabetes |
| 2016 | Treatment of Non-alcoholic Fatty Liver Disease with Curcumin: A Randomized Placebo-controlled Trial. | https://doi.org/10.1002/ptr.5659 | The study population was not diagnosed with diabetes |
| 2022 | The effect of nigella sativa supplementation on cardiometabolic outcomes in patients with non-alcoholic fatty liver: A randomized double-blind, placebo-controlled trial. | https://doi.org/10.1016/j.ctcp.2022.101598 | The study population was not diagnosed with diabetes |
| 2014 | Lack of efficacy of an inhibitor of PDE4 in phase 1 and 2 trials of patients with nonalcoholic steatohepatitis. | https://doi.org/10.1016/j.cgh.2014.01.040 | The study population was not diagnosed with diabetes |
| 2010 | Long-term efficacy of rosiglitazone in nonalcoholic steatohepatitis: results of the fatty liver improvement by rosiglitazone therapy (FLIRT 2) extension trial. | https://doi.org/10.1002/hep.23270 | The study population was not diagnosed with diabetes |
| 2021 | Aramchol in patients with nonalcoholic steatohepatitis: a randomized, double-blind, placebo-controlled phase 2b trial. | https://doi.org/10.1038/s41591-021-01495-3 | The study population was not diagnosed with diabetes |
| 2011 | A randomized controlled trial of high-dose ursodesoxycholic acid for nonalcoholic steatohepatitis. | https://doi.org/10.1016/j.jhep.2010.08.030 | The study population was not diagnosed with diabetes |
| 2008 | Rosiglitazone for nonalcoholic steatohepatitis: one-year results of the randomized placebo-controlled Fatty Liver Improvement with Rosiglitazone Therapy (FLIRT) Trial. | https://doi.org/10.1053/j.gastro.2008.03.078 | The study population was not diagnosed with diabetes |
| 2016 | Elafibranor, an Agonist of the Peroxisome Proliferator-Activated Receptor-α and -δ, Induces Resolution of Nonalcoholic Steatohepatitis Without Fibrosis Worsening. | https://doi.org/10.1053/j.gastro.2016.01.038 | The study population was not diagnosed with diabetes |
| 2023 | Hepatic and renal improvements with FXR agonist vonafexor in individuals with suspected fibrotic NASH. | https://doi.org/10.1016/j.jhep.2022.10.023 | The study population was not diagnosed with diabetes |
| 2020 | Cenicriviroc Treatment for Adults With Nonalcoholic Steatohepatitis and Fibrosis: Final Analysis of the Phase 2b CENTAUR Study. | https://doi.org/10.1002/hep.31108 | The study population was not diagnosed with diabetes |
| 2019 | REGENERATE: Design of a pivotal, randomised, phase 3 study evaluating the safety and efficacy of obeticholic acid in patients with fibrosis due to nonalcoholic steatohepatitis. | https://doi.org/10.1016/j.cct.2019.06.017 | The study population was not diagnosed with diabetes |
| 2012 | A phase 2, randomized, double-blind, placebo-controlled study of GS-9450 in subjects with nonalcoholic steatohepatitis. | https://doi.org/10.1002/hep.24747 | The study population was not diagnosed with diabetes |
| 2016 | The effects of DASH diet on weight loss and metabolic status in adults with non-alcoholic fatty liver disease: a randomized clinical trial. | https://doi.org/10.1111/liv.12990 | Non-drug treatment research |
| 2023 | The rationale and study design of two phase II trials examining the effects of BI 685,509, a soluble guanylyl cyclase activator, on clinically significant portal hypertension in patients with compensated cirrhosis. | https://doi.org/10.1186/s13063-023-07291-3 | Not relevant to our study |
| 2019 | Olive oil lessened fatty liver severity independent of cardiometabolic correction in patients with non-alcoholic fatty liver disease: A randomized clinical trial. | https://doi.org/10.1016/j.nut.2018.02.021 | The study population was not diagnosed with diabetes |
| 2020 | Flaxseed oil in the context of a weight loss programme ameliorates fatty liver grade in patients with non-alcoholic fatty liver disease: a randomised double-blind controlled trial. | https://doi.org/10.1017/s0007114520000318 | The study population was not diagnosed with diabetes |
| 2016 | Randomized clinical trial: benefits of aerobic physical activity for 24 weeks in postmenopausal women with nonalcoholic fatty liver disease. | https://doi.org/10.1097/gme.0000000000000647 | Non-drug treatment research |
| 2020 | Calorie-Restricted Mediterranean and Low-Fat Diets Affect Fatty Acid Status in Individuals with Nonalcoholic Fatty Liver Disease. | https://doi.org/10.3390/nu13010015 | Non-drug treatment research |
| 2017 | Effects of lifestyle intervention on soluble CD163, a macrophage activation marker, in patients with non-alcoholic fatty liver disease. | https://doi.org/10.1080/00365513.2017.1346823 | Basic research |
| 2011 | Decrease of aminotransferase levels in obese women is related to body weight reduction, irrespective of type of diet. |  | Not relevant to our study |
| 2013 | [Non-invasive techniques in diagnosis of steatosis and fibrosis in nonalcoholic fatty liver disease]. |  | Not relevant to our study |
| 2014 | [New metabolic index in the diagnosis of insulin resistance in patients with nonalcoholic fatty liver disease]. |  | Not relevant to our study |
| 2022 | Circulating fatty acids from high-throughput metabolomics platforms as potential biomarkers of dietary fatty acids. | https://doi.org/10.1016/j.clnu.2022.10.005 | Not relevant to our study |
| 2019 | Overeating Saturated Fat Promotes Fatty Liver and Ceramides Compared With Polyunsaturated Fat: A Randomized Trial. | https://doi.org/10.1210/jc.2019-00160 | Not relevant to our study |
| 2022 | Effects of licorice root supplementation on liver enzymes, hepatic steatosis, metabolic and oxidative stress parameters in women with nonalcoholic fatty liver disease: A randomized double-blind clinical trial. | https://doi.org/10.1002/ptr.7543 | The study population was not diagnosed with diabetes |
| 2023 | A prolonged fast improves overnight substrate oxidation without modulating hepatic glycogen in adults with and without nonalcoholic fatty liver: A randomized crossover trial. | https://doi.org/10.1002/oby.23676 | Not relevant to our study |
| 2007 | Serum alanine aminotransferase levels decrease further with carbohydrate than fat restriction in insulin-resistant adults. | https://doi.org/10.2337/dc06-2169 | Not relevant to our study |
| 2013 | The Mediterranean diet improves hepatic steatosis and insulin sensitivity in individuals with non-alcoholic fatty liver disease. | https://doi.org/10.1016/j.jhep.2013.02.012 | Non-drug treatment research |
| 2019 | The effects of curcumin supplementation on liver enzymes, lipid profile, glucose homeostasis, and hepatic steatosis and fibrosis in patients with non-alcoholic fatty liver disease. | https://doi.org/10.1038/s41430-018-0382-9 | The study population was not diagnosed with diabetes |
| 2019 | Curcumin and inflammation in non-alcoholic fatty liver disease: a randomized, placebo controlled clinical trial. | https://doi.org/10.1186/s12876-019-1055-4 | The study population was not diagnosed with diabetes |
| 2020 | Effects of curcuminoids on inflammatory status in patients with non-alcoholic fatty liver disease: A randomized controlled trial. | https://doi.org/10.1016/j.ctim.2020.102322 | The study population was not diagnosed with diabetes |
| 2023 | The antianginal ranolazine fails to improve glycaemia in obese liver-specific pyruvate dehydrogenase deficient male mice. | https://doi.org/10.1111/bcpt.13906 | Animal experiment |
| 2021 | Randomised clinical trial: A phase 2 double-blind study of namodenoson in non-alcoholic fatty liver disease and steatohepatitis. | https://doi.org/10.1111/apt.16664 | The study population was not diagnosed with diabetes |
| 2014 | The fatty acid-bile acid conjugate Aramchol reduces liver fat content in patients with nonalcoholic fatty liver disease. | https://doi.org/10.1016/j.cgh.2014.04.038 | The study population was not diagnosed with diabetes |
| 2005 | Ciprofloxacin suppresses bacterial overgrowth, increases fasting insulin but does not correct low acylated ghrelin concentration in non-alcoholic steatohepatitis. | https://doi.org/10.1111/j.1365-2036.2005.02562.x | Not relevant to our study |
| 2020 | Effects of obesity, metabolic syndrome, and non-alcoholic or alcoholic elevated liver enzymes on incidence of diabetes following lifestyle intervention: A subanalysis of the J-DOIT1. | https://doi.org/10.1002/1348-9585.12109 | Not relevant to our study |
| 2013 | Green tea with high-density catechins improves liver function and fat infiltration in non-alcoholic fatty liver disease (NAFLD) patients: a double-blind placebo-controlled study. | https://doi.org/10.3892/ijmm.2013.1503 | The study population was not diagnosed with diabetes |
| 2022 | Wheat germ improves hepatic steatosis, hepatic enzymes, and metabolic and inflammatory parameters in patients with nonalcoholic fatty liver disease: A randomized, placebo-controlled, double-blind clinical trial. | https://doi.org/10.1002/ptr.7553 | The study population was not diagnosed with diabetes |
| 2011 | Exenatide decreases hepatic fibroblast growth factor 21 resistance in non-alcoholic fatty liver disease in a mouse model of obesity and in a randomised controlled trial. | https://doi.org/10.1007/s00125-011-2317-z | Animal experiment |
| 2011 | Paraoxonase-1 activity, malondialdehyde and glutathione peroxidase in non-alcoholic fatty liver disease and the effect of atorvastatin. | https://doi.org/10.1016/j.ajg.2011.04.008 | The primary outcome measure was not consistent |
| 2011 | Association of nonalcoholic fatty liver disease with metabolic syndrome in Indian population. | https://doi.org/10.1016/j.dsx.2012.02.015 | Not relevant to our study |
| 2013 | [Decrease of liver fat content by aerobic exercise or metformin therapy in overweight or obese women]. |  | The study population was not diagnosed with diabetes |
| 2021 | Effect of l-carnitine supplementation on children and adolescents with nonalcoholic fatty liver disease (NAFLD): a randomized, triple-blind, placebo-controlled clinical trial. | https://doi.org/10.1515/jpem-2020-0642 | The study population was children |
| 2021 | Effects of garlic powder supplementation on metabolic syndrome components, insulin resistance, fatty liver index, and appetite in subjects with metabolic syndrome: A randomized clinical trial. | https://doi.org/10.1002/ptr.7146 | Not relevant to our study |
| 2020 | Effect of garlic powder supplementation on hepatic steatosis, liver enzymes and lipid profile in patients with non-alcoholic fatty liver disease: a double-blind randomised controlled clinical trial. | https://doi.org/10.1017/s0007114520001403 | The study population was not diagnosed with diabetes |
| 2020 | Effects of garlic powder supplementation on insulin resistance, oxidative stress, and body composition in patients with non-alcoholic fatty liver disease: A randomized controlled clinical trial. | https://doi.org/10.1016/j.ctim.2020.102428 | The study population was not diagnosed with diabetes |
| 2022 | Effect of Cornus mas L. fruit extract on lipid accumulation product and cardiovascular indices in patients with non-alcoholic fatty liver disease: A double-blind randomized controlled trial. | https://doi.org/10.1016/j.clnesp.2021.11.023 | The study population was not diagnosed with diabetes |
| 2021 | Effect of l-carnitine supplementation on liver fat content and cardiometabolic indices in overweight/obese women with polycystic ovary syndrome: A randomized controlled trial. | https://doi.org/10.1016/j.clnesp.2021.08.005 | Not relevant to our study |
| 2019 | The effect of total anthocyanin-base standardized (Cornus mas L.) fruit extract on liver function, tumor necrosis factor α, malondealdehyde, and adiponectin in patients with non-alcoholic fatty liver: a study protocol for a double-blind randomized clinical trial. | https://doi.org/10.1186/s12937-019-0465-z | The study population was not diagnosed with diabetes |
| 2021 | The effect of (Cornus mas L.) fruit extract on liver function among patients with nonalcoholic fatty liver: A double-blind randomized clinical trial. | https://doi.org/10.1002/ptr.7199 | The study population was not diagnosed with diabetes |
| 2003 | A randomized double-blind study of the short-time treatment of obese patients with nonalcoholic fatty liver disease with ursodeoxycholic acid. | https://doi.org/10.1590/s0100-879x2003000600007 | The study population was not diagnosed with diabetes |
| 2019 | Pegbelfermin (BMS-986036), a PEGylated fibroblast growth factor 21 analogue, in patients with non-alcoholic steatohepatitis: a randomised, double-blind, placebo-controlled, phase 2a trial. | https://doi.org/10.1016/s0140-6736(18)31785-9 | The study population was not diagnosed with diabetes |
| 2014 | No significant effects of ethyl-eicosapentanoic acid on histologic features of nonalcoholic steatohepatitis in a phase 2 trial. | https://doi.org/10.1053/j.gastro.2014.04.046 | The study population was not diagnosed with diabetes |
| 2010 | Pioglitazone, vitamin E, or placebo for nonalcoholic steatohepatitis. | https://doi.org/10.1056/NEJMoa0907929 | The study population was not diagnosed with diabetes |
| 2023 | Tropifexor for nonalcoholic steatohepatitis: an adaptive, randomized, placebo-controlled phase 2a/b trial. | https://doi.org/10.1038/s41591-022-02200-8 | The study population was not diagnosed with diabetes |
| 2004 | A pilot study of vitamin E versus vitamin E and pioglitazone for the treatment of nonalcoholic steatohepatitis. | https://doi.org/10.1016/s1542-3565(04)00457-4 | The study population was not diagnosed with diabetes |
| 2020 | Monounsaturated fat rapidly induces hepatic gluconeogenesis and whole-body insulin resistance. | https://doi.org/10.1172/jci.insight.134520 | Not relevant to our study |
| 2022 | [Modification of dietary patterns in patients with non-alcoholic steatohepatitis]. | https://doi.org/10.26442/00403660.2022.08.201773 | Non-drug treatment research |
| 2022 | [Efficacy of newly developed food for special dietary use in the diet of patients with non-alcoholic steatohepatitis]. | https://doi.org/10.33029/0042-8833-2022-91-2-31-42 | The study population was not diagnosed with diabetes |
| 2016 | Endocannabinoid receptor blockade increases hepatocyte growth factor and reduces insulin levels in obese women with polycystic ovary syndrome. | https://doi.org/10.1111/cen.13120 | Not relevant to our study |
| 2016 | Do pancrelipase delayed-release capsules have a protective role against nonalcoholic fatty liver disease after pancreatoduodenectomy in patients with pancreatic cancer? A randomized controlled trial. | https://doi.org/10.1002/jhbp.318 | Not relevant to our study |
| 2018 | Empagliflozin is associated with improvements in liver enzymes potentially consistent with reductions in liver fat: results from randomised trials including the EMPA-REG OUTCOME® trial. | https://doi.org/10.1007/s00125-018-4702-3 | Not relevant to our study |
| 2016 | Circulating adiponectin levels in type 2 diabetes mellitus patients with or without non-alcoholic fatty liver disease: Results of a small, open-label, randomized controlled intervention trial in a subgroup receiving short-term exenatide. | https://doi.org/10.1016/j.diabres.2015.12.003 | The study population was not diagnosed with NAFLD |
| 2023 | A phase 2a, randomized, double-blind, placebo-controlled, three-arm, parallel-group study to assess the efficacy, safety, tolerability and pharmacodynamics of PF-06835919 in patients with non-alcoholic fatty liver disease and type 2 diabetes. | https://doi.org/10.1111/dom.14946 | Only one article on the same kind of intervention |
| 2018 | Combined effects of synbiotic and sitagliptin versus sitagliptin alone in patients with nonalcoholic fatty liver disease. | https://doi.org/10.3350/cmh.2018.0006 | The study population was not diagnosed with diabetes |
| 2017 | Chronic Intranasal Insulin Does Not Affect Hepatic Lipids but Lowers Circulating BCAAs in Healthy Male Subjects. | https://doi.org/10.1210/jc.2016-3623 | Not relevant to our study |
| 2022 | Clinical Intervention to Reduce Dietary Sugar Does Not Affect Liver Fat in Latino Youth, Regardless of PNPLA3 Genotype: A Randomized Controlled Trial. | https://doi.org/10.1093/jn/nxac046 | Not relevant to our study |
| 2011 | Differences in the disposition of silymarin between patients with nonalcoholic fatty liver disease and chronic hepatitis C. | https://doi.org/10.1124/dmd.111.040212 | Not relevant to our study |
| 2019 | Similar Weight Loss Induces Greater Improvements in Insulin Sensitivity and Liver Function among Individuals with NAFLD Compared to Individuals without NAFLD. | https://doi.org/10.3390/nu11030544 | Not relevant to our study |
| 2018 | A 12-wk whole-grain wheat intervention protects against hepatic fat: the Graandioos study, a randomized trial in overweight subjects. | https://doi.org/10.1093/ajcn/nqy204 | Not relevant to our study |
| 2018 | [Comparison of a commercially available, formula-based nutritional therapy enriched with oats fiber with a non-formula isocaloric therapy to treat non-alcoholic fatty liver disease (NAFLD) - a randomized, controlled intervention trial]. | https://doi.org/10.1055/a-0668-2891 | Non-drug treatment research |
| 2016 | In Children With Nonalcoholic Fatty Liver Disease, Cysteamine Bitartrate Delayed Release Improves Liver Enzymes but Does Not Reduce Disease Activity Scores. | https://doi.org/10.1053/j.gastro.2016.08.027 | The study population was children |
| 2019 | Effect of a Low Free Sugar Diet vs Usual Diet on Nonalcoholic Fatty Liver Disease in Adolescent Boys: A Randomized Clinical Trial. | https://doi.org/10.1001/jama.2018.20579 | Non-drug treatment research |
| 2020 | Synbiotics Alter Fecal Microbiomes, But Not Liver Fat or Fibrosis, in a Randomized Trial of Patients With Nonalcoholic Fatty Liver Disease. | https://doi.org/10.1053/j.gastro.2020.01.031 | The study population was not diagnosed with diabetes |
| 2018 | Design and rationale of the INSYTE study: A randomised, placebo controlled study to test the efficacy of a synbiotic on liver fat, disease biomarkers and intestinal microbiota in non-alcoholic fatty liver disease. | https://doi.org/10.1016/j.cct.2018.05.010 | Not relevant to our study |
| 2014 | Design and rationale of the WELCOME trial: A randomised, placebo controlled study to test the efficacy of purified long chainomega-3 fatty acid treatment in non-alcoholic fatty liver disease [corrected]. | https://doi.org/10.1016/j.cct.2014.02.002 | The study population was not diagnosed with diabetes |
| 2014 | Effects of purified eicosapentaenoic and docosahexaenoic acids in nonalcoholic fatty liver disease: results from the Welcome* study. | https://doi.org/10.1002/hep.27289 | The study population was not diagnosed with diabetes |
| 2015 | Treating liver fat and serum triglyceride levels in NAFLD, effects of PNPLA3 and TM6SF2 genotypes: Results from the WELCOME trial. | https://doi.org/10.1016/j.jhep.2015.07.036 | Basic research |
| 2014 | [An analysis of the efficacy of low-calorie and isocaloric diets in obese patients with nonalcoholic steatohepatitis]. |  | Non-drug treatment research |
| 2016 | Effects of Multistrain Probiotic Supplementation on Glycemic and Inflammatory Indices in Patients with Nonalcoholic Fatty Liver Disease: A Double-Blind Randomized Clinical Trial. | https://doi.org/10.1080/07315724.2015.1031355 | The study population was not diagnosed with diabetes |
| 2021 | Prevalence, Diagnosis, and Treatment with 3 Different Statins of Non-alcoholic Fatty Liver Disease/Non-alcoholic Steatohepatitis in Military Personnel. Do Genetics Play a Role? | https://doi.org/10.2174/1570161118666201015152921 | The study population was not diagnosed with diabetes |
| 2009 | Diet and exercise interventions reduce intrahepatic fat content and improve insulin sensitivity in obese older adults. | https://doi.org/10.1038/oby.2009.126 | Non-drug treatment research |
| 2020 | Spontaneous Fluctuations in Liver Biochemistries in Patients with Compensated NASH Cirrhosis: Implications for Drug Hepatotoxicity Monitoring. | https://doi.org/10.1007/s40264-019-00896-1 | Not relevant to our study |
| 2014 | Benefits of exenatide on obesity and non-alcoholic fatty liver disease with elevated liver enzymes in patients with type 2 diabetes. | https://doi.org/10.1002/dmrr.2561 | Only one article on the same kind of intervention |
| 2012 | Relation between augmentation index and adiponectin during one-year metformin treatment for nonalcoholic steatohepatosis: effects beyond glucose lowering? | https://doi.org/10.1186/1475-2840-11-61 | The study population was not diagnosed with diabetes |
| 2014 | Does vitamin D improve liver enzymes, oxidative stress, and inflammatory biomarkers in adults with non-alcoholic fatty liver disease? A randomized clinical trial. | https://doi.org/10.1007/s12020-014-0336-5 | The study population was not diagnosed with diabetes |
| 2016 | Women may respond different from men to vitamin D supplementation regarding cardiometabolic biomarkers. | https://doi.org/10.1177/1535370216629009 | Not relevant to our study |
| 2023 | Efficacy of curcumin plus piperine co-supplementation in moderate-to-high hepatic steatosis: A double-blind, randomized, placebo-controlled clinical trial. | https://doi.org/10.1002/ptr.7764 | The study population was not diagnosed with diabetes |
| 2023 | Changes in abdominal adipose tissue depots assessed by MRI correlate with hepatic histologic improvement in non-alcoholic steatohepatitis. | https://doi.org/10.1016/j.jhep.2022.10.027 | Not relevant to our study |
| 2016 | Nutritional Status Prior to Laparoscopic Sleeve Gastrectomy Surgery. | https://doi.org/10.1007/s11695-016-2064-9 | Not relevant to our study |
| 2018 | Probiotics administration following sleeve gastrectomy surgery: a randomized double-blind trial. | https://doi.org/10.1038/ijo.2017.210 | Not relevant to our study |
| 2018 | Luseogliflozin improves liver fat deposition compared to metformin in type 2 diabetes patients with non-alcoholic fatty liver disease: A prospective randomized controlled pilot study. | https://doi.org/10.1111/dom.13061 | Only one article on the same kind of intervention |
| 2018 | The Effects of Extra Virgin Olive Oil on Alanine Aminotransferase, Aspartate Aminotransferase, and Ultrasonographic Indices of Hepatic Steatosis in Nonalcoholic Fatty Liver Disease Patients Undergoing Low Calorie Diet. | https://doi.org/10.1155/2018/1053710 | Non-drug treatment research |
| 2019 | Randomised clinical trial: emricasan versus placebo significantly decreases ALT and caspase 3/7 activation in subjects with non-alcoholic fatty liver disease. | https://doi.org/10.1111/apt.15030 | The study population was not diagnosed with diabetes |
| 2021 | Fucoidan and Fucoxanthin Attenuate Hepatic Steatosis and Inflammation of NAFLD through Modulation of Leptin/Adiponectin Axis. | https://doi.org/10.3390/md19030148 | Animal experiment |
| 2016 | Exercise Training Reduces Liver Fat and Increases Rates of VLDL Clearance But Not VLDL Production in NAFLD. | https://doi.org/10.1210/jc.2016-2353 | Non-drug treatment research |
| 2021 | A Phase 2 Double Blinded, Randomized Controlled Trial of Saroglitazar in Patients With Nonalcoholic Steatohepatitis. | https://doi.org/10.1016/j.cgh.2020.10.051 | The study population was not diagnosed with diabetes |
| 2020 | Impact of obeticholic acid on the lipoprotein profile in patients with non-alcoholic steatohepatitis. | https://doi.org/10.1016/j.jhep.2019.10.006 | The study population was not diagnosed with diabetes |
| 2018 | The nonalcoholic fatty liver disease (NAFLD) fibrosis score, cardiovascular risk stratification and a strategy for secondary prevention with ezetimibe. | https://doi.org/10.1016/j.ijcard.2018.05.087 | Not relevant to our study |
| 2021 | Effects of fructose restriction on liver steatosis (FRUITLESS); a double-blind randomized controlled trial. | https://doi.org/10.1093/ajcn/nqaa332 | Not relevant to our study |
| 2019 | Partitioning of radiological, stress and biochemical changes in pre-diabetic women subjected to Diabetic Yoga Protocol. | https://doi.org/10.1016/j.dsx.2019.07.007 | Not relevant to our study |
| 2016 | Effects of endurance and endurance-strength exercise on biochemical parameters of liver function in women with abdominal obesity. | https://doi.org/10.1016/j.biopha.2016.02.017 | Not relevant to our study |
| 2019 | A carbohydrate-reduced high-protein diet improves HbA(1c) and liver fat content in weight stable participants with type 2 diabetes: a randomised controlled trial. | https://doi.org/10.1007/s00125-019-4956-4 | Non-drug treatment research |
| 2022 | Effect of Omega-3 Polyunsaturated Fatty Acids on Lipid Metabolism in Patients With Metabolic Syndrome and NAFLD. | https://doi.org/10.1002/hep4.1906 | The study population was not diagnosed with diabetes |
| 2016 | Twelve week liraglutide or sitagliptin does not affect hepatic fat in type 2 diabetes: a randomised placebo-controlled trial. | https://doi.org/10.1007/s00125-016-4100-7 | The study population was not diagnosed with NAFLD |
| 2011 | Treatment with insulin sensitizer metformin improves arterial properties, metabolic parameters, and liver function in patients with nonalcoholic fatty liver disease: a randomized, placebo-controlled trial. | https://doi.org/10.1016/j.metabol.2011.01.011 | The study population was not diagnosed with diabetes |
| 2016 | Effect of metformin treatment on circulating osteoprotegerin in patients with nonalcoholic fatty liver disease. | https://doi.org/10.1007/s12072-015-9649-6 | The study population was not diagnosed with diabetes |
| 2010 | Effects of a 1-year dietary intervention with n-3 polyunsaturated fatty acid-enriched olive oil on non-alcoholic fatty liver disease patients: a preliminary study. | https://doi.org/10.3109/09637486.2010.487480 | The study population was not diagnosed with diabetes |
| 2015 | Does metformin treatment influence bone formation in patients with nonalcoholic fatty liver disease? | https://doi.org/10.1055/s-0034-1395652 | The study population was not diagnosed with diabetes |
| 2021 | Protective effects of propolis on hepatic steatosis and fibrosis among patients with nonalcoholic fatty liver disease (NAFLD) evaluated by real-time two-dimensional shear wave elastography: A randomized clinical trial. | https://doi.org/10.1002/ptr.6937 | Not relevant to our study |
| 2020 | Combined effect of n-3 fatty acids and phytosterol esters on alleviating hepatic steatosis in non-alcoholic fatty liver disease subjects: a double-blind placebo-controlled clinical trial. | https://doi.org/10.1017/s0007114520000495 | The study population was not diagnosed with diabetes |
| 2023 | Letter to the editor about the effect of a fruit-rich diet on liver biomarkers, insulin resistance, and lipid profile in patients with non-alcoholic fatty liver disease: a randomized clinical trial"." | https://doi.org/10.1080/00365521.2022.2114808 | A letter |
| 2008 | Effects of n-3 polyunsaturated fatty acids in subjects with nonalcoholic fatty liver disease. | https://doi.org/10.1016/j.dld.2007.10.003 | The study population was not diagnosed with diabetes |
| 2015 | Plasma fatty acid composition in French-Canadian children with non-alcoholic fatty liver disease: Effect of n-3 PUFA supplementation. | https://doi.org/10.1016/j.plefa.2015.04.010 | The study population was children |
| 2016 | Liver Stiffness Evaluation by Transient Elastography in Type 2 Diabetes Mellitus Patients with Ultrasound-proven Steatosis. | https://doi.org/10.15403/jgld.2014.1121.252.lsf | Not relevant to our study |
| 2009 | Effect of a lifestyle intervention in patients with abnormal liver enzymes and metabolic risk factors. | https://doi.org/10.1111/j.1440-1746.2008.05694.x | Not relevant to our study |
| 2009 | Independent effects of physical activity in patients with nonalcoholic fatty liver disease. | https://doi.org/10.1002/hep.22940 | Non-drug treatment research |
| 2016 | Nutritional Strategies for the Individualized Treatment of Non-Alcoholic Fatty Liver Disease (NAFLD) Based on the Nutrient-Induced Insulin Output Ratio (NIOR). | https://doi.org/10.3390/ijms17071192 | Not relevant to our study |
| 2019 | Effects of tesamorelin on non-alcoholic fatty liver disease in HIV: a randomised, double-blind, multicentre trial. | https://doi.org/10.1016/s2352-3018(19)30338-8 | Not relevant to our study |
| 2021 | Growth Hormone Releasing Hormone Reduces Circulating Markers of Immune Activation in Parallel with Effects on Hepatic Immune Pathways in Individuals with HIV-infection and Nonalcoholic Fatty Liver Disease. | https://doi.org/10.1093/cid/ciab019 | Not relevant to our study |
| 2021 | Relationship of IGF-1 and IGF-Binding Proteins to Disease Severity and Glycemia in Nonalcoholic Fatty Liver Disease. | https://doi.org/10.1210/clinem/dgaa792 | Basic research |
| 2013 | [Additional advantages of mexicor used in combined therapy of coronary heat disease and diabetes mellitus of 2nd type]. |  | Not relevant to our study |
| 2014 | Inhibition of 11β-HSD1 with RO5093151 for non-alcoholic fatty liver disease: a multicentre, randomised, double-blind, placebo-controlled trial. | https://doi.org/10.1016/s2213-8587(13)70170-0 | The study population was not diagnosed with diabetes |
| 2017 | Acetyl-coenzyme A carboxylase inhibition reduces de novo lipogenesis in overweight male subjects: A randomized, double-blind, crossover study. | https://doi.org/10.1002/hep.29246 | Not relevant to our study |
| 2023 | Mobile health lifestyle intervention program leads to clinically significant loss of body weight in patients with NASH. | https://doi.org/10.1097/hc9.0000000000000052 | Not relevant to our study |
| 2022 | NASHFit: A randomized controlled trial of an exercise training program to reduce clotting risk in patients with NASH. | https://doi.org/10.1002/hep.32274 | Not relevant to our study |
| 2023 | Serum Fibroblast Growth Factor 21 Is Markedly Decreased following Exercise Training in Patients with Biopsy-Proven Nonalcoholic Steatohepatitis. | https://doi.org/10.3390/nu15061481 | Non-drug treatment research |
| 2009 | Metformin plus pentoxifylline versus prescriptive diet in non-alcoholic steatohepatitis (NASH): a randomized controlled pilot trial. | https://doi.org/10.1016/j.gcb.2009.05.010 | The study population was not diagnosed with diabetes |
| 2011 | [Efficacy comparison between bicyclol and polyene phosphatidylcholine treatments for the patients with nonalcoholic fatty liver disease]. | https://doi.org/10.3760/cma.j.issn.1007-3418.2011.07.019 | The study population was not diagnosed with diabetes |
| 2012 | Randomized trial of exercise effect on intrahepatic triglyceride content and lipid kinetics in nonalcoholic fatty liver disease. | https://doi.org/10.1002/hep.25548 | Non-drug treatment research |
| 2014 | The 'donations for decreased ALT (D4D)' prosocial behavior incentive scheme for NAFLD patients. | https://doi.org/10.1093/pubmed/fdt098 | Not relevant to our study |
| 2017 | Evaluation of High Dosages of Oral Meloxicam in American Kestrels ( Falco sparverius ). | https://doi.org/10.1647/2015-150 | Not relevant to our study |
| 2023 | Causal relationship between nonalcoholic fatty liver disease and different sleep traits: a bidirectional Mendelian randomized study. | https://doi.org/10.3389/fendo.2023.1159258 | Mendelian randomized study. |
| 2020 | A randomised, double-blind, placebo-controlled, multi-centre, dose-range, proof-of-concept, 24-week treatment study of lanifibranor in adult subjects with non-alcoholic steatohepatitis: Design of the NATIVE study. | https://doi.org/10.1016/j.cct.2020.106170 | The study population was not diagnosed with diabetes |
| 2005 | Magnetic resonance spectroscopy to measure hepatic triglyceride content: prevalence of hepatic steatosis in the general population. | https://doi.org/10.1152/ajpendo.00064.2004 | Not relevant to our study |
| 2020 | Energy-restricted Central-European diet stimulates liver microsomal function in obese postmenopausal women - a randomized nutritional trial with a comparison to energy-restricted Mediterranean diet. | https://doi.org/10.26355/eurrev_202011_23604 | Not relevant to our study |
| 2021 | Effect of focused ultrasound cavitation augmented with aerobic exercise on abdominal and intrahepatic fat in patients with non-alcoholic fatty liver disease: A randomized controlled trial. | https://doi.org/10.1371/journal.pone.0250337 | Non-drug treatment research |
| 2020 | Effect of Empagliflozin on Liver Steatosis and Fibrosis in Patients With Non-Alcoholic Fatty Liver Disease Without Diabetes: A Randomized, Double-Blind, Placebo-Controlled Trial. | https://doi.org/10.1007/s12325-020-01498-5 | The study population was not diagnosed with diabetes |
| 2022 | Ipragliflozin Improves the Hepatic Outcomes of Patients With Diabetes with NAFLD. | https://doi.org/10.1002/hep4.1696 | Unable to extract usable data |
| 2022 | Evaluation of the efficacy and safety of chum salmon milt deoxyribonucleic acid for improvement of hepatic functions: a placebo-controlled, randomised, double-blind, and parallel-group, pilot clinical trial. | https://doi.org/10.1039/d2fo01145j | Not relevant to our study |
| 2014 | The effects of ezetimibe on non-alcoholic fatty liver disease and glucose metabolism: a randomised controlled trial. | https://doi.org/10.1007/s00125-013-3149-9 | The study population was not diagnosed with diabetes |
| 2022 | The effects of vitamin B12 supplementation on metabolic profile of patients with non-alcoholic fatty liver disease: a randomized controlled trial. | https://doi.org/10.1038/s41598-022-18195-8 | The study population was not diagnosed with diabetes |
| 2015 | Cross-sectional and longitudinal evaluation of liver volume and total liver fat burden in adults with nonalcoholic steatohepatitis. | https://doi.org/10.1007/s00261-014-0175-0 | Not relevant to our study |
| 2016 | [Efficacy of Zaozhu Yinchen Recipe for Treating Non-alcoholic Steatohepatitis and its Effect on Free Fatty Acid and TNF-alpha]. |  | Study on the treatment of traditional Chinese medicine |
| 2023 | Impact of nonalcoholic fatty liver disease status change on antiviral efficacy of nucleos(t)ide analogues in HBeAg-positive chronic hepatitis B. | https://doi.org/10.1002/jmv.28501 | Not relevant to our study |
| 2022 | A randomized, placebo-controlled clinical trial of hydrogen/oxygen inhalation for non-alcoholic fatty liver disease. | https://doi.org/10.1111/jcmm.17456 | Not relevant to our study |
| 2018 | Evaluation of the effect of Lactobacillus reuteri V3401 on biomarkers of inflammation, cardiovascular risk and liver steatosis in obese adults with metabolic syndrome: a randomized clinical trial (PROSIR). | https://doi.org/10.1186/s12906-018-2371-x | Not relevant to our study |
| 2022 | Dietary carbohydrate restriction augments weight loss-induced improvements in glycaemic control and liver fat in individuals with type 2 diabetes: a randomised controlled trial. | https://doi.org/10.1007/s00125-021-05628-8 | Non-drug treatment research |
| 2018 | Safety, tolerability and pharmacodynamics of apical sodium-dependent bile acid transporter inhibition with volixibat in healthy adults and patients with type 2 diabetes mellitus: a randomised placebo-controlled trial. | https://doi.org/10.1186/s12876-017-0736-0 | Not relevant to our study |
| 2018 | Evaluation of a High Concentrate Omega-3 for Correcting the Omega-3 Fatty Acid Nutritional Deficiency in Non-Alcoholic Fatty Liver Disease (CONDIN). | https://doi.org/10.3390/nu10081126 | The study population was not diagnosed with diabetes |
| 2009 | Liver safety in patients with type 2 diabetes treated with pioglitazone: results from a 3-year, randomized, comparator-controlled study in the US. | https://doi.org/10.2165/11316510-000000000-00000 | The study population was not diagnosed with NAFLD |
| 2005 | AICAR, an AMPK activator, has protective effects on alcohol-induced fatty liver in rats. | https://doi.org/10.1097/01.alc.0000191126.11479.69 | Alcohol-induced fatty liver |
| 2011 | Rosiglitazone versus rosiglitazone and metformin versus rosiglitazone and losartan in the treatment of nonalcoholic steatohepatitis in humans: a 12-month randomized, prospective, open- label trial. | https://doi.org/10.1002/hep.24558 | The study population was not diagnosed with diabetes |
| 2019 | Norursodeoxycholic acid versus placebo in the treatment of non-alcoholic fatty liver disease: a double-blind, randomised, placebo-controlled, phase 2 dose-finding trial. | https://doi.org/10.1016/s2468-1253(19)30184-0 | The study population was not diagnosed with diabetes |
| 2013 | The treatment with ursodeoxycholic acid in elderly patients affected by NAFLD and metabolic syndrome: a case-control study. | https://doi.org/10.7417/ct.2013.1550 | A case-control study. |
| 2018 | Aerobic Physical Activity and a Low Glycemic Diet Reduce the AA/EPA Ratio in Red Blood Cell Membranes of Patients with NAFLD. | https://doi.org/10.3390/nu10091299 | Non-drug treatment research |
| 2023 | Clinical effectiveness of α-lipoic acid, myo-inositol and propolis supplementation on metabolic profiles and liver function in obese patients with NAFLD: A randomized controlled clinical trial. | https://doi.org/10.1016/j.clnesp.2023.02.016 | The study population was not diagnosed with diabetes |
| 2020 | Oleoylethanolamide supplementation in obese patients newly diagnosed with non-alcoholic fatty liver disease: Effects on metabolic parameters, anthropometric indices, and expression of PPAR-α, UCP1, and UCP2 genes. | https://doi.org/10.1016/j.phrs.2020.104770 | The study population was not diagnosed with diabetes |
| 2021 | Expression of NF-κB, IL-6, and IL-10 genes, body composition, and hepatic fibrosis in obese patients with NAFLD-Combined effects of oleoylethanolamide supplementation and calorie restriction: A triple-blind randomized controlled clinical trial. | https://doi.org/10.1002/jcp.29870 | Not relevant to our study |
| 2017 | Impact of liver fat on the differential partitioning of hepatic triacylglycerol into VLDL subclasses on high and low sugar diets. | https://doi.org/10.1042/cs20171208 | Non-drug treatment research |
| 2013 | Beneficial effect of a weight-stable, low-fat/low-saturated fat/low-glycaemic index diet to reduce liver fat in older subjects. | https://doi.org/10.1017/s0007114512002966 | Non-drug treatment research |
| 2022 | The effect of sesame oil consumption compared to sunflower oil on lipid profile, blood pressure, and anthropometric indices in women with non-alcoholic fatty liver disease: a randomized double-blind controlled trial. | https://doi.org/10.1186/s13063-022-06451-1 | The study population was not diagnosed with diabetes |
| 2011 | Effects of Lactobacillus rhamnosus strain GG in pediatric obesity-related liver disease. | https://doi.org/10.1097/MPG.0b013e31821f9b85 | The study population was children |
| 2014 | A randomized trial of iron depletion in patients with nonalcoholic fatty liver disease and hyperferritinemia. | https://doi.org/10.3748/wjg.v20.i11.3002 | Not relevant to our study |
| 2021 | Minor Changes in the Composition and Function of the Gut Microbiota During a 12-Week Whole Grain Wheat or Refined Wheat Intervention Correlate with Liver Fat in Overweight and Obese Adults. | https://doi.org/10.1093/jn/nxaa312 | Not relevant to our study |
| 2011 | Pentoxifylline for the treatment of non-alcoholic steatohepatitis: a randomized controlled trial. |  | The study population was not diagnosed with diabetes |
| 2023 | Bariatric-metabolic surgery versus lifestyle intervention plus best medical care in non-alcoholic steatohepatitis (BRAVES): a multicentre, open-label, randomised trial. | https://doi.org/10.1016/s0140-6736(23)00634-7 | Non-drug treatment research |
| 2009 | Clinical trial: a nutritional supplement Viusid, in combination with diet and exercise, in patients with nonalcoholic fatty liver disease. | https://doi.org/10.1111/j.1365-2036.2009.04122.x | Not relevant to our study |
| 2021 | Impact of the Association Between PNPLA3 Genetic Variation and Dietary Intake on the Risk of Significant Fibrosis in Patients With NAFLD. | https://doi.org/10.14309/ajg.0000000000001072 | Not relevant to our study |
| 2017 | ANGPTL8 (Betatrophin) is Expressed in Visceral Adipose Tissue and Relates to Human Hepatic Steatosis in Two Independent Clinical Collectives. | https://doi.org/10.1055/s-0043-102950 | Basic research |
| 2022 | Randomized placebo-controlled trial of losartan for pediatric NAFLD. | https://doi.org/10.1002/hep.32403 | The study population was children |
| 2009 | Fructose and oxidized low-density lipoprotein in pediatric nonalcoholic fatty liver disease: a pilot study. | https://doi.org/10.1001/archpediatrics.2009.93 | The study population was children |
| 2017 | A Randomized Trial of Silymarin for the Treatment of Nonalcoholic Steatohepatitis. | https://doi.org/10.1016/j.cgh.2017.04.016 | The study population was not diagnosed with diabetes |
| 2008 | Effect of lifestyle intervention on non-alcoholic fatty liver disease in Chinese obese children. | https://doi.org/10.3748/wjg.14.1598 | The study population was children |
| 2022 | Danshao Shugan Granule therapy for non-alcoholic fatty liver disease. | https://doi.org/10.1186/s12944-022-01689-9 | Study on the treatment of traditional Chinese medicine |
| 2023 | Letter to the editor: Comment on the gut microbiome, mild cognitive impairment, and probiotics: A randomized clinical trial in middle-aged and older adults"." | https://doi.org/10.1016/j.clnu.2023.01.004 | A Letter |
| 2022 | Effects of sitagliptin on intrahepatic lipid content in patients with non-alcoholic fatty liver disease. | https://doi.org/10.3389/fendo.2022.866189 | The study population was not diagnosed with diabetes |
| 2021 | Safety, Pharmacokinetics, Pharmacodynamics, and Formulation of Liver-Distributed Farnesoid X-Receptor Agonist TERN-101 in Healthy Volunteers. | https://doi.org/10.1002/cpdd.960 | Not relevant to our study |
| 2020 | Quantifying and monitoring fibrosis in non-alcoholic fatty liver disease using dual-photon microscopy. | https://doi.org/10.1136/gutjnl-2019-318841 | Not relevant to our study |
| 2007 | [Yiqi Huoxue Recipe combined with polyene phosphatidycholine capsule in treating 50 patients with non-alcoholic fatty hepatitis]. |  | Study on the treatment of traditional Chinese medicine |
| 2022 | Effects of Orlistat or Telmisartan on the Serum Free Fatty Acids in Non-alcoholic Fatty Liver Disease Patients: An Open-Labeled Randomized Controlled Study. | https://doi.org/10.5152/tjg.2020.19365 | The study population was not diagnosed with diabetes |
| 2011 | Abnormal liver function in children with metabolic syndrome from a UK-based obesity clinic. | https://doi.org/10.1136/adc.2010.190975 | The study population was children |
| 2023 | Effects of Time-Restricted Eating on Nonalcoholic Fatty Liver Disease: The TREATY-FLD Randomized Clinical Trial. | https://doi.org/10.1001/jamanetworkopen.2023.3513 | Non-drug treatment research |
| 2023 | Effect of unsaturated fat and protein intake on liver fat in people at risk of unhealthy aging: 1-year results of a randomized controlled trial. | https://doi.org/10.1016/j.ajcnut.2023.01.010 | Not relevant to our study |
| 2020 | HDL-apoA-I kinetics in response to 16 wk of exercise training in men with nonalcoholic fatty liver disease. | https://doi.org/10.1152/ajpendo.00019.2020 | Not relevant to our study |
| 2015 | Proposed trial: safety and efficacy of resveratrol for the treatment of non-alcoholic fatty liver disease (NAFLD) and associated insulin resistance in adolescents who are overweight or obese adolescents - rationale and protocol. | https://doi.org/10.1139/bcb-2014-0136 | The study population was children |
| 2016 | Opposite associations between alanine aminotransferase and γ-glutamyl transferase levels and all-cause mortality in type 2 diabetes: Analysis of the Fenofibrate Intervention and Event Lowering in Diabetes (FIELD) study. | https://doi.org/10.1016/j.metabol.2015.12.008 | Not relevant to our study |
| 2016 | Low alanine aminotransferase levels and higher number of cardiovascular events in people with Type 2 diabetes: analysis of the Fenofibrate Intervention and Event Lowering in Diabetes (FIELD) study. | https://doi.org/10.1111/dme.12972 | Not relevant to our study |
| 2018 | Energy-matched moderate and high intensity exercise training improves nonalcoholic fatty liver disease risk independent of changes in body mass or abdominal adiposity - A randomized trial. | https://doi.org/10.1016/j.metabol.2017.08.012 | Non-drug treatment research |
| 2023 | Shorter leukocyte telomere length protects against NAFLD progression in children. | https://doi.org/10.1038/s41598-023-31149-y | The study population was children |
| 2013 | Community-based lifestyle modification programme for non-alcoholic fatty liver disease: a randomized controlled trial. | https://doi.org/10.1016/j.jhep.2013.04.013 | Non-drug treatment research |
| 2013 | Treatment of nonalcoholic steatohepatitis with probiotics. A proof-of-concept study. |  | The study population was not diagnosed with diabetes |
| 2013 | Treatment of non-alcoholic steatohepatitis with Phyllanthus urinaria: a randomized trial. | https://doi.org/10.1111/j.1440-1746.2012.07286.x | The study population was not diagnosed with diabetes |
| 2018 | Beneficial effects of lifestyle intervention in non-obese patients with non-alcoholic fatty liver disease. | https://doi.org/10.1016/j.jhep.2018.08.011 | Non-drug treatment research |
| 2012 | [Clinical study on prevention efficacy of Jianpi Huatan Fang in treating non-alcoholic fatty liver disease in children]. |  | Study on the treatment of traditional Chinese medicine |
| 2021 | Dose-response relationship between serum fibroblast growth factor 21 and liver fat content in non-alcoholic fatty liver disease. | https://doi.org/10.1016/j.diabet.2020.101221 | Not relevant to our study |
| 2022 | Effect of 5:2 Fasting Diet on Liver Fat Content in Patients with Type 2 Diabetic with Nonalcoholic Fatty Liver Disease. | https://doi.org/10.1089/met.2022.0014 | Non-drug treatment research |
| 2011 | [Research on therapeutic effect and hemorrheology change of berberine in new diagnosed patients with type 2 diabetes combining nonalcoholic fatty liver disease]. |  | Study on the treatment of traditional Chinese medicine |
| 2017 | Role of UDP-Glucuronosyltransferase 1A1 in the Metabolism and Pharmacokinetics of Silymarin Flavonolignans in Patients with HCV and NAFLD. | https://doi.org/10.3390/molecules22010142 | Not relevant to our study |
| 2020 | High-protein diet more effectively reduces hepatic fat than low-protein diet despite lower autophagy and FGF21 levels. | https://doi.org/10.1111/liv.14596 | Not relevant to our study |
| 2022 | Effect of growth hormone therapy on liver enzyme and other cardiometabolic risk factors in boys with obesity and nonalcoholic fatty liver disease. | https://doi.org/10.1186/s12902-022-00967-y | Not relevant to our study |
| 2022 | Effect of Fecal Microbiota Transplantation on Non-Alcoholic Fatty Liver Disease: A Randomized Clinical Trial. | https://doi.org/10.3389/fcimb.2022.759306 | The study population was not diagnosed with diabetes |
| 2017 | Effects of Oral Antidiabetic Drugs on Changes in the Liver-to-Spleen Ratio on Computed Tomography and Inflammatory Biomarkers in Patients With Type 2 Diabetes and Nonalcoholic Fatty Liver Disease. | https://doi.org/10.1016/j.clinthera.2017.01.015 | Unable to extract usable data |
| 2022 | Inhibition of 11β-Hydroxysteroid dehydrogenase-1 with AZD4017 in patients with nonalcoholic steatohepatitis or nonalcoholic fatty liver disease: A randomized, double-blind, placebo-controlled, phase II study. | https://doi.org/10.1111/dom.14646 | Not relevant to our study |
| 2021 | The efficacy of calcitriol treatment in non-alcoholic fatty liver patients with different genotypes of vitamin D receptor FokI polymorphism. | https://doi.org/10.1186/s40360-021-00485-y | Not relevant to our study |
| 2017 | Comparison of fenofibrate and pioglitazone effects on patients with nonalcoholic fatty liver disease. | https://doi.org/10.1097/meg.0000000000000981 | The study population was not diagnosed with diabetes |
| 2021 | Gender differences in the efficacy of pioglitazone treatment in nonalcoholic fatty liver disease patients with abnormal glucose metabolism. | https://doi.org/10.1186/s13293-020-00344-1 | Not relevant to our study |
| 2015 | Efficacy of Berberine in Patients with Non-Alcoholic Fatty Liver Disease. | https://doi.org/10.1371/journal.pone.0134172 | Study on the treatment of traditional Chinese medicine |
| 2019 | Liraglutide, Sitagliptin, and Insulin Glargine Added to Metformin: The Effect on Body Weight and Intrahepatic Lipid in Patients With Type 2 Diabetes Mellitus and Nonalcoholic Fatty Liver Disease. | https://doi.org/10.1002/hep.30320 | Only one article on the same kind of intervention |
| 2014 | Effect of piglitazone and metformin on retinol-binding protein-4 and adiponectin in patients with type 2 diabetes mellitus complicated with non-alcohol fatty acid liver diseases. | https://doi.org/10.3881/j.issn.1000-503X.2014.03.015 | Non-English article |
| 2023 | The effect of 12 week-maximum fat oxidation intensity (FATmax) exercise on microvascular function in obese patients with nonalcoholic fatty liver disease and its mechanism. | https://doi.org/10.4149/gpb_2023004 | Non-drug treatment research |
| 2023 | Global publication trends and research hotspots of the gut-liver axis in NAFLD: A bibliometric analysis. | https://doi.org/10.3389/fendo.2023.1121540 | A bibliometric analysis. |
| 2022 | A novel model for detecting advanced fibrosis in patients with nonalcoholic fatty liver disease. | https://doi.org/10.1002/dmrr.3570 | Not relevant to our study |
| 2023 | Effect of Cornus mas L. fruit extract on blood pressure, anthropometric and body composition indices in patients with non-alcoholic fatty liver disease: A double-blind randomized controlled trial. | https://doi.org/10.1016/j.clnesp.2023.04.018 | Not relevant to our study |
| 2021 | The efficacy of flaxseed and hesperidin on non-alcoholic fatty liver disease: an open-labeled randomized controlled trial. | https://doi.org/10.1038/s41430-020-0679-3 | The study population was not diagnosed with diabetes |
| 2016 | Flaxseed supplementation in non-alcoholic fatty liver disease: a pilot randomized, open labeled, controlled study. | https://doi.org/10.3109/09637486.2016.1161011 | The study population was not diagnosed with diabetes |
| 2021 | Effects of lifestyle interventions on epigenetic signatures of liver fat: Central randomized controlled trial. | https://doi.org/10.1111/liv.14916 | Not relevant to our study |
| 2021 | Effect of green-Mediterranean diet on intrahepatic fat: the DIRECT PLUS randomised controlled trial. | https://doi.org/10.1136/gutjnl-2020-323106 | Not relevant to our study |
| 2020 | Preventive Effect of High-Dose Digestive Enzyme Management on Development of Nonalcoholic Fatty Liver Disease after Pancreaticoduodenectomy: A Randomized Controlled Clinical Trial. | https://doi.org/10.1016/j.jamcollsurg.2020.08.761 | Not relevant to our study |
| 2017 | Serum high mobility group box 1 protein levels are not associated with either histological severity or treatment response in children and adults with nonalcoholic fatty liver disease. | https://doi.org/10.1371/journal.pone.0185813 | Not relevant to our study |
| 2017 | Laboratory parameter-based machine learning model for excluding non-alcoholic fatty liver disease (NAFLD) in the general population. | https://doi.org/10.1111/apt.14172 | Not relevant to our study |
| 2022 | Combination of tofogliflozin and pioglitazone for NAFLD: Extension to the ToPiND randomized controlled trial. | https://doi.org/10.1002/hep4.1993 | The study population was not diagnosed with diabetes |
| 2021 | Pharmacokinetics and Pharmacodynamics of Ursodeoxycholic Acid in an Overweight Population With Abnormal Liver Function. | https://doi.org/10.1002/cpdd.790 | Not relevant to our study |
| 2010 | [Taurine in the treatment of non-alcoholic fatty liver disease]. |  | The study population was not diagnosed with diabetes |
| 2023 | Pharmacokinetics, pharmacodynamics, safety and tolerability of cilofexor, a novel nonsteroidal Farnesoid X receptor agonist, in healthy volunteers. | https://doi.org/10.1111/cts.13469 | Not relevant to our study |
| 2018 | The conundrum of cryptogenic cirrhosis: Adverse outcomes without treatment options. | https://doi.org/10.1016/j.jhep.2018.08.013 | Not relevant to our study |
| 2019 | Obeticholic acid for the treatment of non-alcoholic steatohepatitis: interim analysis from a multicentre, randomised, placebo-controlled phase 3 trial. | https://doi.org/10.1016/s0140-6736(19)33041-7 | The study population was not diagnosed with diabetes |
| 2018 | Improvement of hepatic fibrosis and patient-reported outcomes in non-alcoholic steatohepatitis treated with selonsertib. | https://doi.org/10.1111/liv.13706 | The study population was not diagnosed with diabetes |
| 2022 | Obeticholic Acid Impact on Quality of Life in Patients With Nonalcoholic Steatohepatitis: REGENERATE 18-Month Interim Analysis. | https://doi.org/10.1016/j.cgh.2021.07.020 | The study population was not diagnosed with diabetes |
| 2022 | Hepatic Fat Reduction Due to Resmetirom in Patients With Nonalcoholic Steatohepatitis Is Associated With Improvement of Quality of Life. | https://doi.org/10.1016/j.cgh.2021.07.039 | The study population was not diagnosed with diabetes |
| 2015 | [Treating non-alcoholic fatty liver disease patients of Gan stagnation Pi deficiency syndrome by tiaogan lidi recipe: a randomized controlled clinical trial]. |  | Study on the treatment of traditional Chinese medicine |
| 2022 | Helicobacter pylori infection eradication for nonalcoholic fatty liver disease: a randomized controlled trial. | https://doi.org/10.1038/s41598-022-23746-0 | Not relevant to our study |
| 2022 | The effects of Mediterranean diet on hepatic steatosis, oxidative stress, and inflammation in adolescents with non-alcoholic fatty liver disease: A randomized controlled trial. | https://doi.org/10.1111/ijpo.12872 | The study population was children |
| 2018 | The effects of Zataria multiflora Boiss. (Shirazi thyme) on nonalcoholic fatty liver disease and insulin resistance: A randomized double-blind placebo-controlled clinical trial. | https://doi.org/10.1016/j.ctim.2018.09.010 | Not relevant to our study |
| 2018 | Haptoglobin Genotype and Vitamin E Versus Placebo for the Treatment of Nondiabetic Patients with Nonalcoholic Steatohepatitis in China: A Multicenter, Randomized, Placebo-Controlled Trial Design. | https://doi.org/10.1007/s12325-018-0670-8 | The study population was not diagnosed with diabetes |
| 2012 | Pentoxifylline decreases oxidized lipid products in nonalcoholic steatohepatitis: new evidence on the potential therapeutic mechanism. | https://doi.org/10.1002/hep.25778 | Not relevant to our study |
| 2011 | Pentoxifylline improves nonalcoholic steatohepatitis: a randomized placebo-controlled trial. | https://doi.org/10.1002/hep.24544 | The study population was not diagnosed with diabetes |
| 2014 | Effect of resistance training on non-alcoholic fatty-liver disease a randomized-clinical trial. | https://doi.org/10.3748/wjg.v20.i15.4382 | Non-drug treatment research |
| 2006 | A double-blind randomized placebo-controlled trial of orlistat for the treatment of nonalcoholic fatty liver disease. | https://doi.org/10.1016/j.cgh.2006.02.004 | The study population was not diagnosed with diabetes |
| 2021 | Combined metabolic activators therapy ameliorates liver fat in nonalcoholic fatty liver disease patients. | https://doi.org/10.15252/msb.202110459 | The study population was not diagnosed with diabetes |
| 2016 | Effects of Moderate and Vigorous Exercise on Nonalcoholic Fatty Liver Disease: A Randomized Clinical Trial. | https://doi.org/10.1001/jamainternmed.2016.3202 | Non-drug treatment research |
| 2017 | Long-term effect of exercise on improving fatty liver and cardiovascular risk factors in obese adults: A 1-year follow-up study. | https://doi.org/10.1111/dom.12809 | Not relevant to our study |
| 2015 | A CONSORT-compliant, randomized, double-blind, placebo-controlled pilot trial of purified anthocyanin in patients with nonalcoholic fatty liver disease. | https://doi.org/10.1097/md.0000000000000758 | Not relevant to our study |
| 2008 | The effect of QuYuHuaTanTongLuo Decoction on the non-alcoholic steatohepatitis. | https://doi.org/10.1016/j.ctim.2007.08.004 | Study on the treatment of traditional Chinese medicine |
| 2004 | [Clinical observation on treatment of nonalcoholic fatty liver disease complicating hyperuricemia by Zhifang I Decoction]. | https://doi.org/10.3736/jcim20040408 | Study on the treatment of traditional Chinese medicine |
| 2008 | Effects of n-3 polyunsaturated fatty acids from seal oils on nonalcoholic fatty liver disease associated with hyperlipidemia. | https://doi.org/10.3748/wjg.14.6395 | The study population was not diagnosed with diabetes |
| 2022 | Effects of Clostridium butyricum Capsules Combined with Rosuvastatin on Intestinal Flora, Lipid Metabolism, Liver Function and Inflammation in NAFLD Patients. | https://doi.org/10.14715/cmb/2022.68.2.10 | Not relevant to our study |
| 2022 | Upregulated NLRP3 inflammasome activation is attenuated by anthocyanins in patients with nonalcoholic fatty liver disease: A case-control and an intervention study. | https://doi.org/10.1016/j.clinre.2021.101843 | A case-control study. |
| 2017 | Efficacy of docosahexaenoic acid-choline-vitamin E in paediatric NASH: a randomized controlled clinical trial. | https://doi.org/10.1139/apnm-2016-0689 | The study population was children |
| 2014 | Lifestyle intervention involving calorie restriction with or without aerobic exercise training improves liver fat in adults with visceral adiposity. | https://doi.org/10.1155/2014/197216 | Not relevant to our study |

# **Table S7** Certainty of evidence for direct, indirect and network estimates

| **Outcome: liver Fat Content** | | **Direct estimate** | | | | **Indirect estimate** | | | | | | **Networkestimate** | | | | | |
| --- | --- | --- | --- | --- | --- | --- | --- | --- | --- | --- | --- | --- | --- | --- | --- | --- | --- |
| **Comparison** | | **MD(95%CI)** | | | **Certainty** | **MD(95%CI)** | | | **Certainty** | | | **MD(95%CI)** | | | **Certainty** | | |
| Liraglutide vs. Exenatide | | — | | | — | — | | | lowm ++○○ | | | -3.60 (-11.11, 3.90) | | | low ++○○ | | |
| Liraglutide vs. Metformin | | 5.70(3.70, 7.70) | | | lowb,c ++○○ | — | | | lowm ++○○ | | | 5.70 (3.75, 7.65) | | | low ++○○ | | |
| Liraglutide vs. Insulin Glargine | | 3.40 (1.00, 5.70) | | | lowb,c ++○○ | — | | | lowm ++○○ | | | 3.40 (1.16, 5.65) | | | low ++○○ | | |
| Liraglutide vs. Pioglitazone | | 2.50(0.77, 4.20) | | | lowb,c ++○○ | — | | | — | | | 2.50 (0.67, 4.30) | | | low ++○○ | | |
| Liraglutide vs. Tofogliflozin | | — | | | — | — | | | lowm ++○○ | | | 5.92 (1.53, 10.36) | | | low ++○○ | | |
| Exenatide vs. Metformin | | — | | | — | — | | | lowm ++○○ | | | 9.31 (1.75, 16.85) | | | low ++○○ | | |
| Exenatide vs. Insulin Glargine | | 7.00 (1.90, 12.00) | | | lowb,c ++○○ | — | | | — | | | 7.00 (-0.13, 14.14) | | | low ++○○ | | |
| Exenatide vs. Pioglitazone | | — | | | — | — | | | — | | | 6.10 (-1.56, 13.83) | | | Very Low +○○○ | | |
| Exenatide vs. Tofogliflozin | | — | | | — | — | | | — | | | 9.55 (0.85, 18.23) | | | Very Low +○○○ | | |
| Metformin vs. Insulin Glargine | | -2.30(-4.90, 0.28) | | | lowb,c ++○○ | — | | | lowm ++○○ | | | -2.29 (-4.68, 0.08) | | | low ++○○ | | |
| Metformin vs. Pioglitazone | | — | | | — | — | | | lowm ++○○ | | | -3.20 (-5.85, -0.54) | | | low ++○○ | | |
| Metformin vs. Tofogliflozin | | — | | | — | — | | | — | | | 0.22 (-4.62, 5.06) | | | Very Low +○○○ | | |
| Insulin Glargine vs. Pioglitazone | | — | | | — | — | | | lowm ++○○ | | | -0.90 (-3.79, 1.98) | | | low ++○○ | | |
| Insulin Glargine vs. Tofogliflozin | | — | | | — | — | | | — | | | 2.51 (-2.46, 7.49) | | | Very Low +○○○ | | |
| Pioglitazone vs. Tofogliflozin | | 3.4(0.35, 6.5) | | | lowb,c ++○○ | — | | | — | | | 3.43 (-0.61, 7.46) | | | low ++○○ | | |
| I^2^ | | 100% | | | |  | | |  | | | 0% | | | | | |
| PSRF=1.02 | |  | | | |  | | |  | | |  | | | | | |
|  | |  | | |  |  | | |  | | |  | | |  | | |
| **Outcome: HbA1c** | |  | | |  |  | | |  | | |  | | |  | | |
| Liraglutide vs. Exenatide | — | | | — | | — | | low^m^ ++○○ | | -0.32 (-0.52,-0.12) | | | | low ++○○ | | |  |
| Liraglutide vs. Metformin | -0.18(-0.36, 0.03) | | | low^b,d^ ++○○ | | — | | low^m^ ++○○ | | -0.23 (-0.32, -0.14) | | | | low ++○○ | | |  |
| Liraglutide vs. Insulin Glargine | 0.20(-0.67, 1.10) | | | low^b,c^ ++○○ | | -0.16(-0.78, 0.45) | | low^m^ ++○○ | | -0.06 (-0.44, 0.31) | | | | low ++○○ | | |  |
| Liraglutide vs. Gliclazide | 0.43(0.20, 0.65) | | | Moderate^a^ +++○ | | — | | low^g^ ++○○ | | 0.47 (0.39, 0.56) | | | | Moderate +++○ | | |  |
| Liraglutide vs. Omega 3 | — | | | — | | — | | — | | 0.86 (0.23, 1.49) | | | | Very Low +○○○ | | |  |
| Liraglutide vs. Pioglitazone | 0.40(-0.54, 1.30) | | | low^b,c^ ++○○ | | — | | — | | -0.05 (-0.64, 0.55) | | | | low ++○○ | | |  |
| Liraglutide vs. Dapagliflozin | — | | | — | | — | | low^g^ ++○○ | | -0.07 (-0.66, 0.53) | | | | low ++○○ | | |  |
| Liraglutide vs. lpragliflozin | — | | | — | | — | | low^m^ ++○○ | | -0.19 (-0.9, 0.51) | | | | low ++○○ | | |  |
| Liraglutide vs. Empagliflozin | — | | | — | | — | | low^m^ ++○○ | | 0.40 (-0.22, 1.02) | | | | low ++○○ | | |  |
| Liraglutide vs. Tofogliflozin | — | | | — | | — | | low^m^ ++○○ | | 0.24 (-0.36, 0.85) | | | | low ++○○ | | |  |
| Liraglutide vs. Glimepiride | — | | | — | | — | | low^m^ ++○○ | | 0.14 (-0.46, 0.74) | | | | low ++○○ | | |  |
| Liraglutide vs. Sitagliptin | — | | | — | | — | | — | | 0.32 (-0.27, 0.91) | | | | Very Low +○○○ | | |  |
| Liraglutide vs. STA-TRE | — | | | — | | — | | low^m^ ++○○ | | 0.69 (0.10, 1.27) | | | | low ++○○ | | |  |
| Exenatide vs. Metformin | 0.06(-0.27, 0.39) | | | low^b,c^ ++○○ | | — | | low^m^ ++○○ | | 0.09 (-0.09, 0.27) | | | | low ++○○ | | |  |
| Exenatide vs. Insulin Glargine | 0.46(-0.10, 1.00) | | | low^b,c^ ++○○ | | -0.27(-1.20, 0.63) | | low^m^ ++○○ | | 0.26 (-0.11, 0.63) | | | | low ++○○ | | |  |
| Exenatide vs. Gliclazide | — | | | — | | — | | low^g^ ++○○ | | 0.79 (0.59, 1.00) | | | | low ++○○ | | |  |
| Exenatide vs. Omega 3 | — | | | — | | — | | — | | 1.18 (0.53, 1.84) | | | | Very Low +○○○ | | |  |
| Exenatide vs. Pioglitazone | — | | | — | | — | | — | | 0.27 (-0.35, 0.89) | | | | Very Low +○○○ | | |  |
| Exenatide vs. Dapagliflozin | — | | | — | | — | | — | | 0.26 (-0.37, 0.87) | | | | Very Low +○○○ | | |  |
| Exenatide vs. lpragliflozin | — | | | — | | — | | — | | 0.13 (-0.60, 0.86) | | | | Very Low +○○○ | | |  |
| Exenatide vs. Empagliflozin | — | | | — | | — | | — | | 0.72 (0.07, 1.36) | | | | Very Low +○○○ | | |  |
| Exenatide vs. Tofogliflozin | — | | | — | | — | | — | | 0.56 (-0.06, 1.19) | | | | Very Low +○○○ | | |  |
| Exenatide vs. Glimepiride | — | | | — | | — | | — | | 0.46 (-0.16, 1.08) | | | | Very Low +○○○ | | |  |
| Exenatide vs. Sitagliptin | — | | | — | | — | | — | | 0.64 (0.03, 1.26) | | | | Very Low +○○○ | | |  |
| Exenatide vs. STA-TRE | — | | | — | | — | | low^m^ ++○○ | | 1.01 (0.40, 1.62) | | | | low ++○○ | | |  |
| Metformin vs. Insulin Glargine | -0.40(-1.10, 0.32) | | | low^b,c^ ++○○ | | 0.52(-0.11, 1.10) | | low^m^ ++○○ | | 0.17 (-0.20, 0.53) | | | | low ++○○ | | |  |
| Metformin vs. Gliclazide | 0.75(0.52, 0.98) | | | Moderate^a^ +++○ | | — | | low^g^ ++○○ | | 0.70 (0.61, 0.79) | | | | Moderate +++○ | | |  |
| Metformin vs. Omega 3 | — | | | — | | — | | low^m^ ++○○ | | 1.09 (0.46, 1.72) | | | | low ++○○ | | |  |
| Metformin vs. Pioglitazone | — | | | — | | — | | low^m^ ++○○ | | 0.18 (-0.41, 0.77) | | | | low ++○○ | | |  |
| Metformin vs. Dapagliflozin | — | | | — | | — | | low^i^ ++○○ | | 0.17 (-0.43, 0.76) | | | | low ++○○ | | |  |
| Metformin vs. lpragliflozin | — | | | — | | — | | low^m^ ++○○ | | 0.04 (-0.67, 0.74) | | | | low ++○○ | | |  |
| Metformin vs. Empagliflozin | — | | | — | | — | | Very Low^l^ +○○○ | | 0.63 (0.01, 1.24) | | | | Very Low +○○○ | | |  |
| Metformin vs. Tofogliflozin | — | | | — | | — | | — | | 0.47 (-0.13, 1.08) | | | | Very Low +○○○ | | |  |
| Metformin vs. Glimepiride | — | | | — | | — | | — | | 0.37 (-0.23, 0.96) | | | | Very Low +○○○ | | |  |
| Metformin vs. Sitagliptin | — | | | — | | — | | low^m^ ++○○ | | 0.55 (-0.04, 1.14) | | | | low ++○○ | | |  |
| Metformin vs. STA-TRE | 0.60(-0.20, 1.40) | | | low^b,c^ ++○○ | | — | | — | | 0.92 (0.33, 1.50) | | | | low ++○○ | | |  |
| Insulin Glargine vs. Gliclazide | — | | | — | | — | | low^g^ ++○○ | | 0.54 (0.16, 0.92) | | | | low ++○○ | | |  |
| Insulin Glargine vs. Omega 3 | — | | | — | | — | | — | | 0.92 (0.20, 1.66) | | | | Very Low +○○○ | | |  |
| Insulin Glargine vs. Pioglitazone | — | | | — | | — | | low^m^ ++○○ | | 0.02 (-0.68, 0.71) | | | | low ++○○ | | |  |
| Insulin Glargine vs. Dapagliflozin | — | | | — | | — | | — | | 0 (-0.70, 0.69) | | | | Very Low +○○○ | | |  |
| Insulin Glargine vs. lpragliflozin | — | | | — | | — | | — | | -0.13 (-0.92, 0.66) | | | | Very Low +○○○ | | |  |
| Insulin Glargine vs. Empagliflozin | — | | | — | | — | | — | | 0.46 (-0.25, 1.18) | | | | Very Low +○○○ | | |  |
| Insulin Glargine vs. Tofogliflozin | — | | | — | | — | | — | | 0.31 (-0.40, 1.01) | | | | Very Low +○○○ | | |  |
| Insulin Glargine vs. Glimepiride | — | | | — | | — | | — | | 0.20 (-0.50, 0.90) | | | | Very Low +○○○ | | |  |
| Insulin Glargine vs. Sitagliptin | — | | | — | | — | | — | | 0.38 (-0.31, 1.08) | | | | Very Low +○○○ | | |  |
| Insulin Glargine vs. STA-TRE | — | | | — | | — | | low^m^ ++○○ | | 0.75 (0.06, 1.45) | | | | low ++○○ | | |  |
| Gliclazide vs. Omega 3 | — | | | — | | — | | — | | 0.39 (-0.25, 1.02) | | | | Very Low +○○○ | | |  |
| Gliclazide vs. Pioglitazone | — | | | — | | — | | low^g^ ++○○ | | -0.52 (-1.12, 0.07) | | | | low ++○○ | | |  |
| Gliclazide vs. Dapagliflozin | — | | | — | | — | | — | | -0.54 (-1.14, 0.06) | | | | Very Low +○○○ | | |  |
| Gliclazide vs. lpragliflozin | — | | | — | | — | | — | | -0.66 (-1.37, 0.04) | | | | Very Low +○○○ | | |  |
| Gliclazide vs. Empagliflozin | — | | | — | | — | | — | | -0.07 (-0.70, 0.54) | | | | Very Low +○○○ | | |  |
| Gliclazide vs. Tofogliflozin | — | | | — | | — | | — | | -0.23 (-0.84, 0.37) | | | | Very Low +○○○ | | |  |
| Gliclazide vs. Glimepiride | — | | | — | | — | | — | | -0.33 (-0.94, 0.26) | | | | Very Low +○○○ | | |  |
| Gliclazide vs. Sitagliptin | — | | | — | | — | | — | | -0.15 (-0.74, 0.44) | | | | Very Low +○○○ | | |  |
| Gliclazide vs. STA-TRE | — | | | — | | — | | low^g^ ++○○ | | 0.22 (-0.37, 0.80) | | | | low ++○○ | | |  |
| Omega 3 vs. Pioglitazone | — | | | — | | — | | low^g^ ++○○ | | -0.91 (-1.20, -0.61) | | | | low ++○○ | | |  |
| Omega 3 vs. Dapagliflozin | -0.76 (-1.40, -0.16) | | | low^b,c^ ++○○ | | — | | low^i^ ++○○ | | -0.93 (-1.22, -0.64) | | | | low ++○○ | | |  |
| Omega 3 vs. lpragliflozin | — | | | — | | — | | low^m^ ++○○ | | -1.05 (-1.53, -0.58) | | | | low ++○○ | | |  |
| Omega 3 vs. Empagliflozin | — | | | — | | — | | low^l^ ++○○ | | -0.46 (-0.78, -0.14) | | | | low ++○○ | | |  |
| Omega 3 vs. Tofogliflozin | — | | | — | | — | | — | | -0.62 (-0.93, -0.30) | | | | Very Low +○○○ | | |  |
| Omega 3 vs. Glimepiride | — | | | — | | — | | low^m^ ++○○ | | -0.72 (-1.02, -0.42) | | | | low ++○○ | | |  |
| Omega 3 vs. Sitagliptin | — | | | — | | — | | low^m^ ++○○ | | -0.54 (-0.79, -0.29) | | | | low ++○○ | | |  |
| Omega 3 vs. STA-TRE | -0.22(-0.61, 0.17) | | | low^b,c^ ++○○ | | — | | low^i^ ++○○ | | -0.17 (-0.42, 0.07) | | | | low ++○○ | | |  |
| Pioglitazone vs. Dapagliflozin | -0.03(-0.27, 0.22) | | | Moderate^d^ +++○ | | — | | low^i^ ++○○ | | -0.02 (-0.09, 0.05) | | | | Moderate +++○ | | |  |
| Pioglitazone vs. lpragliflozin | 0.26(-0.40, 0.92) | | | low^b,c^ ++○○ | | -0.64(-1.40, 0.16) | | low^m^ ++○○ | | -0.14 (-0.56, 0.27) | | | | low ++○○ | | |  |
| Pioglitazone vs. Empagliflozin | 0.56(-0.03, 1.10) | | | low^b,c^ ++○○ | | 0.48(-0.10, 1.10) | | Very Low^l^ +○○○ | | 0.44 (0.18, 0.71) | | | | low ++○○ | | |  |
| Pioglitazone vs. Tofogliflozin | 0.29(-0.02, 0.59) | | | low^b,c^ ++○○ | | — | | — | | 0.29 (0.18, 0.40) | | | | low ++○○ | | |  |
| Pioglitazone vs. Glimepiride | 0.18(-0.12, 0.48) | | | low^b,c^ ++○○ | | — | | low^g^ ++○○ | | 0.19 (0.10, 0.27) | | | | low ++○○ | | |  |
| Pioglitazone vs. Sitagliptin | — | | | — | | — | | low^m^ ++○○ | | 0.37 (0.16, 0.57) | | | | low ++○○ | | |  |
| Pioglitazone vs. STA-TRE | 0.97(0.43, 1.50) | | | low^b,c^ ++○○ | | 0.68(0.31, 1.00) | | Very Low^l^ +○○○ | | 0.74 (0.53, 0.94) | | | | low ++○○ | | |  |
| Dapagliflozin vs. lpragliflozin | — | | | — | | — | | low^g^ ++○○ | | -0.12 (-0.55, 0.30) | | | | low ++○○ | | |  |
| Dapagliflozin vs. Empagliflozin | — | | | — | | — | | low^g^ ++○○ | | 0.46 (0.20, 0.73) | | | | low ++○○ | | |  |
| Dapagliflozin vs. Tofogliflozin | — | | | — | | — | | low^g^ ++○○ | | 0.31 (0.18, 0.44) | | | | low ++○○ | | |  |
| Dapagliflozin vs. Glimepiride | 0.21(-0.10, 0.51) | | | low^b,c^ ++○○ | | — | | low^g^ ++○○ | | 0.21 (0.13, 0.28) | | | | low ++○○ | | |  |
| Dapagliflozin vs. Sitagliptin | — | | | — | | — | | low^i^ ++○○ | | 0.39 (0.18, 0.59) | | | | low ++○○ | | |  |
| Dapagliflozin vs. STA-TRE | 0.66(0.34, 0.97) | | | High Q | | 1.10(0.44, 1.70) | | low^g^ ++○○ | | 0.75 (0.55, 0.95) | | | | High Q | | |  |
| lpragliflozin vs. Empagliflozin | — | | | — | | — | | low^m^ ++○○ | | 0.59 (0.13, 1.04) | | | | low ++○○ | | |  |
| lpragliflozin vs. Tofogliflozin | — | | | — | | — | | low^m^ ++○○ | | 0.43 (0, 0.87) | | | | low ++○○ | | |  |
| lpragliflozin vs. Glimepiride | — | | | — | | — | | low^m^ ++○○ | | 0.33 (-0.10, 0.75) | | | | low ++○○ | | |  |
| lpragliflozin vs. Sitagliptin | — | | | — | | — | | low^m^ ++○○ | | 0.51 (0.10, 0.93) | | | | low ++○○ | | |  |
| lpragliflozin vs. STA-TRE | 1.20(0.59, 1.80) | | | low^b,c^ ++○○ | | 0.15(-0.78, 1.10) | | low^m^ ++○○ | | 0.88 (0.47, 1.29) | | | | low ++○○ | | |  |
| Empagliflozin vs. Tofogliflozin | — | | | — | | — | | low^m^ ++○○ | | -0.15 (-0.45, 0.13) | | | | low ++○○ | | |  |
| Empagliflozin vs. Glimepiride | — | | | — | | — | | low^m^ ++○○ | | -0.26 (-0.54, 0.02) | | | | low ++○○ | | |  |
| Empagliflozin vs. Sitagliptin | -0.01(-0.39, 0.38) | | | low^b,c^ ++○○ | | -0.57(-1.50, 0.40) | | Very Low^l^ +○○○ | | -0.08 (-0.29, 0.13) | | | | low ++○○ | | |  |
| Empagliflozin vs. STA-TRE | 0.18(-0.26, 0.61) | | | Very Low^a,b,d^ +○○○ | | 0.35(-0.19, 0.89) | | low^m^ ++○○ | | 0.29 (0.08, 0.50) | | | | low ++○○ | | |  |
| Tofogliflozin vs. Glimepiride | — | | | — | | — | | low^m^ ++○○ | | -0.10 (-0.24, 0.04) | | | | low ++○○ | | |  |
| Tofogliflozin vs. Sitagliptin | — | | | — | | — | | — | | 0.08 (-0.16, 0.31) | | | | Very Low +○○○ | | |  |
| Tofogliflozin vs. STA-TRE | — | | | — | | — | | low^m^ ++○○ | | 0.44 (0.21, 0.68) | | | | low ++○○ | | |  |
| Glimepiride vs. Sitagliptin | — | | | — | | — | | — | | 0.18 (-0.03, 0.39) | | | | Very Low +○○○ | | |  |
| Glimepiride vs. STA-TRE | — | | | — | | — | | low^m^ ++○○ | | 0.55 (0.34, 0.76) | | | | low ++○○ | | |  |
| Sitagliptin vs. STA-TRE | 0.37(0.08, 0.66) | | | low^b,c^ ++○○ | | — | | Very Low^l^ +○○○ | | 0.37 (0.33, 0.41) | | | | low ++○○ | | |  |
| I^2^ | | 32.87% | | | |  | | |  | | | 40.35% | | | | | |
| PSRF=1.01 | |  | | | |  | | |  | | |  | | | | | |
|  | |  | | |  |  | | |  | | |  | | |  | | |
| **Outcome: BMI** | |  | | |  |  | | |  | | |  | | |  | | |
| Liraglutide vs. Exenatide | — | | — | | | — | Low^g^ ++○○ | | | | -0.81 (-1.45, -0.18 ) | | Low ++○○ | | |  |  |
| Liraglutide vs. Metformin | 0.88(0.46, 1.50) | | Moderate^b^ +++○ | | | — | Moderate^e^ +++○ | | | | 0.76 (0.55, 0.98) | | Moderate +++○ | | |  |  |
| Liraglutide vs. Insulin Glargine | 1.50(-1.30, 4.30) | | low^b,c^ ++○○ | | | 0.59(-0.75, 1.90) | Low^g^ ++○○ | | | | 0.61 (-0.20, 1.43) | | Low ++○○ | | |  |  |
| Liraglutide vs. Gliclazide | 1.70(1.10, 2.30) | | Moderate^a^ +++○ | | | — | Moderate^e^ +++○ | | | | 1.78 (1.58, 1.98) | | Moderate +++○ | | |  |  |
| Liraglutide vs. Omega 3 | — | | — | | | — | Low^g^ ++○○ | | | | 0.98 (-0.93, 2.88) | | Low ++○○ | | |  |  |
| Liraglutide vs. Pioglitazone | 4.10(1.80, 6.40) | | low^b,c^ ++○○ | | | 1.80(-1.80, 5.40) | — | | | | 3.40 (1.58, 5.20) | | Low ++○○ | | |  |  |
| Liraglutide vs. Dapagliflozin | — | | — | | | — | — | | | | 1.18 (-0.78, 3.16) | | Very Low +○○○ | | |  |  |
| Liraglutide vs. Empagliflozin | — | | — | | | — | Low^m^ ++○○ | | | | 1.43 (-0.64, 3.52) | | Low ++○○ | | |  |  |
| Liraglutide vs. Sitagliptin | — | | — | | | — | — | | | | 2.03 (-0.11, 4.18) | | Very Low +○○○ | | |  |  |
| Liraglutide vs. STA-TRE | — | | — | | | — | Low^m^ ++○○ | | | | 2.07 (0.14, 4.00) | | Low ++○○ | | |  |  |
| Exenatide vs. Metformin | 1.60(0.65, 2.60) | | low^b,c^ ++○○ | | | 1.10(-3.10, 5.30) | Low^m^ ++○○ | | | | 1.58 (0.98, 2.18) | | Low ++○○ | | |  |  |
| Exenatide vs. Insulin Glargine | 1.40(0.46, 2.30) | | low^b,c^ ++○○ | | | 3.00(-3.40, 9.50) | Low^m^ ++○○ | | | | 1.43 (0.84, 2.01) | | Low ++○○ | | |  |  |
| Exenatide vs. Gliclazide | — | | — | | | — | Low^g^ ++○○ | | | | 2.59 (1.95, 3.23) | | Low ++○○ | | |  |  |
| Exenatide vs. Omega 3 | — | | — | | | — | Low^m^ ++○○ | | | | 1.80 (-0.19, 3.78) | | Low ++○○ | | |  |  |
| Exenatide vs. Pioglitazone | — | | — | | | — | — | | | | 4.21 (2.30, 6.11) | | Very Low +○○○ | | |  |  |
| Exenatide vs. Dapagliflozin | — | | — | | | — | — | | | | 2.00 (-0.06, 4.06) | | Very Low +○○○ | | |  |  |
| Exenatide vs. Empagliflozin | — | | — | | | — | — | | | | 2.24 (0.09, 4.42) | | Very Low +○○○ | | |  |  |
| Exenatide vs. Sitagliptin | — | | — | | | — | — | | | | 2.85 (0.63, 5.08) | | Very Low +○○○ | | |  |  |
| Exenatide vs. STA-TRE | — | | — | | | — | Low^m^ ++○○ | | | | 2.88 (0.86, 4.91) | | Low ++○○ | | |  |  |
| Metformin vs. Insulin Glargine | -0.41(-2.80, 1.90) | | low^b,c^ ++○○ | | | -0.07(-1.30,1.20) | Low^g^ ++○○ | | | | -0.15 (-0.94, 0.64) | | Low ++○○ | | |  |  |
| Metformin vs. Gliclazide | 1.10(0.51, 1.70) | | Moderate^a^ +++○ | | | — | Moderate^e^ +++○ | | | | 1.01 (0.79, 1.24) | | Moderate +++○ | | |  |  |
| Metformin vs. Omega 3 | 0.15(-2.10, 2.40) | | low^b,c^ ++○○ | | | 0.36(-4.40, 5.20) | Low^m^ ++○○ | | | | 0.22 (-1.68, 2.11) | | Low ++○○ | | |  |  |
| Metformin vs. Pioglitazone | — | | — | | | — | Low^g^ ++○○ | | | | 2.63 (0.82, 4.44) | | Low ++○○ | | |  |  |
| Metformin vs. Dapagliflozin | — | | — | | | — | Low^m^ ++○○ | | | | 0.42 (-1.55, 2.39) | | Low ++○○ | | |  |  |
| Metformin vs. Empagliflozin | — | | — | | | — | Low^g^ ++○○ | | | | 0.67 (-1.41, 2.75) | | Low ++○○ | | |  |  |
| Metformin vs. Sitagliptin | — | | — | | | — | — | | | | 1.27 (-0.87, 3.42) | | Very Low +○○○ | | |  |  |
| Metformin vs. STA-TRE | -0.26(-3.70, 3.30) | | low^b,c^ ++○○ | | | 1.90(-0.58, 4.40) | Low^m^ ++○○ | | | | 1.30 (-0.63, 3.24) | | Low ++○○ | | |  |  |
| Insulin Glargine vs. Gliclazide | — | | — | | | — | Low^g^ ++○○ | | | | 1.17 (0.35, 1.98) | | Low ++○○ | | |  |  |
| Insulin Glargine vs. Omega 3 | — | | — | | | — | Low^m^ ++○○ | | | | 0.37 (-1.69, 2.40 ) | | Low ++○○ | | |  |  |
| Insulin Glargine vs. Pioglitazone | — | | — | | | — | Low^m^ ++○○ | | | | 2.78 (0.81, 4.76) | | Low ++○○ | | |  |  |
| Insulin Glargine vs. Dapagliflozin | — | | — | | | — | — | | | | 0.57 (-1.54, 2.69) | | Very Low +○○○ | | |  |  |
| Insulin Glargine vs. Empagliflozin | — | | — | | | — | — | | | | 0.82 (-1.40, 3.05) | | Very Low +○○○ | | |  |  |
| Insulin Glargine vs. Sitagliptin | — | | — | | | — | — | | | | 1.42 (-0.86, 3.71) | | Very Low +○○○ | | |  |  |
| Insulin Glargine vs. STA-TRE | — | | — | | | — | Low^m^ ++○○ | | | | 1.46 (-0.62, 3.54) | | Low ++○○ | | |  |  |
| Gliclazide vs. Omega 3 | — | | — | | | — | Low^g^ ++○○ | | | | -0.80 (-2.71, 1.10) | | Low ++○○ | | |  |  |
| Gliclazide vs. Pioglitazone | — | | — | | | — | Low^g^ ++○○ | | | | 1.62 (-0.20, 3.43) | | Low ++○○ | | |  |  |
| Gliclazide vs. Dapagliflozin | — | | — | | | — | — | | | | -0.60 (-2.56, 1.38) | | Very Low +○○○ | | |  |  |
| Gliclazide vs. Empagliflozin | — | | — | | | — | — | | | | -0.34 (-2.43, 1.74) | | Very Low +○○○ | | |  |  |
| Gliclazide vs. Sitagliptin | — | | — | | | — | — | | | | 0.25 (-1.89, 2.40) | | Very Low +○○○ | | |  |  |
| Gliclazide vs. STA-TRE | — | | — | | | — | Low^g^ ++○○ | | | | 0.29 (-1.64, 2.23) | | Low ++○○ | | |  |  |
| Omega 3 vs. Pioglitazone | — | | — | | | — | Low^m^ ++○○ | | | | 2.42 (0.03, 4.80) | | Low ++○○ | | |  |  |
| Omega 3 vs. Dapagliflozin | — | | — | | | — | Low^i^ ++○○ | | | | 0.20 (-2.14, 2.57) | | Low ++○○ | | |  |  |
| Omega 3 vs. Empagliflozin | — | | — | | | — | Low^g^ ++○○ | | | | 0.46 (-2.05, 2.97) | | Low ++○○ | | |  |  |
| Omega 3 vs. Sitagliptin | — | | — | | | — | — | | | | 1.06 (-1.51, 3.62) | | Very Low +○○○ | | |  |  |
| Omega 3 vs. STA-TRE | 0.91(-2.80, 4.60) | | low^b,c^ ++○○ | | | 1.10(-2.20, 4.40) | Low^m^ ++○○ | | | | 1.09 (-1.23, 3.42) | | Low ++○○ | | |  |  |
| Pioglitazone vs. Dapagliflozin | — | | — | | | — | Low^i^ ++○○ | | | | -2.21 (-3.83, -0.59) | | Low ++○○ | | |  |  |
| Pioglitazone vs. Empagliflozin | -1.70(-3.70, 0.34) | | low^b,c^ ++○○ | | | -2.50(-5.60, 0.54) | Low^g^ ++○○ | | | | -1.96 (-3.50, -0.43) | | Low ++○○ | | |  |  |
| Pioglitazone vs. Sitagliptin | — | | — | | | — | Low^m^ ++○○ | | | | -1.36 (-2.98, 0.25) | | Low ++○○ | | |  |  |
| Pioglitazone vs. STA-TRE | -0.75(-3.50, 2.00) | | low^b,c^ ++○○ | | | -1.70(-3.80, 0.48) | Low^g^ ++○○ | | | | -1.33 (-2.90, 0.25) | | Low ++○○ | | |  |  |
| Dapagliflozin vs. Empagliflozin | — | | — | | | — | Moderate^h^ +++○ | | | | 0.25 (-1.11, 1.61) | | Moderate +++○ | | |  |  |
| Dapagliflozin vs. Sitagliptin | — | | — | | | — | — | | | | 0.85 (-0.59, 2.31) | | Very Low +○○○ | | |  |  |
| Dapagliflozin vs. STA-TRE | 0.86(0.13, 1.50) | | High ++++ | | | — | — | | | | 0.88 (0.52, 1.24) | | High ++++ | | |  |  |
| Empagliflozin vs. Sitagliptin | 0.60(-0.29, 1.50) | | low^b,c^ ++○○ | | | — | — | | | | 0.60 (0.08, 1.12) | | Low ++○○ | | |  |  |
| Empagliflozin vs. STA-TRE | 0.78(-0.78, 2.30) | | Moderate^d^ +++○ | | | — | Low^m^ ++○○ | | | | 0.64 (-0.68, 1.94) | | Moderate +++○ | | |  |  |
| Sitagliptin vs. STA-TRE | — | | — | | | — | Low^g^ ++○○ | | | | 0.03 (-1.38, 1.44) | | Low ++○○ | | |  |  |
| I^2^ | | 14.16% | | | |  | | |  | | | 0% | | | | | |
| PSRF=1.03 | |  | | | |  | | |  | | |  | | | | | |
|  | |  | | |  |  | | |  | | |  | | |  | | |
| **Outcome: waist circumference** | |  | | |  |  | | |  | | |  | | |  | | |
|  | |  | | |  |  | | |  | | |  | | |  | | |
| Liraglutide vs. Exenatide | | — | | | — | — | | | Low^m^ ++○○ | | | -1.59(-5.68 to 2.51) | | | Low ++○○ | | |
| Liraglutide vs. Metformin | | 1.90(0.89 to 3.10) | | | Moderate^a^ +++○ | — | | | Moderate^e^ +++○ | | | 1.89(1.27 to 2.50) | | | Moderate +++○ | | |
| Liraglutide vs. Insulin Glargine | | 4.70(-0.81 to 10.00) | | | Low^b,c^ ++○○ | — | | | Low^g^ ++○○ | | | 2.81(-0.66 to 6.27) | | | Low ++○○ | | |
| Liraglutide vs. Gliclazide | | 3.10(2 to 40.1.00) | | | Moderate^a^ +++○ | — | | | Moderate^e^ +++○ | | | 3.09(2.52 to 3.65) | | | Moderate +++○ | | |
| Liraglutide vs. Omega 3 | | — | | | — | — | | | Low^g^ ++○○ | | | 3.02(-0.73 to 6.80) | | | Low ++○○ | | |
| Liraglutide vs. Pioglitazone | | 9.10(5.70 to 12.00) | | | Low^b,c^++○○ | -27.00(-47.00 to -6.40) | | | — | | | 8.10(5.21 to 10.97) | | | Low++○○ | | |
| Liraglutide vs. Dapagliflozine | | — | | | — | — | | | Low^m^ ++○○ | | | 1.46(-2.34 to 5.30) | | | Low ++○○ | | |
| Liraglutide vs. STA-TRE | | — | | | — | — | | | — | | | 3.52(-0.39 to 7.45) | | | Very Low +○○○ | | |
| Exenatide vs. Metformin | | — | | | — | — | | | Low^m^ ++○○ | | | 3.47(-0.62 to 7.56) | | | Low ++○○ | | |
| Exenatide vs. Insulin Glargine | | 4.40(1.90 to 6.90) | | | Low^b,c^ ++○○ | NA( to ) | | | — | | | 4.40(2.20 to 6.59) | | | Low ++○○ | | |
| Exenatide vs. Gliclazide | | — | | | — | — | | | — | | | 4.68(0.55 to 8.80) | | | Very Low +○○○ | | |
| Exenatide vs. Omega 3 | | — | | | — | — | | | — | | | 4.6(-0.92 to 10.14) | | | Very Low +○○○ | | |
| Exenatide vs. Pioglitazone | | — | | | — | — | | | — | | | 9.68(4.70 to 14.69) | | | Very Low +○○○ | | |
| Exenatide vs. Dapagliflozine | | — | | | — | — | | | — | | | 3.06(-2.53 to 8.66) | | | Very Low +○○○ | | |
| Exenatide vs. STA-TRE | | — | | | — | — | | | — | | | 5.11(-0.52 to 10.79) | | | Very Low +○○○ | | |
| Metformin vs. Insulin Glargine | | -0.37(-5.00 to 4.30) | | | Low^b,c^ ++○○ | — | | | Low^g^ ++○○ | | | 0.93(-2.53 to 4.37) | | | Low ++○○ | | |
| Metformin vs. Gliclazide | | 1.30(0.05 to 2.50) | | | Moderate^a^ +++○ | — | | | Moderate^e^ +++○ | | | 1.20(0.54 to 1.86) | | | Moderate +++○ | | |
| Metformin vs. Omega 3 | | -1.20(-6.20 to 3.70) | | | Low^b,c^ ++○○ | 7.10(-1.30 to 15.00) | | |  | | | 1.13(-2.60 to 4.90) | | | Low ++○○ | | |
| Metformin vs. Pioglitazone | | — | | | — | — | | | Low^g^ ++○○ | | | 6.21(3.29 to 9.12) | | | Low ++○○ | | |
| Metformin vs. Dapagliflozine | | — | | | — | — | | | Low^m^ ++○○ | | | -0.42(-4.21 to 3.40) | | | Low ++○○ | | |
| Metformin vs. STA-TRE | | — | | | — | — | | | Low^m^ ++○○ | | | 1.64(-2.27 to 5.57) | | | Low ++○○ | | |
| Insulin Glargine vs. Gliclazide | | — | | | — | — | | | Low^g^ ++○○ | | | 0.28(-3.20 to 3.77) | | | Low ++○○ | | |
| Insulin Glargine vs. Omega 3 | | — | | | — | — | | | Low^m^++○○ | | | 0.20(-4.86 to 5.29) | | | Low++○○ | | |
| Insulin Glargine vs. Pioglitazone | | — | | | — | — | | | Low^m^ ++○○ | | | 5.29(0.83 to 9.79) | | | Low ++○○ | | |
| Insulin Glargine vs. Dapagliflozine | | — | | | — | — | | | — | | | -1.35(-6.47 to 3.79) | | | Very Low +○○○ | | |
| Insulin Glargine vs. STA-TRE | | — | | | — | — | | | — | | | 0.71(-4.48 to 5.93) | | | Very Low +○○○ | | |
| Gliclazide vs. Omega 3 | | — | | | — | — | | | Low^g^ ++○○ | | | -0.07(-3.83 to 3.73) | | | Low ++○○ | | |
| Gliclazide vs. Pioglitazone | | — | | | — | — | | | Low^g^ ++○○ | | | 5.01(2.09 to 7.93) | | | Low ++○○ | | |
| Gliclazide vs. Dapagliflozine | | — | | | — | — | | | — | | | -1.61(-5.44 to 2.22) | | | Very Low +○○○ | | |
| Gliclazide vs. STA-TRE | | — | | | — | — | | | — | | | 0.44(-3.50 to 4.39) | | | Very Low +○○○ | | |
| Omega 3 vs. Pioglitazone | | — | | | — | — | | | Low^m^ ++○○ | | | 5.09(1.31 to 8.87) | | | Low ++○○ | | |
| Omega 3 vs. Dapagliflozine | | -2.30(-4.80 to -0.14) | | | Low^b,c^ ++○○ | 9.20(0.46 to 18.00) | | | Low^g^ ++○○ | | | -1.55(-3.17 to 0.07) | | | Low ++○○ | | |
| Omega 3 vs. STA-TRE | | 0.84(-1.70 to 3.30) | | | Low^b,c^ ++○○ | -2.20(-11.00 to 6.40) | | | Low^g^ ++○○ | | | 0.51(-1.16 to 2.19) | | | Low ++○○ | | |
| Pioglitazone vs. Dapagliflozine | | -4.40(-9.20 to 0.40) | | | Low^b,c^ ++○○ | -13.00(-22.00 to -4.50) | | | — | | | -6.64(-10.35 to -2.92) | | | Low ++○○ | | |
| Pioglitazone vs. STA-TRE | | — | | | — | — | | | Low^g^ ++○○ | | | -4.58(-8.44 to -0.71) | | | Low ++○○ | | |
| Dapagliflozine vs. STA-TRE | | 1.90(0.19 to 3.80) | | | Moderate^a^ +++○ | — | | | Low^m^ ++○○ | | | 2.06(0.68 to 3.43) | | | Moderate +++○ | | |
| I^2^ | | 0% | | | |  | | |  | | | 0% | | | | | |
|  | |  | | |  |  | | |  | | |  | | |  | | |
| PSRF=1.00 | |  | | |  |  | | |  | | |  | | |  | | |
| **Outcome: ALT** | |  | | |  |  | | |  | | |  | | |  | | |
|  | |  | | |  |  | | |  | | |  | | |  | | |
| Liraglutide vs. Exenatide | | — | | | — | — | | | Very Lowl +○○○ | | | -10.96 (-16.66, -5.27 ) | | | Very Low +○○○ | | |
| Liraglutide vs. Metformin | | 2.80(-1.40, 8.60) | | | Very Lowo,d +○○○ | 2.60(-9.40, 15.00) | | | lowm ++○○ | | | 1.11 (-0.58, 2.81) | | | low ++○○ | | |
| Liraglutide vs. Insulin Glargine | | 4.10(-8.00, 16.00) | | | lowb,c ++○○ | 2.00(-9.80, 14.00) | | | Very Lowl +○○○ | | | 1.80 (-3.52, 7.11) | | | low ++○○ | | |
| Liraglutide vs. Gliclazide | | 11.00(4.90, 18.00) | | | Moderatea +++○ | — | | | Very Lowf +○○○ | | | 12.03 (10.3, 13.77) | | | Moderate +++○ | | |
| Liraglutide vs. Omega 3 | | — | | | — | — | | | — | | | 15.99 (-8.76, 40.76) | | | Very Low +○○○ | | |
| Liraglutide vs. Pioglitazone | | 9.90(1.10,19.00) | | | lowb,c ++○○ | — | | | — | | | 9.87 (9.12, 10.62) | | | low ++○○ | | |
| Liraglutide vs. Dapagliflozin | | — | | | — | — | | | lowg ++○○ | | | 11.77 (9.38, 14.17) | | | low ++○○ | | |
| Liraglutide vs. lpragliflozin | | — | | | — | — | | | lowm ++○○ | | | 3.50 (-6.53, 13.51) | | | low ++○○ | | |
| Liraglutide vs. Empagliflozin | | — | | | — | — | | | lowm ++○○ | | | 10.03 (3.37, 16.64) | | | low ++○○ | | |
| Liraglutide vs. Tofogliflozin | | — | | | — | — | | | lowm ++○○ | | | 20.32 (2.40, 38.34) | | | low ++○○ | | |
| Liraglutide vs. Glimepiride | | — | | | — | — | | | lowm ++○○ | | | 23.8 (21.48, 26.12) | | | low ++○○ | | |
| Liraglutide vs. Sitagliptin | | — | | | — | — | | | — | | | 13.18 (-3.27, 29.83) | | | Very Low +○○○ | | |
| Liraglutide vs. STA-TRE | | — | | | — | — | | | lowm ++○○ | | | 12.08 (6.43, 17.82) | | | low ++○○ | | |
| Exenatide vs. Metformin | | 12.00(1.10, 22.00) | | | lowb,c ++○○ | 14.00(-0.45, 28.00) | | | lowm ++○○ | | | 12.07 (6.58, 17.56) | | | low ++○○ | | |
| Exenatide vs. Insulin Glargine | | 14.00(1.30, 26.00) | | | lowb,c ++○○ | 11.00(-2.40, 25.00) | | | lowm ++○○ | | | 12.77 (6.43, 19.14) | | | low ++○○ | | |
| Exenatide vs. Gliclazide | | — | | | — | — | | | lowg ++○○ | | | 23.00 (17.26, 28.74) | | | low ++○○ | | |
| Exenatide vs. Omega 3 | | — | | | — | — | | | — | | | 27.00 (1.52, 52.39) | | | Very Low +○○○ | | |
| Exenatide vs. Pioglitazone | | — | | | — | — | | | — | | | 20.83 (15.08, 26.56) | | | Very Low +○○○ | | |
| Exenatide vs. Dapagliflozin | | — | | | — | — | | | — | | | 22.73 (16.57, 28.88) | | | Very Low +○○○ | | |
| Exenatide vs. lpragliflozin | | — | | | — | — | | | — | | | 14.45 (2.95, 25.96) | | | Very Low +○○○ | | |
| Exenatide vs. Empagliflozin | | — | | | — | — | | | — | | | 20.99 (12.30, 29.73) | | | Very Low +○○○ | | |
| Exenatide vs. Tofogliflozin | | — | | | — | — | | | — | | | 31.3 (12.48, 50.12) | | | Very Low +○○○ | | |
| Exenatide vs. Glimepiride | | — | | | — | — | | | — | | | 34.76 (28.59, 40.89) | | | Very Low +○○○ | | |
| Exenatide vs. Sitagliptin | | — | | | — | — | | | — | | | 24.15 (6.73, 41.7) | | | Very Low +○○○ | | |
| Exenatide vs. STA-TRE | | — | | | — | — | | | lowm ++○○ | | | 23.03 (15.12, 30.96) | | | low ++○○ | | |
| Metformin vs. Insulin Glargine | | -2.10(-14.00, 9.50) | | | lowb,c ++○○ | 2.20(-9.00, 13.00) | | | Very Lowm +○○○ | | | 0.68 (-4.59, 5.96) | | | low ++○○ | | |
| Metformin vs. Gliclazide | | 12.00(5.20, 18.00) | | | Moderatea +++○ | — | | | Very Lowl +○○○ | | | 10.92 (9.17, 12.65) | | | Moderate +++○ | | |
| Metformin vs. Omega 3 | | — | | | — | — | | | lowm ++○○ | | | 14.88 (-9.90, 39.68) | | | low ++○○ | | |
| Metformin vs. Pioglitazone | | — | | | — | — | | | Very Lowl +○○○ | | | 8.75 (6.90, 10.61) | | | Very Low +○○○ | | |
| Metformin vs. Dapagliflozin | | — | | | — | — | | | — | | | 10.65 (7.72, 13.60) | | | Very Low +○○○ | | |
| Metformin vs. lpragliflozin | | — | | | — | — | | | lowm ++○○ | | | 2.38 (-7.75, 12.52) | | | low ++○○ | | |
| Metformin vs. Empagliflozin | | — | | | — | — | | | lowm ++○○ | | | 8.91 (2.09, 15.66) | | | low ++○○ | | |
| Metformin vs. Tofogliflozin | | — | | | — | — | | | — | | | 19.21 (1.22, 37.28) | | | Very Low +○○○ | | |
| Metformin vs. Glimepiride | | — | | | — | — | | | — | | | 22.69 (19.80, 25.55) | | | Very Low +○○○ | | |
| Metformin vs. Sitagliptin | | — | | | — | — | | | — | | | 12.07 (-4.44, 28.75) | | | Very Low +○○○ | | |
| Metformin vs. STA-TRE | | 9.20(-3.20, 22.00) | | | lowb,c ++○○ | — | | | — | | | 10.95 (5.24, 16.74) | | | low ++○○ | | |
| Insulin Glargine vs. Gliclazide | | — | | | — | — | | | lowg ++○○ | | | 10.23 (4.77, 15.67) | | | low ++○○ | | |
| Insulin Glargine vs. Omega 3 | | — | | | — | — | | | — | | | 14.22 (-11.14, 39.47) | | | Very Low +○○○ | | |
| Insulin Glargine vs. Pioglitazone | | — | | | — | — | | | lowm ++○○ | | | 8.07 (2.69, 13.43) | | | low ++○○ | | |
| Insulin Glargine vs. Dapagliflozin | | — | | | — | — | | | — | | | 9.97 (4.11, 15.80) | | | Very Low +○○○ | | |
| Insulin Glargine vs. lpragliflozin | | — | | | — | — | | | — | | | 1.70 (-9.70, 13.05) | | | Very Low +○○○ | | |
| Insulin Glargine vs. Empagliflozin | | — | | | — | — | | | — | | | 8.21 (-0.24, 16.72) | | | Very Low +○○○ | | |
| Insulin Glargine vs. Tofogliflozin | | — | | | — | — | | | — | | | 18.49 (-0.19, 37.26) | | | Very Low +○○○ | | |
| Insulin Glargine vs. Glimepiride | | — | | | — | — | | | — | | | 22.00 (16.16, 27.79) | | | Very Low +○○○ | | |
| Insulin Glargine vs. Sitagliptin | | — | | | — | — | | | — | | | 11.37 (-5.92, 28.84) | | | Very Low +○○○ | | |
| Insulin Glargine vs. STA-TRE | | — | | | — | — | | | lowm ++○○ | | | 10.29 (2.56, 18.01) | | | low ++○○ | | |
| Gliclazide vs. Omega 3 | | — | | | — | — | | | — | | | 3.99 (-20.79, 28.81) | | | Very Low +○○○ | | |
| Gliclazide vs. Pioglitazone | | — | | | — | — | | | lowg ++○○ | | | -2.16 (-4.05, -0.28) | | | low ++○○ | | |
| Gliclazide vs. Dapagliflozin | | — | | | — | — | | | — | | | -0.27 (-3.22, 2.70) | | | Very Low +○○○ | | |
| Gliclazide vs. lpragliflozin | | — | | | — | — | | | — | | | -8.55 (-18.70, 1.64) | | | Very Low +○○○ | | |
| Gliclazide vs. Empagliflozin | | — | | | — | — | | | — | | | -2.01 (-8.84, 4.81) | | | Very Low +○○○ | | |
| Gliclazide vs. Tofogliflozin | | — | | | — | — | | | — | | | 8.28 (-9.74, 26.39) | | | Very Low +○○○ | | |
| Gliclazide vs. Glimepiride | | — | | | — | — | | | — | | | 11.78 (8.86, 14.65) | | | Very Low +○○○ | | |
| Gliclazide vs. Sitagliptin | | — | | | — | — | | | — | | | 1.15 (-15.38, 17.84) | | | Very Low +○○○ | | |
| Gliclazide vs. STA-TRE | | — | | | — | — | | | lowg ++○○ | | | 0.04 (-5.78, 5.95) | | | low ++○○ | | |
| Omega 3 vs. Pioglitazone | | — | | | — | — | | | lowm ++○○ | | | -6.14 (-30.89, 18.62) | | | low ++○○ | | |
| Omega 3 vs. Dapagliflozin | | — | | | — | — | | | — | | | -4.24 (-29.16, 20.62) | | | Very Low +○○○ | | |
| Omega 3 vs. lpragliflozin | | — | | | — | — | | | lowm ++○○ | | | -12.52 (-38.86, 13.80) | | | low ++○○ | | |
| Omega 3 vs. Empagliflozin | | — | | | — | — | | | lowm ++○○ | | | -6.03 (-30.98, 19.06) | | | low ++○○ | | |
| Omega 3 vs. Tofogliflozin | | — | | | — | — | | | — | | | 4.27 (-26.22, 34.88) | | | Very Low +○○○ | | |
| Omega 3 vs. Glimepiride | | — | | | — | — | | | — | | | 7.77 (-17.13, 32.56) | | | Very Low +○○○ | | |
| Omega 3 vs. Sitagliptin | | — | | | — | — | | | — | | | -2.86 (-31.97, 26.39) | | | Very Low +○○○ | | |
| Omega 3 vs. STA-TRE | | -3.90(-30.00, 22.00) | | | lowb,c ++○○ | — | | | — | | | -3.93 (-28.02, 20.22) | | | low ++○○ | | |
| Pioglitazone vs. Dapagliflozin | | 1.40(-5.80, 8.20) | | | Moderated +++○ | — | | | lowm ++○○ | | | 1.90 (-0.37, 4.18) | | | Moderate +++○ | | |
| Pioglitazone vs. lpragliflozin | | -2.90(-17.00,11.00) | | | lowb,c ++○○ | -14.00(-35.00, 7.00) | | | lowm ++○○ | | | -6.37 (-16.39, 3.62) | | | low ++○○ | | |
| Pioglitazone vs. Empagliflozin | | 0.53(-11.00, 12.00) | | | lowb,c ++○○ | -0.10(-17.00, 17.00) | | | lowm ++○○ | | | 0.16 (-6.48, 6.75) | | | low ++○○ | | |
| Pioglitazone vs. Tofogliflozin | | 10.00(-9.40, 30.00) | | | lowb,c ++○○ | — | | | — | | | 10.46 (-7.46, 28.46) | | | low ++○○ | | |
| Pioglitazone vs. Glimepiride | | 14.00(4.90, 23.00) | | | lowb,c ++○○ | — | | | lowg ++○○ | | | 13.93 (11.73, 16.13) | | | low ++○○ | | |
| Pioglitazone vs. Sitagliptin | | — | | | — | — | | | lowm ++○○ | | | 3.30 (-13.13, 19.94) | | | low ++○○ | | |
| Pioglitazone vs. STA-TRE | | 1.10(-12.00, 14.00) | | | lowb,c ++○○ | 5.10(-6.00, 16.00) | | | lowm ++○○ | | | 2.20 (-3.43, 7.94) | | | low ++○○ | | |
| Dapagliflozin vs. lpragliflozin | | — | | | — | — | | | lowg ++○○ | | | -8.27 (-18.59, 2.00) | | | low ++○○ | | |
| Dapagliflozin vs. Empagliflozin | | — | | | — | — | | | lowg ++○○ | | | -1.73 (-8.77, 5.20) | | | low ++○○ | | |
| Dapagliflozin vs. Tofogliflozin | | — | | | — | — | | | lowg ++○○ | | | 8.55 (-9.46, 26.69) | | | low ++○○ | | |
| Dapagliflozin vs. Glimepiride | | 12.00(2.90, 21.00) | | | lowb,c ++○○ | — | | | lowg ++○○ | | | 12.03 (10.00, 14.08) | | | low ++○○ | | |
| Dapagliflozin vs. Sitagliptin | | — | | | — | — | | | — | | | 1.40 (-15.21, 18.22) | | | Very Low +○○○ | | |
| Dapagliflozin vs. STA-TRE | | — | | | — | — | | | lowg ++○○ | | | 0.30 (-5.81, 6.49) | | | low ++○○ | | |
| lpragliflozin vs. Empagliflozin | | — | | | — | — | | | lowm ++○○ | | | 6.53 (-5.16, 18.18) | | | low ++○○ | | |
| lpragliflozin vs. Tofogliflozin | | — | | | — | — | | | lowm ++○○ | | | 16.81 (-3.70, 37.32) | | | low ++○○ | | |
| lpragliflozin vs. Glimepiride | | — | | | — | — | | | lowm ++○○ | | | 20.31 (10.05, 30.57) | | | low ++○○ | | |
| lpragliflozin vs. Sitagliptin | | — | | | — | — | | | — | | | 9.73 (-9.41, 28.70) | | | Very Low +○○○ | | |
| lpragliflozin vs. STA-TRE | | 18.00(-3.10, 39.00) | | | lowb,c ++○○ | 5.00(-11.00, 21.00) | | | lowm ++○○ | | | 8.58 (-2.13, 19.34) | | | low ++○○ | | |
| Empagliflozin vs. Tofogliflozin | | — | | | — | — | | | lowm ++○○ | | | 10.31 (-8.77, 29.48) | | | low ++○○ | | |
| Empagliflozin vs. Glimepiride | | — | | | — | — | | | lowm ++○○ | | | 13.77 (6.84, 20.78) | | | low ++○○ | | |
| Empagliflozin vs. Sitagliptin | | 3.00(-14.00, 20.00) | | | lowb,c ++○○ | — | | | — | | | 3.17 (-11.90, 18.39) | | | low ++○○ | | |
| Empagliflozin vs. STA-TRE | | 3.10(-7.70, 14.00) | | | lowa,d ++○○ | — | | | lowm ++○○ | | | 2.06 (-4.70, 8.81) | | | low ++○○ | | |
| Tofogliflozin vs. Glimepiride | | — | | | — | — | | | lowm ++○○ | | | 3.48 (-14.66, 21.50) | | | low ++○○ | | |
| Tofogliflozin vs. Sitagliptin | | — | | | — | — | | | — | | | -7.17 (-31.62, 17.32) | | | Very Low +○○○ | | |
| Tofogliflozin vs. STA-TRE | | — | | | — | — | | | lowm ++○○ | | | -8.24 (-27.13, 10.50) | | | low ++○○ | | |
| Glimepiride vs. Sitagliptin | | — | | | — | — | | | — | | | -10.62 (-27.21, 6.20) | | | Very Low +○○○ | | |
| Glimepiride vs. STA-TRE | | — | | | — | — | | | lowm ++○○ | | | -11.72 (-17.82, -5.57) | | | low ++○○ | | |
| Sitagliptin vs. STA-TRE | | — | | | — | — | | | lowm ++○○ | | | -1.12 (-17.76, 15.39) | | | low ++○○ | | |
| I^2^ | | 59.64% | | | |  | | |  | | | 39.69% | | | | | |
| PSRF=1.04 | |  | | |  |  | | |  | | |  | | |  | | |
|  | |  | | |  |  | | |  | | |  | | |  | | |
| **Outcome: insulin resistance** | |  | | |  |  | | |  | | |  | | |  | | |
|  | |  | | |  |  | | |  | | |  | | |  | | |
| Liraglutide vs. Exenatide | | — | | | — | — | | | Very Lowl +○○○ | | | 0.08 (-0.18, 0.34) | | | Very Low +○○○ | | |
| Liraglutide vs. Metformin | | 0.77(-4.20, 6.10) | | | Very Lowo,d +○○○ | — | | | lowm ++○○ | | | 0.20 (0.02, 0.38) | | | low ++○○ | | |
| Liraglutide vs. Insulin Glargine | | 1.90(-5.40, 9.10) | | | lowb,c ++○○ | — | | | Very Lowl +○○○ | | | 0.97 (0.37, 1.56) | | | low ++○○ | | |
| Liraglutide vs. Omega 3 | | — | | | — | — | | | — | | | 2.67 (0.52, 4.83) | | | Very Low +○○○ | | |
| Liraglutide vs. Pioglitazone | | 1.00(-6.40, 8.40) | | | lowb,c ++○○ | — | | | — | | | 1.01 (-0.69, 2.69) | | | low ++○○ | | |
| Liraglutide vs. Dapagliflozin | | — | | | — | — | | | lowm ++○○ | | | 1.04 (-0.69, 2.75) | | | low ++○○ | | |
| Liraglutide vs. lpragliflozin | | — | | | — | — | | | lowm ++○○ | | | 0.68 (-1.57, 2.88) | | | low ++○○ | | |
| Liraglutide vs. Empagliflozin | | — | | | — | — | | | lowm ++○○ | | | 2.18 (-0.1, 4.44) | | | low ++○○ | | |
| Liraglutide vs. Glimepiride | | — | | | — | — | | | lowm ++○○ | | | 1.83 (0.11, 3.54) | | | low ++○○ | | |
| Liraglutide vs. STA-TRE | | — | | | — | — | | | lowm ++○○ | | | 2.04 (0.17, 3.88) | | | low ++○○ | | |
| Exenatide vs. Metformin | | 0.13(-7.10, 7.40) | | | lowb,c ++○○ | — | | | — | | | 0.12 (-0.07, 0.31) | | | low ++○○ | | |
| Exenatide vs. Insulin Glargine | | — | | | — | — | | | lowm ++○○ | | | 0.89 (0.27, 1.50) | | | low ++○○ | | |
| Exenatide vs. Omega 3 | | — | | | — | — | | | — | | | 2.59 (0.42, 4.76) | | | Very Low +○○○ | | |
| Exenatide vs. Pioglitazone | | — | | | — | — | | | — | | | 0.93 (-0.79, 2.63) | | | Very Low +○○○ | | |
| Exenatide vs. Dapagliflozin | | — | | | — | — | | | — | | | 0.96 (-0.79, 2.70) | | | Very Low +○○○ | | |
| Exenatide vs. lpragliflozin | | — | | | — | — | | | — | | | 0.60 (-1.66, 2.82) | | | Very Low +○○○ | | |
| Exenatide vs. Empagliflozin | | — | | | — | — | | | — | | | 2.10 (-0.20, 4.38) | | | Very Low +○○○ | | |
| Exenatide vs. Glimepiride | | — | | | — | — | | | — | | | 1.75 (0.01, 3.48) | | | Very Low +○○○ | | |
| Exenatide vs. STA-TRE | | — | | | — | — | | | — | | | 1.96 (0.08, 3.82) | | | Very Low +○○○ | | |
| Metformin vs. Insulin Glargine | | 0.30(-6.90, 7.60) | | | lowb,c ++○○ | — | | | Very Lowl +○○○ | | | 0.77 (0.18, 1.35) | | | low ++○○ | | |
| Metformin vs. Omega 3 | | — | | | — | — | | | — | | | 2.47 (0.30, 4.64) | | | Very Low +○○○ | | |
| Metformin vs. Pioglitazone | | — | | | — | — | | | Very Lowl +○○○ | | | 0.81 (-0.90, 2.50) | | | Very Low +○○○ | | |
| Metformin vs. Dapagliflozin | | — | | | — | — | | | — | | | 0.84 (-0.90, 2.56) | | | Very Low +○○○ | | |
| Metformin vs. lpragliflozin | | — | | | — | — | | | — | | | 0.48 (-1.78, 2.70) | | | Very Low +○○○ | | |
| Metformin vs. Empagliflozin | | — | | | — | — | | | — | | | 1.98 (-0.31, 4.26) | | | Very Low +○○○ | | |
| Metformin vs. Glimepiride | | — | | | — | — | | | — | | | 1.63 (-0.10, 3.34) | | | Very Low +○○○ | | |
| Metformin vs. STA-TRE | | — | | | — | — | | | — | | | 1.84 (-0.03, 3.69) | | | Very Low +○○○ | | |
| Insulin Glargine vs. Omega 3 | | — | | | — | — | | | — | | | 1.71 (-0.53, 3.95) | | | Very Low +○○○ | | |
| Insulin Glargine vs. Pioglitazone | | — | | | — | — | | | lowm ++○○ | | | 0.04 (-1.75, 1.83) | | | low ++○○ | | |
| Insulin Glargine vs. Dapagliflozin | | — | | | — | — | | | — | | | 0.07 (-1.76, 1.89) | | | Very Low +○○○ | | |
| Insulin Glargine vs. lpragliflozin | | — | | | — | — | | | — | | | -0.29 (-2.61, 1.99) | | | Very Low +○○○ | | |
| Insulin Glargine vs. Empagliflozin | | — | | | — | — | | | — | | | 1.21 (-1.14, 3.56) | | | Very Low +○○○ | | |
| Insulin Glargine vs. Glimepiride | | — | | | — | — | | | — | | | 0.86 (-0.95, 2.68) | | | Very Low +○○○ | | |
| Insulin Glargine vs. STA-TRE | | — | | | — | — | | | — | | | 1.07 (-0.88, 3.00) | | | Very Low +○○○ | | |
| Omega 3 vs. Pioglitazone | | — | | | — | — | | | lowm ++○○ | | | -1.67 (-3.02, -0.32) | | | low ++○○ | | |
| Omega 3 vs. Dapagliflozin | | -1.40(-8.80, 6.10) | | | lowb,c ++○○ | -2.40(-7.70, 2.90) | | | Very Lowl +○○○ | | | -1.64 (-2.97, -0.32) | | | low ++○○ | | |
| Omega 3 vs. lpragliflozin | | — | | | — | — | | | Very Lowl +○○○ | | | -2.00 (-3.91, -0.11) | | | Very Low +○○○ | | |
| Omega 3 vs. Empagliflozin | | — | | | — | — | | | Very Lowl +○○○ | | | -0.50 (-2.48, 1.48) | | | Very Low +○○○ | | |
| Omega 3 vs. Glimepiride | | — | | | — | — | | | lowm ++○○ | | | -0.85 (-2.19, 0.49) | | | low ++○○ | | |
| Omega 3 vs. STA-TRE | | -1.80(-8.70, 3.10) | | | Very Lowa,b +○○○ | — | | | lowm ++○○ | | | -0.64 (-1.96, 0.68) | | | low ++○○ | | |
| Pioglitazone vs. Dapagliflozin | | 0.01(-7.20, 7.20) | | | lowb,c ++○○ | 0.42(-3.90, 4.70) | | | lowm ++○○ | | | 0.03 (-0.30, 0.37) | | | low ++○○ | | |
| Pioglitazone vs. lpragliflozin | | 0.91(-6.60, 8.30) | | | lowb,c ++○○ | -0.68(-5.20, 3.80) | | | lowm ++○○ | | | -0.33 (-1.78, 1.11) | | | low ++○○ | | |
| Pioglitazone vs. Empagliflozin | | 1.10(-6.20, 8.50) | | | lowb,c ++○○ | — | | | lowm ++○○ | | | 1.17 (-0.34, 2.68) | | | low ++○○ | | |
| Pioglitazone vs. Glimepiride | | 0.81(-6.40, 8.00) | | | lowb,c ++○○ | — | | | lowm ++○○ | | | 0.82 (0.53, 1.12) | | | low ++○○ | | |
| Pioglitazone vs. STA-TRE | | 0.80(-6.70, 8.20) | | | lowb,c ++○○ | 1.30(-2.50, 5.20) | | | lowm ++○○ | | | 1.03 (0.28, 1.78) | | | low ++○○ | | |
| Dapagliflozin vs. lpragliflozin | | — | | | — | — | | | lowm ++○○ | | | -0.36 (-1.83, 1.10) | | | low ++○○ | | |
| Dapagliflozin vs. Empagliflozin | | — | | | — | — | | | lowm ++○○ | | | 1.14 (-0.40, 2.67) | | | low ++○○ | | |
| Dapagliflozin vs. Glimepiride | | 0.80(-6.50, 8.10) | | | lowb,c ++○○ | — | | | lowm ++○○ | | | 0.79 (0.54, 1.04) | | | low ++○○ | | |
| Dapagliflozin vs. STA-TRE | | 0.90(-6.30, 8.20) | | | lowb,c ++○○ | 0.90(-3.30, 5.10) | | | lowm ++○○ | | | 1.00 (0.27, 1.73) | | | low ++○○ | | |
| lpragliflozin vs. Empagliflozin | | — | | | — | — | | | lowm ++○○ | | | 1.51 ( -0.54, 3.54) | | | low ++○○ | | |
| lpragliflozin vs. Glimepiride | | — | | | — | — | | | lowm ++○○ | | | 1.15 (-0.30, 2.62) | | | low ++○○ | | |
| lpragliflozin vs. STA-TRE | | 2.70(-4.80, 10.00) | | | lowb,c ++○○ | 1.10(-3.40, 5.60) | | | lowm ++○○ | | | 1.36 (-0.09, 2.82) | | | low ++○○ | | |
| Empagliflozin vs. Glimepiride | | — | | | — | — | | | lowm ++○○ | | | -0.35 (-1.88, 1.19) | | | low ++○○ | | |
| Empagliflozin vs. STA-TRE | | -0.29(-8.00, 7.30) | | | lowb,c ++○○ | — | | | lowm ++○○ | | | -0.15 (-1.71, 1.44) | | | low ++○○ | | |
| Glimepiride vs. STA-TRE | | — | | | — | — | | | lowm ++○○ | | | 0.21 (-0.54, 0.96) | | | low ++○○ | | |
| I^2^ | | 96.22% | | | |  | | |  | | | 38.95% | | | | | |
| PSRF=1.02 | |  | | |  |  | | |  | | |  | | |  | | |
| **Outcome: VATA** | |  | | |  |  | | |  | | |  | | |  | | |
|  | |  | | |  |  | | |  | | |  | | |  | | |
| Liraglutide vs. Exenatide | | — | | | — | — | | | low^m^ ++○○ | | | -4.27(-33.93 to 25.43） | | | low ++○○ | | |
| Liraglutide vs. Metformin | | 44.00(29.00 to 59.00) | | | low^b,c^ ++○○ | — | | | low^m^ ++○○ | | | 43.90(31.18 to 56.51) | | | low ++○○ | | |
| Liraglutide vs. Insulin Glargine | | 31.00 ( 13.00 to 49.00) | | | low^b,c^ ++○○ | — | | | low^m^ ++○○ | | | 31.01(16.11 to 45.79) | | | low ++○○ | | |
| Exenatide vs. Metformin | | — | | | — | — | | | low^m^++○○ | | | 48.13(19.24 to 77.03) | | | low++○○ | | |
| Exenatide vs. Insulin Glargine | | 35.00(14.00 to 56) | | | low^b,c^ ++○○ | — | | | — | | | 35.27(9.52 to 61.17) | | | low ++○○ | | |
| Metformin vs. Insulin Glargine | | -13.00(-28.00 to 2.30） | | | low^b,c^ ++○○ | — | | | low^m^ ++○○ | | | -12.87(-25.93 to 0.18） | | | low ++○○ | | |
| I^2^ | | 100% | | | |  | | |  | | | 0% | | | | | |
| PSRF=1.01 | |  | | |  |  | | |  | | |  | | |  | | |
| **Outcome: SATA** | |  | | |  |  | | |  | | |  | | |  | | |
| Liraglutide vs. Exenatide | | — | | | — | — | | | low^m^ ++○○ | | | -17.61  (-62.50 to 27.34) | | | low ++○○ | | |
| Liraglutide vs. Metformin | | 29.00(-10.00 to 68.00) | | | low^b,c^ ++○○ | — | | | low^m^ ++○○ | | | 29.41(-9.73 to 68.16) | | | low ++○○ | | |
| Liraglutide vs. Insulin Glargine | | 13.00(-23.00 to 50.00) | | | low^b,c^ ++○○ | — | | | low^m^ ++○○ | | | 13.27(-23.95 to 50.61) | | | low ++○○ | | |
| Exenatide vs. Metformin | | — | | | — | — | | | low^m^ ++○○ | | | 46.91(2.82 to 91.21) | | | low ++○○ | | |
| Exenatide vs. Insulin Glargine | | 31.00(6.60 to 56.00) | | | low^b,c^ ++○○ | — | | | — | | | 30.88(6.13 to 55.74) | | | low ++○○ | | |
| Metformin vs. Insulin Glargine | | -16.00(-51.00 to 19.00) | | | low^b,c^ ++○○ | — | | | low^m^ ++○○ | | | -16.06  (-52.54 to 20.48) | | | low ++○○ | | |
| I^2^ | | 100% | | | |  | | | 0% | | | | | |  | | |
| PSRF=1.03 | |  | | | |  | | |  | | | | | |  | | |
| Notes: a, Risk of bias; b, Inconsistency; c, Indirectness; d, Imprecision; e, Contributing direct evidence of moderate quality; f, Contributing direct evidence of moderate or very low quality; g, Contributing direct evidence of moderate or low quality; h, Contributing direct evidence of high or moderate quality; i, Contributing direct evidence of high or low quality; j, Contributing direct evidence of high quality; k, Contributing direct evidence of high or very low quality; l, Contributing direct evidence of low or very low quality; m, Contributing direct evidence of low quality; n, Severe imprecision; o, Severe inconsistency | | | | | | | | | | | | | | | | | |

# **Table S8** Adverse events of the interventions

| **Intervention** | **Adverse events** | | | | | | | | |  |
| --- | --- | --- | --- | --- | --- | --- | --- | --- | --- | --- |
|  | **Hypoglycemia** | **Edema** | **Urinary tract infection** | **Nocturia and polyuria** | **Cutaneous symptom** | **Urticaria** | **Hyperphagia** | **Appetite suppression** | **Gastrointestinal reactions** | **Heart failure** |
| **Liraglutide** | 1/30[57] 0/29[55] 1/31[60] |  |  |  |  |  |  | 22/29[55] | 9/30[57] 3/29[55] 8/31[60] | 0/30[57] |
| **Exenatide** | 3/38[56] |  |  |  |  |  |  |  |  |  |
| **Pioglitazone** | 2/30[57] 0/33[59] 1/34[72] | 5/33[59] 2/34[51]  2/19[58] | 0/33[59] 0/34[51]  0/19[58] | 0/34[72] | 0/33[59] | 1/34[72] | 2/33[59] 2/34[51] |  | 2/30[57] 1/34[51] | 1/30[57] |
| **STA-TRE** | 1/37[72] |  |  | 0/37[72] |  | 0/37[72] |  |  |  |  |
| **Dapagliflozin** | 0/32[59] | 0/32[59] | 1/32[59] |  | 2/32[59] |  | 2/32[59] |  |  |  |
| **Empagliflozin** | 1/35[72] |  |  | 1/35[72]  3/23[65] |  | 1/35[72]  0/23[65] |  |  |  |  |
| **Metformin** | 2/29[55]  1/30[60] |  |  |  |  |  |  | 6/29[55] | 4/29[55] 1/30[60] |  |
| **Insulin glargine** | 6/38[56]  3/30[60] |  |  |  |  |  |  |  | 2/30[60] |  |
| **Gliclazide** | 2/27[55] |  |  |  |  |  |  | 0/27[55] | 0/27[55] |  |
| **Ipragliflozin** |  | 0/32[51] | 3/32[51] |  |  |  | 2/32[51] |  | 1/32[51] |  |
| **Glimepiride** | 2/33[59]  2/20[64] |  |  | 0/20[64] | 0/33[59]  0/20[64] |  | 1/33[59] |  |  |  |
| **Tofogliflozin** | 0/20[64] | 0/21[58] | 1/21[58] | 1/20[64] | 1/20[64] |  |  |  |  |  |
| **Sitagliptin** |  |  |  | 0/21[65] |  | 1/21[65] |  |  |  |  |

# **Continued Table S8** Adverse events of the interventions

| **Intervention** | **Adverse events** | | | | | | | | |
| --- | --- | --- | --- | --- | --- | --- | --- | --- | --- |
|  | **Severe weakness and fatigue** | **Ddiabetic foot ulcer** | **Headache** | **Diarrhea** | **Abdominal distention** | **Nasopharyngitis** | **Vaginal candidiasis** | **Balanoposthitis** | **Joint pain** |
| **Liraglutide** |  |  |  | 4/29[55] 1/31[60] | 3/29[55] |  |  |  |  |
| **Exenatide** |  |  |  |  |  |  |  |  |  |
| **Pioglitazone** | 0/34[72] | 1/34[72] | 1/34[51] | 0/34[51] |  | 1/34[51] | 0/34[51] |  |  |
| **STA-TRE** | 0/37[72] 0/20[52] | 1/37[72] |  |  |  |  |  | 0/20[52] | 0/20[52] |
| **Dapagliflozin** |  |  |  |  |  |  |  |  |  |
| **Empagliflozin** | 1/35[72] 1/22[52] | 0/35[72] |  |  |  |  |  | 1/22[52] | 1/22[52] |
| **Metformin** |  |  |  | 10/29[55] 0/30[60] | 5/29[55] |  |  |  |  |
| **Insulin glargine** |  |  |  | 0/30[60] |  |  |  |  |  |
| **Gliclazide** |  |  |  | 0/27[55] | 0/27[55] |  |  |  |  |
| **Ipragliflozin** |  |  | 1/32[51] | 1/32[51] |  | 0/32[51] | 1/32[51] |  |  |
| **Glimepiride** |  |  |  | 0/20[64] |  |  |  |  |  |
| **Tofogliflozin** |  |  |  | 1/20[64] |  |  |  |  |  |

# **Continued Table S8** Adverse events of the interventions

| **Intervention** | **Adverse events** | | | | | | | | |
| --- | --- | --- | --- | --- | --- | --- | --- | --- | --- |
|  | **Weight gain (>3%)** | **Itching in the genital area** | **Cystitis** | **Arthritis** | **Thirst** | **Constipation** | **Pharyngitis** | **Upper respiratory tract infection** | **Onychomycosis** |
| **Pioglitazone** | 6/19[58] |  |  |  |  |  |  |  |  |
| **Tofogliflozin** | 0/21[58] | 7/20[64] | 1/20[64] | 1/20[64] | 0/20[64] |  |  |  |  |
| **Glimepiride** |  | 0/20[64] | 0/20[64] | 0/20[64] | 1/20[64] |  |  |  |  |
| **Empagliflozin** |  |  |  |  | 2/23[65] | 1/23[65] | 1/23[65] | 1/23[65] | 1/23[65] |
| **Sitagliptin** |  |  |  |  | 0/21[65] | 0/21[65] | 0/21[65] | 0/21[65] | 0/21[65] |

# **Figure S1** Quality assessment of the included studies and risk of bias summary for RCTs and risk of bias graph for RCTs.


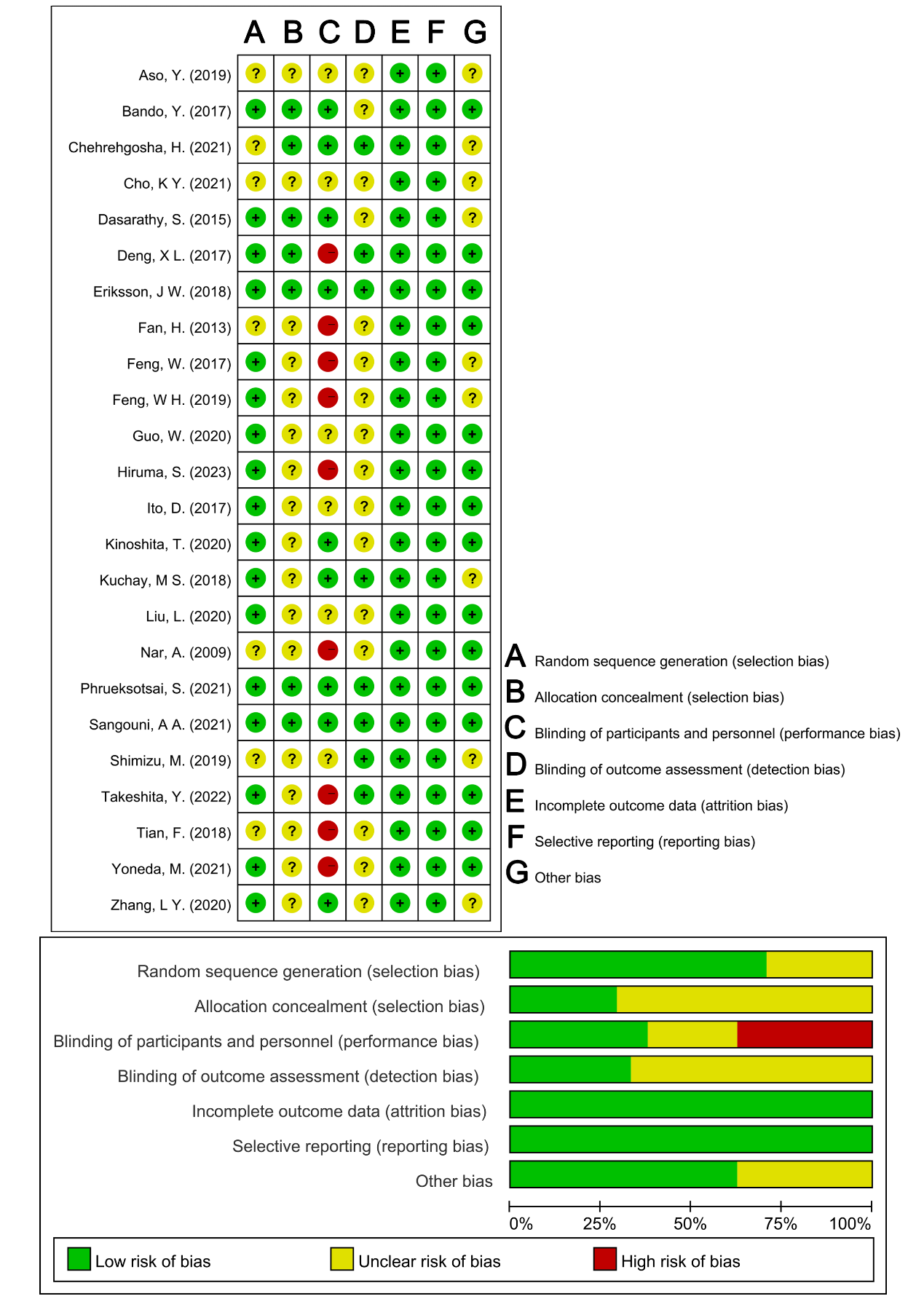


Individual risk of bias and overall risk of bias for each included study are visualised in green yellow and red representing low risk of bias unclear risk of bias and high risk of bias, respectively.

# **Figure S2** SUCRA ranking plot of intervention


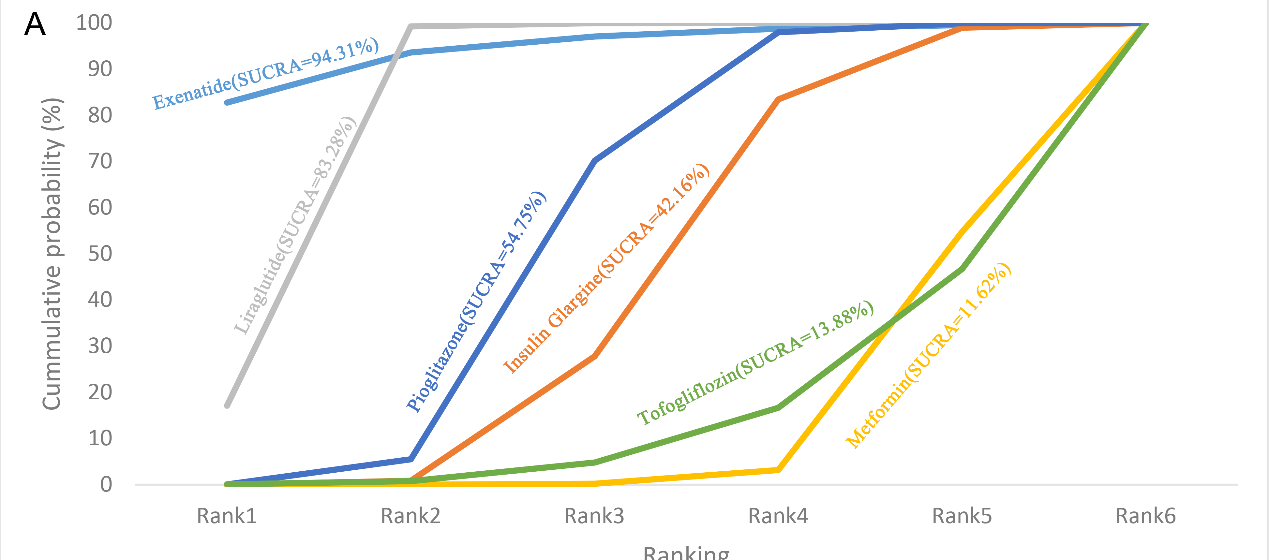


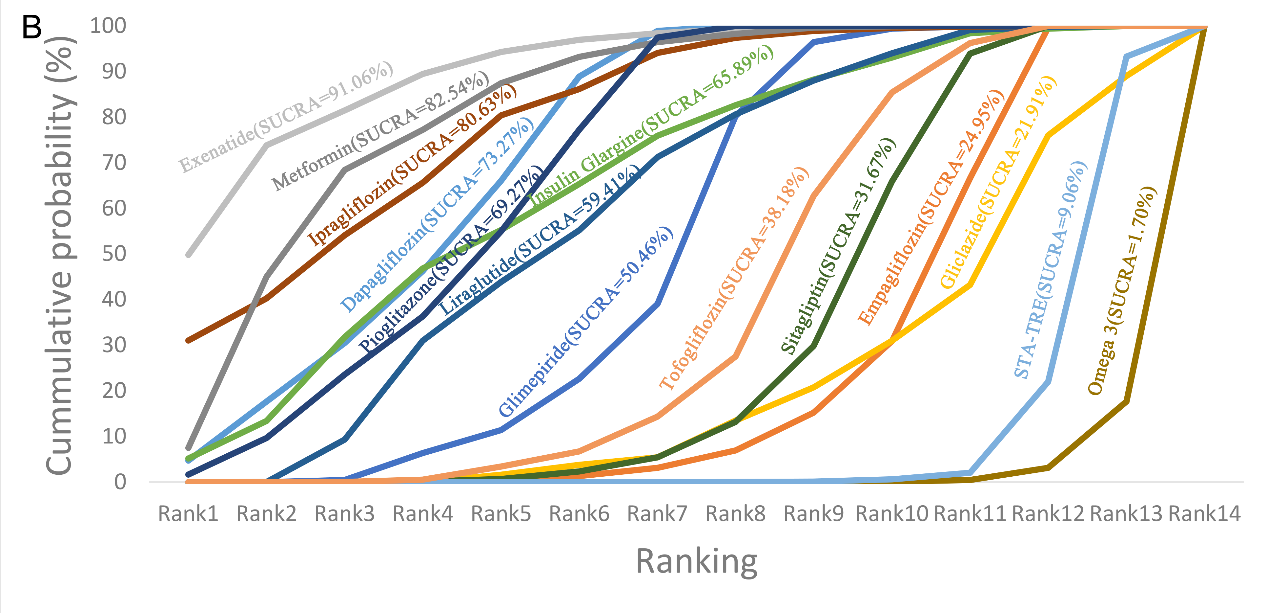


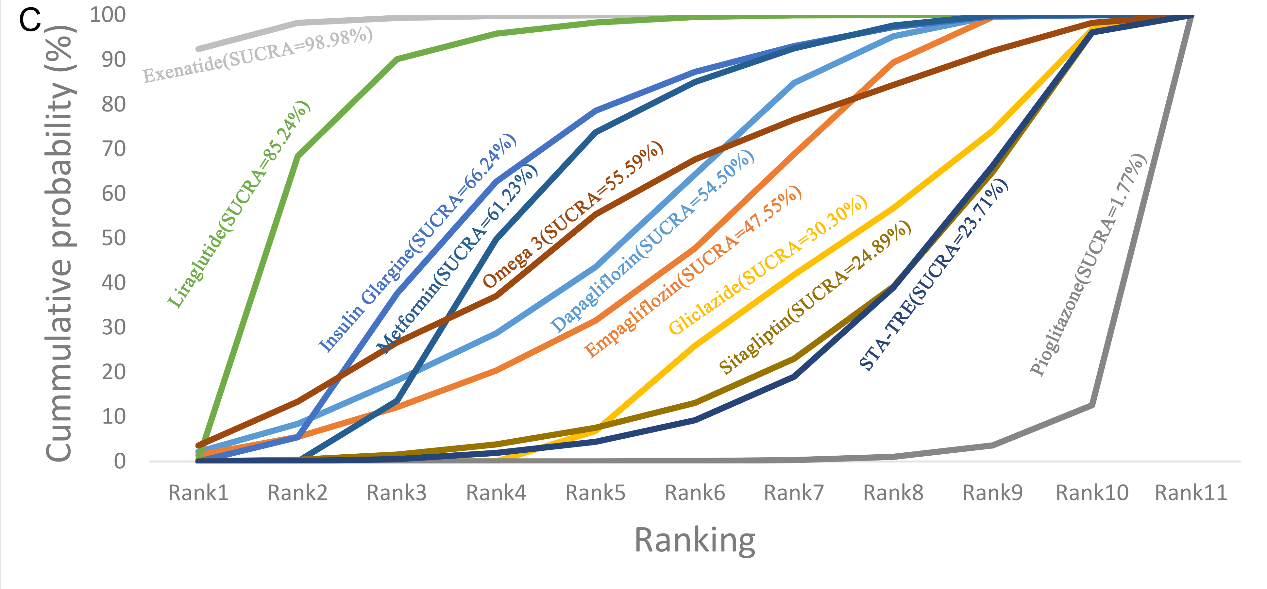


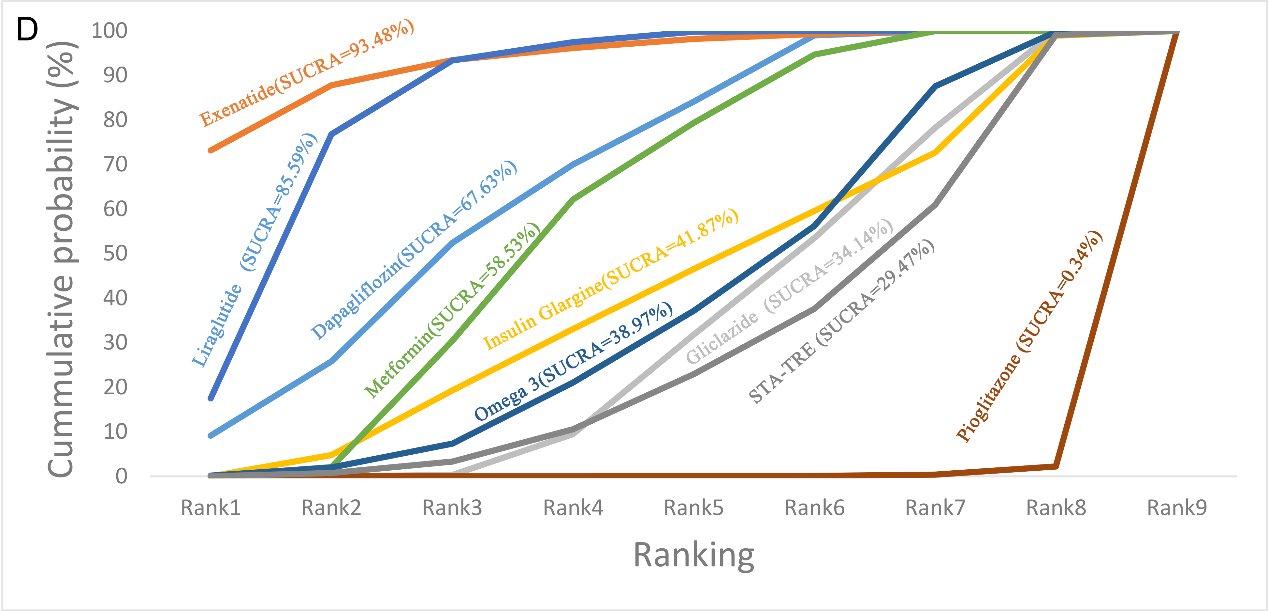


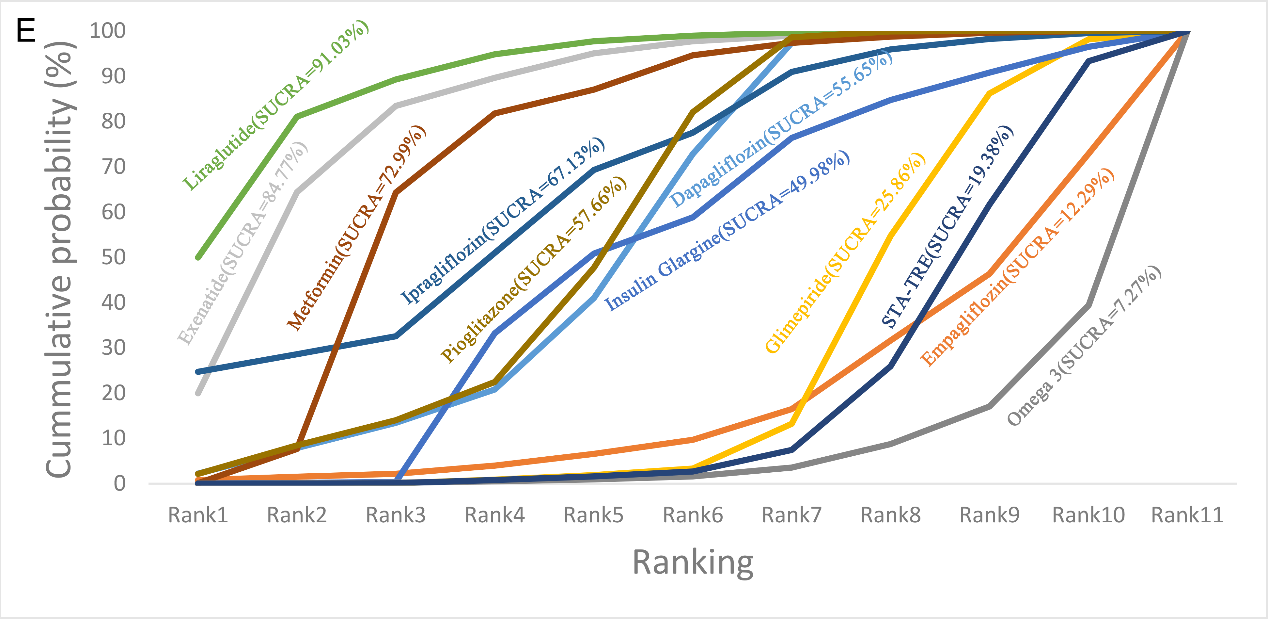


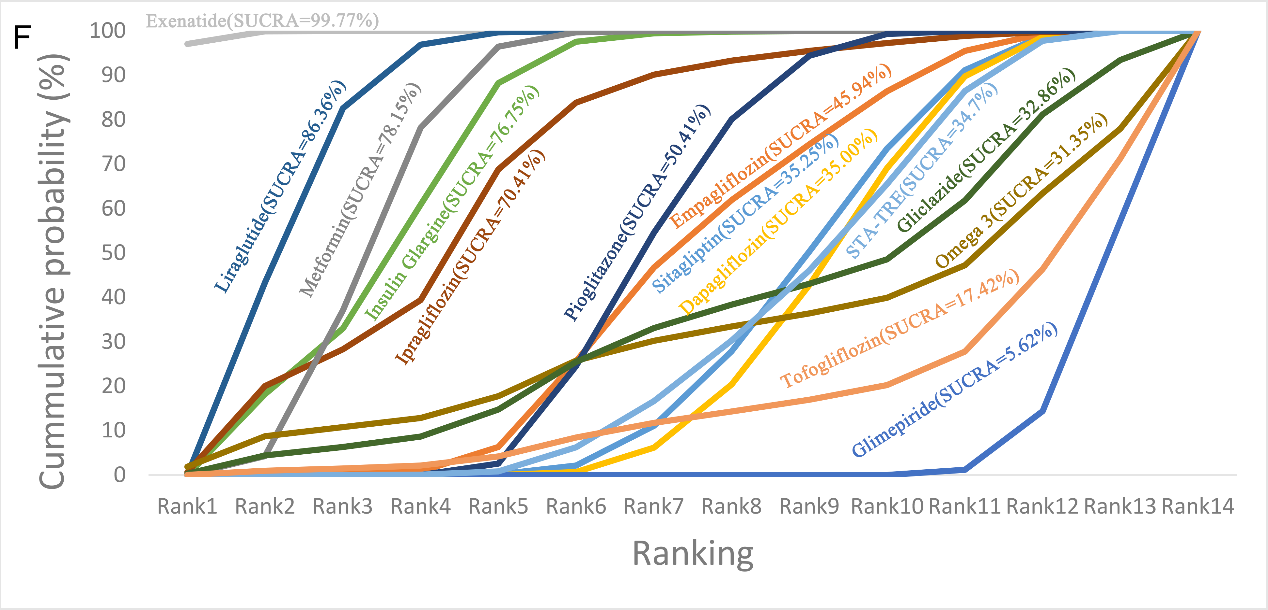


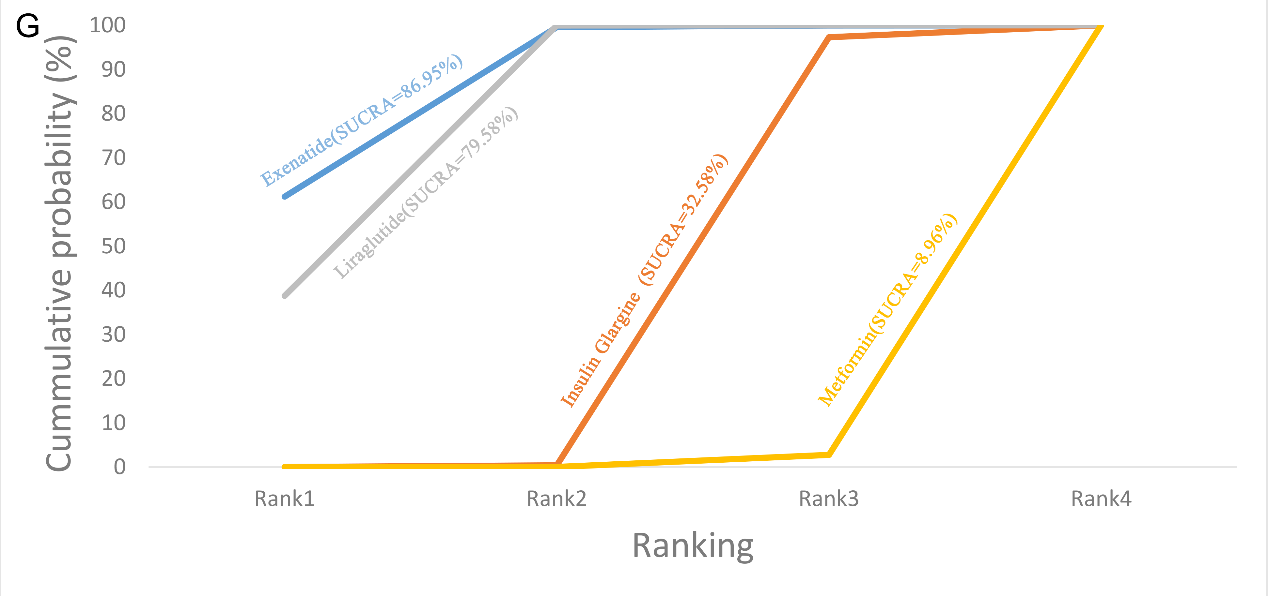


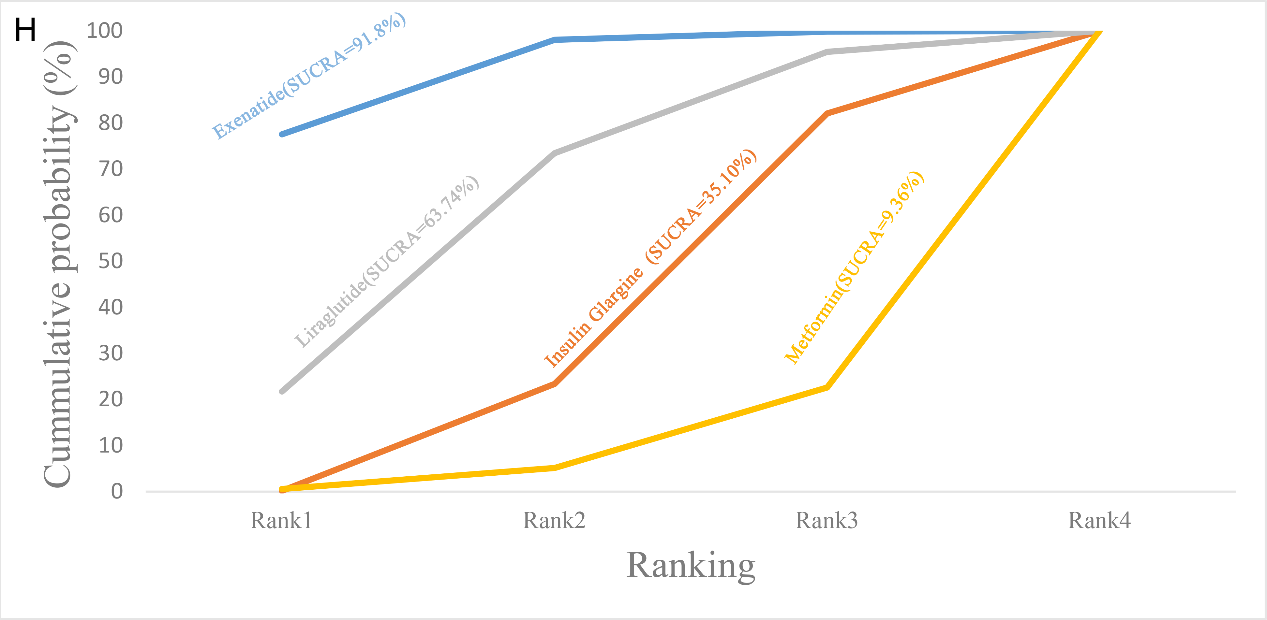


(A) Liver fat content; (B) HbA1c; (C) BMI; (D) Waist circumference; (E) Insulin resistance; (F) ALT; (G) VATA; (H)SATA. The ranking plot represents the cumulative probability of being the best intervention, second and third. x-axis shows the relative rankings and y-axis shows the cumulative probability of each ranking. the larger the SUCRA, the higher it is ranked.

# **Figure S3** League matrix table of important but not crucial results


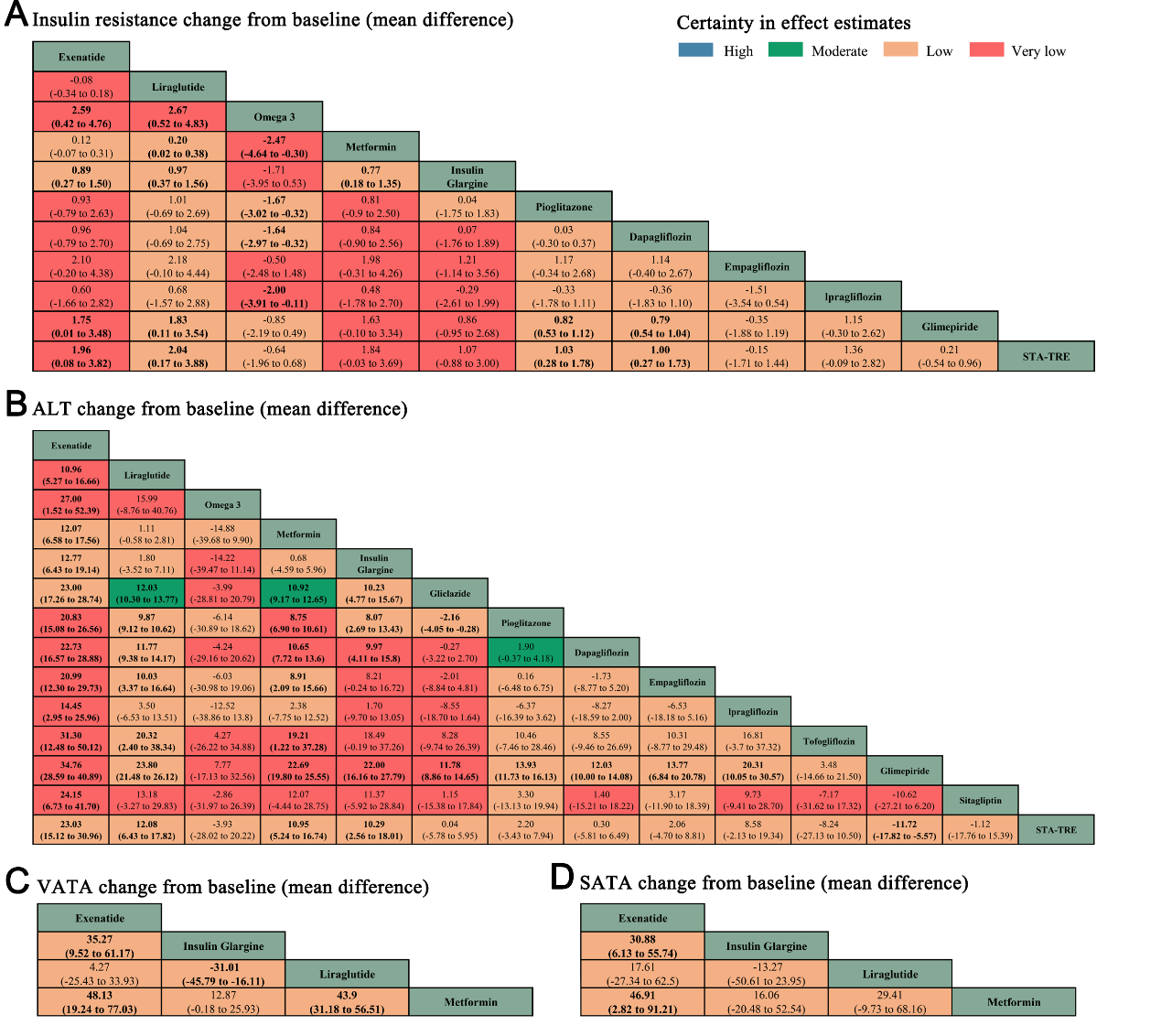


(A) Insulin resistance; (B) ALT; (C) VATA; (D) SATA.

# **Figure S4** Funnel plots and Egger's test


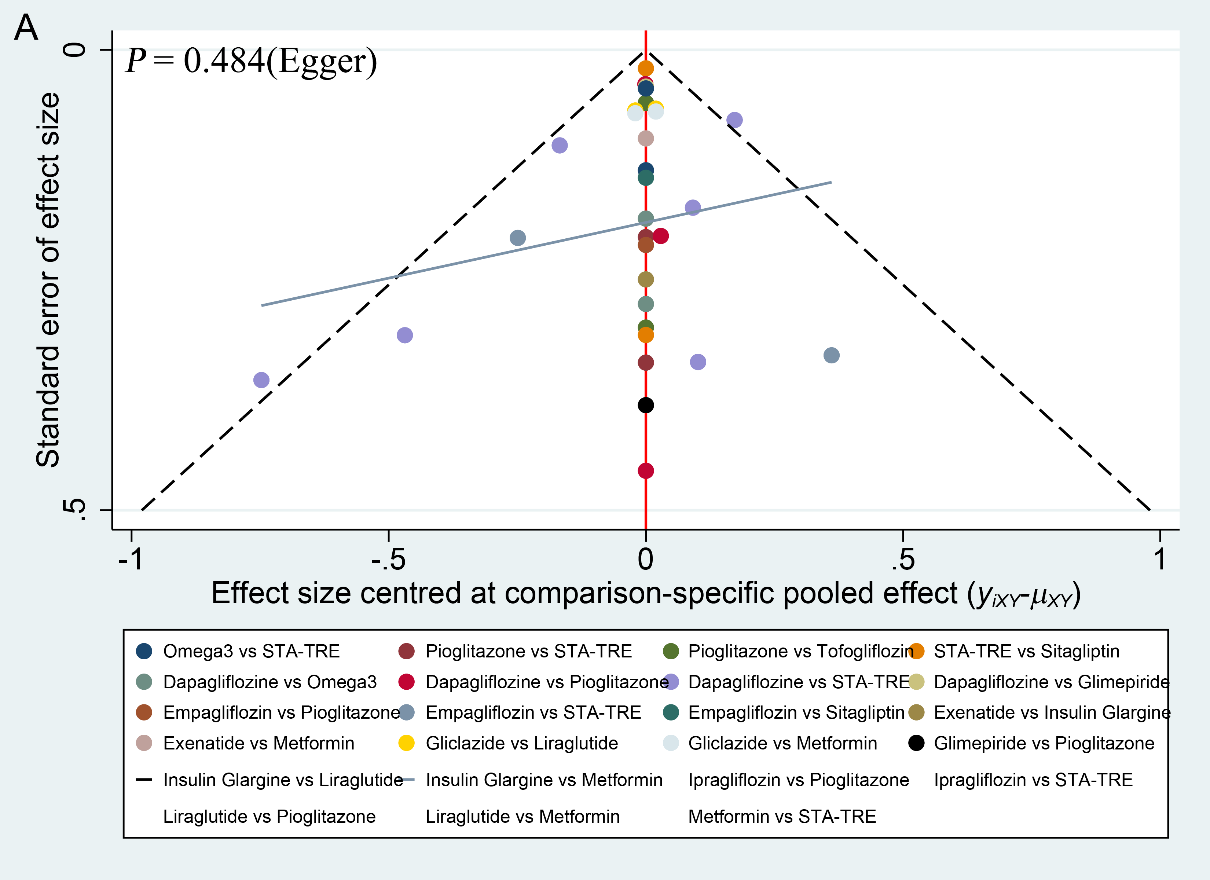


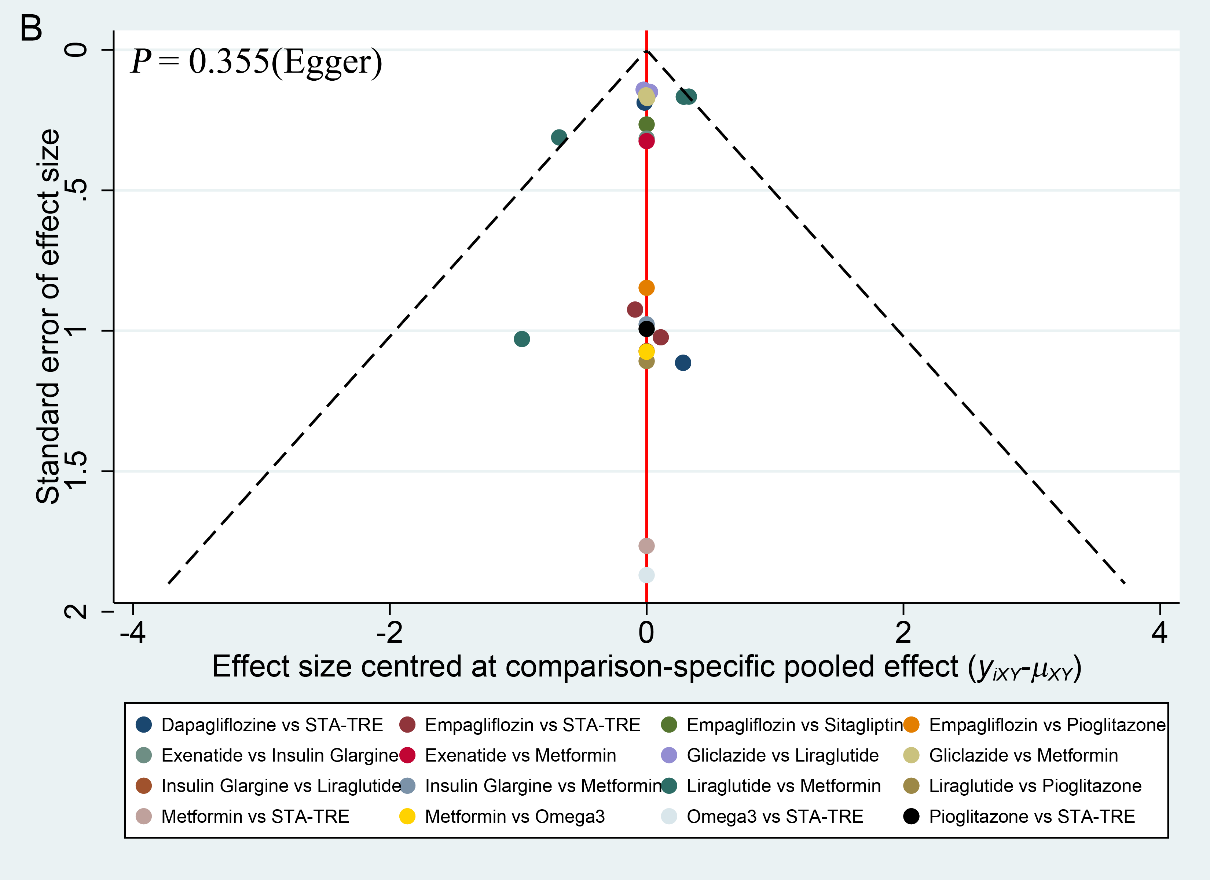


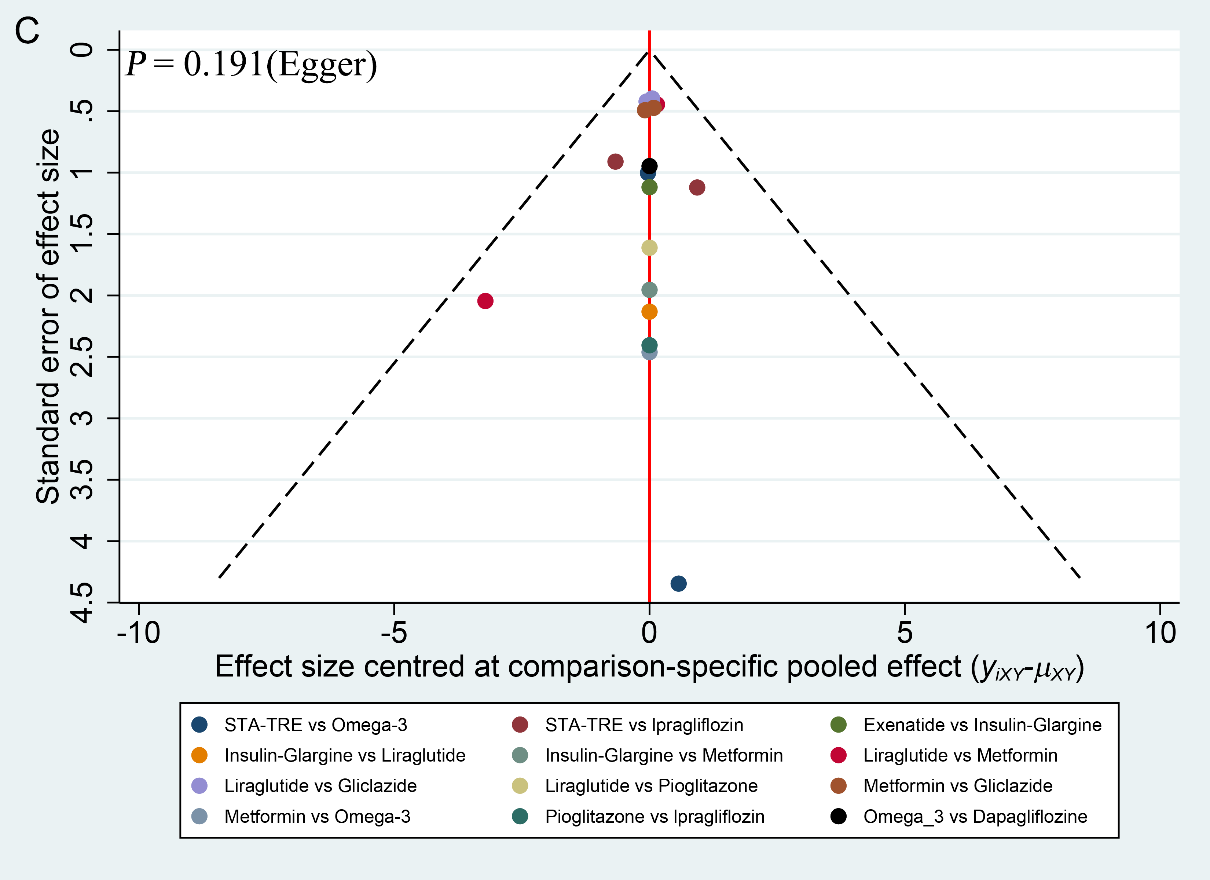


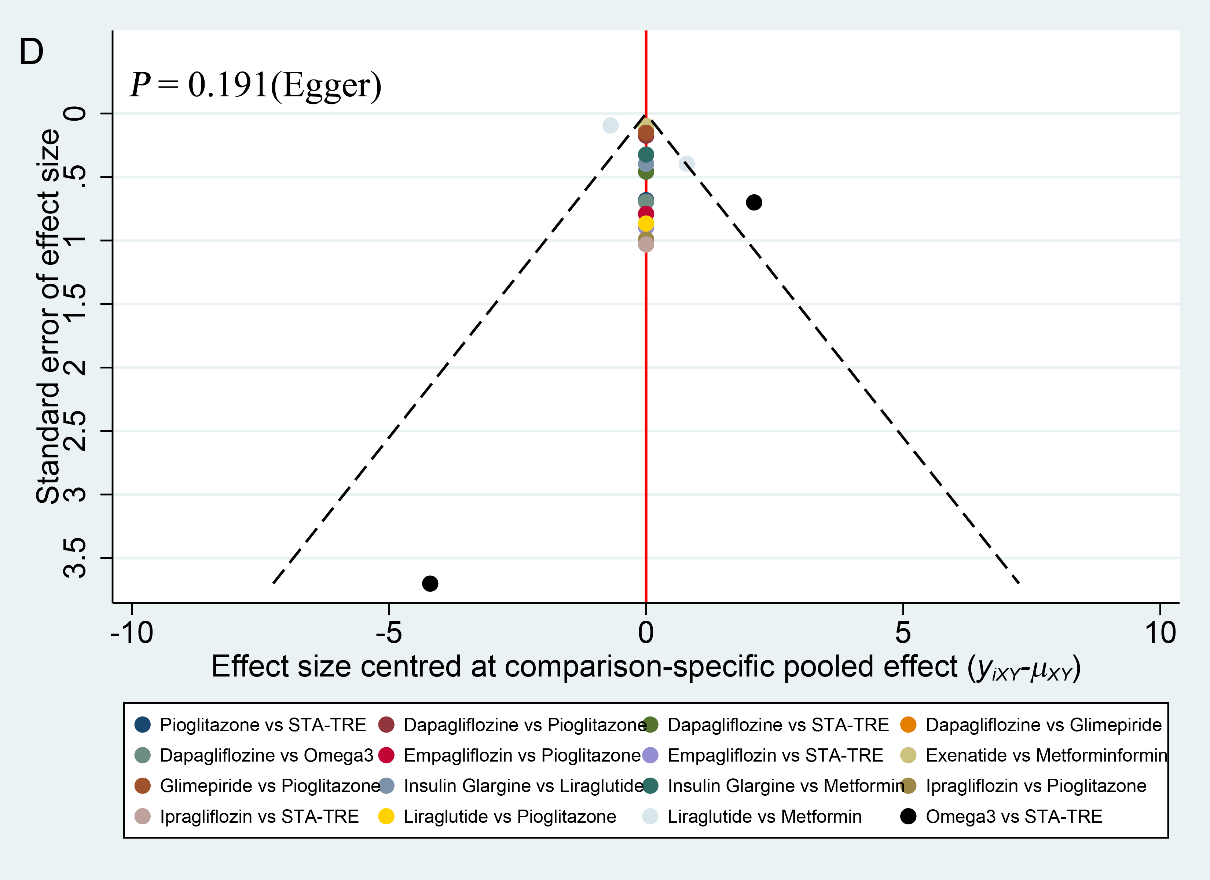


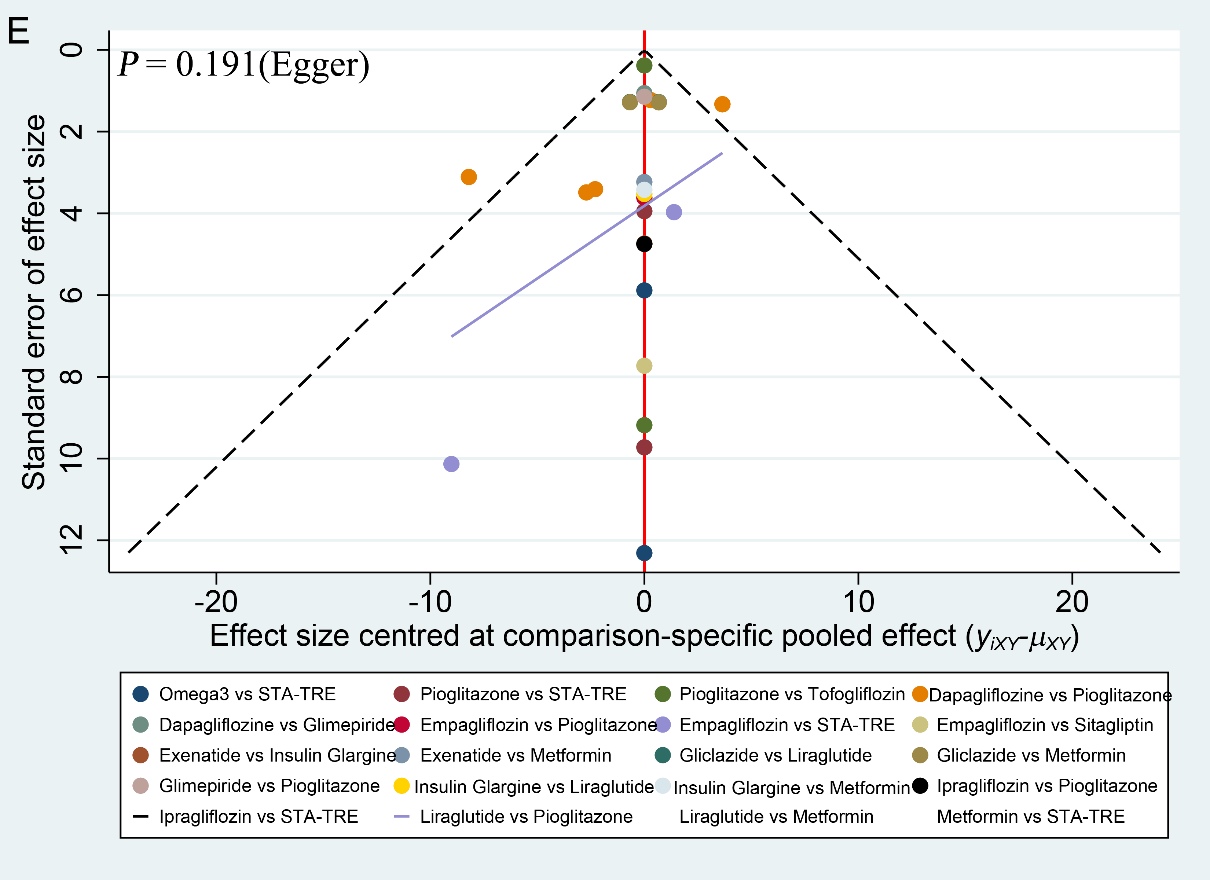


(A) HbA1c; (B) BMI; (C) Waist circumference; (D) Insulin resistance; (E) ALT

# **Figure S5** Forest plot for inconsistency testing


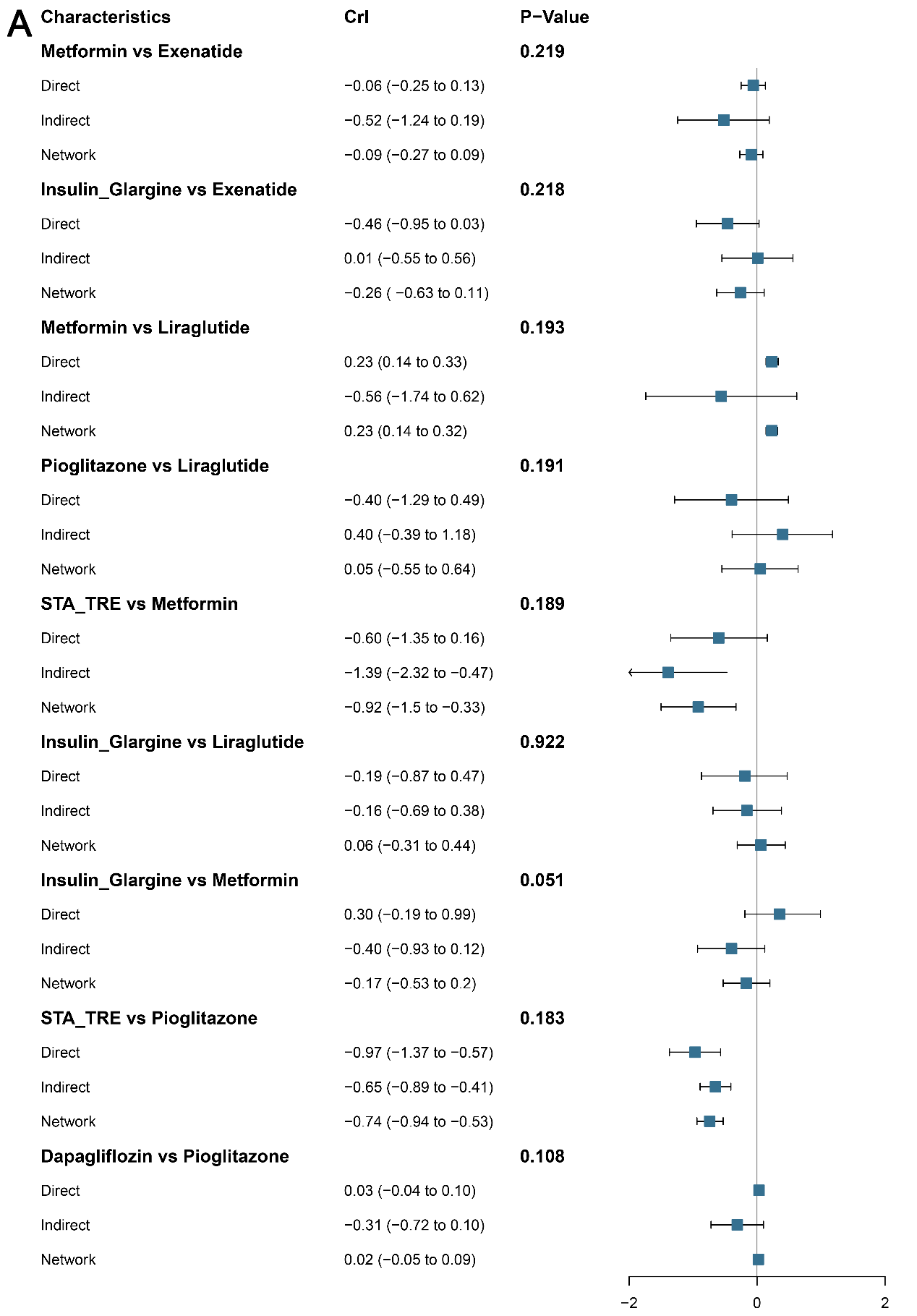


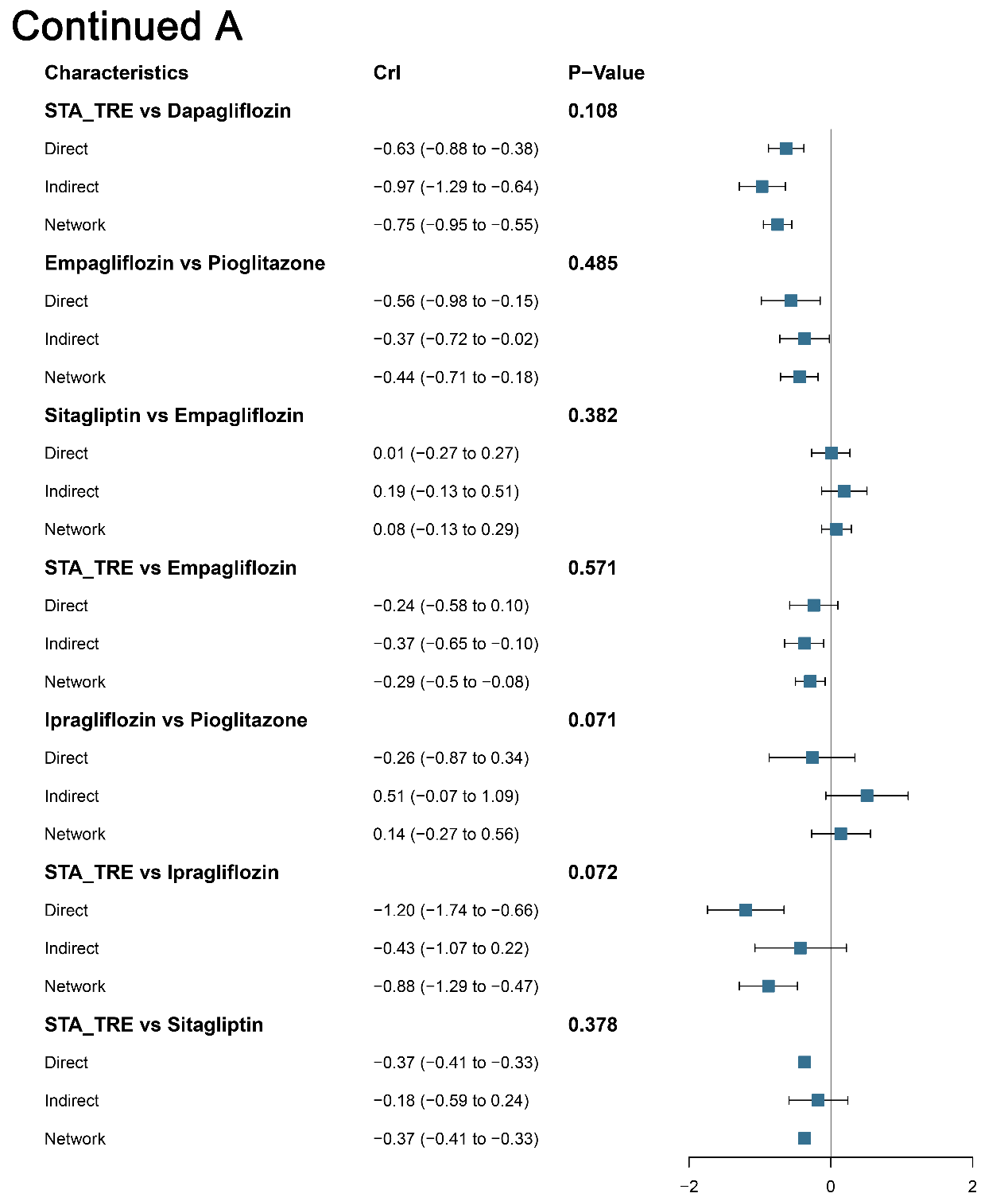


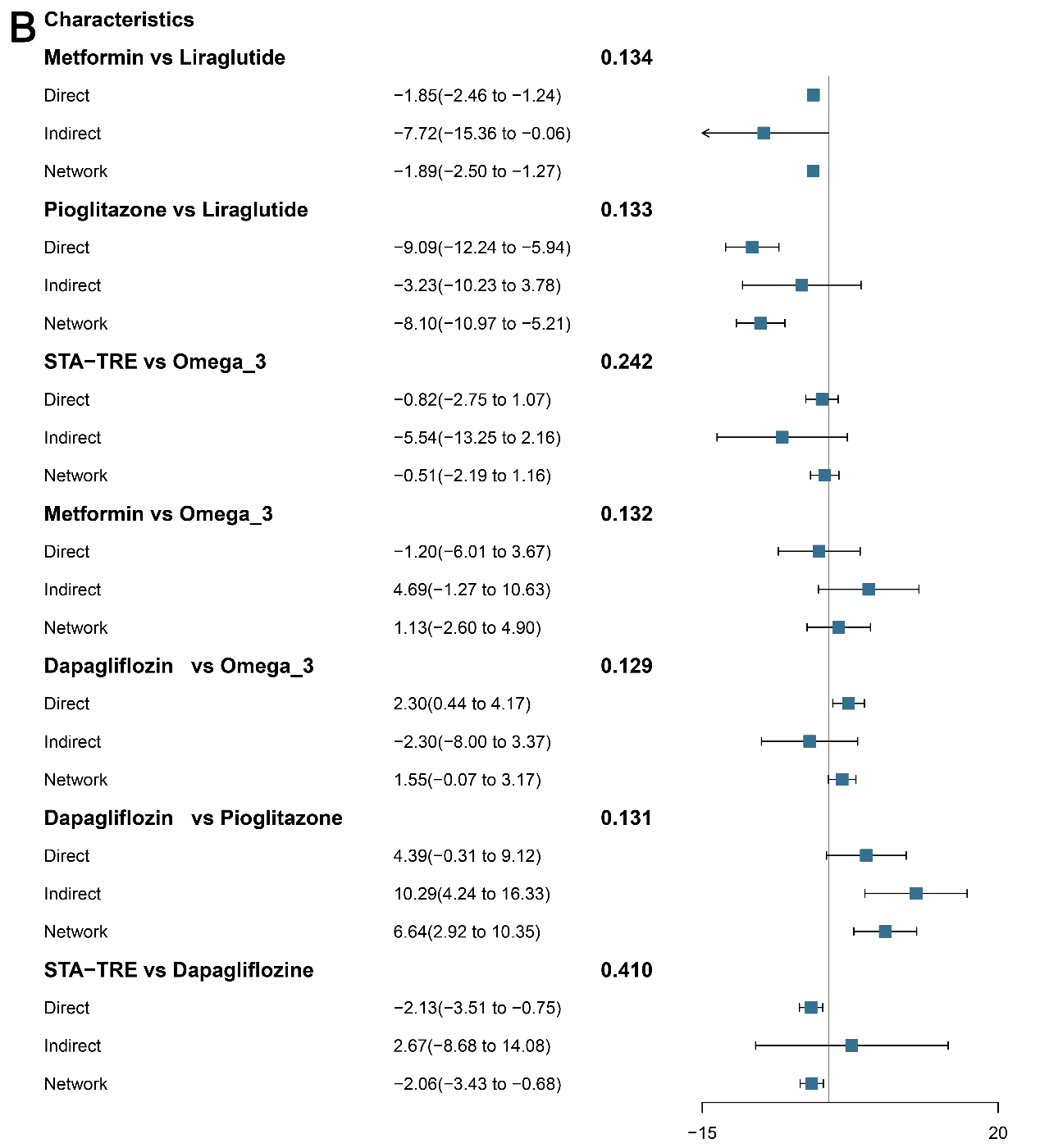


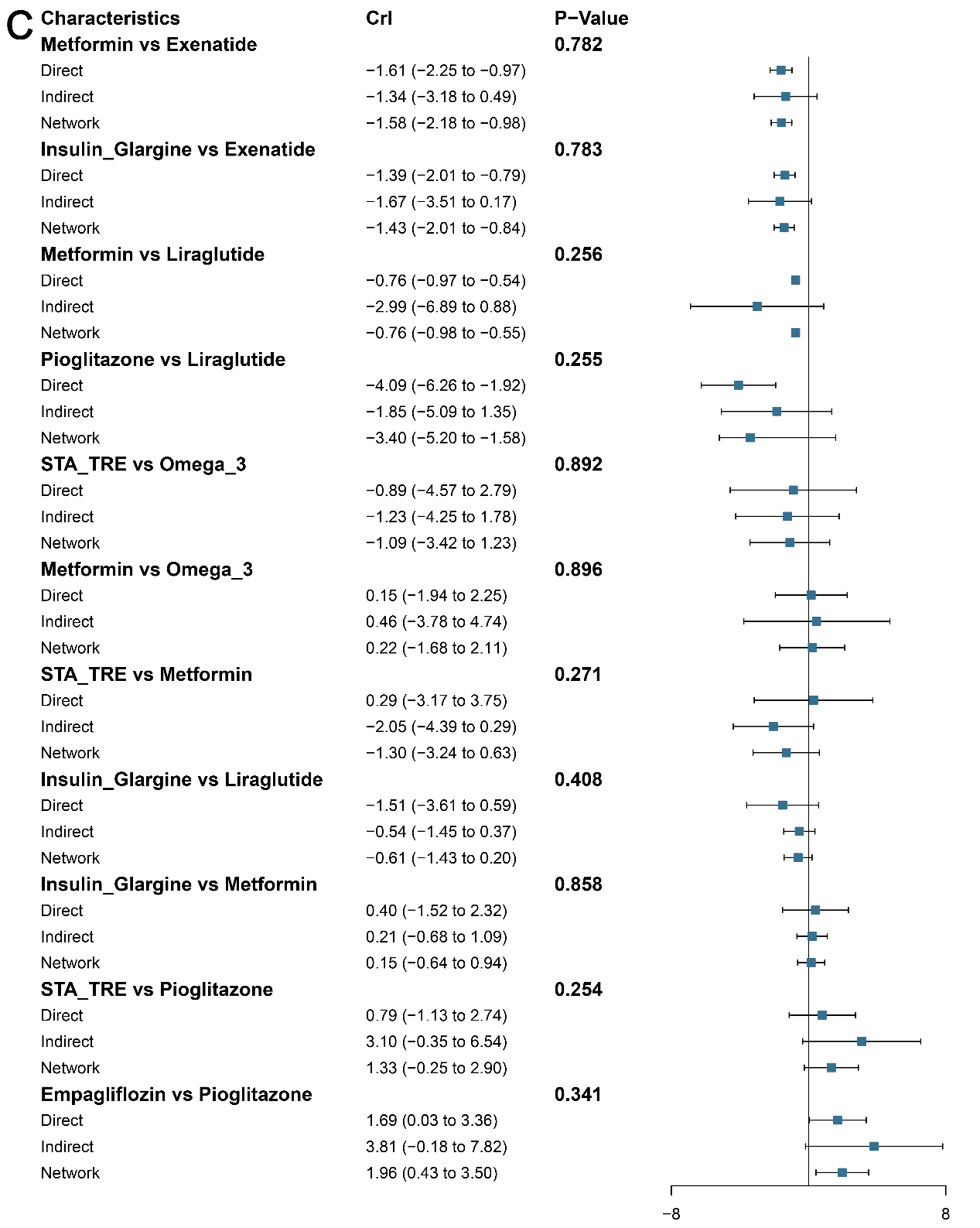


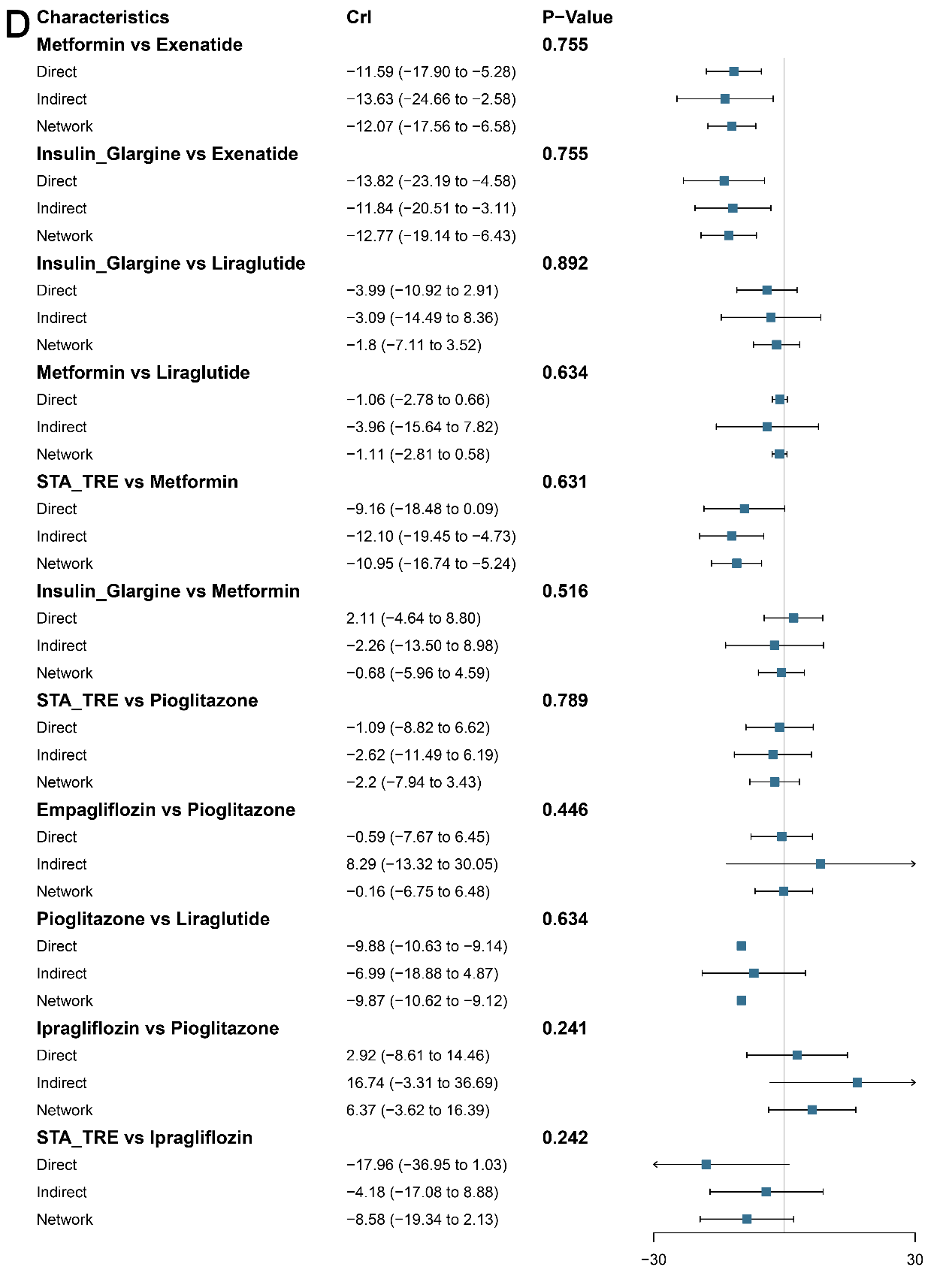


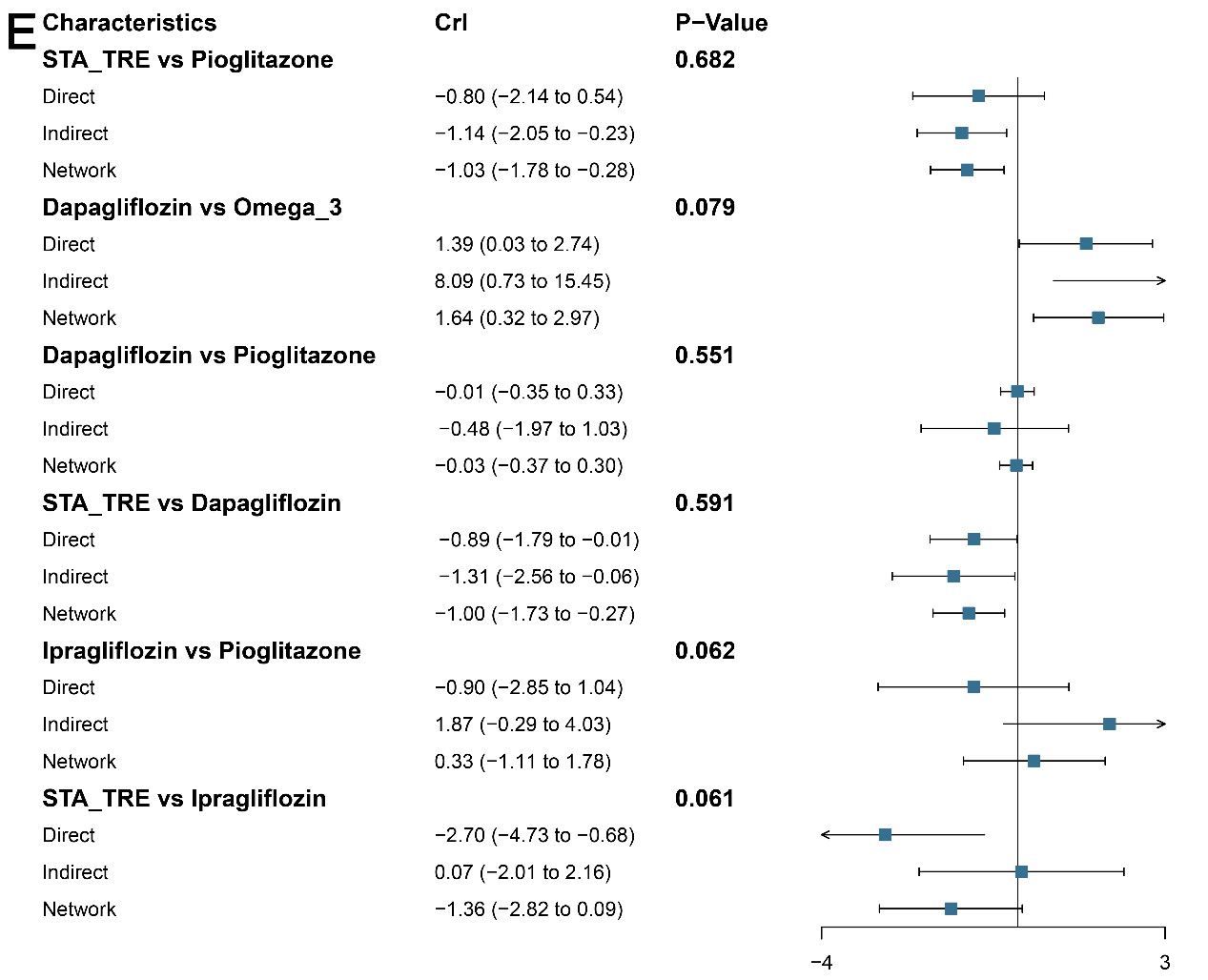


(A) HbA1c; (B) Waist circumference; (C) BMI; (D) Insulin resistance; (E) ALT.
